# A High Quality Draft Consensus Sequence of the Genome of a Heterozygous Grapevine Variety

**DOI:** 10.1371/journal.pone.0001326

**Published:** 2007-12-19

**Authors:** Riccardo Velasco, Andrey Zharkikh, Michela Troggio, Dustin A. Cartwright, Alessandro Cestaro, Dmitry Pruss, Massimo Pindo, Lisa M. FitzGerald, Silvia Vezzulli, Julia Reid, Giulia Malacarne, Diana Iliev, Giuseppina Coppola, Bryan Wardell, Diego Micheletti, Teresita Macalma, Marco Facci, Jeff T. Mitchell, Michele Perazzolli, Glenn Eldredge, Pamela Gatto, Rozan Oyzerski, Marco Moretto, Natalia Gutin, Marco Stefanini, Yang Chen, Cinzia Segala, Christine Davenport, Lorenzo Demattè, Amy Mraz, Juri Battilana, Keith Stormo, Fabrizio Costa, Quanzhou Tao, Azeddine Si-Ammour, Tim Harkins, Angie Lackey, Clotilde Perbost, Bruce Taillon, Alessandra Stella, Victor Solovyev, Jeffrey A. Fawcett, Lieven Sterck, Klaas Vandepoele, Stella M. Grando, Stefano Toppo, Claudio Moser, Jerry Lanchbury, Robert Bogden, Mark Skolnick, Vittorio Sgaramella, Satish K. Bhatnagar, Paolo Fontana, Alexander Gutin, Yves Van de Peer, Francesco Salamini, Roberto Viola

**Affiliations:** 1 IASMA Research Center, San Michele all'Adige, Trento, Italy; 2 Myriad Genetics Inc, Salt Lake City, Utah, United States of America; 3 454 Life Sciences Corporation, Branford, Connecticut, United States of America; 4 Roche Diagnostics Corporation, Roche Applied Science, Indianapolis, Indiana, United States of America; 5 Amplicon Express Inc., Pullman, Washington, United States of America; 6 Technology Park Lodi, Lodi, Italy; 7 Department of Plant Systems Biology, VIB, Gent University, Gent, Belgium; 8 Department of Biological Chemistry, Padova University, Padova, Italy; 9 Department of Computer Science, Royal Holloway, University of London, Egham, Surrey, United Kingdom; University of California at Davis, Genome Center, United States of America

## Abstract

**Background:**

Worldwide, grapes and their derived products have a large market. The cultivated grape species *Vitis vinifera* has potential to become a model for fruit trees genetics. Like many plant species, it is highly heterozygous, which is an additional challenge to modern whole genome shotgun sequencing. In this paper a high quality draft genome sequence of a cultivated clone of *V. vinifera* Pinot Noir is presented.

**Principal Findings:**

We estimate the genome size of *V. vinifera* to be 504.6 Mb. Genomic sequences corresponding to 477.1 Mb were assembled in 2,093 metacontigs and 435.1 Mb were anchored to the 19 linkage groups (LGs). The number of predicted genes is 29,585, of which 96.1% were assigned to LGs. This assembly of the grape genome provides candidate genes implicated in traits relevant to grapevine cultivation, such as those influencing wine quality, via secondary metabolites, and those connected with the extreme susceptibility of grape to pathogens. Single nucleotide polymorphism (SNP) distribution was consistent with a diffuse haplotype structure across the genome. Of around 2,000,000 SNPs, 1,751,176 were mapped to chromosomes and one or more of them were identified in 86.7% of anchored genes. The relative age of grape duplicated genes was estimated and this made possible to reveal a relatively recent *Vitis*-specific large scale duplication event concerning at least 10 chromosomes (duplication not reported before).

**Conclusions:**

Sanger shotgun sequencing and highly efficient sequencing by synthesis (SBS), together with dedicated assembly programs, resolved a complex heterozygous genome. A consensus sequence of the genome and a set of mapped marker loci were generated. Homologous chromosomes of Pinot Noir differ by 11.2% of their DNA (hemizygous DNA plus chromosomal gaps). SNP markers are offered as a tool with the potential of introducing a new era in the molecular breeding of grape.

## Introduction

Grapes (67 million t; http://faostat.fao.org/site/336/DesktopDefault.aspx) and their derivatives have a large and expanding worldwide market. Grapes can be grown at latitudes from 50°N to 40°S and up to 3,000 meters above sea level, with almost 98% of grape vineyards planted with *Vitis vinifera* L. ssp. *sativa* cultivars of Eurasian origin. Ever since the development of wine-making in Iran between 5,440 and 5,000 B.C. [1], wine has been an important component of many cultures. It has been celebrated by the Ecclesiates, by Horace, Goethe, Jefferson and the Nobel laureate J. C. Cela. A traditional icon of the Mediterranean diet [2], the grape has more recently been extensively cultivated in the New World and its cultivation is now moving to Asia. Given grape's content of resveratrol, quercitin and ellagic acid, grape products may contribute to reducing the incidence of cardiovascular and other diseases [3].

*V. vinifera* ssp. s*ativa*, domesticated from the wild ssp. *sylvestris* [4], bears hermaphroditic self-fertilizing flowers. However, outbreeding by means of wind and insect pollination is the norm. As a result, cultivars are highly heterozygous and carry many deleterious recessive mutations [5]. Inbreeding depression is severe, so that sterility often ensues from the second or third generation of selfing. All wild *Vitis* species have 38 chromosomes (n = 19) and most interspecies hybrids are fertile [5]. The high chromosome number suggests a paleopolyploid state of the genome [6], an argument recently presented in the frame of a recent partial assembly of the grape genome [7] but still remaining controversial.

Grape has the potential to become a model organism for fruit trees. The species can be transformed [8] and micropropagated *via* somatic embryogenesis [9]. Compared to other perennials, the genome size is relatively small, 475 Mb [10], similar to rice (*Oryza sativa*, 430 Mb; [11]), barrel medic *(Medicago truncatula,* 500 Mb, http://medicago.org/) and black cottonwood poplar *(Populus trichocarpa*, 465 Mb; [12]).

In this paper we report a high-quality draft sequence of the grapevine genome. The genome is derived from the Pinot Noir clone ENTAV 115, a variety grown in a range of soils for the production of red and sparkling wines. The sequence provides information on the overall organization, gene content and structural components of the DNA of the 19 LGs of *V. vinifera*. The Sanger sequencing method was used to generate 6.5X coverage of the genome. This has been integrated with sequence reads generated by a scalable, highly parallel sequencing by synthesis (SBS) method with throughput significantly greater than capillary electrophoresis. The 4.2X coverage provided by SBS was crucial in identifying polymorphic sites and in closing most of the gaps between DNA contigs. This is the first project which utilizes both the longer Sanger and shorter SBS methods to determine the sequence of a large eukaryotic genome.

## Results and Discussion

### Sequencing and assembly

The DNA of *V. vinifera* was extracted from young shoots and sequenced and assembled using the whole genome shotgun (WGS) method. Two techniques were adopted: the Sanger dye primer sequencing of paired reads [13] and 454 (SBS) of unpaired reads [14], which provided 6.5X and 4.2X genome coverage respectively (see Materials and Methods).

In order to develop criteria for assembly, a preliminary experiment was conducted to assess heterozygosity: it was found to correspond to approximately 1 SNP per 0.1 Kb and 1 in/del per 0.45 Kb (see Text S1). The assembly program [11] was accordingly modified to accept a specified level of mismatches in overlapping sequences (details in Materials and Methods and in Text S1). The program also incorporated information on clone size, which ranged from 2 to 130 Kb (Table S1).

The assembly started with unique sequences and progressively included sequences with a higher degree of repetitiveness. To avoid merging repeats into a single genomic sequence, the overlapping unique sequence contigs were merged if the rate of polymorphism did not exceed 2% and if the resulting sequence coverage of the overlap did not exceed 150% of the average coverage (see Text S1). These criteria were modified so that contigs with many supporting links were merged. In most cases, this procedure produced a correct assembly.

Applying the procedure to about 6.6 M reads from Sanger sequencing, 90.6% of which represented paired clone ends, 211,374 initial seed contigs of unique sequences were generated. By using long clone links with non-repetitive clone ends, seed contigs were ordered into metacontigs (ordered assembly of contigs, referred to as supercontigs or scaffolds in other publications). After the sequences were merged into 120,000 contigs, data were combined with 4.2 genome-equivalents of SBS data. This helped to identify polymorphic sites and closed 25% of the remaining gaps between contigs. After removal of 10,847 contigs composed only of tandemly repeated sequences and disposal of 7,003 contigs shorter than 1,000 bp, the iterative assembly produced 58,611 contigs (Figure S1 and Table S2) corresponding to 530.9 Mb of genomic DNA. 44,179 of the 58,611 contigs were assembled into 2,093 metacontigs and the remaining 14,432 contigs were singletons. The final assembled sequences are deposited at the EMBL/Genbank/DDBJ databases (accession numbers: AM423240-AM489403, data released 2006-12-19). Metacontig data are available at http://genomics.research.iasma.it. The removed contigs represented mostly centromeric and rRNA gene sequences. Based on their read coverage, their sizes were estimated as 14.5 Mb and 16.3 Mb, respectively.

Cultivated *V. vinifera* is highly heterozygous. As a result, many of the resulting contigs were consensus sequences derived from an alignment of the two haplotypes. The set of Pinot Noir chromosome pairs included a considerable number of haplotype-specific gaps (sequences present in one haplotype but not in the other; on this issue see also the ‘Pinot Noir genome structure and evolution’ section). The total length of the 1,042,174 identified gaps corresponded to 48.9 Mb. In some chromosomal regions, the two alternative haplotypes were too different for the algorithm employed during assembly to combine them into a single contig. Such separated contigs corresponded to the hemizygous DNA (22,061 contigs with the total length of 65.1 Mb). The total size of the genome represented by different homologous chromosomes can be estimated as twice the length of the sequences represented by the two haplotypes merged into a consensus (416.8×2 = 833.6 Mb), plus the sequence length represented by hemizygous DNA and gaps, respectively 65.1 and 48.9 Mb. After including the centromeric and rRNA regions (14.5×2+16.3×2 = 61.6 Mb), the size of the diploid genome was subsequently estimated to be 1,009.2 Mb, which gives an average 504.6 Mb per haploid genome (Table 1).

**Table 1:** Number and sizes of assembled sequences in Mb.

	Number	Total length (Mb)	Contribution to the genome size (Mb)
Contigs with polymorphisms	36,550	465.7	
Heterozygous gaps		48.9	24.5^1^
Non-gap sequences		416.8	416.8
Contigs without polymorphisms	22,061	65.1	32.5^1^
Centromeric regions		14.5	14.5
rRNA clusters		16.3	16.3
**Total**	**58,611**		**504.6**

1 Gaps and hemizygous DNA represent regions which belong to only one of the two homologous pairs of Pinot Noir. Therefore, averaging them in the overall genome sequence is equivalent to reducing their total size by one half.

A region of 403,443 bp (preliminary experiment; see Text S1) was used to monitor the correctness of the assembly. Thirty four of the 37 contigs which mapped to the preliminary experiment sequence belonged to the metacontig assembled from the full genome sequence and were in the correct order. The remaining three contigs were not included because they contained repetitive clone links. Twenty two of the 36 boundaries between adjacent contigs were overlapping but not aligned due to large heterozygous inserts. The remaining 14 contig pairs corresponded to gaps: nine short gaps between 52 and 354 bp and five gaps larger than 500 bp. The largest gap (2.4 Kb) contained tandem repeats. Most of the gaps were associated to heterozygous inserts of repetitive elements. The total gap size, 8,067 bp, corresponded to about 2% of the region considered.

### Metacontig integration into the genetic map

The next phase of the assembly involved positioning metacontigs in the genome using a genetic map developed at the Istituto Agrario di San Michele all'Adige (IASMA). Genetic mapping was based on 94 individuals derived from a F_1 _Syrah X Pinot Noir cross where the latter was the pollen donor. The map contained 1,006 markers [15], which were used both to anchor BAC contigs to a physical map (http://genomics.research.iasma.it) and to order metacontigs along linkage groups (LGs).

A set of 799 additional SNP markers was developed based on polymorphic sites identified in contigs and was used to anchor and orient metacontigs to LGs. This genetic map included 1,767 molecular markers arranged in 19 LGs covering 1,276 cM (Figure S2; http://genomics.research.iasma.it). The SNP-based markers were also helpful in merging the adjacent metacontigs not previously merged because of repetitive or low-quality links between them.

**Figure 1:**
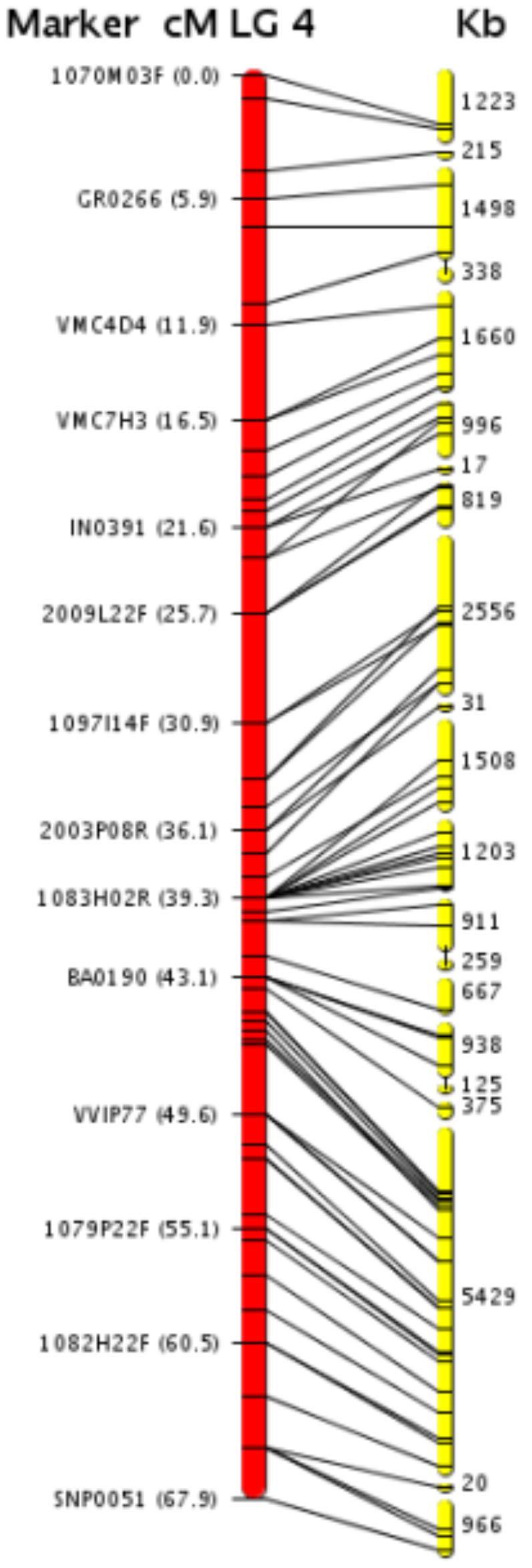
*V. vinifera* genomic metacontigs anchored to the LGs. *V. vinifera* genomic metacontigs (yellow bars) positioned along LG 4 of the Syrah x Pinot Noir genetic map. The map was assembled according to Cartwright et al. [113]. On the left are marker names and positions, in centimorgans, from Troggio et al. [15] (http://genomics.research.iasma.it). Most metacontigs were anchored to the map using markers with unique sequence locations: SSRs, BAC-end sequences and SNP-based markers derived from either ESTs or assembled sequences of the two haplotypes of the Pinot Noir genome. Metacontigs without bridge markers were anchored based on their association to other metacontigs (details in Materials and Methods). Approximate size in Kb of each metacontig is indicated on the right. Gaps separating metacontigs are of undefined size.

Integration of the DNA sequence and genetic map of LG4 is shown in Figure 1 (other LGs are in Figure S2). Table 2 summarizes the state of metacontig anchoring to the genetic map. The 2,093 metacontigs covered 477.1 Mb of genomic DNA. Of these, 435.1 Mb were anchored to the 19 LGs and 81.1% of these were oriented by two or more genetic markers (see Text S1). The smallest LG is covered by 26 metacontigs, the largest by 21 metacontigs. The order of markers established by meiotic recombination-based methods was almost co-linear with the metacontigs. In total, 82% of the genomic sequence was mapped to LGs. Most of the unmapped sequences were contained in 1,696 short metacontigs and singleton contigs with multiple tandem repetitive sequences. The assembly of metacontigs and facilitation of their placement on the genome using a genetic map avoided issues related to physical mapping.

**Table 2:** Correspondence, based on 1,356 markers, between the draft genome sequence of *V. vinifera,* presented in this paper, and the most advanced genetic map produced at IASMA.

Linkage group	Anchoring markers (no.)	cM	Metacontig (no.)	Size (Kb)	Contigs (no.)	SNP/Kb (no.)
1	79	78.1	14	26,109	2,222	3.9
2	75	52.3	21	18,582	1,676	4.3
3	49	49.5	19	18,967	1,522	3.4
4	71	67.9	21	25,533	2,097	3.9
5	62	67.0	16	21,708	1,672	3.5
6	74	75.6	10	20,950	1,833	4.4
7	92	94.7	19	32,087	2,812	4.1
8	98	75.9	26	27,023	2,418	4.5
9	52	53.9	20	18,263	1,795	3.7
10	73	81.5	18	24,862	2,321	4.6
11	62	67.7	23	18,722	1,719	4.4
12	71	70.2	22	20,676	1,839	4.2
13	86	71.9	26	26,447	2,373	4.2
14	60	62.1	20	22,360	1,394	2.9
15	55	48.3	26	18,867	1,857	4.0
16	63	52.5	27	21,046	2,449	4.4
17	52	62.5	15	17,344	1,452	4.3
18	104	95.1	21	31,342	2,436	4.0
19	78	51.5	33	24,260	2,023	3.5
**Total**	**1,356**	**1,278.2**	**397**	**435,146**	**37,910**	**4.0**

The genetic map is an extension of the map of Troggio et al. [15] and contains 1,767 markers. Metacontigs were assigned to the 19 LGs of grape based on the localization of DNA sequences underlying the markers present in the genetic map. LGs are numbered according to the International Grapevine Genome Program (www.vitaceae.org; [128]). Average SNP frequency in metacontigs anchored to 19 LGs of *V. vinifera* are listed for each LG.

### Gene annotation and gene content

Five quality levels were adopted for transcript assignment (see Materials and Methods): i) transcripts confirmed by tentative consensus sequences (TCs) and gene predictions (8,110); ii) transcripts confirmed by TCs aligned to the genome (8,160) and among transcripts not confirmed by TC; iii) the retained transcripts predicted at the exon level by different methods (4,028); iv) transcripts which were positive in gene prediction methods with differences at the exon level but with correct gene boundaries (308); v) transcripts which were found by different methods with contrasting results: only genes encoding proteins with significant similarities to known proteins were accepted (8,979). In total 29,585 genes were predicted. Grape gene content is comparable to Arabidopsis (26,819) and markedly different compared with rice (41,046) and poplar (45,555) genomes.

**Figure 2:**
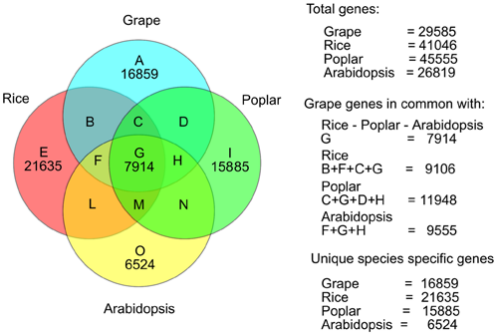
Comparison of four plant genomes based on gene homology. All genes were compared each other as all-*vs-*all similarity searches using BLAST. Genes predicted for poplar, Arabidopsis and rice are respectively from www.genome.jgi-psf.org; www.arabidopsis.org; www.tigr.org. Grape gene estimates have been carried out on 58,611 assembled contigs. Genes of similar length with over 60% of similarity alignments at protein level were considered homologous using BLOSUM62 matrix [123]. The frequencies of sequences shared among species are reported on the right.

Gene annotation followed a consensus approach. More than 79% of the genes predicted for the grape genome were annotated. Conserved putative grape genes were searched by the BLAST program with rice, poplar and Arabidopsis as references. A decision tree was implemented and used to carry this out. Sets of gene clusters with different levels of similarities among species as well as unique and putative species-specific genes were built. Using strict rules for homology determination, the subset of grape specific genes amounted to 16,859 (Figure 2).

Functional classification of the predicted genes was carried out by an automatic procedure. The manually revised final classification (Figure S3) shows the functional classes and their percentage in the gene set. Putative grape-specific genes were not characterized by a particular annotation profile or by relative abundance in the functional classes. A slight numerical difference in favour of grape was noted for genes related to lignin biosynthesis and to berry specific pectins. These metabolic pathways are less significant in Arabidopsis and poplar respectively. Genes relative to disease resistance and wine quality are discussed in further detail below.

### Disease resistance genes

Resistance to parasites in plants is controlled by the non-host and gene-for-gene pathways [16]. The non-host type was discovered only recently [17], [18]. The gene-to-gene pathway is frequently present in cultivated plants displaying dominant resistance genes, responsible for the initiation of signal transduction leading to deployment of defense mechanisms [19]. The majority of R proteins contain a nucleotide binding site (NBS) and a carboxy-terminal leucine-rich repeat (LRR) domain. The NBS is part of a conserved domain acting as a molecular switch for the signal transduction. The LRR is credited with recognition specificity akin to an antibody-like detector of pathogen effectors [20]. At the N-terminus NBS-LRR proteins carry either the coiled coil (CC) domain or a domain homologous to the Toll/Interleukin-1 Receptor (TIR, [21]), allowing classification of NBS genes into two groups, the CC-NBS-LRR, present in all angiosperms, and the TIR-NBS-LRR, specific to dicotyledonous species [22].

**Figure 3:**
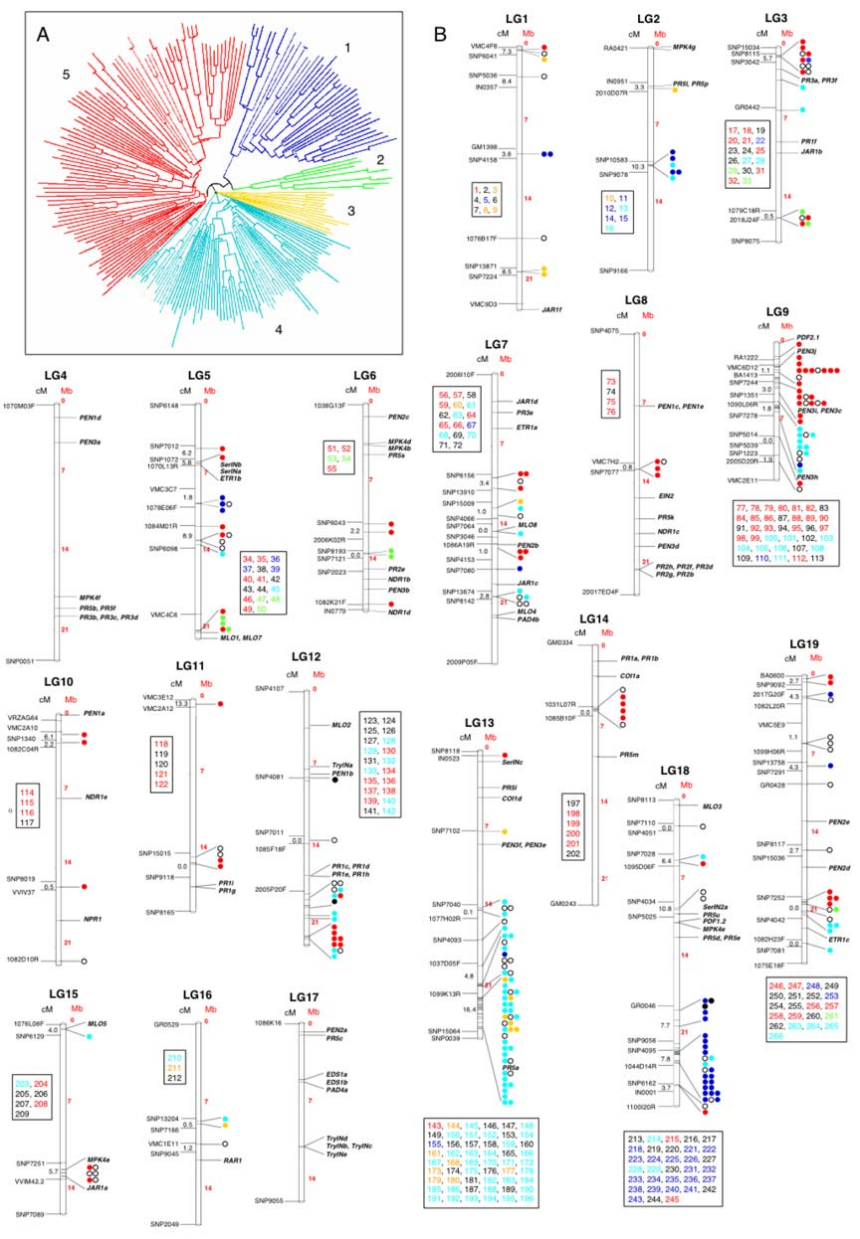
Chromosomal organization of disease resistance genes of *V. vinifera*. A) Phylogenetic analysis of NBS-LRR protein sequences of *V. vinifera* present in Pinot Noir. The phylogeny of these genes is based on a distance-matrix neighbour-joining analysis (Clustal X, [124]; bootstrap of 1000) after alignment of sequences by TCoffee (version 5.05, [125]). The phylogenetic clades, in general, correspond to the classification based on protein domains (however, see text and Table S3). B) Genes assigned to LGs are represented by dots. Their gene number is specified in LG-specific insets and in Table S3. NBS clades (see A above) contain mainly genes of the following classes: (1) TIR-NBS-LRR in blue; (2) CC-NBS-LRRa in green; (3) CC-NBS-LRRb in yellow; (4) NBS-LRR in cyan; (5) CC-NBS-LRR in red. Other resistance genes, belonging to NBS and TIR-NBS groups, are represented by the open and filled dots, respectively. Resistance-related genes different from NBS genes are shown in black. The size of each LG is given in Mb (on the right), whereas markers of the genetic map ([15] and http://genomics.research.iasma.it) are shown on the left, together with the interval in cM between the two closest markers in each gene cluster.

Based on resistance domain analyses, the grape genome was found to contain 341 NBS genes (Figure 3 and Table S3), whereas 207 were found in Arabidopsis [21] and 398 in poplar [12]. The 233 NBS genes which contain the LRR domain can be grouped in 5 major clades (1 to 5 in Figure 3A). The clades were comprised of CC-NBS-LRR, the dictot-specific TIR-NBS-LRR and their truncated structures as follows: (1) mainly TIR-NBS-LRR; (2) and (3) mainly CC-NBS-LRR; (4) mainly NBS-LRR; and (5) CC-NBS-LRR. The CC-NBS-LRR group included 84 genes in grape, 51 in Arabidopsis and 119 in poplar, while the TIR-NBS-LRR group included 37 genes in grape, 64 in poplar and 83 in Arabidopsis. In addition, the grape NBS gene family included 5 truncated TIR-NBS genes, 112 truncated NBS-LRR genes and 103 genes characterized only by the NBS domain (Table S3).

Besides NBS genes, the grape genome contains several signalling components of plant disease response which are encoded by genes *EDS1*, *PAD4*, *COI1*, *MPK4, JAR1, ETR1* and *NDR1,* known to be recruited by resistance gene products (Table S3). The *NPR1* gene, a regulator of the systemic acquired response to pathogens [23], is present in one copy in grape and in Arabidopsis, but has five copies in poplar. Likewise, *RAR1* and *EIN2* are present in single copies in the grape genome.

Genes encoding the pathogenesis-related proteins (PRs, [24]) include nine copies of *PR-1*, eight of *PR-2*, five of *PR-3*, one copy of *PDF1*, one of *PDF2*, and several copies of *PR5* and protease inhibitor-like genes (Figure 3B and Table S3).

In addition, the grape genome contains eight genes similar to the *MLO* gene for mildew resistance in barley, compared to the 15 *MLO-like* genes known for Arabidopsis [25]. MLO proteins belong to a large family of seven-transmembrane domain proteins specific to plants, encoded by genes homologous to barley *MLO* [25]. *MLO* recessive alleles confer an effective resistance against mildew pathogens. Furthermore, the powdery mildew non-host resistance-related genes *PEN1*, *PEN2* and *PEN3* [17], [18] were found in 5, 5 and 10 copies, respectively.

In grape, the disease-related genes represent a significant part of the genome. In spite of this, many grape varieties, including Pinot Noir, are susceptible to several fungi, such as grey mould (*Botrytis cinerea*), downy mildew [26] and powdery mildew [27], which have to be kept under control by heavy fungicide treatments. The failure to mount an effective defense response is probably due to a defective pathogen recognition. It is known that NBS-LRR genes are undergoing diversifying selection [28], e.g., variation in the sequence of the Arabidopsis gene *RPS2* shows a signature consistent with pathogen-stimulated selection [29]. Moreover, the extent of variation in the activity of NBS-LRR genes may have been affected by balancing selection [30]–[33]. Grape alleles of the same resistance genes did not co-evolve in the presence of the agriculturally most important grape pathogens [34]. Indeed, allelic variation due to SNPs present in functional resistance domains was associated with the phenotypic divergence between resistant and susceptible genotypes only when susceptible *V. vinifera* and resistant non*-vinifera* clones were considered [34]. In addition, the long time interval necessary for the grape to complete one generation, together with its vegetative propagation, makes it difficult to match the evolutionary rates of microbial or insect pests, which in vineyards are boosted by massive use of chemicals [35]. Such detailed knowledge of the grape genome will serve to accelerate the development of genetic strategies to counter crop loss due to dynamic and genetically diverse pathogens.

The TIR-NBS-LRR genes are preferentially located in LG 18, the CC-NBS-LRR genes in LGs 9 and 13 and the truncated NBS genes in LGs 12 and 13 (Figure 3B). The NBS genes are also organized in clusters and superclusters. As noted in Arabidopsis [36], each cluster may include NBS genes of different phylogenetic lineages, although they frequently consist of tandem repeats of the same gene. The heterogeneity of NBS clusters has been discussed and interpreted as a consequence of evolutionary events such as ectopic recombination, chromosomal translocation and gene-cluster remobilization. This type of genome evolution is difficult to explain other than in terms of a hypothesis where a positive selection for cluster complexity provides the basic materials for the generation of new resistance specificities [37].

Several clusters of NBS genes mapped to chromosomal regions where genetic resistance to fungal diseases, such as downy and powdery mildew, were previously assigned (Figure 3B). This included LGs 12 and 18 [38] and LGs 14 and 15 [27], [38], [39]. Thus, the genome sequence of grape indicates candidate NBS genes responsible for extant variation and provides a starting point for breeding grape varieties resistant to important pathogens.

### Phenolic and terpenoid pathways

Grape secondary metabolites, particularly polyphenols, have a strong influence on wine quality [40]. Most phenolics derive from phenylalanine via phenylalanine ammonia-lyase (PAL). They encompass a range of structural classes and biological functions and include lignins, phenolic acids such as hydroxycinnamic and hydroxybenzoic acids, and polyphenols such as flavonoids and stilbenes.

Flavonoids are the most common plant phenolics. In flowers and fruits they attract pollinators and seed dispersers and are particularly involved in UV-scavenging and disease resistance [41]. Flavonoids contribute to human health [42]. The flavonoid skeleton, synthesized by chalcone synthase (CHS), is converted to chalcones, flavanones, flavonols, flavanols, anthocyanins and proanthocyanidins (condensed tannins). In red grape, flavanols and anthocyanins are abundant, the latter accumulating mostly in the berry skin and the former in the seeds [43]. In the last decade considerable effort has been made in identifying and cloning grape flavonoid biosynthetic genes [44]–[47]. The grape genome sequence now offers the opportunity of compiling an exhaustive overview of the phenylpropanoid pathway.

**Figure 4:**
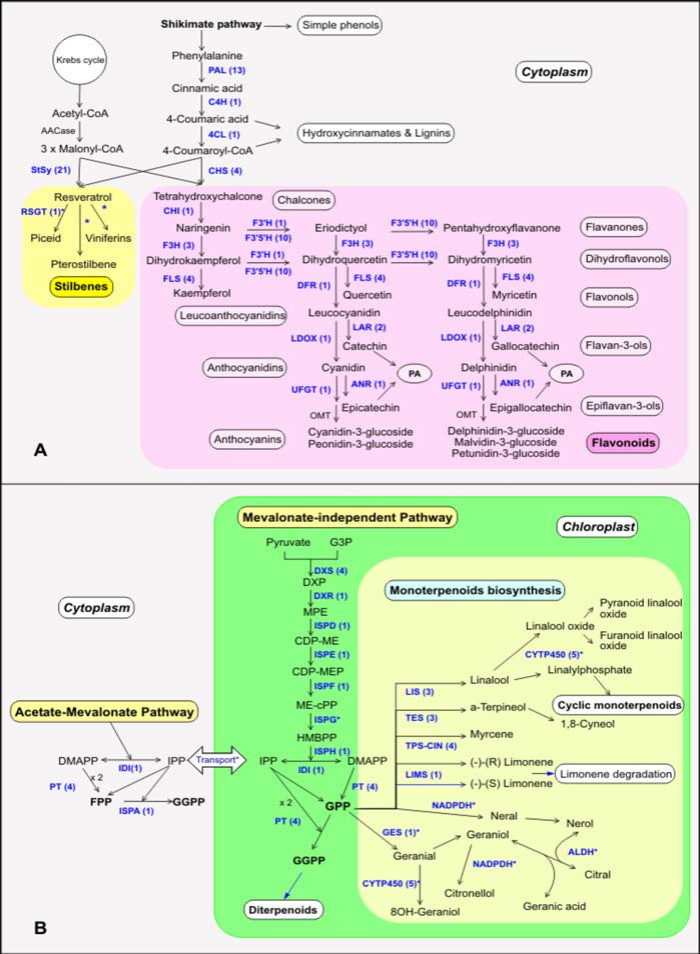
*V. vinifera* pathways for phenolic and terpenoid biosynthesis. A) *V. vinifera* general pathway for phenolics biosynthesis leading to stilbenes (C6-C2-C6) and flavonoids (C6-C3-C6). For each enzyme, the gene copy number is reported in brackets. Genes were identified by similarity search using BLAST where the references were the sequences of phenolic biosynthetic genes previously characterized in grape and in other plant species. Putative homologues and gene copy numbers were determined by comparing aligned amino acid sequences based on a threshold of 80% similarity between the grape sequences, and 60% similarity between grape and other species. For the large StSy, PAL and F3'5'H families, phylogenetic analysis was performed with MEGA4 package [126] after aligning with ClustalW [127]. The following enzymes involved in the pathway are shown: PAL, phenylalanine ammonia-lyase; C4H, cinnamate 4-hydroxylase; 4CL, 4-coumarate-CoA ligase; CHS, chalcone synthase; StSy, stilbene synthase; RSGT, resveratrol glucosyltransferase; CHI, chalcone isomerase; F3H, flavanone 3-hydroxylase; F3'H, flavonoid 3′-hydroxylase; F3'5'H, flavonoid 3′,5′-hydroxylase; DFR, dihydroflavonol-4-reductase; FLS, flavonol synthase; LDOX, leucoanthocyanidin dioxygenase; LAR, leucoanthocyanidin reductase; ANR, anthocyanidin reductase; UFGT, UDP-glucose:flavonoid 3-*O*-glucosyltransferase; OMT, *O*-methyltransferase; ACCase, acetyl CoA carboxylase. PA refers to proanthocyanidins. Enzymatic steps that have not been experimentally confirmed are marked with an asterisk (*). B) Steps of plastidic isoprenoid pathway and monoterpenoids biosynthesis. For each enzyme, the gene copy number is reported in brackets. Gene annotation was performed as described in Material and methods. Abbreviations: G3P, glyceraldehyde 3-phosphate; DXP, 1-deoxy-D-xylulose-5-phosphate; MEP, 2-C-methyl-D-erythritol 4-phosphate; CDP-ME, 4-diphosphocytidyl-2C-methyl-D-erythritol; CDP-MEP, 4-diphosphocytidyl-2Cmethyl-D-erythritol 2-phosphate; ME-cPP, 2C-ethyl-erythritol 2,4-cyclodiphosphate; HMBPP, 1-hydroxy-2-methyl-2-(E)-butenyl 4-diphosphate; IPP, isopentenyl pyrophosphate; DMAPP, dimethylallyl pyrophosphate. The enzymes in the pathway are indicated in blue: DXS, 1-deoxy-D-xylulose 5-phosphate synthase; DXR 1-deoxy-D-xylulose 5-phosphate reductase; ISPD, 4-diphosphocytidyl-2-C-methyl-D-erythritol synthase; ISPE, 4-diphosphocytidyl-2-C-methyl-D-erythritol kinase; ISPF, 2-C-methyl-D-erythritol 2,4-cyclodiphosphate synthase; ISPG, 2-C-methyl-D-erythritol 2,4-cyclodiphosphate synthase and ISPH 2-C-methyl-D-erythritol 2,4-cyclodiphosphate reductase (ISPG and ISPH are probably the same enzyme and convert directly MEcPP in IPP and DMAPP); ISPA, geranyltransferase; IDI, isopentenyl diphosphate delta-isomerase; PT, prenyltransferase; LIMS, limonene synthase; LIS, linalool synthase; GES, geraniol synthase; TES, α-terpineol synthase; TPS-CIN, myrcene/(E)-beta-ocimene synthase; CYTP450, cytochrome P450 hydroxylase; ALDH, aldehyde dehydrogenase; NADPDH, NADP dehydrogenase. Enzymatic steps that have not been experimentally confirmed are marked with an asterisk (*).

Gene predictions corresponding to all those genes known to encode enzymes of the pathway could now be found. These include C4H and 4CL (acronyms are explicated in note 1 of Figure 4A) which were not previously identified in grape. The majority of genes were organized in large (PAL, F3'5'H) or small (CHS, F3H, FLS, LAR) gene families, the remainder consisting of single copy genes (C4H, 4CL, CHI, F3'H, DFR, LDOX, ANR, UFGT) (Figure 4A; Table S4).

Within the phenylpropanoid pathway, relatively large gene families have been described for poplar compared to Arabidopsis [48]. Our results highlight some significant differences, such as the number of PAL and F3'5'H gene copies which were even greater in grape. In general, grape and poplar secondary metabolism exhibits a tendency toward gene family expansion. Conversely, in Arabidopsis all enzymes of the central flavonoid metabolism, except for FLS, are encoded by single genes [41]. This is consistent with the noted low metabolic investment in flavonoids of Arabidopsis, a species which reproduces without the need for insect pollination and has no perennial woody habit.

In grape, as in a few other species, the condensation of p-coumaroyl-CoA with malonyl-CoA gives rise to stilbenes via stilbene synthase (StSy; [49]). Among stilbenes, monomers and oligomers (viniferins) of resveratrol contribute to resistance to fungal pathogens [50]. Resveratrol has gained attention due to its alleged beneficial effects on human health [51]. Stilbene synthase belongs to a large family: the analysis of the grape genome predicts at least 21 copies. This number agrees well with a recent StSy sequence analysis in infected grape leaves [26] but it differs from the one predicted in the PN40024 grape genome sequence [7]. Most of these copies, as well as most PAL genes, are clustered in LG 16. Further, several peroxidase genes were predicted, some of which could participate in the formation of viniferins, as previously suggested [50]. Recently, a resveratrol glucosyltransferase putatively involved in piceid synthesis has been isolated and biochemically characterized in *V. labrusca* grape berry [52]. Our analysis revealed that its homolog in Pinot Noir (99% sequence similarity) is present as a single gene mapping on LG 3.

Terpenoids are among the most abundant and structurally diverse group of natural metabolites. Volatile and non-volatile terpenes are essential for plant growth and development (e.g., gibberellin phytohormones), but they are also key players in the interaction of plants with the environment [53]. The substrates for the biosynthesis of about 22,000 terpenes are isopentenyl diphosphate (IPP) and dimethylallyl diphosphate (DMAPP). The mevalonate (MVA) and the mevalonate-independent DOXP/MEP pathways are responsible for the synthesis of IPP and DMAPP in the cytosolic and plastidic compartments respectively [54]. DOXP/MEP is the dominant route for monoterpene biosynthesis in the grape berry [55]. Three prenyltransferases produce terpene precursors, prenyl diphosphates, geranyl diphosphate (GPP), farnesyl diphosphate (FPP) and geranylgeranyl diphosphate (GGPP). Terpene synthases (TPS) catalyze the formation of hemiterpenes [51], monoterpenes (C10), sesquiterpenes (C15) or diterpenes (C20) from the substrates DMAPP, GPP, FPP or GGPP respectively (Figure 4B).

All TPSs are similar in physico-chemical properties. Moreover, the close sequence relatedness of their genes prevents discrimination of their catalytic functions, supporting a rapid divergence of catalytic activity of closely related TPS genes [53]. Three classes of TPSs are described and only classes II and III are specific for the plant secondary metabolism [56]. Forty seven TPS genes participate in the secondary metabolism in poplar [12], while in grape only 35 TPSs were identified, a number close to the 32 found in Arabidopsis. In the grape genome, they are located mainly on LGs 9, 10 and 19 (class I TPs on LGs 7, 9, 10 and 19, Table S5).

Several higher plant genes of the terpenoid pathways have been cloned [57], but only a few of them had previously been identified in grape [58]. Having the complete sequence of the grape genome, 124 genes related to the terpenoid pathway were identified (Table S5). Of these, 110 were mapped to all LGs. Functionally, 24 are related to carotenoids, 24 to abscisic acid metabolism, 10 to gibberellin hormones, and 6 cover steps of the core terpenoids pathway: 5 prenyltransferases and 1 isopentenyl diphosphate delta-isomerase. For the MVA and non-MVA pathways, nine (4 DXS, DXR, ISPD, ISPE, ISPF, ISPH) and eight (2 AACT, HMGS, 3 HMGR, MK/MVK, MVD) putative genes were identified respectively.

Plant monoterpens are preferentially confined to specialized organs. They play an important role in defense as well as acting as allelopathic agents and attractants for pollinators [59]. In grape, monoterpenes contribute to wine free volatiles: typical components of the aroma-rich grape varieties are linalool, geraniol, nerol, citronellol and α-terpineol, which are stored in exocarps and vacuoles. Monoterpene biosynthesis has not yet been studied because several metabolic steps may take place without enzymatic catalysis. Moreover, the knowledge of mechanisms controlling monoterpene synthase activity is still largely incomplete. In the grape genome four monoterpene synthase genes were identified encoding linalool synthase, limonene synthase, myrcene synthase and α-terpineol synthase.

### Transcription factors

**Figure 5:**
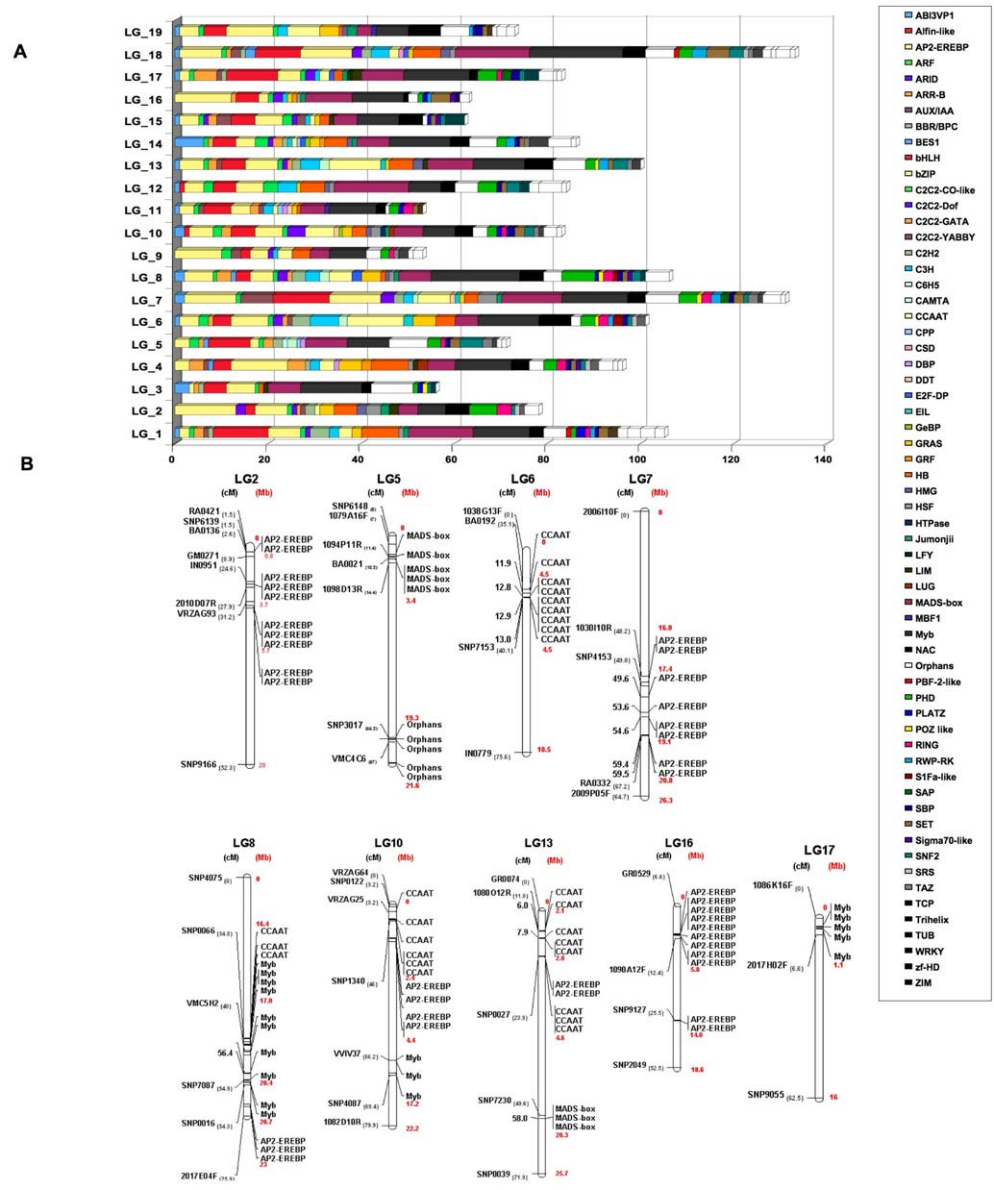
Distribution of transcription factors along the 19 *V. vinifera* LGs. A) Distribution of 1,617 transcription factors along the 19 *V. vinifera* LGs inferred from the positions of anchored metacontigs. Different colours of the histograms corresponds to the different TF classes. B) Distribution of transcription factor clusters over the grape genome. TF organization in LGs 2, 5, 6, 7, 8, 10, 13, 16, 17 is presented. For each LG, markers of the genetic map, developed by Troggio et al. [15] (see also http://genomics.research.iasma.it) are reported on the left together with the interval in cM between the two closest markers for each TF cluster. TF types are reported on the right.

In grape, 2004 TF genes were identified (Figure 5A and Table S6) which represent 6.7 % of the genome, similar to the 6% for Arabidopsis [60], 4.8 % for rice [61] and 6% for poplar [62]. Among the grape TF genes, 80.6% are present in marker-anchored metacontigs (Figure 5A).

**Figure 6:**
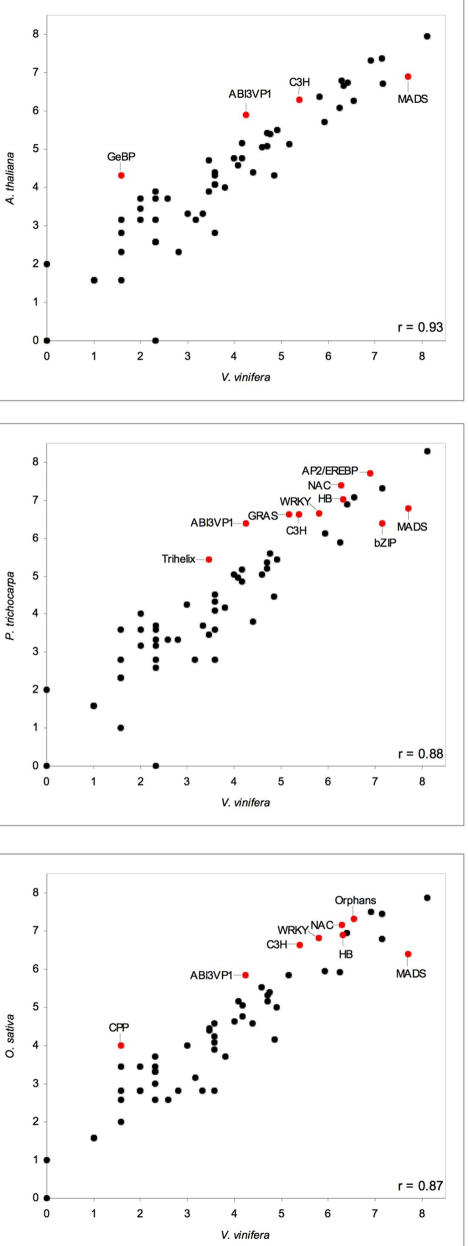
Scatter plot of the distribution of *V. vinifera* transcription factors. For each of the 60 families (1983 genes) of *V. vinifera* TFs (X-axis) (log base 2 transformed), family members have been plotted against the corresponding number reported for three other genomes: A) *A. thaliana* (http://arabtfdb.bio.uni-potsdam.de/v1.1), B) *P. trichocarpa* (http://poplartfdb.bio.uni-potsdam.de/v2.0) and C) *O. sativa* (http://ricetfdb.bio.uni-potsdam.de/v2.i). The degree of the correlation among TF gene numbers is indicated by the Pearson correlation value (r). Each scatter plot shows the TF families which were statistically over- or under- represented in pair-wise comparisons (χ^2^ tests were applied to untransformed data; p = 0.05).

Sixty-two families of TF genes were found, a number similar to the 64 for Arabidopsis, 62 for rice and 63 for poplar [63]. TF families like MYB, AP2/EREBP, bHLH and MADS-box include a large number of members [11], [60]. We compared the number of genes in each of the 60 grape TF families in common to the other three plant genomes: finding a nearly linear correlation (Figure 6). Thus the organization and number of TFs seem to be highly conserved in plant genomes. TF distribution in the grape genome (Figure 5A) indicates that only LGs 7 and 18 have a higher than average TF content. Clusters of AP2/EREBP genes are repeated in tandem on LGs 2, 7, 10 and 16; CCAAT genes on LGs 6, 8, 10 and 13; MADS-box genes on LGs 5 and 13; Myb genes on LG 8 and 17 (Figure 5B).

Across the species mentioned, MYB (279) are the most abundant [11], [64]. They play a role in controlling the accumulation of secondary metabolites in the grape berry [65]–[67]. A gene from this TF family is also known to play a key role in the regulation of anthocyanins and flavonols during the non-climacteric ripening of strawberry [68]. Non-climacteric ripening (occurring in fruit such as strawberry and grapevine) is a process characterized by the absence in respiratory pick and ethylene bursts, two phenomena typical of the climacteric fruits ripening.

In the grape genome were also found 143 leucine-zipper genes. Together with EREBP TFs they contribute to the plant's defense response [69]. In tepary and common bean, a bZIP gene plays an important role in the response to water deficit and in the regulation of abscisic acid levels. [70]

In the grape genome, the MADS-box family is also over-represented. These TFs regulate flowering-related phenomena, as well as other metabolisms [71]. MADS-box TFs may have been important during plant evolution because they allow plant reproductive structures to adapt to variations in climatic conditions [72]. It was found that two tandem MADS-box genes (MADS-RIN and MADS-MC) regulate fruit ripening and inflorescence determinancy in the climacteric fruit tomato. Mutation at *rin* locus caused a failure in the normal ripening physiology [73].

A ripening mechanism common to both climacteric and non-climacteric species, such as grape, has been hypothesized [74]. In support of this, we identified two TF classes in grape, AP2/EREBP and EIL, which contribute to ethylene signalling during ripening of climacteric fruits, and also found ethylene receptors belonging to ETR/ERS families.

### Repetitive elements

Matching the sequences of assembled contigs with original reads made it possible to characterize each DNA segment by the number of matching reads (see Materials and methods). For the read coverage of 10.7X, a DNA segment was considered unique when represented by 15 or fewer matches. Moderately repeated sequences (2 to 8 copies per genome) were expected to have 16–100 matches. Sequences with more than 100 matches were considered highly repetitive. They were masked before gene prediction, thus excluding most of the coding parts of repetitive elements from the putative gene set.

Dispersed highly repetitive DNA sequences were identified by an iterative procedure, and the resulting collection of 90,483 repetitive segments were grouped into 136 types. Members of each type were translated and compared to each other and the similarity scores were used in a UPGMA-like clustering. The similarity tree consisted of eight clusters lacking a common root (Figure S4), each of which was assigned to the known classes of repetitive DNA sequences (Table S7).

Grape transposable elements (TEs), totalling 108.5 Mb, represent the most abundant set of repeats. The repeats were included in group I (retrotransposons: Copia, Gypsy, LINE) and group II (DNA transposons: Mutator, CACTA, hAT) according to Feschotte et al. [75]. The most abundant TEs were Gypsy/athila-like elements followed by Copia elements. DNA transposons were represented by 9,562 copies (7.1 Mb). The TEs seem to be more abundant in grape compared to poplar [12], Arabidopsis and rice [11]. Putatively autonomous TEs were identified by significant BLAST analysis against the Uniprot database. TEs without a significant BLAST hit were attributed to the non-autonomous group (Table S7). Out of 136 repeat types, 20 were classified as long tandem repeats with a unit size from 100 to 430 bp. They were grouped into ten major sub-classes.

Short tandem repeats (microsatellites) were also identified. Their thresholds, number of copies and total DNA length are reported in Table S8. Microsatellites cover 2.1 Mb, including the telomeric repeats (TTTAGGG). Out of 171 contigs with identified telomeric sequences, 42 had telomeric ends. In the linkage map, they represent potential markers for telomeres.

An alternative estimation of the length of identified repetitive DNA was performed using the number and total length of reads matching repeat sequences, identified above. This new estimate gave a value of 138.5 Mb, corresponding to 27.4% of the 504.6 Mb genome size.

### Non-coding RNAs

#### MicroRNAs

MicroRNAs (miRNAs) and trans-acting siRNAs (ta-siRNAs) have a significant role in plant development and stress response [76], [77]. The majority of the 1220 plant miRNAs listed in the miRBAse [78] are from Arabidopsis (184), rice (243) and poplar (215). A BLAST search of sequences similar to the Arabidopsis miRNAs genes was performed on the grape genome. Allowing for three or fewer mismatches, 143 miRNA genes representing 28 families ([78]; Table 3, Table S9) were identified.

**Table 3:** Distribution of miRNA encoding genes of *V. vinifera* on LGs and number of their putative target genes grouped in families.

miRNA family^1^	Distribution on LGs^2^	Putative gene target families^3^
miR156/157 (**11**)	1, 4, 8, 11, 12, 14, 17	TF, *SQUAMOSA-BINDING PROTEINS* (**15**)
miR159/319 (**10**)	1, 2, 6, 11, 17	TF, *TCP/MYB* (**10**)
miR160 (**6**)	6, 8, 10, 13, 16	TF, *AUXIN RESPONSE FACTOR* (**4**)
miR162 (**1**)	n.d.	*DICER-LIKE* (**1**)
miR164 (**4**)	7, 8, 14, 17	TF, *NAC* (**4**)
miR165/166 (**9**)	2, 5, 7, 12, 15, 16	TF, *HDZIP-III* (**9**)
miR167 (**5**)	1, 5, 7, 14	TF, *AUXIN RESPONSE FACTOR* (**5**)
miR168 (**1**)	14	*ARGONAUTE* (**1**)
miR169 (**17**)	1, 4, 8, 11, 14, 17	TF, *HAP2-like* (**17**)
miR170/171 (**12**)	2, 4, 9, 10, 11, 12, 14, 15, 17, 18	TF, *SCARECROW-LIKE* (**12**)
miR172 (**9**)	6, 8, 13	TF, *APETALA-like* (**9**)
miR390 (**2**)	6	*TAS3* (**2**)
miR393 (**2**)	6, 16	Auxin transporter (**5**)
miR394 (**5**)	18	F-box (**2**)
miR395 (**16**)	1, 11, 12	Sulfate transporter (**2**)
miR396 (**7**)	1, 11, 12, 19	RNA-dependent DNA polymerases (**47**)
miR397 (**2**)	10	Laccases (**2**)
miR398 (**3**)	1, 6	*COPPER SUPEROXIDE DISMUTASE* (**3**)
miR399 (**16**)	10, 15, 16	Ubiquitin conjugating enzyme E2 (**2**)
miR400 (**1**)	n.d.	-
miR403 (**12**)	5, 7, 10	*AGO* (**1**)
miR408 (**1**)	7	Laccases (**2**)
miR414 (**8**)	1, 7, 9	Unknown (**2**)
miR773 (**1**)	n.d.	Unknown (**2**)
miR782 (**1**)	n.d.	Unknown (**2**)
miR827 (**1**)	n.d.	Unknown (**6**)
miR828 (**1**)	n.d.	TF, *MYB* (**3**)
miR846 (**1**)	n.d.	RNA-dependent DNA polymerases (**9**)

Grape miRNAs identified by BLAST search using *A. thaliana* miRNAs as reference are assigned to the 19 LGs of grape. Genes predicted to be targeted by miRNA are reported. Sequences of the mature miRNAs and the miRNAs*, secondary structures of some predicted pre-miRNAs are presented in Table S9.

1 The prediction of grape miRNAs by BLAST search (<3 mismatches) was performed as described by Jones- Rhoades et al. [129]. The number of loci is indicated in brackets.

2 The position of some loci on LGs is non determined (n.d.). miR169, miR395 and miR399 loci cluster frequently.

3 Potential target genes with a pairing site (score <2.5) of the corresponding miRNA family according to the rules of Jones-Rhoades et al. [129]. The number of putative target genes is indicated between brackets. Abbreviations: TF, Transcription factor; *TAS*, *trans*-acting short interfering RNA transcript.

4 Number of miRNA loci and families in Arabidopsis, rice and poplar according to miRNA sequence database release 10.0 (miRBase, [78]).

Three types of miRNAs (miR827, miR828 and miR846) were not previously found outside Arabidopsis, and were considered “non-conserved” miRNAs [79], [80]. However, these genes are present in the grape genome, indicating that they were either lost in the lineage leading to *Populus* or are missing from its genome assembly. The miRNA passenger strands (miRNAs*) are highly conserved between grape and Arabidopsis [79]. Sequences predicted to produce ta-siRNAs [81] are conserved in several plant species, grape included.

Putative grape miRNAs and siRNAs target the same classes of genes as they do in Arabidopsis, rice and poplar: transcription factor genes, genes involved in stress response and nutrient uptake, genes for RNA silencing and the non coding RNA *TAS3* (Table 3). In grape, 56 RNA-dependent DNA polymerase genes are potentially targeted by miR396 and miR846, a phenomenon not reported in other plant species.

BLAST searches identified four Dicer-like proteins (Helicase, RNAse IIIa/b domains), nine *Argonautes* (PAZ/PIWI domains), and six RNA-dependent RNA polymerases (RdRp domains), indicating the presence in the grape genome of a complex RNA processing machinery (Figure S5).

#### Transfer RNA

The tRNAscan-SE program [82] identified 719 putative tRNA genes. 163 of them are pseudogenes, 3 are suppressors for the TAA codon while 553 correspond to 52 anticodons for all amino acids (Table S10).

#### Small nuclear RNA

Non-coding RNAs include five major and four minor snRNA families, all components of splicing factors. The Arabidopsis snRNA list of Wang and Brendel [83] was used to search for similar sequences in grape. We found 89 snRNA genes and pseudogenes (75 in Arabidopsis) (Table S11). Several snRNA genes were clustered in the genome.

#### Ribosomal RNA

Large rRNA units consist of two segments, one hosting the genes 18S rRNA, 5.8S and 28S, the second containing three arrays of tandem repeats. In grape the length of the rRNA unit is around 10.8 Kb. The variable segment includes three arrays of tandem repeats: about 40 copies of a 44–45 bp repeat, three copies of a 150 bp repeat and 5.5 copies of a 193 bp repeat. The unit is repeated 1450–1550 times in the genome (16.1 Mb). rRNA units may contain insertions of retrotransposons of three different lengths (2870, 2950, and 5800 bp). Retroelements in rRNA sequences may cause transposition of rRNA sequences.

The DNA sequence for the small ribosomal RNA unit (1,250 bp) contains two genes for 5S rRNA, 120 bp each with a single nucleotide difference between them. In the genome the unit was represented by 170–180 copies. Together, large rRNA and 5S rRNA sequences were estimated to amount to 16.3 Mb.

#### Small nucleolar RNA

Based on the Arabidopsis snoRNA genes [84], 166 sequences representing 79 families were found in grape (Table S12). Most of the grape snoRNA genes (110) are clustered; 62 snoRNA genes were located inside 34 genes encoding six ribosomal proteins and one eIF-4F factor.

### Pinot Noir genome structure and evolution

The existence of structural diversity between homologous chromosomes within plant species has been reported [85]. This type of molecular variation seems to be common in allogamous plants [86] and could also be a characteristic of autogamous species [33]. Grape does not tolerate long term inbreeding [5] and high outcrossing rates maintain the genome in a heterozygous state, as evident in the remarkable variation found in collections of grape varieties [87].

The genome sequence data from a cultivated grape variety provides unprecedented insight into the structural nature of heterozygosity in an outcrossing species. The variation within this clone of grape consists largely of chromosome-specific gaps and hemizygous DNA. In addition to the regions in which it was possible to merge haplotypes representing DNA from both chromosomes in a consensus sequence, regions were found which were chromosome-specific, i.e., either with different DNA sequence flanked by orthologous regions of the two homologous chromosomes (hemizygous DNA) or gaps corresponding to sequences absent in one chromosome but not in the other. One million gaps, covering 48.9 Mb, and 65.1 Mp corresponding to hemizygous DNA distributed in 22,610 contigs were identified. These data allow us to conclude that the homologous chromosomes of Pinot Noir differ on average by 11.2 % of their DNA sequences and that the grape genome exists in a dynamic state, mediated at least in part by transposable element activity, as reported for helitron TE [88]. Indeed, the large grape genomic gaps are frequently bordered by 5 bp direct repeats, reminiscent of a type of DNA excision mediated by a precise process of transposition [89].

**Figure 7:**
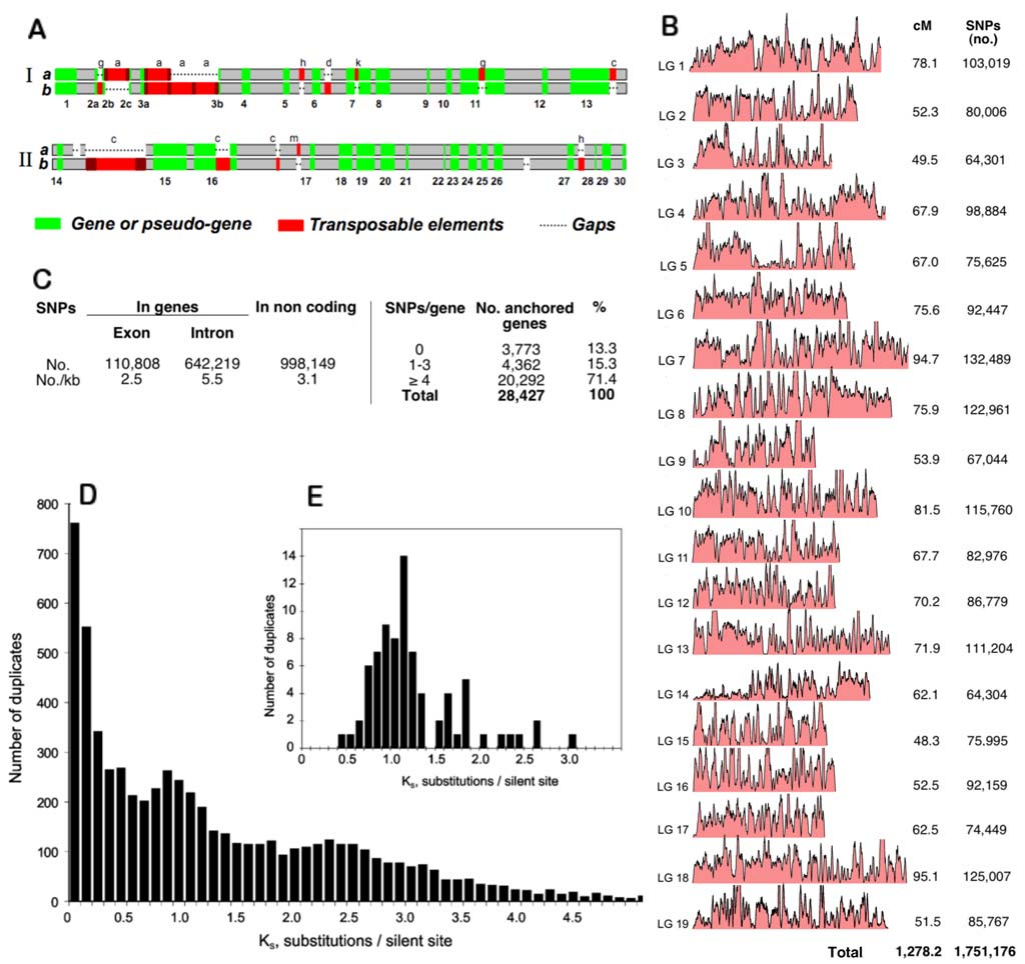
Features of the Pinot Noir heterozygous genome. A) Comparison of constrasting haplotypes (*a* and *b*) co-mapping at two almost contigous regions in metacontig 32,921 of chromosome 1. Above: the 188 kb region; below: the 215 kb region. I from contig groups 1030-H15, 1079-G03, 2068-K04, 1034-C17 and II 2010-J07, 2044-L11, 1030-N10. In the genetic map the two regions are positioned at 60.1 cM: see preliminary experiment in Text S1. TE elements are labeled as follows: c: Copia; g: Gypsy/gypsy; a: Gypsy/athila; d: hAT/Dart; k: Karma; h: hAT; m: Mutator. B) SNP profiles of the 19 LGs of *V. vinifera*. Left and right of the figure correspond respectively to top and bottom of LGs of Troggio et al. [15]. The SNP values reported do not consider gaps in and among metacontigs. C) SNPs in exons and non-coding DNA and percentage of anchored genes tagged with SNPs. In parts B to E of this figure, gene prediction and annotation and the exon-intron boundaries were based on the methods described in Solovyev et al. [114]; Korf et al. [101]; Majoros et al. [115]; Altschul et al. [116]; Huang and Madan [117]. D) Relative age of grape duplicated genes estimated from the number of synonymous substitutions per synonymous sites (K_S_ values). The peak between 0.6 and 1.2 KS supports a relatively large scale duplication event. Paralog genes were identified as in Li et al. [120] and K_S_ distributions were calculated as in Maere et al. [105]. E) The same as in D for genes present in duplicated chromosome segments.

The genomic region represented in Figure 7A highlights the differences which exist between homologous haplotypes. Notable differences in this region concern the presence of gaps and the number of copies of TE.

In the preliminary experiment (see Text S1), it was found that the frequency of SNPs correlated with deletions and insertions. Segments with less than one in/del per Kb had 4.4 SNPs per Kb, whereas segments with one or more in/del per Kb had 16.7 SNPs per Kb. A total of 2 millions SNPs (1,751,176 anchored and the remaining present in other assembled sequences) were discovered and validated and more than a million in/dels were annotated on the sequence with defined location. Our data allow us to extend the evaluation of nucleotide variation to the entire genome rather than to limited resequenced DNA regions [86]. Among recently sequenced animal genomes, a high SNP frequency was found in sea urchin [90] and *Cyona intestinalis* [91]. Across the grape genetic map (Figure 7B), the SNP frequency had an average value of 4.0 per Kb.

Coding and non-coding regions demonstrated different degrees of polymorphism with 2.5 and 5.5 SNPs per Kb respectively. One or more SNPs were found in 86.7% of anchored genes and 71.4% of genes had more than four SNPs (Figure 7C). Those gene-based markers are valuable tools, as SNPs present in functional genes may cause natural phenotypic variation [92], [93] and help in genetic diagnosis. In addition, we noticed some reduction of SNP frequency in gene desert regions, described for the dog genome [94].

In several regions of the 19 LGs, SNP frequency peaks between 5 and 7.5 per 1 Kb, even if the frequency may reach values much higher than those cited (Figure 7B). Other regions displayed dramatically reduced frequencies. Therefore, as shown for human [95], dog [94] and *Anopheles* [96] genomes, the Pinot Noir chromosomes consist of large blocks where two haplotypes are present. The sparseness of putative quasi-homozygous haplotypic blocks indicates that heterozygosity prevails.

Arabidopsis and poplar have likely undergone three rounds of whole genome duplications during evolution [12], [97], [98], although this has been challenged recently [7]. The first duplication (referred to as 1R, [98], [99]) may have predated the divergence of monocots and eudicots, while the second one (2R) probably occurred around the radiation of the core-eudicots prior to the divergence of poplar and Arabidopsis [12], [99]. The most recent duplications in poplar and Arabidopsis have occurred after their divergence [94]. The current thinking is that *Vitis* is an early diverging lineage within the rosids that has diverged prior to the divergence of Arabidopsis and poplar [100]. We determined the relative age of grape duplicated genes from the number of synonymous substitutions per synonymous site (K_S_). The age distribution of *Vitis* duplicates shows a clear peak of K_S_ values between 0.6 and 1.2 suggesting a relatively recent large-scale duplication event (Figure 7D). A smaller peak is also visible for K_S_ values between 2.0 and 2.5, probably corresponding to more ancient large-scale duplications, as is the case for poplar and Arabidopsis [101].

Different approaches were taken to estimate the age of the youngest large-scale duplication event. First, it should be noted that the youngest peak lies to the left of the peak formed by K_S_ values between orthologs of *Vitis* and Arabidopsis (Figure S6) although one should be very cautious in comparing different K_S_ distributions due to different substitution rates in different organisms. Second, we also detected duplicated segments, covering about half of the genome, using a previously described method [102]. K_S_ values of genes in these duplicated blocks (Figure 7E) showed that the majority of these are responsible for the 0.6–1.2 K_S_ peak (Figure 7D) and thus likely to be remnants of a single large-scale duplication event. We have also used phylogenetic approaches (see Methods) to estimate the relative age of genes in duplicated blocks. In total, 485 gene pairs support duplication prior to the split Arabidopsis–*Vitis*, while 523 gene pairs support duplication after the divergence of Arabidopsis and *Vitis*, i.e., are *Vitis* specific, although distributions of K_S_ values for these two sets of genes are not discernable (not shown). When duplicated blocks of which at least two-thirds of the anchors support the same tree topology are considered, almost twice as many blocks support duplication within the *Vitis* lineage than before the divergence of *Vitis*. As a matter of fact, we suspect the actual number of genes supporting a *Vitis*-specific duplication to be higher. Indeed, it has been shown in several studies that, following gene duplication, one of the duplicates evolves at an increased rate [103], [104]. This could easily lead to the inference of erroneous tree topologies where one of the *Vitis* duplicates branches off earlier than it should, in particular if the duplication event occurred shortly after the specialtion of *Vitis* (see further).

Jaillon et al. [7] propose that three ancestral genomes contributed to the *Vitis* lineage and suggest ancestral hexaploidization for most eudicots, while not finding evidence for a recent duplication in grape. Furthermore, they suggest that, since their split, poplar has undergone an additional whole genome duplication, while Arabidopsis has undergone two additional genome duplications. These results are at odds with our findings. Reanalysis of Arabidopsis and poplar genomes (not shown) uncovers, for both, many homologous segments with a multiplication level between five and eight, which suggests three rounds of duplications for both genomes [97]. If the Arabidopsis and poplar genomes were ancient hexaploids, to which two additional genome duplications had been added, fragment multiplication of up to twelve should be expected for Arabidopsis, and up to six in poplar.

**Figure 8:**
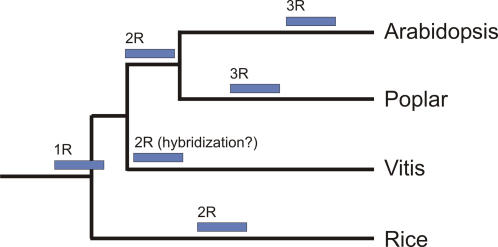
Scenario of angiosperm genome evolution. Alternative scenario to the one proposed by Jaillon et al. [7] to explain angiosperm genome evolution. Our analyses seem to suggest that there has been a large-scale duplication event, likely a hybridization event, in the *Vitis* lineage, rather than before the split of *Vitis* and other dicots. See text for details.

The fact that there is substantial ambiguity in the dating of the duplicates in duplicated segments suggests that the most recent large-scale duplication event reported here for *Vitis* might have occurred in close proximity to the *Vitis* speciation event. Therefore, an alternative scenario than the one presented by Jaillon et al. [7] that we would like to put forward is shown in Figure 8. We assume three genome duplications to have occurred in both poplar and Arabidopsis, as proposed earlier [12], [98], [105], one of which has been shared by all dicots (and possible also by the monocots, see [98]), one that has been shared by Arabidopsis and poplar, but not *Vitis*, and one that has been specific to Arabidopsis [98], [105] and poplar [12], respectively. Since many regions of the *Vitis* genome appear in triplicate in both Jaillon et al. [7] and our own analyses (not shown), the genome duplication shared by all dicots might have been followed by a hybridization event in *Vitis*, shortly after its divergence from the lineage leading to poplar and Arabidopsis (see Figure 8).

### Concluding remarks

The Grapevine Genome Initiative was established with the aim of accelerating the breeding of a difficult perennial species. Grape breeding for disease resistance, if not for immunity, would be a solution to the problem of the emergence of aggressive races of micro-organisms that are currently controlled by massive use of agrochemicals. The problem is not a simple one: how to modify a complex and highly heterozygous genome without altering wine quality. Precise knowledge of all the genes influencing quality and resistance traits is an absolute prerequisite for such modifications.

A high number of genes related to disease–resistance have been identified; many of them have been mapped to LGs and a large part of them are tagged with one or more SNPs. These resistance genes, however, did not co-evolve in the presence of the most important grape pathogens [34], a condition which may have not sufficiently protected the species. This is in part the reason why a deep knowledge of the grape genome is the starting point for developing genetic strategies to counter pathogens.

Description of the grape genome sequence opens the opportunity for molecular breeding in grape. The fertility of hybrids between wild and domesticated grape species with 19 seemingly co-linear chromosomes [5], [106]–[108] makes it feasible to introduce new resistance genes via traditional breeding. The NBS gene clusters identified here can be associated with QTLs affecting disease resistance or tolerance behaviour of grape varieties (this is the case with LGs 12, 14, 15 and 18; [27], [39]). This large and underexploited reservoir of resistance genes could be easily moved in clusters across genomes by choosing appropriate molecular markers to selectively introgress only the resistance traits. This would prevent the loss of alleles important for grape and wine quality. Thus, the anchored sequence of the grape genome, together with the large arsenal of SNP loci, now offers a tool to open a new era in the molecular breeding of grape.

WGS using longer read dye-terminator sequences can be combined with shorter SBS sequence data using dedicated assembly programs. Using this method we have resolved a complex heterozygous eukaryotic genome. Future whole genome sequencing efforts should be able to combine these two methods to produce assemblies in shorter times while reducing the need for resources. The ability to resolve the haplotypes in Pinot Noir suggests that sequencing DNA mixtures, for example more than one genotype of a given crop, is practical. Such an approach generates both a consensus sequence of the genome and a set of mapped marker loci to be used in breeding programs.

## Materials and Methods

### DNA source

In order to prepare shotgun libraries, DNA was extracted from young shoots of Pinot Noir, clone ENTAV115, randomly sheared and size-selected. Two BAC libraries were also constructed ([109]; Keygene, Wageningen, NL) and clones assembled in a physical map (http://genomics.research.iasma.it). A population of 94 F_1_ plants from the cross between Syrah and Pinot Noir was the source of the DNA used for mapping markers and anchoring metacontigs.

### Libraries

Fosmid and shotgun libraries were from DNA purified by a CTAB method [110]. Sheared DNA (Gene Machines Hydroshear, Ann Arbor, MI) was size selected to produce libraries with insert sizes of 2, 3, 6, 10 and 12 Kb. DNA was ligated to a high copy plasmid vector and transformed into DH10B T1r *E.coli* cells (Invitrogen, Carlsbad, CA). The fosmid library was produced from DNA fragments between 30 and 45 Kb. DNA inserts were ligated into a pCC1FOS vector packaged with MaxPlax lambda extracts and transfected into EPI300-T1r *E.coli* cells (Epicentre, Madison, WI). LB agar contained chloramphenicol and 99,840 clones were picked (QPix2 Genetix, Hampshire, UK) into 384 well plates containing LB freezing medium, incubated for 18 h, replicated and stored at −80°C.

### Sanger shotgun sequencing

DNA was amplified from bacterial cultures by a rolling circle technology (Templiphi kit; GE Healthcare, Amersham) and Sanger sequenced on MegaBACE 4500. Clones with inserts from 6 to 20 Kb, BAC clones and clones from fosmid libraries were amplified by the Templiphi large kit. BAC clones were bidirectional dye terminator sequenced on ABI Prism® 3730.

### Sequencing by synthesis (SBS)

Pinot Noir DNA isolated as described was subjected to nebulization to generate fragments of approximately 620 bp. These were amplified as in Margulies et al. [14] and sequenced on the Genome Sequencer 20 (Roche Applied Sciences, Indianapolis, IN). The standard protocols for 454 Sequencing using the Genome Sequencer 20 system call for the generation of a library of tagged single stranded DNA molecules (see Margulies et al [14] for details). This single stranded library is then tested for optimal sequencing parameter through generation of sequencing beads by emulsion PCR with dilutions of the single stranded library. This titration step determined that three microlites of a single stranded library were used to generate 23 million beads. The standard GS20 pyrosequencing profile uses a sequencial flow of each nucleotide in a repeating pattern of TACG. This pattern is repeated for 42 cycles as per the standard protocol and generates 100bp of sequence information on average. For the purposes of generating longer sequencing reads the sequence profile of 42 cycles of nucleotide flows was changed to 100 cycles which increased the average read length from 105 bp to 200 bp. The GS20 has standard software to recognize high quality reads and convert the signal (light) into a base call. The standard software GS20 package was used to generate the sequence files. In total, 12.5 million reads corresponding to 2,111 million Q20 bases were produced.

### Primer walking

Clones bridging neighboring contigs were selected for gap closure. The clones were grown in 384-well plates and sequence-specific primers were designed and used in dye terminator sequencing reactions resolved on MegaBACE 4500.

### Genome assembly

6.2 million reads for a total of 3.5 billion Q20 bases were produced by Sanger sequencing from 43 libraries (Table S1) and about 90.6% of reads were paired. Chloroplast sequences were detected and the chloroplast genome was assembled for assessing the sequence quality and insert size distribution of each library, characteristics that were used in assembly. Chloroplast forward and reverse reads validated the correctness of data tracking and the contamination level for each sequencing plate. The size of the chloroplast genome was 160,928 bp. Remarkably, the sequence was identical (without a single mismatch) to the one already published [100].

SBS data were essential to identify polymorphic sites and close small gaps. The amount of chloroplast and mitochondrial sequences in SBS data was 5.5 and 2.0%, respectively, *vs* 3.1 and 1.8% in Sanger sequences. Four programs developed at Myriad Genetics Inc. were organized into a pipeline for WGS assembly: (1) Sanger and SBS sequences were compared by the Match program. It produced a table of pairwise sequence overlaps with indication of the sequence orientation, offset and match score. The overlaps were accepted if they involved more than 50 bp with no more than 2% of polymorphic positions. (2) Consensus sequences were built using the Assemble program, adapted to specified levels of heterozygosity (2% or less) and large gaps (up to 500 bp). The program reads the sequence and quality data in Fasta or GDE format, considers clone sizes and performs multiple alignments, building the consensus sequence and reporting polymorphisms of the sequence. (3) Sequences were aligned with the Align program in a two-step procedure including fast search of identical segments and optimal alignment of gaps up to 7 Kb. Larger or multiple gaps may still be a problem for the alignment and leave some overlapping contigs not merged. (4) Visual comparison of two sequences was performed by the Dotmap program. The result of the assembly is a Fasta file of assembled contig sequences with quality values assigned for each position and the list of positions of polymorphisms. (5) Metacontigs were constructed as ordered and oriented groups of contigs linked with paired reads matching to non-repetitive parts of the contigs. We used also marker information to avoid building chimeric metacontigs from different LGs (see Text S1 for more details).

### Genetic maps and genome integration

Metacontigs were integrated in the 19 grape LGs based on the genetic map derived from the cross Syrah X Pinot Noir. To improve marker density, polymorphic sites identified during WGS were selected for developing 799 additional SNP-based markers (http://genomics.research.iasma.it) using the SNPlex™ Genotyping System [111]. DNA was prepared according to the instructions and the samples were analyzed on the ABI PRISM® 3730xl (Applied Biosystems, Foster City, CA). Data were analyzed by Gene Mapper v. 4.0 (Applied Biosystems, Foster City, CA). The genetic maps were followed a double pseudo-testcross strategy [112]. Marker phase was determined by the Phasing algorithm (http://math.berkeley.edu/dustin/tmap/; [113]), which provides LG assignment and ordering of loci. LG were assembled with a minimum LOD of 8.0 and a maximum distance of 35 cM. Homologous LGs of the two parents were merged in a consensus map.

### Genes and gene families

Methods used were FgenesH [114], homology-based FgenesH+ [114], Twinscan [101], GlimmerHMM [115] and Tentative Consensus [94] transcripts derived from 320,000 ESTs deposited in databases. Trimmed sequences were clustered using MegaBLAST [116] and aligned using Cap3 [117]. After quality testing 28,856 TCs were retained.

BLAST searches against Uniprot and plant protein databases, annotated with GO terms, of various domain libraries were the base for gene annotations GO terms were extracted from BLAST searches against KEGG databases, KOBAS of metabolic pathways and InterproScan [118] and clustered using their semantic similarity [119], accuracy weight and the path from the root node of the ontology to the most detailed annotation. More than 79% of the gene models were annotated.

Functional classification was based on Gene Ontology (www.geneontology.org) and manually controlled.

Homologs across species were established using a BLAST search against Rice, Poplar and Arabidopsis, considering sequence alignment coverage, best multi directional BLAST hits, sequence identity and protein domains. Sets of clusters reflected different levels of similarity among species as well as unique and putative species-specific genes. For the analysis of specific gene families, methodological variations were introduced as reported in text.

### Genome duplication

Genes with similarities to TEs were removed and paralogs identified as in Li et al. [120]. Age distributions were build as described by Maere et al [105]. Duplicated segments were analyzed with i-ADHoRe [102], based on the following parameters: gap size of 40 genes, Q value of 0.9, probability cut off of 0.001, and a minimum of 3 homologs to define a duplicated segment.

Phylogenetic trees for duplicated genes (so-called anchors) in duplicated segments were based on pairs of grape paralogs representing the reciprocal best hits with aligned length of >150 amino acids and considering comparisons with proteins from *Physcomitrella patens*, used as outgroup, and the best Arabidopsis homolog. Proteins were aligned with CLUSTALW and only unambiguously aligned regions were considered. Tree construction used seqbot, protdist, neighbour and consense from the PHYLIP package [121] with 1000 replicates. Only topologies with over 70% bootstrap support were considered. For each paralog, if the topology was (Grape1, Grape2) Arabidopsis, it was concluded that the paralog was duplicated after the split of grape and Arabidopsis.

### Repetitive elements

Based on 10.7X coverage, a DNA segment was defined unique when associated to 15 or less matches. The threshold was selected as the middle point between two Poisson distributions, with 10X and 20X the expected coverages corresponding to unique and duplicated segments, respectively. For dispersed repetitive sequences, an iterative procedure was developed. Each segment was searched against all sequences, starting with the repeat presenting the highest number of matches. At each iteration, the program identified repeats with decreasing similarity to the original seed repeat, and the complete set of copies of a particular repeat cluster was obtained. These DNA segments were masked and the remaining sequences were searched for the next repeat with the highest number of matches. Members of each of the identified repeat types were translated and compared using BLAST program. The similarity scores were used in a UPGMA-like clusterization. Short tandem repeat (microsatellite) motifs were identified by a specifically designed program considering their number above a threshold. This was selected based on the occurrence of the motif in the genome so that the number of segments with units exceeding the threshold would be less than 1.

### Non-coding RNAs

Methods used for miRNA detection and individuation are cited in the caption of Figure S5. Methods and reference papers for tRNA, snRNA and snoRNA are cited in the text. Ribosomal RNA were defined and computed according to assembly program of Myriad Genetics Inc. (Salt Lake City, Utah).

### Transcription factors

The reference information was from PlnTFDB, an integrate plant transcription factor database [63] including genes from *A. thaliana* (ArabTFDB), *P. trichocarpa* (PoplarTFDB) and *O. sativa* (Rice TFDB) (available at http://plntfdb.bio.uni-potsdam.de). For each TF family, conserved domains were used as queries for searching similar sequences in the grape genome. The protein domains of identified TF were classified using the Pfam database [122].

## Supporting Information

Text S1. Supporting text; supporting references
(0.04 MB DOC)

Figure S1. Histograms of contig size distribution. Histograms showing the distribution of the assembled contigs in size classes. The average contig size is 9.1 Kb. Half of the genome is covered by 7,878 contigs larger than 18.2 Kb.
(0.08 MB TIF)

Figure S2. Anchored and oriented metacontigs along the 19 LGs. Representation of the 435.1 Mb of V. vinifera genomic sequence contained in 397 metacontigs aligned and oriented to the genetic map of the 19 LGs. Distances (shown in brackets on the left for some markers) refer to Troggio et al.'s dense map [1] (http://genomics.research.iasma.it). Most metacontigs were anchored to the map using markers with unique sequence locations: SSRs, BAC-end sequences or SNP-based markers derived from either ESTs or assembled sequences of the two haplotypes of the Pinot Noir genome. Metacontigs with no marker information were associated to other metacontigs anchored to the map. There are reliable links between them but they are not merged for several reasons, i.e., too large an overlap between them due to some contigs at the end of one metacontig not being in the proper place; gaps too large due to missing contigs; poor quality or insufficient number of links. Approximate size in Kb of each metacontig is indicated on the right. Gaps separating metacontigs are of undefined size.
(2.27 MB TIF)

Figure S3. Grape gene class assignment based on putative function. Functional classification of putative grape genes (total and grape-specific) based on Gene Ontology (www.geneontology.org).
(4.92 MB TIF)

Figure S4. Repetitive element classification and clustering. Phenograms showing the relative similarities of 95 types of repetitive elements out of the 136 identified in the assembled V. vinifera genome. The remaining 41 repeat types (without ORFs or with ORFs shorter than 200 bp) are not included. Repeat types were classified according to Feschotte et al. [2]. Clustering was performed by an all vs all comparison using the BLAST program and was visualized by DrawTree (Myriad Genetics, Salt Lake City, Utah).
(0.65 MB TIF)

Figure S5. Major RNA silencing proteins present in V. vinifera. Major proteins participating in the RNA silencing pathways in V. vinifera have been identified by homology to Arabidopsis proteins using tBLASTN against the V. vinifera genome. Coding sequences of predicted genes were verified in the TC trancripts database. Protein alignments and trees were performed using MEGA version 4 [3]. Aligned protein sequences are: A) Dicer-like proteins (DCLs). Aligned protein sequences are Vitis vinifera putative Dicer-like proteins and A. thaliana DCL1 (At1g01040/Q9SP32), DCL2 (At3g03300/NP_566199), DCL3 (At3g43920/NP_189978) and DCL4 (At5g20320/AAZ80387). B) AGO proteins. Aligned protein sequences are V. vinifera putative Argonaute proteins and A. thaliana AGO1 (At1g48410/NP_849784), AGO2 (At1g31280/NP_174413), AGO3 (At1g31290/NP_174414), AGO4 (At2g27040/NP_565633), AGO5 (At2g27880/Q9SJK3), AGO6 (At2g32940/NP_180853), AGO7 (At1g69440/AAQ92355), AGO8 (At5g21030/NP_197602), AGO9 (At5g21150/CAD66636), and AGO10 (At5g43810/Q9XGW1). C) RNA-dependent-RNA-polymerases (RDRs). Aligned proteins are V. vinifera putative RDRs and A. thaliana RDR1 (AT1g14790/NP_172932), RDR2 (AT4g11130/NP_192851), RDR3 (AT2g19910/NP_179581), RDR4 (AT2g19920/NP_179582), RDR5 (AT2g19930/ NP_179583), and RDR6 (AT3g49500/NP_190519).
(0.11 MB TIF)

Figure S6. Duplicated state of the grape genome. Age distributions of Vitis paralogs (pink line) and Vitis-Arabidopsis orthologs (blue bins).
(0.40 MB TIF)

Table S1. Details of libraries used in sequencing and estimation of the V. vinifera genome coverage.
(0.03 MB DOC)

Table S2. Summary of the whole genome shotgun assembly of V. vinifera.
(0.04 MB DOC)

Table S3. Resistance-related genes of V. vinifera. (0.10 MB DOC)

Table S4. Gene family members involved in the core phenylpropanoid pathway, flavonoid and stilbene branches in V. vinifera.
(0.10 MB DOC)

Table S5. Putative genes encoding enzymes participating in the terpenoid pathway of V. vinifera.
(0.15 MB DOC)

Table S6. Transcription factors of V. vinifera.
(2.20 MB DOC)

Table S7. Repetitive elements in the assembled V. vinifera genome.
(0.04 MB DOC)

Table S8. Microsatellites identified in the assembled V. vinifera genome.
(0.03 MB DOC)

Table S9. Current state of IASMA database dedicated to V. vinifera mature miRNAs and miRNAs*, including the predicted fold-back structures of the pre-miRNAs.
(0.09 MB DOC)

Table S10. Number of identified tRNAs containing specified anticodons compared with the corresponding numbers of Arabidopsis.
(0.07 MB DOC)

Table S11. Number of genes identified in the V. vinifera genome for each of the nine families of snRNAs compared to Arabidopsis.
(0.04 MB DOC)

Table S12. Number of genes included in the different snoRNA families identified in the V. vinifera genome by searching against Arabidopsis snoRNAs [10].
(0.10 MB DOC)
