# Supplementary material for: A High Quality Draft Consensus Sequence of the Genome of a Heterozygous Grapevine Variety
Source: PLoS One. 2007 Dec 19;2(12):e1326. doi: 10.1371/journal.pone.0001326 (PMC2147077; doi:10.1371/journal.pone.0001326)
Supplement: Text S1. — Supporting text; supporting references (0.04 MB DOC) [file pone.0001326.s001.doc]

**Supporting Text**

***S1. Preliminary experiment.*** Heterozygosity and repeat content of the grape genome was estimated prior to WGS sequencing. Seven overlapping BACs from a 0.5 Mb region of chromosome 1q (positioned at 60.1 cM on the genetic map) were sequenced (10X coverage). Metacontigs were assembled using 10 Kb clones from a 7-BAC pooled library. Gaps between the adjacent contigs were closed using an automated primer walking procedure. The assembly of 403 Kb was represented by two non-overlapping contigs of 188 Kb and 215 Kb with a 4.5 Kb gap between them. Two alignments were obtained: haplotype **a** represented by three BACs (1030-H15, 2044-L11, and 2010-J07) and haplotype **b** represented by four (1079-G03, 2068-K04, 1034-C17, and 1030-N10). The overlap of 123 kb between the haplotypes contained 1258 SNPs, 276 gaps shorter than 100 bp and 6 large gaps (from 100 bp to more than 10 kb). The average estimate was of 1 SNP per 100 bp, 1 small gap per 450 bp and 1 large gap per 20.5 kb, which could be extrapolated to about 5.0 million SNPs, 1.1 million small gaps, and 24,000 large gaps per genome. Almost every large gap had a flanking pentanucleotide duplication of the target sequence which may be helpful in correct identification of the gap location.

***S2. WGS sequencing*.** Creation of the consensus sequence of a highly heterozygous genome is a considerable computational challenge. In the grape genome, the level of heterozygosity would be sufficient (10-15 SNPs per pair of reads from a single clone if both reads of the clone are successful and are of good quality) to assemble individual haplotypes if coverage of each haplotype were sufficient. However, selected clones with a high quality of Sanger reads (700 or more Q20 bases) provided only around 2.6X coverage of paired reads per haplotype. This would have generated around 384,000 contigs with an average length 2.5 Kb, compared to the expected 38,600 contigs with an average length 12.9 kb for a 5X coverage of a homozygous genome.

As a consequence, assembly of a heterozygous genome requires that the similarity between sequences be estimated in order to allow merging when the number of mismatches does not exceed 1-2% (based on the degree of heterozygosity estimated from the preliminary sequence) and when it is supported by a sufficient number of paired reads.

For conservative repeats and in order to avoid erroneous assembly, merging repetitive sequences was allowed only when the supporting pairs of reads were non-repetitive with the sizes of the clones large enough to cover the repeat segment. For each clone with non-repetitive ends, a chain of overlapping reads was created that covered the gap between the forward and reverse ends of the clone. Using this procedure it was possible to assemble all unique regions of the genome and put them in their correct order, but it did not provide identification of the conservative repeats filling the gaps between the unique segments.

Assembly across long (10-15 kb) repeats required large (40 kb) fosmid clones with both ends in unique segments. The 5.2X coverage of fosmid clones provided on average 1.7 clones per 10 kb repeat or 1.4 clones per 15 kb repeat. At this rate, about 20-25% of long repeats were expected to be without covering fosmid clones.

Short tandem repeats with a unit size smaller than the range of variability of the clone sizes represented an additional problem. As the number of tandem copies exceeded several dozen, chances of finding incorrect matches between unrelated sequences approached 100%. Thus, we completely removed contigs consisting of tandem repeats from further assembly.

The assembly started by comparing all Sanger reads with each other. Comparison was based on the Match program (Myriad Genetics Inc., Salt Lake City, Utah), designed for parallel matching of large amounts of sequence data. This resulted in a table of pairwise sequence overlaps with an indication of the sequence orientation, offset and match score. The expected number of read overlaps in the unique part of the genome was almost twice the sequence coverage of the genome, i.e., 6 x 2 = 12 overlaps per read. Reads with 12 or less overlaps were selected for assembling initial unique contigs. Such a selection involved about 58% of reads from unique regions and only 0.5% from duplicated segments.

Building the consensus sequence was performed by the Assemble program (Myriad Genetics Inc., Salt Lake City, Utah), modified for sequences with a specified level of heterozygosity and large gaps. This program takes into account the sequence and quality data in Fasta or GDE format together with information on the expected clone sizes and performs multiple alignments of the sequences while building the consensus sequence and analyzing the polymorphism of contrasting haplotypes. Short repeats (1 kb or less) were easily accomodated by this procedure. A large fraction of the contig ends resided in repetitive or non-covered regions. The next step was to find neighboring contigs linked by pairs of reads from the same clones where each read was located in a unique sequence of the contigs. At this stage, contigs with more than 2% of polymorphic sites were merged if the coverage of each contig was half the normal value (which means that each contig represents an alternative haplotype at one DNA region).

A problem with the criteria used in this approach, i.e. expected SNP frequences and sequence coverage, was that there remained the possibility of misassembly in repetitive or recently duplicated regions. Errors were identified by the presence of clone links between contigs that were not supported by the sequence overlap. When the position of missassembly in a contig was identified the contig was split and the reads were reassigned. Both merging and splitting contigs were easily achieved due to having complete information on the alignment of all original reads in an extended GDE structure which contained the quality values for each aligned read in addition to the sequences.

When it was possible to align alternative haplotype sequences, they were represented by a single consensus sequence. In SNP positions, the alternative bases were represented by a degenerate base in IUPAC notation. Sequences inserted only in one haplotype were included in the consensus sequence. There were cases, however, when a segment was inverted between the haplotypes. In such cases both sequences were retained in their original form.

***S3. Assembly results.*** Given a 6X sequence coverage, a read was considered potentially repetitive if it overlapped more than 12 other reads. All selected non-repetitive reads were assembled into 211,374 initial “seed” contigs. By using long clone links with non-repetitive clone ends, the seed contigs were ordered into metacontigs (supercontigs, scaffolds) where adjacent contigs were tested for overlaps. With each iteration the stringency for identifying sequence overlaps was decreased allowing a higher level of heterozygosity in the overlap. When the assembly reached 120,000 contigs, the data were enhanced with SBS sequencing. The threshold for the gap size in the alignment of neighboring contigs was also increased with each iteration to account for the large gaps present in contrasting haplotypes. Gaps larger than 1 kb (up to 15 kb), however, were still were problematic. In such cases, the assembly procedure generated several alternative contigs representing various combinations of sequences from two haplotypes with missing repetitive reads in the gap segment.

Despite this stringent approach to avoiding incorrect assembly over repetitive segments, a small fraction of erroneous overlaps and clone links produced incorrect contigs and metacontigs. It was possible to resolve some of these ambiguities by adopting a rule of majority: that is, either the variants with the largest number of compatible overlaps and clone links were selected, or contigs and metacontigs were mapped to chromosomal maps and cases where metacontigs mapped to alternative LGs were resolved.

After removal of 10,847 completely repetitive contigs, mostly from centromeric regions and rRNA gene clusters, and discarding 7,003 contigs shorter than 1,000 bp, the iterative assembly produced 58,611 contigs corresponding to 530.9 Mb of genomic DNA. 44,179 of the 58,611 contigs were assembled into 2,093 metacontigs covering 477.1 Mb.

***S4. LG anchored-DNA and LG/chromosome representations.*** Metacontigs with a large size were easily anchored to specific linkage groups. Metacontigs anchored by a single bridge were also included in the anchored genome (127 contigs covering 40.2 Mb with an average size of 316 Kb). The average gene content of 316 Kb corresponds to about 22 genes. Half of these contigs have the chance to be correctly anchored. For the other half, their genes may have a reversed orientation. Given the reduced average gene content of these genomic regions, our macro representation of LGs in terms of gene content and SNP and GC distributions should have been only marginally affected.

**Supporting References**

1. Troggio M, Malacarne G, Coppola G, Segala C, Cartwright DA et al. (2007) A Dense Single-Nucleotide Polymorphism-Based Genetic Linkage Map of Grapevine (*Vitis vinifera* L.) Anchoring Pinot Noir Bacterial Artificial Chromosome Contigs. Genetics 176: 2637-2650.

2. Feschotte C, Jiang N, Wessler SR (2002) Plant transposable elements: where genetics meets genomics. Nat Rev Genet 3: 329-341.

3. Tamura K, Dudley J, Nei M, Kumar S (2007) MEGA4: Molecular Evolutionary Genetics Analysis (MEGA) Software Version 4.0. Mol Biol Evol

4. Thompson JD, Higgins DG, Gibson TJ (1994) CLUSTAL W: improving the sensitivity of progressive multiple sequence alignment through sequence weighting, position-specific gap penalties and weight matrix choice. Nucleic Acids Res 22: 4673-4680.

5. Notredame C, Higgins DG, Heringa J (2000) T-Coffee: A novel method for fast and accurate multiple sequence alignment. J Mol Biol 302: 205-217.

6. Finn RD, Mistry J, Schuster-Bockler B, Griffiths-Jones S, Hollich V et al. (2006) Pfam: clans, web tools and services. Nucleic Acids Res 34: D247-251.

7. Zuker M (2003) Mfold web server for nucleic acid folding and hybridization prediction. Nucleic Acids Res 31: 3406-3415.

8. Lowe T, Eddy S (1997) tRNAscan-SE: a program for improved detection of transfer RNA genes in genomic sequence. Nucleic Acids Res 25: 955-964.

9. Wang BB, Brendel V (2004) The ASRG database: identification and survey of *Arabidopsis thaliana* genes involved in pre-mRNA splicing. Genome Biol 5: R102.

10. Brown JW, Clark GP, Leader DJ, Simpson CG, Lowe T (2001) Multiple snoRNA gene clusters from *Arabidopsis*. RNA 7: 1817-1832.
