# Supplementary figures and images for: A High Quality Draft Consensus Sequence of the Genome of a Heterozygous Grapevine Variety
Source: PLoS One. 2007 Dec 19;2(12):e1326. doi: 10.1371/journal.pone.0001326 (PMC2147077; doi:10.1371/journal.pone.0001326)

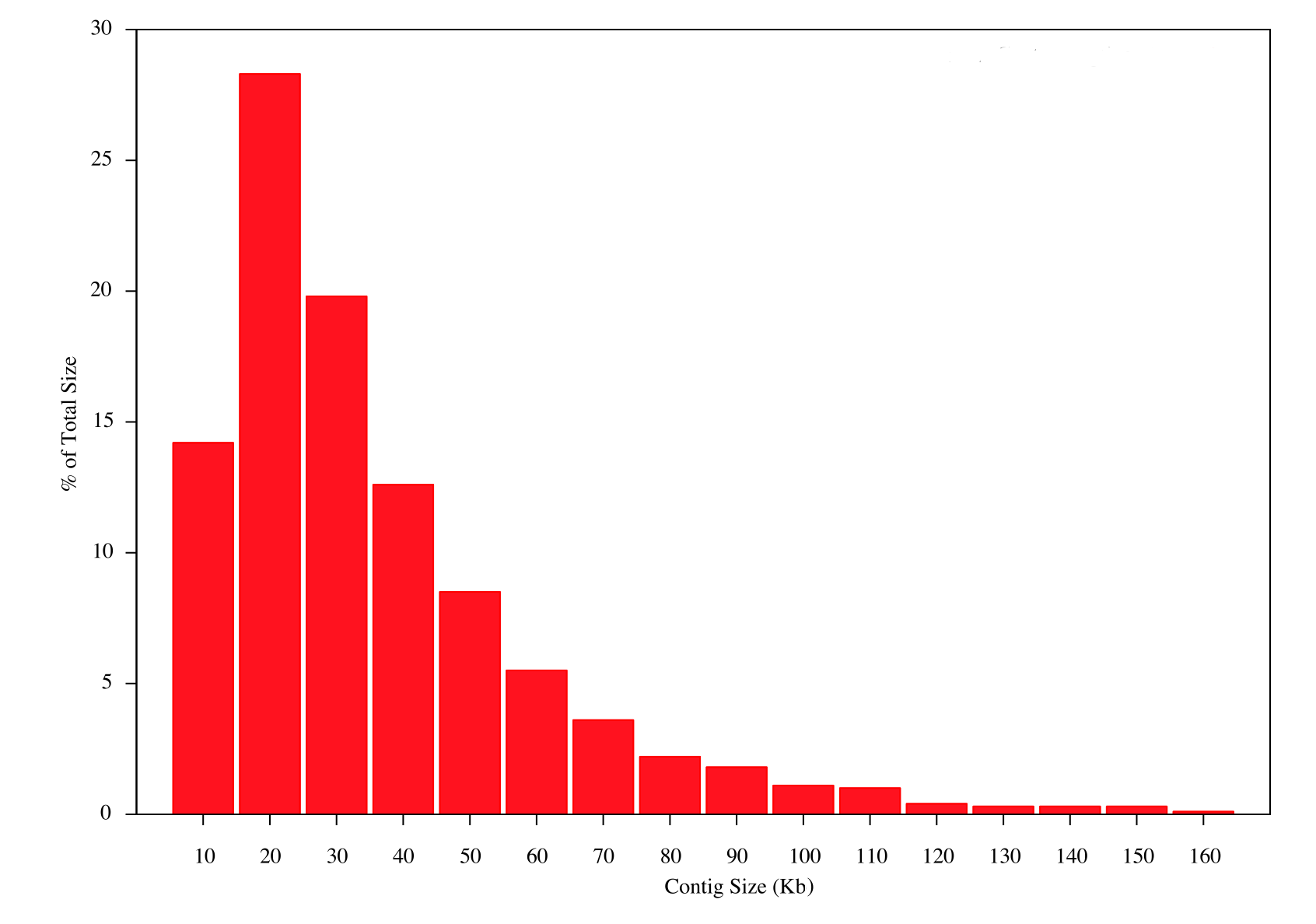

Supplement: Figure S1. — Histograms of contig size distribution. Histograms showing the distribution of the assembled contigs in size classes. The average contig size is 9.1 Kb. Half of the genome is covered by 7,878 contigs larger than 18.2 Kb. (0.08 MB TIF) [file pone.0001326.s002.tif]

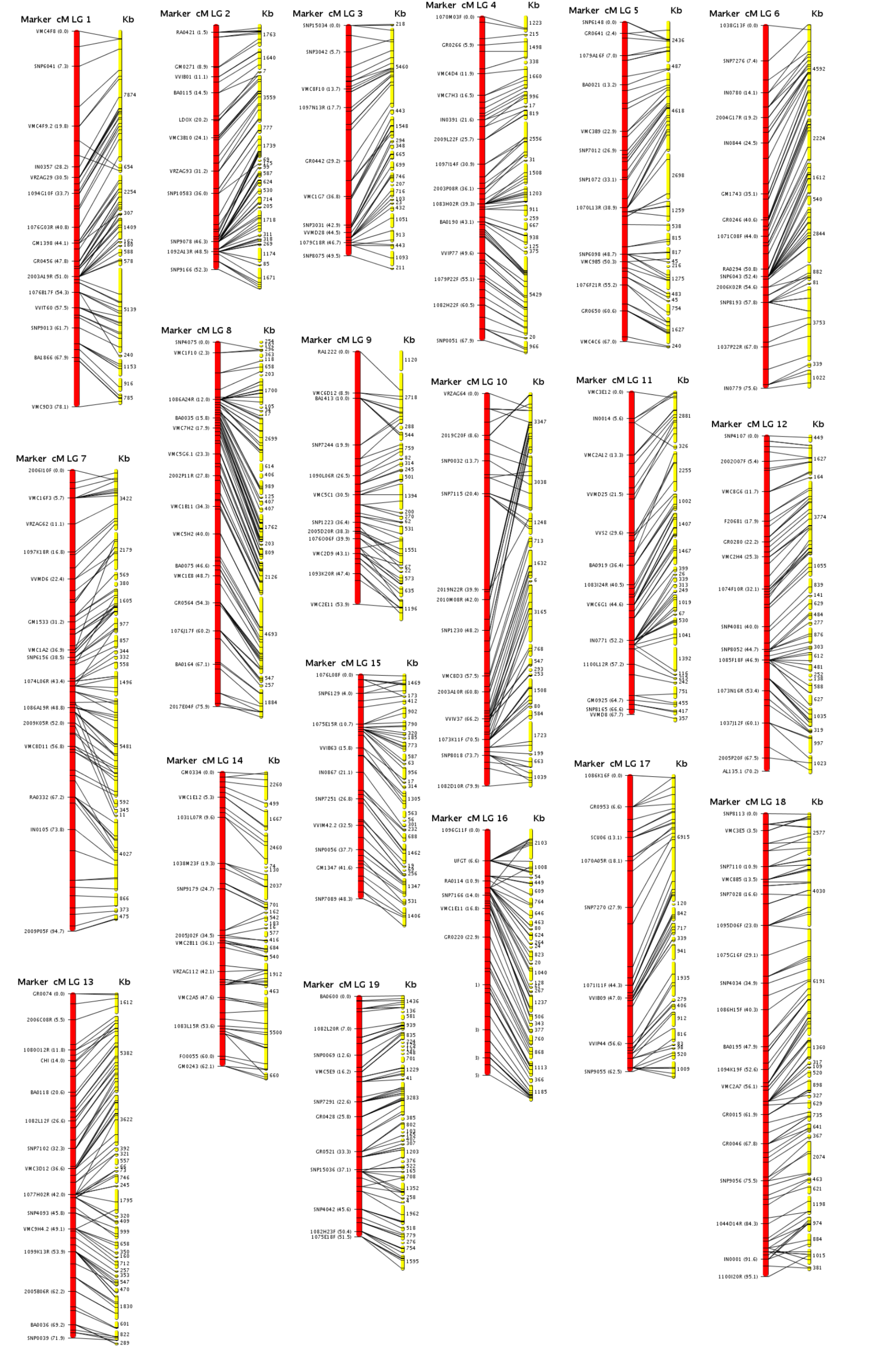

Supplement: Figure S2. — Anchored and oriented metacontigs along the 19 LGs. Representation of the 435.1 Mb of V. vinifera genomic sequence contained in 397 metacontigs aligned and oriented to the genetic map of the 19 LGs. Distances (shown in brackets on the left for some markers) refer to Troggio et al.'s dense map [1] (http://genomics.research.iasma.it). Most metacontigs were anchored to the map using markers with unique sequence locations: SSRs, BAC-end sequences or SNP-based markers derived from either ESTs or assembled sequences of the two haplotypes of the Pinot Noir genome. Metacontigs with no marker information were associated to other metacontigs anchored to the map. There are reliable links between them but they are not merged for several reasons, i.e., too large an overlap between them due to some contigs at the end of one metacontig not being in the proper place; gaps too large due to missing contigs; poor quality or insufficient number of links. Approximate size in Kb of each metacontig is indicated on the right. Gaps separating metacontigs are of undefined size. (2.27 MB TIF) [file pone.0001326.s003.tif]

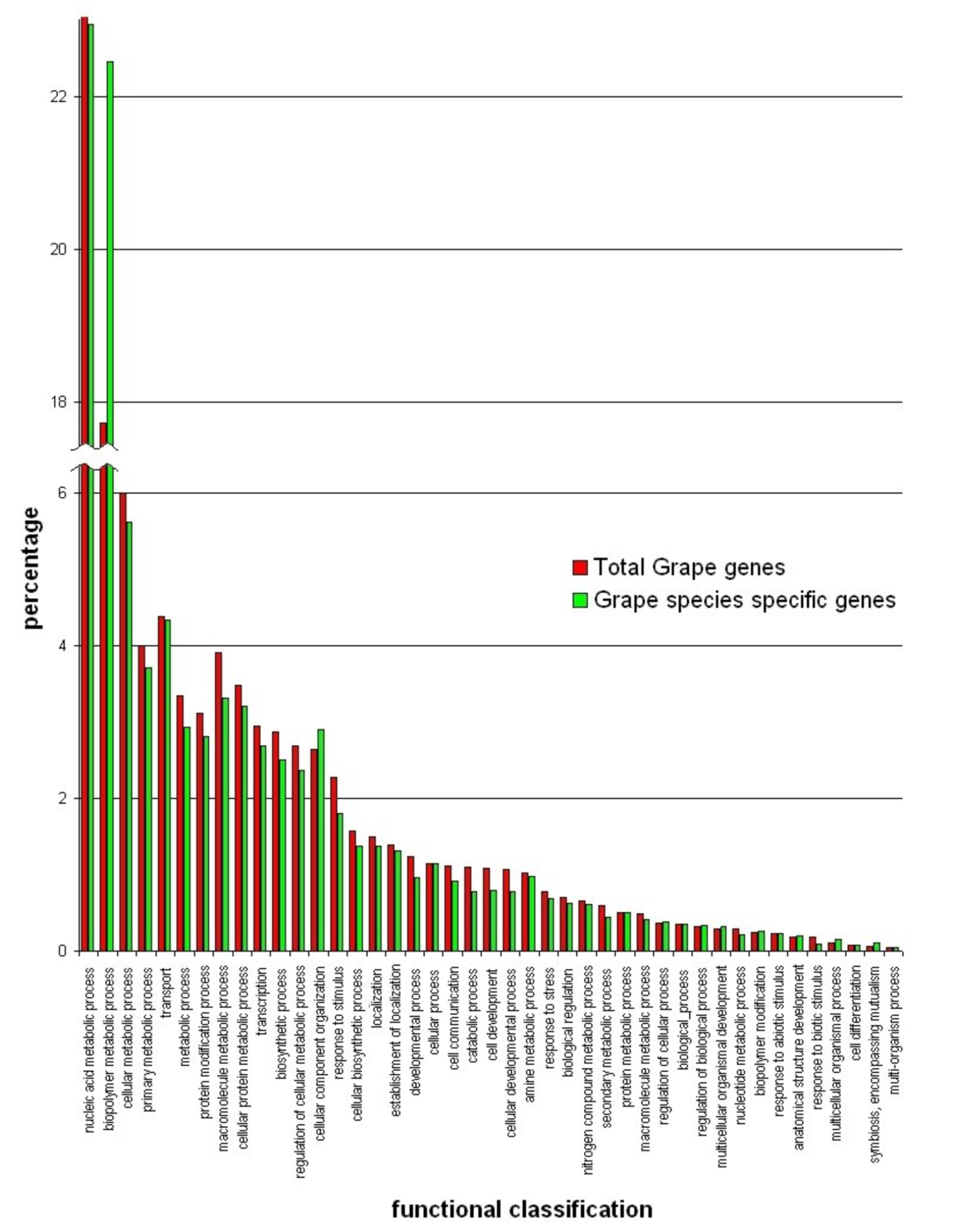

Supplement: Figure S3. — Grape gene class assignment based on putative function. Functional classification of putative grape genes (total and grape-specific) based on Gene Ontology (www.geneontology.org). (4.92 MB TIF) [file pone.0001326.s004.tif]

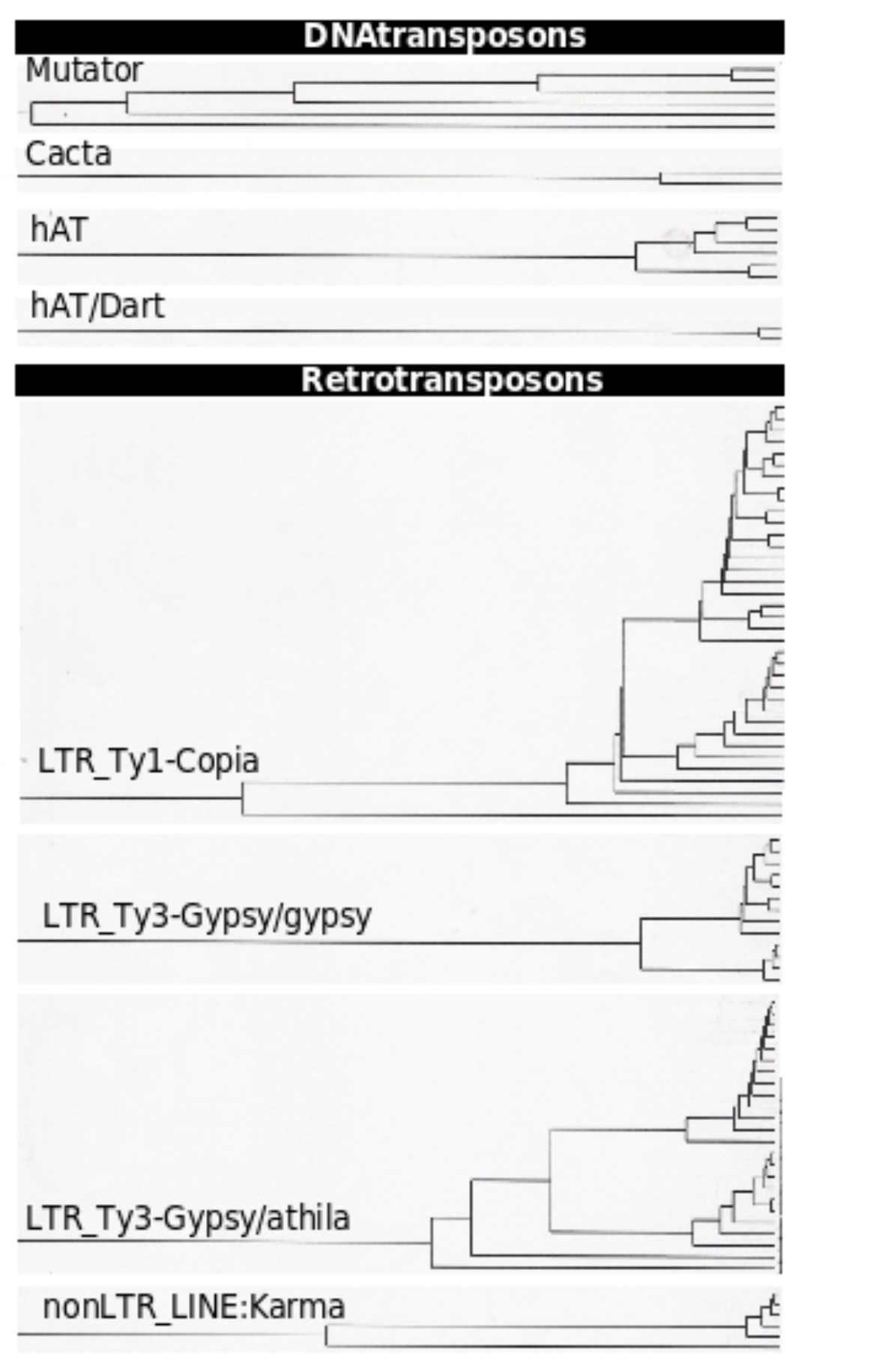

Supplement: Figure S4. — Repetitive element classification and clustering. Phenograms showing the relative similarities of 95 types of repetitive elements out of the 136 identified in the assembled V. vinifera genome. The remaining 41 repeat types (without ORFs or with ORFs shorter than 200 bp) are not included. Repeat types were classified according to Feschotte et al. [2]. Clustering was performed by an all vs all comparison using the BLAST program and was visualized by DrawTree (Myriad Genetics, Salt Lake City, Utah). (0.65 MB TIF) [file pone.0001326.s005.tif]

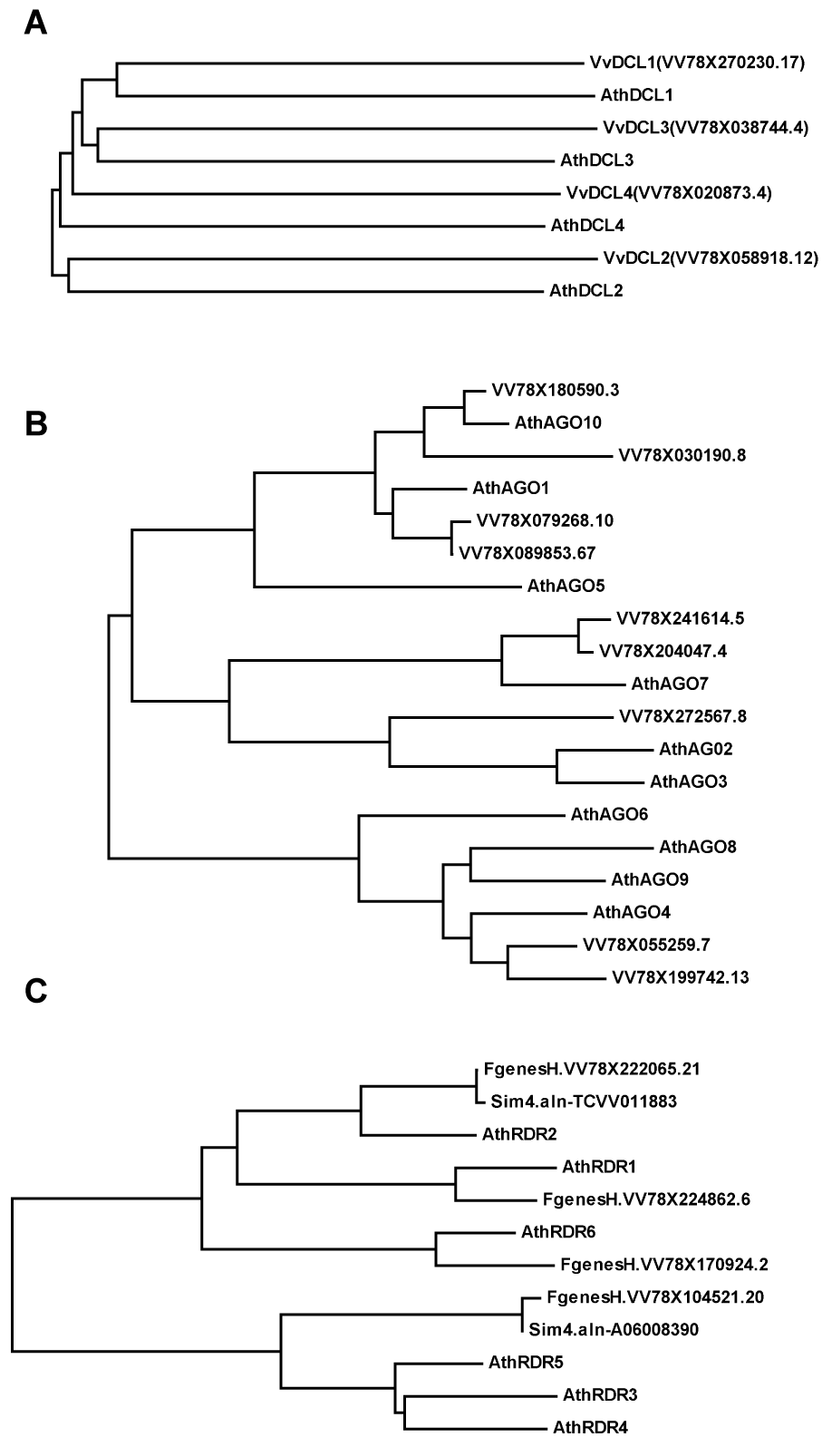

Supplement: Figure S5. — Major RNA silencing proteins present in V. vinifera. Major proteins participating in the RNA silencing pathways in V. vinifera have been identified by homology to Arabidopsis proteins using tBLASTN against the V. vinifera genome. Coding sequences of predicted genes were verified in the TC trancripts database. Protein alignments and trees were performed using MEGA version 4 [3]. Aligned protein sequences are: A) Dicer-like proteins (DCLs). Aligned protein sequences are Vitis vinifera putative Dicer-like proteins and A. thaliana DCL1 (At1g01040/Q9SP32), DCL2 (At3g03300/NP_566199), DCL3 (At3g43920/NP_189978) and DCL4 (At5g20320/AAZ80387). B) AGO proteins. Aligned protein sequences are V. vinifera putative Argonaute proteins and A. thaliana AGO1 (At1g48410/NP_849784), AGO2 (At1g31280/NP_174413), AGO3 (At1g31290/NP_174414), AGO4 (At2g27040/NP_565633), AGO5 (At2g27880/Q9SJK3), AGO6 (At2g32940/NP_180853), AGO7 (At1g69440/AAQ92355), AGO8 (At5g21030/NP_197602), AGO9 (At5g21150/CAD66636), and AGO10 (At5g43810/Q9XGW1). C) RNA-dependent-RNA-polymerases (RDRs). Aligned proteins are V. vinifera putative RDRs and A. thaliana RDR1 (AT1g14790/NP_172932), RDR2 (AT4g11130/NP_192851), RDR3 (AT2g19910/NP_179581), RDR4 (AT2g19920/NP_179582), RDR5 (AT2g19930/ NP_179583), and RDR6 (AT3g49500/NP_190519). (0.11 MB TIF) [file pone.0001326.s006.tif]

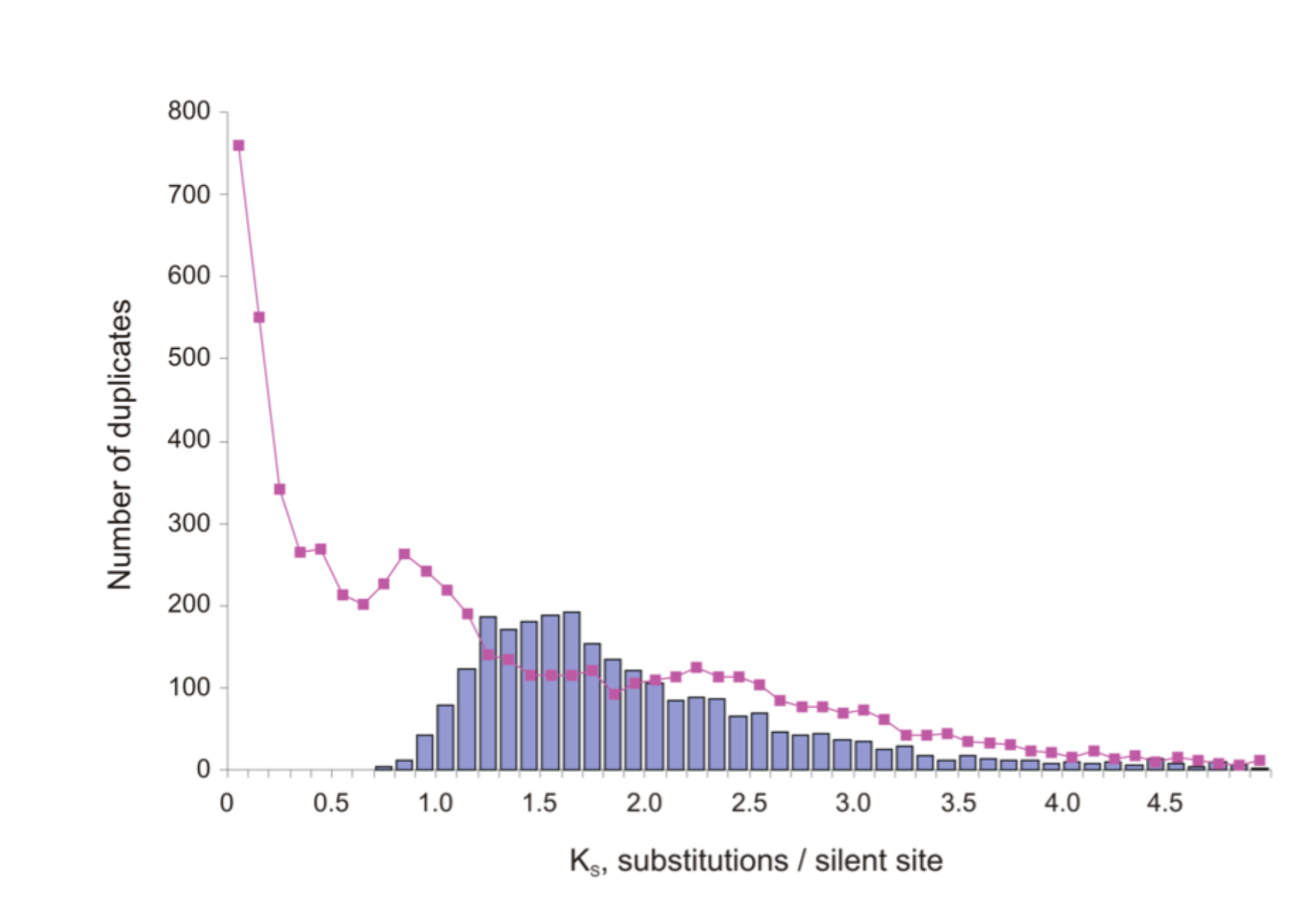

Supplement: Figure S6. — Duplicated state of the grape genome. Age distributions of Vitis paralogs (pink line) and Vitis-Arabidopsis orthologs (blue bins). (0.40 MB TIF) [file pone.0001326.s007.tif]
