# Supplementary material for: A High Quality Draft Consensus Sequence of the Genome of a Heterozygous Grapevine Variety
Source: PLoS One. 2007 Dec 19;2(12):e1326. doi: 10.1371/journal.pone.0001326 (PMC2147077; doi:10.1371/journal.pone.0001326)
Supplement: Table S1. — Details of libraries used in sequencing and estimation of the V. vinifera genome coverage. (0.03 MB DOC) [file pone.0001326.s008.doc]

**Table S1.** Details of libraries used in sequencing and estimation of the *V. vinifera* genome coverage.

| **Insert size (Kb)** | **Libraries**  **(no.)** | **Reads no. (millions)** | **Q20 bases (millions)** | **Read coverage** | **Clone coverage** |
| --- | --- | --- | --- | --- | --- |
| 1.5 - 2.5 | 6 | 2.899 | 1,710.3 | 3.42 | 5.70 |
| 2.0 – 3.0 | 7 | 1.094 | 673.1 | 1.35 | 2.80 |
| 2.5 – 3.5 | 4 | 0.203 | 101.9 | 0.20 | 0.51 |
| 3.0 – 4.0 | 5 | 0.535 | 297.0 | 0.59 | 1.75 |
| 4.0 – 5.0 | 6 | 0.511 | 291.2 | 0.58 | 2.16 |
| 5.0 – 6.0 | 4 | 0.049 | 24.6 | 0.05 | 0.22 |
| 6.0 – 8.0 | 4 | 0.084 | 46.3 | 0.09 | 0.54 |
| 8.0 – 10.0 | 3 | 0.002 | 1.3 | 0.003 | 0.02 |
| 10.0 – 20.0 | 1 | 0.577 | 300.1 | 0.60 | 7.50 |
| 30.0 – 50.0 | 1 fosmid | 0.144 | 66.2 | 0.13 | 4.41 |
| 70.0 – 180.0 | 2 BAC | 0.068 | 25.1 | 0.05 | 4.20 |
