# Supplementary material for: A High Quality Draft Consensus Sequence of the Genome of a Heterozygous Grapevine Variety
Source: PLoS One. 2007 Dec 19;2(12):e1326. doi: 10.1371/journal.pone.0001326 (PMC2147077; doi:10.1371/journal.pone.0001326)
Supplement: Table S2. — Summary of the whole genome shotgun assembly of V. vinifera. (0.04 MB DOC) [file pone.0001326.s009.doc]

**Table S2. Summary of the whole genome shotgun assembly of *V. vinifera***.

|  | **Total** | | |  | **Anchored** | |  | **Unanchored** | |
| --- | --- | --- | --- | --- | --- | --- | --- | --- | --- |
|  | **Contigs**  **(no.)** | **Contigs in metacontigs** | **Metacontigs** |  | **Contigs** | **Metacontigs** |  | **Contigs** | **Metacontigs** |
| **Total number** | 58,611 | 44,179 | 2,093 |  | 37,910 | 397 |  | 20,701 | 1,696 |
| **Total length (Kb)** | 530,852 | 477,100 | 421,300^1^ |  | 435,146 | 360,478^1^ |  | 95,713 | 60,822 |
| **N50 number** | 7,878 | 6,581 | 81 |  | 5741 | 60 |  | 3,054 | 163 |
| **N50 length (Kb)** | 18.2 | 20.0 | 1,330.3 |  | 21.2 | 1,589.8 |  | 8.2 | 106.5 |
| **Largest length (Kb)** | 237.9 | 237.9 | 7,798.9 |  | 237.9 | 7,798.9 |  | 143.2 | 102.3 |

^1^ The discrepancy between total contig length and total metacontig length can be explained by : 1) 14,432 contigs are single-haplotypes contigs; 2) regions with large and multiple gaps having several alternatives; and 3) incorrect offset between contigs in case of large heterozygous gaps.
