# Supplementary material for: A High Quality Draft Consensus Sequence of the Genome of a Heterozygous Grapevine Variety
Source: PLoS One. 2007 Dec 19;2(12):e1326. doi: 10.1371/journal.pone.0001326 (PMC2147077; doi:10.1371/journal.pone.0001326)
Supplement: Table S3. — (0.10 MB DOC) [file pone.0001326.s010.doc]

**Table S3**. Resistance-related genes of *V. vinifera*.

| **Vitis_Gene_ID** | **RGA_ID** | **RGA** | **Clade** | **Colour** | **Group** | **LG** | **Mb** | **Direction** | **Disease Resistance** | **NB-ARC** | **TIR** | **LRR1** | **LRR3** | **LRR** |
| --- | --- | --- | --- | --- | --- | --- | --- | --- | --- | --- | --- | --- | --- | --- |
| FgenesH.VV78X126351.9_5 | RGA001 | 1 | 5 | red | NBS-LRR | 1 | 2,1 | 1 | IPR000767 | IPR002182 |  | IPR001611 |  |  |
| FgenesH.VV78X071402.8_1 | RGA002 | 2 | - | white | NBS | 1 | 2,3 | 1 | IPR000767 | IPR002182 |  |  |  |  |
| FgenesH.VV78X005680.17_8 | RGA003 | 3 | 3 | yellow | CC-NBS-LRR | 1 | 2,4 | 1 | IPR000767 | IPR002182 |  | IPR001611 |  |  |
| Sim4.aln-TCVV019843 | RGA004 | 4 | - | white | NBS | 1 | 9,1 | -1 | IPR000767 | IPR002182 |  |  |  |  |
| FgenesH.VV78X148541.8_4 | RGA005 | 5 | 1 | blue | TIR-NBS-LRR | 1 | 28,6 | 1 | IPR000767 | IPR002182 | IPR000157 | IPR001611 |  | IPR003591 |
| FgenesH.VV78X022182.39_3 | RGA006 | 6 | 1 | blue | TIR-NBS-LRR | 1 | 28,6 | -1 | IPR000767 | IPR002182 | IPR000157 | IPR001611 |  | IPR003591 |
| FgenesH.VV78X208093.9_1 | RGA007 | 7 | - | white | NBS | 1 | 50,1 | -1 |  | IPR002182 |  |  |  |  |
| FgenesH.VV78X210944.11_3 | RGA008 | 8 | 3 | yellow | CC-NBS-LRR | 1 | 58,6 | -1 | IPR000767 | IPR002182 |  | IPR001611 |  |  |
| FgenesH.VV78X210944.11_4 | RGA009 | 9 | 3 | yellow | CC-NBS-LRR | 1 | 58,7 | -1 | IPR000767 | IPR002182 |  | IPR001611 |  |  |
| FgenesH.VV78X260437.23_3 | RGA010 | 10 | 3 | yellow | CC-NBS-LRR | 2 | 10,9 | -1 | IPR000767 | IPR002182 |  | IPR001611 |  |  |
| FgenesH.VV78X184721.3_1 | RGA011 | 11 | 1 | blue | TIR-NBS-LRR | 2 | 29,6 | -1 | IPR000767 | IPR002182 | IPR000157 | IPR001611 | IPR011713 | IPR003591 |
| FgenesH.VV78X003499.5_1 | RGA012 | 12 | 1 | blue | TIR-NBS-LRR | 2 | 30,3 | 1 | IPR000767 | IPR002182 | IPR000157 | IPR001611 | IPR011713 | IPR003591 |
| FgenesH.VV78X129992.3_3 | RGA013 | 13 | 4 | cyan | NBS-LRR | 2 | 30,4 | -1 | IPR000767 | IPR002182 |  | IPR001611 |  |  |
| FgenesH.VV78X023046.6_1 | RGA014 | 14 | 1 | blue | TIR-NBS-LRR | 2 | 30,6 | 1 | IPR000767 | IPR002182 | IPR000157 | IPR001611 | IPR011713 |  |
| FgenesH.VV78X012363.25_1 | RGA015 | 15 | 1 | blue | TIR-NBS-LRR | 2 | 30,6 | -1 | IPR000767 | IPR002182 | IPR000157 | IPR001611 | IPR011713 | IPR003591 |
| FgenesH.VV78X104210.12_3 | RGA016 | 16 | 4 | cyan | NBS-LRR | 2 | 30,7 | -1 | IPR000767 | IPR002182 |  | IPR001611 |  |  |
| FgenesH.VV78X178162.7_1 | RGA017 | 17 | 5 | red | CC-NBS-LRR | 3 | 2,1 | -1 | IPR000767 | IPR002182 |  |  |  |  |
| FgenesH.VV78X275204.13_9 | RGA018 | 18 | 5 | red | CC-NBS-LRR | 3 | 2,3 | -1 | IPR000767 | IPR002182 |  | IPR001611 |  |  |
| FgenesH.VV78X003983.6_1 | RGA019 | 19 | - | white | NBS | 3 | 2,4 | -1 | IPR000767 | IPR002182 |  |  |  |  |
| FgenesH.VV78X003983.6_2 | RGA020 | 20 | 5 | red | NBS-LRR | 3 | 2,4 | -1 | IPR000767 | IPR002182 |  | IPR001611 |  |  |
| FgenesH.VV78X115004.17_4 | RGA021 | 21 | 5 | red | CC-NBS-LRR | 3 | 2,4 | 1 | IPR000767 | IPR002182 |  |  |  |  |
| FgenesH.VV78X195327.12_1 | RGA022 | 22 | 1 | blue | NBS-LRR | 3 | 2,4 | -1 | IPR000767 | IPR002182 |  | IPR001611 |  |  |
| FgenesH.VV78X246325.2_1 | RGA023 | 23 | - | white | NBS | 3 | 2,6 | 1 | IPR000767 | IPR002182 |  |  |  |  |
| FgenesH.VV78X071823.11_1 | RGA024 | 24 | - | white | NBS | 3 | 2,6 | 1 | IPR000767 | IPR002182 |  |  |  |  |
| FgenesH.VV78X071823.11_4 | RGA025 | 25 | 5 | red | CC-NBS-LRR | 3 | 2,6 | 1 | IPR000767 | IPR002182 |  | IPR001611 |  |  |
| Sim4.aln-TCVV013819 | RGA026 | 26 | - | white | NBS | 3 | 2,6 | 1 |  | IPR002182 |  |  |  |  |
| FgenesH.VV78X027394.7_1 | RGA027 | 27 | 4 | cyan | NBS-LRR | 3 | 8,4 | -1 | IPR000767 | IPR002182 |  | IPR001611 |  |  |
| FgenesH.VV78X051009.7_1 | RGA028 | 28 | 4 | cyan | NBS-LRR | 3 | 16,0 | 1 | IPR000767 | IPR002182 |  | IPR001611 |  |  |
| FgenesH.VV78X149600.4_1 | RGA029 | 29 | 2 | green | CC-NBS-LRR | 3 | 43,4 | -1 | IPR000767 | IPR002182 |  | IPR001611 |  |  |
| FgenesH.VV78X192949.27_3 | RGA030 | 30 | - | white | NBS | 3 | 43,5 | 1 | IPR000767 | IPR002182 |  |  |  |  |
| FgenesH.VV78X050682.9_2 | RGA031 | 31 | 5 | red | NBS-LRR | 3 | 43,5 | -1 |  | IPR002182 |  | IPR001611 |  |  |
| FgenesH.VV78X133626.18_2 | RGA032 | 32 | 5 | red | CC-NBS-LRR | 3 | 43,5 | 1 |  | IPR002182 |  | IPR001611 |  |  |
| FgenesH.VV78X192949.20_1 | RGA033 | 33 | 2 | green | CC-NBS-LRR | 3 | 43,5 | -1 | IPR000767 | IPR002182 |  |  |  |  |
| FgenesH.VV78X138606.12_6 | RGA034 | 34 | 5 | red | NBS-LRR | 5 | 14,3 | 1 |  | IPR002182 |  | IPR001611 |  |  |
| FgenesH.VV78X158691.20_3 | RGA035 | 35 | 5 | red | NBS-LRR | 5 | 17,6 | -1 | IPR000767 | IPR002182 |  | IPR001611 |  |  |
| FgenesH.VV78X249650.8_1 | RGA036 | 36 | 1 | blue | TIR-NBS-LRR | 5 | 28,2 | 1 | IPR000767 | IPR002182 | IPR000157 | IPR001611 | IPR011713 | IPR003591 |
| FgenesH.VV78X093335.6_1 | RGA037 | 37 | 1 | blue | TIR-NBS-LRR | 5 | 28,3 | 1 | IPR000767 | IPR002182 | IPR000157 | IPR001611 | IPR011713 | IPR003591 |
| FgenesH.VV78X015844.10_1 | RGA038 | 38 | - | white | NBS | 5 | 28,3 | -1 |  | IPR002182 |  |  |  |  |
| FgenesH.VV78X125512.5_1 | RGA039 | 39 | 1 | blue | TIR-NBS-LRR | 5 | 28,4 | -1 | IPR000767 | IPR002182 | IPR000157 | IPR001611 | IPR011713 | IPR003591 |
| FgenesH.VV78X070583.19_3 | RGA040 | 40 | 5 | red | CC-NBS-LRR | 5 | 34,1 | 1 | IPR000767 | IPR002182 |  | IPR001611 |  |  |
| FgenesH.VV78X193990.5_2 | RGA041 | 41 | 5 | red | CC-NBS-LRR | 5 | 37,6 | -1 | IPR000767 | IPR002182 |  |  |  |  |
| FgenesH.VV78X193990.5_6 | RGA042 | 42 | - | white | NBS | 5 | 37,6 | -1 | IPR000767 | IPR002182 |  |  |  |  |
| Sim4.aln-A06016414 | RGA043 | 43 | - | white | NBS | 5 | 37,6 | -1 |  | IPR002182 |  |  |  |  |
| FgenesH.VV78X015687.5_3 | RGA044 | 44 | - | white | NBS | 5 | 37,7 | -1 |  | IPR002182 |  |  |  |  |
| FgenesH.VV78X239622.11_4 | RGA045 | 45 | 4 | cyan | NBS-LRR | 5 | 38,8 | -1 |  | IPR002182 |  | IPR001611 |  |  |
| FgenesH.VV78X139260.10_1 | RGA046 | 46 | 5 | red | NBS-LRR | 5 | 60,5 | -1 | IPR000767 | IPR002182 |  | IPR001611 |  |  |
| FgenesH.VV78X102894.12_2 | RGA047 | 47 | 2 | green | CC-NBS-LRR | 5 | 60,6 | -1 |  | IPR002182 |  | IPR001611 |  |  |
| FgenesH.VV78X035788.5_1 | RGA048 | 48 | 2 | green | CC-NBS-LRR | 5 | 60,8 | 1 | IPR000767 | IPR002182 |  | IPR001611 |  |  |
| FgenesH.VV78X067887.5_2 | RGA049 | 49 | 5 | red | CC-NBS-LRR | 5 | 61,1 | 1 | IPR000767 | IPR002182 |  | IPR001611 |  |  |
| FgenesH.VV78X067887.5_3 | RGA050 | 50 | 2 | green | CC-NBS-LRR | 5 | 61,1 | 1 | IPR000767 | IPR002182 |  | IPR001611 |  |  |
| FgenesH.VV78X118651.5_1 | RGA051 | 51 | 5 | red | NBS-LRR | 6 | 31,2 | -1 | IPR000767 | IPR002182 |  | IPR001611 |  |  |
| FgenesH.VV78X110776.6_1 | RGA052 | 52 | 5 | red | NBS-LRR | 6 | 33,2 | 1 | IPR000767 | IPR002182 |  | IPR001611 |  |  |
| FgenesH.VV78X251728.45_3 | RGA053 | 53 | 2 | green | CC-NBS-LRR | 6 | 38,6 | 1 | IPR000767 | IPR002182 |  | IPR001611 |  |  |
| FgenesH.VV78X102540.11_1 | RGA054 | 54 | 2 | green | CC-NBS-LRR | 6 | 38,8 | -1 | IPR000767 | IPR002182 |  | IPR001611 |  |  |
| FgenesH.VV78X248343.6_2 | RGA055 | 55 | 5 | red | NBS-LRR | 6 | 51,6 | 1 | IPR000767 | IPR002182 |  | IPR001611 |  |  |
| FgenesH.VV78X101301.8_1 | RGA056 | 56 | 5 | red | NBS-LRR | 7 | 28,0 | -1 | IPR000767 | IPR002182 |  | IPR001611 |  |  |
| FgenesH.VV78X101301.8_2 | RGA057 | 57 | 5 | red | NBS-LRR | 7 | 28,0 | -1 | IPR000767 | IPR002182 |  | IPR001611 |  |  |
| FgenesH.VV78X019431.42_1 | RGA058 | 58 | - | white | NBS | 7 | 28,1 | -1 |  | IPR002182 |  |  |  |  |
| FgenesH.VV78X078454.13_2 | RGA059 | 59 | 5 | red | NBS-LRR | 7 | 30,7 | 1 | IPR000767 | IPR002182 |  | IPR001611 |  |  |
| FgenesH.VV78X069700.8_1 | RGA060 | 60 | 3 | yellow | CC-NBS-LRR | 7 | 35,7 | -1 | IPR000767 | IPR002182 |  | IPR001611 |  |  |
| FgenesH.VV78X083131.15_1 | RGA061 | 61 | 4 | cyan | NBS-LRR | 7 | 38,3 | -1 | IPR000767 | IPR002182 |  | IPR001611 |  |  |
| FgenesH.VV78X082809.15_3 | RGA062 | 62 | - | white | NBS | 7 | 38,4 | 1 | IPR000767 | IPR002182 |  |  |  |  |
| FgenesH.VV78X185588.23_1 | RGA063 | 63 | 4 | cyan | NBS-LRR | 7 | 41,7 | 1 | IPR000767 | IPR002182 |  | IPR001611 |  |  |
| FgenesH.VV78X248660.7_1 | RGA064 | 64 | 5 | red | CC-NBS-LRR | 7 | 44,8 | 1 | IPR000767 | IPR002182 |  | IPR001611 |  |  |
| Sim4.aln-TCVV023138 | RGA065 | 65 | 5 | red | NBS-LRR | 7 | 44,8 | 1 | IPR000767 | IPR002182 |  | IPR001611 |  |  |
| FgenesH.VV78X250222.4_1 | RGA066 | 66 | 5 | red | CC-NBS-LRR | 7 | 44,9 | 1 | IPR000767 | IPR002182 |  | IPR001611 |  |  |
| FgenesH.VV78X153300.16_1 | RGA067 | 67 | 1 | blue | TIR-NBS-LRR | 7 | 51,2 | 1 | IPR000767 | IPR002182 | IPR000157 | IPR001611 |  | IPR003591 |
| FgenesH.VV78X127313.6_2 | RGA068 | 68 | 4 | cyan | NBS-LRR | 7 | 58,2 | -1 | IPR000767 | IPR002182 |  | IPR001611 |  |  |
| FgenesH.VV78X276947.32_1 | RGA069 | 69 | - | white | NBS | 7 | 58,3 | -1 | IPR000767 | IPR002182 |  |  |  |  |
| Glimmer1.VV78X127313.6_3 | RGA070 | 70 | 4 | cyan | NBS-LRR | 7 | 58,3 | -1 | IPR000767 | IPR002182 |  | IPR001611 |  |  |
| FgenesH.VV78X276947.32_3 | RGA071 | 71 | - | white | NBS | 7 | 58,3 | -1 |  | IPR002182 |  |  |  |  |
| FgenesH.VV78X276947.32_4 | RGA072 | 72 | - | white | NBS | 7 | 58,3 | -1 |  | IPR002182 |  |  |  |  |
| FgenesH.VV78X267550.21_1 | RGA073 | 73 | 5 | red | NBS-LRR | 8 | 36,4 | 1 | IPR000767 | IPR002182 |  | IPR001611 |  | IPR003591 |
| FgenesH.VV78X164499.37_1 | RGA074 | 74 | - | white | NBS | 8 | 36,4 | 1 | IPR000767 | IPR002182 |  |  |  |  |
| FgenesH.VV78X189970.2_1 | RGA075 | 75 | 5 | red | CC-NBS-LRR | 8 | 36,4 | 1 | IPR000767 | IPR002182 |  | IPR001611 |  | IPR003591 |
| FgenesH.VV78X017515.3_2 | RGA076 | 76 | 5 | red | NBS-LRR | 8 | 36,5 | 1 | IPR000767 | IPR002182 |  | IPR001611 |  | IPR003591 |
| FgenesH.VV78X053358.9_4 | RGA077 | 77 | 5 | red | CC-NBS-LRR | 9 | 5,5 | 1 | IPR000767 | IPR002182 |  | IPR001611 |  |  |
| FgenesH.VV78X268761.4_1 | RGA078 | 78 | 5 | red | NBS-LRR | 9 | 8,0 | -1 |  | IPR002182 |  | IPR001611 |  |  |
| FgenesH.VV78X268761.4_2 | RGA079 | 79 | 5 | red | NBS-LRR | 9 | 8,1 | -1 |  | IPR002182 |  | IPR001611 |  |  |
| FgenesH.VV78X062729.15_1 | RGA080 | 80 | 5 | red | NBS-LRR | 9 | 8,2 | -1 |  | IPR002182 |  | IPR001611 |  |  |
| FgenesH.VV78X062729.15_2 | RGA081 | 81 | 5 | red | CC-NBS-LRR | 9 | 8,2 | -1 | IPR000767 | IPR002182 |  | IPR001611 |  |  |
| FgenesH.VV78X150846.7_1 | RGA082 | 82 | 5 | red | CC-NBS-LRR | 9 | 8,2 | 1 | IPR000767 | IPR002182 |  | IPR001611 |  |  |
| Sim4.aln-TCVV004569 | RGA083 | 83 | - | white | NBS | 9 | 8,2 | 1 |  | IPR002182 |  |  |  |  |
| FgenesH.VV78X062729.15_3 | RGA084 | 84 | 5 | red | CC-NBS-LRR | 9 | 8,2 | -1 | IPR000767 | IPR002182 |  | IPR001611 |  |  |
| FgenesH.VV78X049616.5_1 | RGA085 | 85 | 5 | red | CC-NBS-LRR | 9 | 8,2 | -1 | IPR000767 | IPR002182 |  | IPR001611 |  |  |
| FgenesH.VV78X037181.25_1 | RGA086 | 86 | 5 | red | CC-NBS-LRR | 9 | 9,0 | 1 | IPR000767 | IPR002182 |  | IPR001611 |  |  |
| Glimmer1.VV78X096440.7_2 | RGA087 | 87 | - | white | NBS | 9 | 9,6 | 1 | IPR000767 | IPR002182 |  |  |  |  |
| FgenesH.VV78X194565.7_1 | RGA088 | 88 | 5 | red | CC-NBS-LRR | 9 | 10,8 | -1 | IPR000767 | IPR002182 |  | IPR001611 |  |  |
| FgenesH.VV78X084754.24_2 | RGA089 | 89 | 5 | red | CC-NBS-LRR | 9 | 10,9 | -1 | IPR000767 | IPR002182 |  | IPR001611 |  |  |
| FgenesH.VV78X085337.4_1 | RGA090 | 90 | 5 | red | NBS-LRR | 9 | 10,9 | 1 | IPR000767 | IPR002182 |  | IPR001611 |  |  |
| FgenesH.VV78X221714.8_1 | RGA091 | 91 | - | white | NBS | 9 | 10,9 | -1 |  | IPR002182 |  |  |  |  |
| FgenesH.VV78X128663.5_1 | RGA092 | 92 | 5 | red | NBS-LRR | 9 | 11,0 | 1 | IPR000767 | IPR002182 |  | IPR001611 |  |  |
| FgenesH.VV78X128663.5_2 | RGA093 | 93 | 5 | red | CC-NBS-LRR | 9 | 11,0 | 1 | IPR000767 | IPR002182 |  |  |  |  |
| FgenesH.VV78X006995.8_1 | RGA094 | 94 | - | white | NBS | 9 | 11,0 | 1 |  | IPR002182 |  |  |  |  |
| FgenesH.VV78X234265.45_3 | RGA095 | 95 | 5 | red | CC-NBS-LRR | 9 | 11,0 | -1 | IPR000767 | IPR002182 |  | IPR001611 |  |  |
| FgenesH.VV78X269490.11_2 | RGA096 | 96 | - | white | NBS | 9 | 11,0 | 1 | IPR000767 | IPR002182 |  |  |  |  |
| FgenesH.VV78X258052.13_1 | RGA097 | 97 | 5 | red | CC-NBS-LRR | 9 | 11,4 | -1 | IPR000767 | IPR002182 |  | IPR001611 |  |  |
| FgenesH.VV78X276463.11_1 | RGA098 | 98 | 5 | red | CC-NBS-LRR | 9 | 13,1 | 1 | IPR000767 | IPR002182 |  | IPR001611 |  |  |
| FgenesH.VV78X228757.7_1 | RGA099 | 99 | 5 | red | CC-NBS-LRR | 9 | 13,5 | 1 | IPR000767 | IPR002182 |  | IPR001611 |  |  |
| FgenesH.VV78X131611.14_2 | RGA100 | 100 | 4 | cyan | CC-NBS-LRR | 9 | 17,0 | -1 | IPR000767 | IPR002182 |  | IPR001611 |  |  |
| FgenesH.VV78X162353.9_2 | RGA101 | 101 | 4 | cyan | CC-NBS-LRR | 9 | 17,8 | -1 | IPR000767 | IPR002182 |  | IPR001611 |  |  |
| FgenesH.VV78X058132.18_2 | RGA102 | 102 | - | white | NBS | 9 | 17,8 | 1 |  | IPR002182 |  |  |  |  |
| FgenesH.VV78X058132.18_1 | RGA103 | 103 | 4 | cyan | CC-NBS-LRR | 9 | 17,8 | 1 | IPR000767 | IPR002182 |  | IPR001611 |  |  |
| FgenesH.VV78X116983.10_2 | RGA104 | 104 | 4 | cyan | CC-NBS-LRR | 9 | 18,1 | 1 | IPR000767 | IPR002182 |  | IPR001611 |  | IPR003591 |
| FgenesH.VV78X119916.16_1 | RGA105 | 105 | 4 | cyan | CC-NBS-LRR | 9 | 18,3 | 1 | IPR000767 | IPR002182 |  | IPR001611 |  | IPR003591 |
| FgenesH.VV78X039958.13_2 | RGA106 | 106 | 4 | cyan | CC-NBS-LRR | 9 | 18,3 | 1 | IPR000767 | IPR002182 |  | IPR001611 |  |  |
| FgenesH.VV78X119916.16_3 | RGA107 | 107 | - | white | NBS | 9 | 18,4 | 1 | IPR000767 | IPR002182 |  |  |  |  |
| FgenesH.VV78X036507.3_2 | RGA108 | 108 | 4 | cyan | NBS-LRR | 9 | 18,4 | -1 | IPR000767 | IPR002182 |  | IPR001611 |  |  |
| FgenesH.VV78X009571.2_2 | RGA109 | 109 | - | white | NBS | 9 | 18,5 | -1 | IPR000767 | IPR002182 |  |  |  |  |
| FgenesH.VV78X063614.6_2 | RGA110 | 110 | 1 | blue | CC-NBS-LRR | 9 | 18,6 | -1 | IPR000767 | IPR002182 |  |  |  |  |
| FgenesH.VV78X088039.13_1 | RGA111 | 111 | 4 | cyan | NBS-LRR | 9 | 18,7 | 1 | IPR000767 | IPR002182 |  | IPR001611 |  |  |
| FgenesH.VV78X152498.11_2 | RGA112 | 112 | 5 | red | NBS-LRR | 9 | 25,9 | 1 | IPR000767 | IPR002182 |  | IPR001611 |  |  |
| Glimmer1.VV78X004134.9_1 | RGA113 | 113 | - | white | NBS | 9 | 30,6 | 1 | IPR000767 | IPR002182 |  |  |  |  |
| FgenesH.VV78X156410.3_5 | RGA114 | 114 | 5 | red | CC-NBS-LRR | 10 | 5,3 | -1 | IPR000767 | IPR002182 |  | IPR001611 |  |  |
| Glimmer1.VV78X127277.18_3 | RGA115 | 115 | 5 | red | NBS-LRR | 10 | 8,2 | -1 | IPR000767 | IPR002182 |  | IPR001611 |  |  |
| FgenesH.VV78X224903.5_1 | RGA116 | 116 | 5 | red | NBS-LRR | 10 | 44,8 | -1 |  | IPR002182 |  | IPR001611 |  |  |
| Glimmer1.VV78X028296.12_2 | RGA117 | 117 | - | white | NBS | 10 | 64,5 | -1 | IPR000767 | IPR002182 |  |  |  |  |
| FgenesH.VV78X010001.4_2 | RGA118 | 118 | 5 | red | CC-NBS-LRR | 11 | 3,0 | 1 | IPR000767 | IPR002182 |  | IPR001611 |  | IPR003591 |
| FgenesH.VV78X234630.4_4 | RGA119 | 119 | - | white | NBS | 11 | 41,2 | -1 | IPR000767 | IPR002182 |  |  |  |  |
| FgenesH.VV78X209776.3_3 | RGA120 | 120 | - | white | NBS | 11 | 42,5 | 1 | IPR000767 | IPR002182 |  |  |  |  |
| FgenesH.VV78X201022.18_1 | RGA121 | 121 | 5 | red | NBS-LRR | 11 | 42,6 | -1 | IPR000767 | IPR002182 |  | IPR001611 |  | IPR003591 |
| FgenesH.VV78X089761.6_4 | RGA122 | 122 | 5 | red | NBS-LRR | 11 | 42,9 | -1 | IPR000767 | IPR002182 |  | IPR001611 |  | IPR003591 |
| FgenesH.VV78X012107.6_3 | RGA123 | 123 | - | black | TIR-NBS | 12 | 23,2 | -1 | IPR000767 | IPR002182 | IPR000157 |  |  |  |
| FgenesH.VV78X194269.2_1 | RGA124 | 124 | - | white | NBS | 12 | 39,1 | -1 |  | IPR002182 |  |  |  |  |
| FgenesH.VV78X230353.8_4 | RGA125 | 125 | - | white | NBS | 12 | 53,5 | -1 |  | IPR002182 |  |  |  |  |
| FgenesH.VV78X183215.6_1 | RGA126 | 126 | - | white | NBS | 12 | 53,5 | -1 | IPR000767 | IPR002182 |  |  |  |  |
| FgenesH.VV78X254581.2_1 | RGA127 | 127 | - | white | NBS | 12 | 54,9 | 1 |  | IPR002182 |  |  |  |  |
| FgenesH.VV78X254581.2_3 | RGA128 | 128 | 4 | cyan | CC-NBS-LRR | 12 | 54,9 | 1 | IPR000767 | IPR002182 |  | IPR001611 |  |  |
| FgenesH.VV78X081614.4_1 | RGA129 | 129 | 4 | cyan | CC-NBS-LRR | 12 | 55,0 | 1 | IPR000767 | IPR002182 |  | IPR001611 |  |  |
| Glimmer1.VV78X254581.2_3 | RGA130 | 130 | 5 | red | NBS-LRR | 12 | 55,0 | 1 | IPR000767 | IPR002182 |  | IPR001611 |  |  |
| FgenesH.VV78X081605.30_2 | RGA131 | 131 | - | white | NBS | 12 | 55,4 | -1 |  | IPR002182 |  |  |  |  |
| FgenesH.VV78X057788.4_1 | RGA132 | 132 | 4 | cyan | CC-NBS-LRR | 12 | 57,4 | -1 | IPR000767 | IPR002182 |  | IPR001611 |  |  |
| FgenesH.VV78X218256.23_1 | RGA133 | 133 | 4 | cyan | NBS-LRR | 12 | 58,2 | -1 | IPR000767 | IPR002182 |  | IPR001611 |  |  |
| FgenesH.VV78X240488.6_1 | RGA134 | 134 | 5 | red | NBS-LRR | 12 | 62,6 | 1 | IPR000767 | IPR002182 |  | IPR001611 |  | IPR003591 |
| FgenesH.VV78X025992.4_2 | RGA135 | 135 | 5 | red | NBS-LRR | 12 | 62,9 | -1 | IPR000767 | IPR002182 |  | IPR001611 |  |  |
| FgenesH.VV78X059161.22_2 | RGA136 | 136 | 5 | red | NBS-LRR | 12 | 63,0 | -1 |  | IPR002182 |  | IPR001611 |  |  |
| Twinscan1.VV79X001149.1_1 | RGA137 | 137 | 5 | red | NBS-LRR | 12 | 63,0 | -1 | IPR000767 | IPR002182 |  | IPR001611 |  |  |
| FgenesH.VV78X235125.7_3 | RGA138 | 138 | 5 | red | NBS-LRR | 12 | 63,4 | -1 |  | IPR002182 |  | IPR001611 |  |  |
| FgenesH.VV78X210166.4_2 | RGA139 | 139 | 5 | red | NBS-LRR | 12 | 63,4 | 1 | IPR000767 | IPR002182 |  | IPR001611 |  |  |
| FgenesH.VV78X195066.3_1 | RGA140 | 140 | 4 | cyan | CC-NBS-LRR | 12 | 64,9 | 1 | IPR000767 | IPR002182 |  | IPR001611 |  |  |
| Sim4.aln-A06016836 | RGA141 | 141 | - | white | NBS | 12 | 64,9 | -1 |  | IPR002182 |  |  |  |  |
| FgenesH.VV78X009812.2_1 | RGA142 | 142 | 4 | cyan | NBS-LRR | 12 | 65,0 | -1 | IPR000767 | IPR002182 |  | IPR001611 |  |  |
| FgenesH.VV78X122122.5_8 | RGA143 | 143 | 5 | red | CC-NBS-LRR | 13 | 1,4 | -1 | IPR000767 | IPR002182 |  | IPR001611 |  |  |
| FgenesH.VV78X171242.6_1 | RGA144 | 144 | 3 | yellow | CC-NBS-LRR | 13 | 20,7 | 1 | IPR000767 | IPR002182 |  | IPR001611 |  |  |
| FgenesH.VV78X222628.7_2 | RGA145 | 145 | 4 | cyan | NBS-LRR | 13 | 40,2 | 1 | IPR000767 | IPR002182 |  | IPR001611 |  |  |
| Sim4.aln-A06008203 | RGA146 | 146 | - | white | NBS | 13 | 40,7 | 1 | IPR000767 | IPR002182 |  |  |  |  |
| Sim4.aln-TCVV018390 | RGA147 | 147 | - | white | NBS | 13 | 40,7 | 1 |  | IPR002182 |  |  |  |  |
| FgenesH.VV78X094332.13_4 | RGA148 | 148 | 4 | cyan | NBS-LRR | 13 | 40,8 | 1 | IPR000767 | IPR002182 |  | IPR001611 |  |  |
| FgenesH.VV78X080871.3_1 | RGA149 | 149 | - | white | NBS | 13 | 48,2 | 1 |  | IPR002182 |  |  |  |  |
| FgenesH.VV78X052634.12_4 | RGA150 | 150 | 4 | cyan | NBS-LRR | 13 | 54,9 | -1 | IPR000767 | IPR002182 |  | IPR001611 |  |  |
| FgenesH.VV78X076993.7_2 | RGA151 | 151 | 4 | cyan | NBS-LRR | 13 | 59,4 | -1 | IPR000767 | IPR002182 |  | IPR001611 |  |  |
| FgenesH.VV78X117472.3_1 | RGA152 | 152 | 4 | cyan | NBS-LRR | 13 | 59,4 | -1 | IPR000767 | IPR002182 |  | IPR001611 |  |  |
| FgenesH.VV78X011476.4_2 | RGA153 | 153 | - | white | NBS | 13 | 59,5 | -1 |  | IPR002182 |  |  |  |  |
| FgenesH.VV78X231194.10_3 | RGA154 | 154 | 4 | cyan | NBS-LRR | 13 | 59,7 | 1 | IPR000767 | IPR002182 |  | IPR001611 |  |  |
| FgenesH.VV78X243986.6_4 | RGA155 | 155 | 1 | blue | CC-NBS-LRR | 13 | 59,8 | -1 | IPR000767 | IPR002182 |  | IPR001611 |  |  |
| FgenesH.VV78X006360.19_1 | RGA156 | 156 | - | white | NBS | 13 | 59,9 | 1 |  | IPR002182 |  |  |  |  |
| FgenesH.VV78X006360.17_2 | RGA157 | 157 | - | white | NBS | 13 | 59,9 | -1 |  | IPR002182 |  |  |  |  |
| Glimmer1.VV78X111276.11_4 | RGA158 | 158 | - | white | NBS | 13 | 60,0 | 1 | IPR000767 | IPR002182 |  |  |  |  |
| FgenesH.VV78X006360.14_3 | RGA159 | 159 | 4 | cyan | NBS-LRR | 13 | 60,1 | -1 | IPR000767 | IPR002182 |  | IPR001611 |  |  |
| FgenesH.VV78X006360.14_4 | RGA160 | 160 | - | white | NBS | 13 | 60,1 | -1 |  | IPR002182 |  |  |  |  |
| FgenesH.VV78X157915.4_1 | RGA161 | 161 | 3 | yellow | CC-NBS-LRR | 13 | 62,9 | 1 | IPR000767 | IPR002182 |  | IPR001611 |  | IPR003591 |
| FgenesH.VV78X063659.12_1 | RGA162 | 162 | 4 | cyan | NBS-LRR | 13 | 62,9 | 1 | IPR000767 | IPR002182 |  | IPR001611 |  |  |
| FgenesH.VV78X028186.17_1 | RGA163 | 163 | 4 | cyan | NBS-LRR | 13 | 63,0 | -1 | IPR000767 | IPR002182 |  | IPR001611 |  |  |
| Glimmer1.VV78X030048.70_4 | RGA164 | 164 | 4 | cyan | NBS-LRR | 13 | 63,1 | 1 | IPR000767 | IPR002182 |  | IPR001611 |  |  |
| FgenesH.VV78X102000.7_3 | RGA165 | 165 | - | white | NBS | 13 | 63,2 | 1 | IPR000767 | IPR002182 |  |  |  |  |
| FgenesH.VV78X065262.7_1 | RGA166 | 166 | 4 | cyan | NBS-LRR | 13 | 63,4 | 1 |  | IPR002182 |  | IPR001611 |  |  |
| FgenesH.VV78X122207.5_1 | RGA167 | 167 | 4 | cyan | NBS-LRR | 13 | 63,7 | 1 | IPR000767 | IPR002182 |  | IPR001611 |  | IPR003591 |
| FgenesH.VV78X169339.25_1 | RGA168 | 168 | 3 | yellow | CC-NBS-LRR | 13 | 63,8 | 1 | IPR000767 | IPR002182 |  | IPR001611 |  | IPR003591 |
| FgenesH.VV78X169339.25_3 | RGA169 | 169 | 4 | cyan | NBS-LRR | 13 | 63,8 | 1 | IPR000767 | IPR002182 |  | IPR001611 |  |  |
| Glimmer1.VV78X101622.7_2 | RGA170 | 170 | 4 | cyan | NBS-LRR | 13 | 64,3 | 1 | IPR000767 | IPR002182 |  | IPR001611 |  |  |
| FgenesH.VV79X001828.2_1 | RGA171 | 171 | 4 | cyan | NBS-LRR | 13 | 64,6 | 1 | IPR000767 | IPR002182 |  | IPR001611 |  |  |
| FgenesH.VV78X109122.3_1 | RGA172 | 172 | 4 | cyan | NBS-LRR | 13 | 64,8 | -1 | IPR000767 | IPR002182 |  | IPR001611 |  |  |
| FgenesH.VV78X260050.2_1 | RGA173 | 173 | 3 | yellow | CC-NBS-LRR | 13 | 64,8 | 1 | IPR000767 | IPR002182 |  | IPR001611 |  |  |
| Sim4.aln-TCVV015423 | RGA174 | 174 | - | white | NBS | 13 | 64,9 | 1 |  | IPR002182 |  |  |  |  |
| Glimmer1.VV78X260050.2_5 | RGA175 | 175 | 4 | cyan | NBS-LRR | 13 | 64,9 | 1 | IPR000767 | IPR002182 |  | IPR001611 |  | IPR003591 |
| Glimmer1.VV78X219975.3_2 | RGA176 | 176 | - | white | NBS | 13 | 64,9 | 1 | IPR000767 | IPR002182 |  |  |  |  |
| FgenesH.VV78X171202.65_1 | RGA177 | 177 | 3 | yellow | CC-NBS-LRR | 13 | 65,3 | -1 | IPR000767 | IPR002182 |  | IPR001611 |  |  |
| FgenesH.VV78X180769.5_2 | RGA178 | 178 | 4 | cyan | NBS-LRR | 13 | 65,4 | -1 | IPR000767 | IPR002182 |  | IPR001611 |  | IPR003591 |
| FgenesH.VV78X220144.38_2 | RGA179 | 179 | 3 | yellow | CC-NBS-LRR | 13 | 65,5 | 1 | IPR000767 | IPR002182 |  | IPR001611 |  | IPR003591 |
| FgenesH.VV78X014944.5_1 | RGA180 | 180 | 3 | yellow | CC-NBS-LRR | 13 | 65,7 | -1 | IPR000767 | IPR002182 |  | IPR001611 |  |  |
| FgenesH.VV78X221967.8_3 | RGA181 | 181 | - | white | NBS | 13 | 66,0 | -1 |  | IPR002182 |  |  |  |  |
| FgenesH.VV78X125776.5_1 | RGA182 | 182 | 4 | cyan | CC-NBS-LRR | 13 | 66,1 | -1 | IPR000767 | IPR002182 |  | IPR001611 |  |  |
| FgenesH.VV78X027885.8_3 | RGA183 | 183 | 4 | cyan | NBS-LRR | 13 | 67,0 | 1 | IPR000767 | IPR002182 |  | IPR001611 |  |  |
| FgenesH.VV78X076653.8_1 | RGA184 | 184 | 4 | cyan | NBS-LRR | 13 | 67,3 | 1 | IPR000767 | IPR002182 |  | IPR001611 |  |  |
| Glimmer1.VV78X220013.6_2 | RGA185 | 185 | 4 | cyan | NBS-LRR | 13 | 67,8 | 1 |  | IPR002182 |  | IPR001611 |  |  |
| FgenesH.VV78X204279.6_1 | RGA186 | 186 | 4 | cyan | NBS-LRR | 13 | 68,2 | 1 | IPR000767 | IPR002182 |  | IPR001611 |  |  |
| Sim4.aln-TCVV008594 | RGA187 | 187 | - | white | NBS | 13 | 68,2 | 1 |  | IPR002182 |  |  |  |  |
| FgenesH.VV78X046949.18_1 | RGA188 | 188 | 4 | cyan | NBS-LRR | 13 | 70,4 | -1 |  | IPR002182 |  | IPR001611 |  |  |
| FgenesH.VV78X137751.6_1 | RGA189 | 189 | - | white | NBS | 13 | 70,4 | 1 |  | IPR002182 |  |  |  |  |
| FgenesH.VV78X137751.6_2 | RGA190 | 190 | 4 | cyan | NBS-LRR | 13 | 70,4 | 1 | IPR000767 | IPR002182 |  | IPR001611 |  |  |
| FgenesH.VV78X162720.7_1 | RGA191 | 191 | 4 | cyan | CC-NBS-LRR | 13 | 70,5 | 1 | IPR000767 | IPR002182 |  | IPR001611 |  |  |
| FgenesH.VV78X162720.7_2 | RGA192 | 192 | 4 | cyan | NBS-LRR | 13 | 70,5 | 1 | IPR000767 | IPR002182 |  | IPR001611 |  |  |
| FgenesH.VV78X170959.11_1 | RGA193 | 193 | 4 | cyan | NBS-LRR | 13 | 70,5 | 1 | IPR000767 | IPR002182 |  | IPR001611 |  |  |
| FgenesH.VV78X278970.4_1 | RGA194 | 194 | 4 | cyan | NBS-LRR | 13 | 70,8 | -1 | IPR000767 | IPR002182 |  | IPR001611 |  |  |
| FgenesH.VV78X194068.4_1 | RGA195 | 195 | 4 | cyan | NBS-LRR | 13 | 70,9 | -1 | IPR000767 | IPR002182 |  | IPR001611 |  |  |
| FgenesH.VV78X271543.4_3 | RGA196 | 196 | 4 | cyan | NBS-LRR | 13 | 70,9 | 1 | IPR000767 | IPR002182 |  | IPR001611 |  |  |
| FgenesH.VV78X272840.9_2 | RGA197 | 197 | - | white | NBS | 14 | 16,5 | 1 | IPR000767 | IPR002182 |  |  |  |  |
| FgenesH.VV78X237456.9_2 | RGA198 | 198 | 5 | red | NBS-LRR | 14 | 16,8 | 1 | IPR000767 | IPR002182 |  | IPR001611 |  |  |
| FgenesH.VV78X232089.12_2 | RGA199 | 199 | 5 | red | NBS-LRR | 14 | 16,9 | 1 | IPR000767 | IPR002182 |  | IPR001611 |  |  |
| FgenesH.VV78X227300.4_1 | RGA200 | 200 | 5 | red | NBS-LRR | 14 | 17,1 | -1 | IPR000767 | IPR002182 |  | IPR001611 |  |  |
| FgenesH.VV78X178456.3_4 | RGA201 | 201 | 5 | red | NBS-LRR | 14 | 17,3 | 1 | IPR000767 | IPR002182 |  | IPR001611 |  |  |
| FgenesH.VV78X195966.6_1 | RGA202 | 202 | - | white | NBS | 14 | 17,6 | 1 | IPR000767 | IPR002182 |  |  |  |  |
| FgenesH.VV78X252551.18_2 | RGA203 | 203 | 4 | cyan | CC-NBS-LRR | 15 | 1,8 | -1 | IPR000767 | IPR002182 |  | IPR001611 |  |  |
| FgenesH.VV78X124158.82_5 | RGA204 | 204 | 5 | red | CC-NBS-LRR | 15 | 37,3 | 1 | IPR000767 | IPR002182 |  |  |  |  |
| Glimmer1.VV78X035180.3_2 | RGA205 | 205 | - | white | NBS | 15 | 37,5 | 1 |  | IPR002182 |  |  |  |  |
| Sim4.aln-TCVV017073 | RGA206 | 206 | - | white | NBS | 15 | 38,2 | 1 | IPR000767 | IPR002182 |  |  |  |  |
| Glimmer1.VV78X088068.4_1 | RGA207 | 207 | - | white | NBS | 15 | 38,3 | 1 | IPR000767 | IPR002182 |  |  |  |  |
| FgenesH.VV78X063016.8_2 | RGA208 | 208 | 5 | red | CC-NBS-LRR | 15 | 39,0 | -1 | IPR000767 | IPR002182 |  | IPR001611 |  |  |
| FgenesH.VV78X172014.5_1 | RGA209 | 209 | - | white | NBS | 15 | 39,2 | 1 |  | IPR002182 |  |  |  |  |
| FgenesH.VV78X275934.2_1 | RGA210 | 210 | 4 | cyan | NBS-LRR | 16 | 26,9 | 1 | IPR000767 | IPR002182 |  | IPR001611 |  |  |
| FgenesH.VV78X147582.13_1 | RGA211 | 211 | 3 | yellow | CC-NBS-LRR | 16 | 27,6 | -1 | IPR000767 | IPR002182 |  | IPR001611 |  |  |
| FgenesH.VV78X191068.8_4 | RGA212 | 212 | - | white | NBS | 16 | 32,8 | -1 |  | IPR002182 |  |  |  |  |
| FgenesH.VV78X077165.10_4 | RGA213 | 213 | - | white | NBS | 18 | 6,9 | -1 |  | IPR002182 |  |  |  |  |
| FgenesH.VV78X085010.8_3 | RGA214 | 214 | 4 | cyan | NBS-LRR | 18 | 15,6 | -1 | IPR000767 | IPR002182 |  | IPR001611 |  |  |
| FgenesH.VV78X265499.17_1 | RGA215 | 215 | 5 | red | NBS-LRR | 18 | 17,1 | -1 | IPR000767 | IPR002182 |  | IPR001611 |  |  |
| FgenesH.VV78X157482.9_1 | RGA216 | 216 | - | white | NBS | 18 | 27,7 | -1 | IPR000767 | IPR002182 |  |  |  |  |
| Glimmer1.VV78X233392.5_4 | RGA217 | 217 | - | white | NBS | 18 | 28,2 | -1 | IPR000767 | IPR002182 |  |  |  |  |
| FgenesH.VV78X124233.5_2 | RGA218 | 218 | 1 | blue | TIR-NBS-LRR | 18 | 53,8 | 1 | IPR000767 | IPR002182 | IPR000157 | IPR001611 | IPR011713 | IPR003591 |
| Sim4.aln-A06017346 | RGA219 | 219 | - | black | TIR-NBS | 18 | 53,8 | 1 |  | IPR002182 | IPR000157 |  |  |  |
| FgenesH.VV78X043953.13_2 | RGA220 | 220 | - | black | TIR-NBS | 18 | 56,3 | -1 |  | IPR002182 | IPR000157 |  |  |  |
| FgenesH.VV78X163078.4_2 | RGA221 | 221 | 1 | blue | TIR-NBS-LRR | 18 | 57,8 | -1 | IPR000767 | IPR002182 | IPR000157 | IPR001611 | IPR011713 |  |
| FgenesH.VV78X052352.4_2 | RGA222 | 222 | 1 | blue | TIR-NBS-LRR | 18 | 62,2 | -1 | IPR000767 | IPR002182 | IPR000157 | IPR001611 | IPR011713 |  |
| FgenesH.VV78X048927.11_1 | RGA223 | 223 | 1 | blue | TIR-NBS-LRR | 18 | 63,3 | 1 | IPR000767 | IPR002182 | IPR000157 | IPR001611 | IPR011713 | IPR003591 |
| FgenesH.VV78X174559.7_5 | RGA224 | 224 | 1 | blue | TIR-NBS-LRR | 18 | 63,8 | -1 | IPR000767 | IPR002182 | IPR000157 | IPR001611 | IPR011713 |  |
| FgenesH.VV78X022531.8_1 | RGA225 | 225 | 1 | blue | TIR-NBS-LRR | 18 | 63,8 | 1 | IPR000767 | IPR002182 | IPR000157 | IPR001611 | IPR011713 |  |
| FgenesH.VV78X130162.8_1 | RGA226 | 226 | 1 | blue | TIR-NBS-LRR | 18 | 64,1 | 1 | IPR000767 | IPR002182 | IPR000157 | IPR001611 |  |  |
| Glimmer1.VV78X194983.10_2 | RGA227 | 227 | - | white | NBS | 18 | 64,2 | -1 | IPR000767 | IPR002182 |  |  |  |  |
| FgenesH.VV78X015370.3_2 | RGA228 | 228 | 4 | cyan | NBS-LRR | 18 | 64,3 | 1 | IPR000767 | IPR002182 |  | IPR001611 |  |  |
| FgenesH.VV78X057586.2_1 | RGA229 | 229 | 4 | cyan | NBS-LRR | 18 | 64,3 | 1 | IPR000767 | IPR002182 |  | IPR001611 |  |  |
| Glimmer1.VV78X196130.36_4 | RGA230 | 230 | - | white | NBS | 18 | 64,4 | 1 | IPR000767 | IPR002182 |  |  |  |  |
| FgenesH.VV78X003543.10_1 | RGA231 | 231 | 1 | blue | TIR-NBS-LRR | 18 | 64,6 | -1 | IPR000767 | IPR002182 | IPR000157 | IPR001611 | IPR011713 | IPR003591 |
| FgenesH.VV78X072046.2_2 | RGA232 | 232 | 1 | blue | TIR-NBS-LRR | 18 | 64,7 | 1 | IPR000767 | IPR002182 | IPR000157 | IPR001611 | IPR011713 |  |
| FgenesH.VV78X193388.6_2 | RGA233 | 233 | 1 | blue | TIR-NBS-LRR | 18 | 64,8 | -1 | IPR000767 | IPR002182 | IPR000157 | IPR001611 | IPR011713 | IPR003591 |
| FgenesH.VV78X073612.6_1 | RGA234 | 234 | 1 | blue | TIR-NBS-LRR | 18 | 64,8 | 1 | IPR000767 | IPR002182 | IPR000157 | IPR001611 |  | IPR003591 |
| FgenesH.VV78X068727.3_2 | RGA235 | 235 | 1 | blue | TIR-NBS-LRR | 18 | 64,9 | 1 | IPR000767 | IPR002182 | IPR000157 | IPR001611 | IPR011713 | IPR003591 |
| FgenesH.VV78X044248.8_2 | RGA236 | 236 | 1 | blue | TIR-NBS-LRR | 18 | 64,9 | 1 | IPR000767 | IPR002182 | IPR000157 | IPR001611 | IPR011713 |  |
| FgenesH.VV78X216007.22_2 | RGA237 | 237 | 1 | blue | TIR-NBS-LRR | 18 | 65,0 | -1 | IPR000767 | IPR002182 | IPR000157 | IPR001611 | IPR011713 |  |
| FgenesH.VV78X040047.2_1 | RGA238 | 238 | 1 | blue | TIR-NBS-LRR | 18 | 65,0 | 1 | IPR000767 | IPR002182 | IPR000157 | IPR001611 | IPR011713 | IPR003591 |
| FgenesH.VV78X069950.2_1 | RGA239 | 239 | 1 | blue | TIR-NBS-LRR | 18 | 65,1 | -1 | IPR000767 | IPR002182 | IPR000157 | IPR001611 | IPR011713 |  |
| FgenesH.VV78X205894.9_1 | RGA240 | 240 | 1 | blue | TIR-NBS-LRR | 18 | 66,6 | -1 | IPR000767 | IPR002182 | IPR000157 | IPR001611 | IPR011713 | IPR003591 |
| FgenesH.VV78X155079.8_2 | RGA241 | 241 | 1 | blue | TIR-NBS-LRR | 18 | 66,7 | 1 | IPR000767 | IPR002182 | IPR000157 | IPR001611 | IPR011713 |  |
| Glimmer1.VV78X115602.14_2 | RGA242 | 242 | - | white | NBS | 18 | 67,1 | 1 |  | IPR002182 |  |  |  |  |
| FgenesH.VV78X115602.14_5 | RGA243 | 243 | 1 | blue | TIR-NBS-LRR | 18 | 67,2 | 1 | IPR000767 | IPR002182 | IPR000157 | IPR001611 |  |  |
| FgenesH.VV78X187008.9_1 | RGA244 | 244 | - | white | NBS | 18 | 72,8 | -1 | IPR000767 | IPR002182 |  |  |  |  |
| FgenesH.VV78X187008.9_3 | RGA245 | 245 | 5 | red | NBS-LRR | 18 | 72,9 | -1 |  | IPR002182 |  | IPR001611 |  |  |
| Glimmer1.VV78X102999.17_5 | RGA246 | 246 | 5 | red | NBS-LRR | 19 | 1,1 | 1 | IPR000767 | IPR002182 |  | IPR001611 |  | IPR003591 |
| FgenesH.VV78X024905.29_2 | RGA247 | 247 | 5 | red | NBS-LRR | 19 | 1,8 | -1 | IPR000767 | IPR002182 |  | IPR001611 |  | IPR003591 |
| FgenesH.VV78X025601.5_1 | RGA248 | 248 | 1 | blue | TIR-NBS-LRR | 19 | 5,5 | -1 | IPR000767 | IPR002182 | IPR000157 | IPR001611 | IPR011713 | IPR003591 |
| FgenesH.VV78X108201.9_1 | RGA249 | 249 | - | white | NBS | 19 | 6,0 | -1 |  | IPR002182 |  |  |  |  |
| FgenesH.VV78X166709.2_2 | RGA250 | 250 | - | white | NBS | 19 | 17,4 | 1 |  | IPR002182 |  |  |  |  |
| FgenesH.VV78X182902.14_2 | RGA251 | 251 | - | white | NBS | 19 | 17,5 | 1 |  | IPR002182 |  |  |  |  |
| FgenesH.VV79X004527.4_1 | RGA252 | 252 | - | white | NBS | 19 | 17,6 | -1 | IPR000767 | IPR002182 |  |  |  |  |
| FgenesH.VV78X063746.5_1 | RGA253 | 253 | 1 | blue | TIR-NBS-LRR | 19 | 22,3 | 1 | IPR000767 | IPR002182 | IPR000157 | IPR001611 | IPR011713 |  |
| FgenesH.VV78X211090.8_5 | RGA254 | 254 | - | white | NBS | 19 | 27,8 | 1 |  | IPR002182 |  |  |  |  |
| FgenesH.VV78X254837.15_1 | RGA255 | 255 | - | white | NBS | 19 | 44,6 | -1 | IPR000767 | IPR002182 |  |  |  |  |
| FgenesH.VV78X147990.12_1 | RGA256 | 256 | 5 | red | CC-NBS-LRR | 19 | 57,3 | -1 | IPR000767 | IPR002182 |  | IPR001611 |  |  |
| FgenesH.VV78X220407.9_11 | RGA257 | 257 | 5 | red | CC-NBS-LRR | 19 | 57,7 | 1 | IPR000767 | IPR002182 |  | IPR001611 |  |  |
| FgenesH.VV78X244202.4_1 | RGA258 | 258 | 5 | red | CC-NBS-LRR | 19 | 57,8 | -1 | IPR000767 | IPR002182 |  | IPR001611 |  |  |
| FgenesH.VV78X114707.11_1 | RGA259 | 259 | 5 | red | CC-NBS-LRR | 19 | 57,9 | 1 | IPR000767 | IPR002182 |  | IPR001611 |  |  |
| FgenesH.VV78X056978.5_1 | RGA260 | 260 | - | white | NBS | 19 | 58,2 | 1 | IPR000767 | IPR002182 |  |  |  |  |
| FgenesH.VV78X240337.11_1 | RGA261 | 261 | 2 | green | CC-NBS-LRR | 19 | 58,4 | 1 |  | IPR002182 |  | IPR001611 |  |  |
| FgenesH.VV78X061725.29_2 | RGA262 | 262 | - | white | NBS | 19 | 60,1 | 1 |  | IPR002182 |  |  |  |  |
| FgenesH.VV78X083420.6_1 | RGA263 | 263 | 4 | cyan | CC-NBS-LRR | 19 | 60,5 | 1 | IPR000767 | IPR002182 |  | IPR001611 |  |  |
| FgenesH.VV78X245461.4_1 | RGA264 | 264 | 4 | cyan | NBS-LRR | 19 | 60,5 | 1 | IPR000767 | IPR002182 |  | IPR001611 |  |  |
| FgenesH.VV78X077369.15_1 | RGA265 | 265 | 4 | cyan | NBS-LRR | 19 | 61,1 | 1 | IPR000767 | IPR002182 |  | IPR001611 |  | IPR003591 |
| FgenesH.VV78X082108.2_2 | RGA266 | 266 | 4 | cyan | NBS-LRR | 19 | 68,3 | -1 | IPR000767 | IPR002182 |  | IPR001611 |  | IPR003591 |
| FgenesH.VV78X115004.17_1 | RGA267 | 267 | - | white | NBS | n.d. | n.d. | n.d. |  | IPR002182 |  |  |  |  |
| Sim4.aln-TCVV013873 | RGA268 | 268 | - | white | NBS | n.d. | n.d. | n.d. | IPR000767 | IPR002182 |  |  |  |  |
| FgenesH.VV78X075301.12_1 | RGA269 | 269 | 4 | cyan | NBS-LRR | n.d. | n.d. | n.d. | IPR000767 | IPR002182 |  | IPR001611 |  |  |
| Sim4.aln-TCVV019207 | RGA270 | 270 | - | white | NBS | n.d. | n.d. | n.d. | IPR000767 | IPR002182 |  |  |  |  |
| Sim4.aln-TCVV023016 | RGA271 | 271 | - | white | NBS | n.d. | n.d. | n.d. |  | IPR002182 |  |  |  |  |
| Glimmer1.VV78X240488.6_4 | RGA272 | 272 | 5 | red | NBS-LRR | n.d. | n.d. | n.d. |  | IPR002182 |  | IPR001611 |  |  |
| FgenesH.VV78X108101.17_1 | RGA273 | 273 | 4 | cyan | NBS-LRR | n.d. | n.d. | n.d. | IPR000767 | IPR002182 |  | IPR001611 |  |  |
| Sim4.aln-TCVV021635 | RGA274 | 274 | - | white | NBS | n.d. | n.d. | n.d. | IPR000767 | IPR002182 |  |  |  |  |
| FgenesH.VV78X030048.70_3 | RGA275 | 275 | 4 | cyan | NBS-LRR | n.d. | n.d. | n.d. | IPR000767 | IPR002182 |  | IPR001611 |  |  |
| Glimmer1.VV78X122207.5_1 | RGA276 | 276 | 1 | blue | CC-NBS-LRR | n.d. | n.d. | n.d. | IPR000767 | IPR002182 |  | IPR001611 |  | IPR003591 |
| Glimmer1.VV78X235774.23_2 | RGA277 | 277 | - | white | NBS | n.d. | n.d. | n.d. | IPR000767 | IPR002182 |  |  |  |  |
| Sim4.aln-TCVV013660 | RGA278 | 278 | - | white | NBS | n.d. | n.d. | n.d. |  | IPR002182 |  |  |  |  |
| FgenesH.VV78X202688.2_2 | RGA279 | 279 | 5 | red | NBS-LRR | n.d. | n.d. | n.d. | IPR000767 | IPR002182 |  | IPR001611 |  |  |
| FgenesH.VV79X010287.4_1 | RGA280 | 280 | 5 | red | CC-NBS-LRR | n.d. | n.d. | n.d. | IPR000767 | IPR002182 |  | IPR001611 |  | IPR003591 |
| FgenesH.VV78X009711.6_3 | RGA281 | 281 | 5 | red | CC-NBS-LRR | n.d. | n.d. | n.d. | IPR000767 | IPR002182 |  | IPR001611 |  |  |
| FgenesH.VV78X115183.9_3 | RGA282 | 282 | 5 | red | CC-NBS-LRR | n.d. | n.d. | n.d. | IPR000767 | IPR002182 |  | IPR001611 |  |  |
| FgenesH.VV78X083051.7_2 | RGA283 | 283 | 5 | red | CC-NBS-LRR | n.d. | n.d. | n.d. | IPR000767 | IPR002182 |  | IPR001611 |  |  |
| FgenesH.VV79X005129.3_12 | RGA284 | 284 | 5 | red | CC-NBS-LRR | n.d. | n.d. | n.d. | IPR000767 | IPR002182 |  | IPR001611 |  |  |
| FgenesH.VV78X155423.10_1 | RGA285 | 285 | 5 | red | NBS-LRR | n.d. | n.d. | n.d. | IPR000767 | IPR002182 |  | IPR001611 |  |  |
| FgenesH.VV78X116426.6_5 | RGA286 | 286 | - | white | NBS | n.d. | n.d. | n.d. | IPR000767 | IPR002182 |  |  |  |  |
| FgenesH.VV78X220284.5_1 | RGA287 | 287 | 4 | cyan | CC-NBS-LRR | n.d. | n.d. | n.d. | IPR000767 | IPR002182 |  | IPR001611 |  |  |
| FgenesH.VV78X039425.4_1 | RGA288 | 288 | 4 | cyan | CC-NBS-LRR | n.d. | n.d. | n.d. | IPR000767 | IPR002182 |  | IPR001611 |  |  |
| FgenesH.VV78X135422.8_1 | RGA289 | 289 | 2 | green | CC-NBS-LRR | n.d. | n.d. | n.d. | IPR000767 | IPR002182 |  | IPR001611 |  |  |
| FgenesH.VV78X261596.20_1 | RGA290 | 290 | 4 | cyan | CC-NBS-LRR | n.d. | n.d. | n.d. | IPR000767 | IPR002182 |  |  |  |  |
| FgenesH.VV78X174581.14_1 | RGA291 | 291 | 4 | cyan | NBS-LRR | n.d. | n.d. | n.d. | IPR000767 | IPR002182 |  | IPR001611 |  |  |
| Glimmer1.VV78X224244.8_1 | RGA292 | 292 | - | black | TIR-NBS | n.d. | n.d. | n.d. | IPR000767 | IPR002182 | IPR000157 |  |  |  |
| FgenesH.VV78X106678.4_2 | RGA293 | 293 | - | black | TIR-NBS | n.d. | n.d. | n.d. | IPR000767 | IPR002182 | IPR000157 |  |  |  |
| Glimmer1.VV78X244192.9_3 | RGA294 | 294 | 4 | cyan | NBS-LRR | n.d. | n.d. | n.d. | IPR000767 | IPR002182 |  | IPR001611 |  |  |
| FgenesH.VV78X180694.3_1 | RGA295 | 295 | 4 | cyan | NBS-LRR | n.d. | n.d. | n.d. | IPR000767 | IPR002182 |  | IPR001611 |  | IPR003591 |
| FgenesH.VV78X039425.4_2 | RGA296 | 296 | 4 | cyan | NBS-LRR | n.d. | n.d. | n.d. | IPR000767 | IPR002182 |  | IPR001611 |  |  |
| Glimmer1.VV78X232181.3_1 | RGA297 | 297 | - | white | NBS | n.d. | n.d. | n.d. | IPR000767 | IPR002182 |  |  |  |  |
| Glimmer1.VV78X150163.4_2 | RGA298 | 298 | - | white | NBS | n.d. | n.d. | n.d. | IPR000767 | IPR002182 |  |  |  |  |
| FgenesH.VV78X021896.11_1 | RGA299 | 299 | 3 | yellow | CC-NBS-LRR | n.d. | n.d. | n.d. | IPR000767 | IPR002182 |  | IPR001611 |  | IPR003591 |
| FgenesH.VV79X004014.3_6 | RGA300 | 300 | 1 | blue | TIR-NBS-LRR | n.d. | n.d. | n.d. | IPR000767 | IPR002182 | IPR000157 | IPR001611 | IPR011713 | IPR003591 |
| FgenesH.VV78X079954.20_3 | RGA301 | 301 | 1 | blue | TIR-NBS-LRR | n.d. | n.d. | n.d. | IPR000767 | IPR002182 | IPR000157 | IPR001611 |  |  |
| FgenesH.VV78X239003.6_1 | RGA302 | 302 | 5 | red | NBS-LRR | n.d. | n.d. | n.d. | IPR000767 | IPR002182 |  | IPR001611 |  |  |
| FgenesH.VV78X272865.9_2 | RGA303 | 303 | 5 | red | NBS-LRR | n.d. | n.d. | n.d. | IPR000767 | IPR002182 |  | IPR001611 |  | IPR003591 |
| FgenesH.VV78X257589.7_1 | RGA304 | 304 | 5 | red | NBS-LRR | n.d. | n.d. | n.d. | IPR000767 | IPR002182 |  | IPR001611 |  |  |
| FgenesH.VV78X251334.16_4 | RGA305 | 305 | 5 | red | NBS-LRR | n.d. | n.d. | n.d. | IPR000767 | IPR002182 |  |  |  |  |
| FgenesH.VV78X028454.18_1 | RGA306 | 306 | 5 | red | NBS-LRR | n.d. | n.d. | n.d. | IPR000767 | IPR002182 |  | IPR001611 |  |  |
| FgenesH.VV78X214367.23_2 | RGA307 | 307 | 1 | blue | TIR-NBS-LRR | n.d. | n.d. | n.d. | IPR000767 | IPR002182 | IPR000157 | IPR001611 | IPR011713 |  |
| FgenesH.VV78X266640.9_4 | RGA308 | 308 | 1 | blue | TIR-NBS-LRR | n.d. | n.d. | n.d. | IPR000767 | IPR002182 | IPR000157 | IPR001611 | IPR011713 | IPR003591 |
| FgenesH.VV78X073201.6_2 | RGA309 | 309 | 1 | blue | TIR-NBS-LRR | n.d. | n.d. | n.d. | IPR000767 | IPR002182 | IPR000157 | IPR001611 | IPR011713 |  |
| FgenesH.VV78X261731.23_1 | RGA310 | 310 | 1 | blue | TIR-NBS-LRR | n.d. | n.d. | n.d. | IPR000767 | IPR002182 | IPR000157 | IPR001611 |  | IPR003591 |
| Glimmer1.VV78X126751.10_2 | RGA311 | 311 | 1 | blue | CC-NBS-LRR | n.d. | n.d. | n.d. |  | IPR002182 |  | IPR001611 |  | IPR003591 |
| FgenesH.VV78X093536.11_1 | RGA312 | 312 | 5 | red | CC-NBS-LRR | n.d. | n.d. | n.d. | IPR000767 | IPR002182 |  | IPR001611 |  | IPR003591 |
| FgenesH.VV78X171068.13_1 | RGA313 | 313 | 5 | red | CC-NBS-LRR | n.d. | n.d. | n.d. | IPR000767 | IPR002182 |  | IPR001611 |  |  |
| Sim4.aln-TCVV021391 | RGA314 | 314 | - | white | NBS | n.d. | n.d. | n.d. |  | IPR002182 |  |  |  |  |
| Sim4.aln-TCVV017417 | RGA315 | 315 | - | white | NBS | n.d. | n.d. | n.d. |  | IPR002182 |  |  |  |  |
| Sim4.aln-TCVV012842 | RGA316 | 316 | - | white | NBS | n.d. | n.d. | n.d. |  | IPR002182 |  |  |  |  |
| Sim4.aln-TCVV008876 | RGA317 | 317 | - | white | NBS | n.d. | n.d. | n.d. |  | IPR002182 |  |  |  |  |
| Sim4.aln-A06013465 | RGA318 | 318 | - | white | NBS | n.d. | n.d. | n.d. |  | IPR002182 |  |  |  |  |
| Sim4.aln-A06008652 | RGA319 | 319 | - | white | NBS | n.d. | n.d. | n.d. |  | IPR002182 |  |  |  |  |
| Glimmer1.VV78X252604.5_7 | RGA320 | 320 | 5 | red | NBS-LRR | n.d. | n.d. | n.d. | IPR000767 | IPR002182 |  | IPR001611 |  | IPR003591 |
| Glimmer1.VV78X024016.13_1 | RGA321 | 321 | 5 | red | NBS-LRR | n.d. | n.d. | n.d. | IPR000767 | IPR002182 |  | IPR001611 |  |  |
| Glimmer1.VV78X005257.8_4 | RGA322 | 322 | 4 | cyan | NBS-LRR | n.d. | n.d. | n.d. | IPR000767 | IPR002182 |  | IPR001611 |  |  |
| FgenesH.VV78X269027.21_8 | RGA323 | 323 | - | white | NBS | n.d. | n.d. | n.d. |  | IPR002182 |  |  |  |  |
| FgenesH.VV78X264730.14_1 | RGA324 | 324 | 5 | red | NBS-LRR | n.d. | n.d. | n.d. | IPR000767 | IPR002182 |  | IPR001611 | IPR011713 | IPR003591 |
| FgenesH.VV78X236675.4_1 | RGA325 | 325 | - | white | NBS | n.d. | n.d. | n.d. |  | IPR002182 |  |  |  |  |
| FgenesH.VV78X230605.16_1 | RGA326 | 326 | - | white | NBS | n.d. | n.d. | n.d. | IPR000767 | IPR002182 |  |  |  |  |
| FgenesH.VV78X218576.14_1 | RGA327 | 327 | - | white | NBS | n.d. | n.d. | n.d. |  | IPR002182 |  |  |  |  |
| FgenesH.VV78X214367.23_1 | RGA328 | 328 | 5 | red | NBS-LRR | n.d. | n.d. | n.d. | IPR000767 | IPR002182 |  | IPR001611 |  |  |
| FgenesH.VV78X178802.5_4 | RGA329 | 329 | - | white | NBS | n.d. | n.d. | n.d. | IPR000767 | IPR002182 |  |  |  |  |
| FgenesH.VV78X129577.6_7 | RGA330 | 330 | - | white | NBS | n.d. | n.d. | n.d. |  | IPR002182 |  |  |  |  |
| FgenesH.VV78X125821.6_2 | RGA331 | 331 | - | white | NBS | n.d. | n.d. | n.d. | IPR000767 | IPR002182 |  |  |  |  |
| FgenesH.VV78X123043.13_2 | RGA332 | 332 | - | white | NBS | n.d. | n.d. | n.d. | IPR000767 | IPR002182 |  |  |  |  |
| FgenesH.VV78X102680.7_6 | RGA333 | 333 | 4 | cyan | NBS-LRR | n.d. | n.d. | n.d. | IPR000767 | IPR002182 |  | IPR001611 |  |  |
| FgenesH.VV78X091071.15_3 | RGA334 | 334 | - | white | NBS | n.d. | n.d. | n.d. |  | IPR002182 |  |  |  |  |
| FgenesH.VV78X079954.20_5 | RGA335 | 335 | - | white | NBS | n.d. | n.d. | n.d. | IPR000767 | IPR002182 |  |  |  |  |
| FgenesH.VV78X048178.14_2 | RGA336 | 336 | - | white | NBS | n.d. | n.d. | n.d. |  | IPR002182 |  |  |  |  |
| FgenesH.VV78X033257.17_2 | RGA337 | 337 | 5 | red | NBS-LRR | n.d. | n.d. | n.d. |  | IPR002182 |  | IPR001611 |  |  |
| FgenesH.VV78X029516.3_1 | RGA338 | 338 | - | white | NBS | n.d. | n.d. | n.d. |  | IPR002182 |  |  |  |  |
| FgenesH.VV78X021187.4_1 | RGA339 | 339 | - | white | NBS | n.d. | n.d. | n.d. |  | IPR002182 |  |  |  |  |
| FgenesH.VV78X011475.16_6 | RGA340 | 340 | - | white | NBS | n.d. | n.d. | n.d. | IPR000767 | IPR002182 |  |  |  |  |
| Sim4.aln-TCVV021439 | RGA341 | 341 | - | white | NBS | n.d. | n.d. | n.d. |  | IPR002182 |  |  |  |  |

**Resistance-related genes**

| **Vitis_Gene_ID** | **R-gene_ID** | **A.t. Homolog** | **E-Value** | **Abbreviation** | **LG** | **Mb** | **Direction** | **Description** |
| --- | --- | --- | --- | --- | --- | --- | --- | --- |
| FgenesH.VV78X083692.14_1 | COI1a | AT2G39940.1 | 6E-81 | COI1 | 14 | 8,6 | 1 | coronatine insensitive 1 |
| Sim4.aln-TCVV004234 | COI1b | AT2G39940.1 | 1E-67 | COI1 | n.d. | n.d. | n.d. | coronatine insensitive 1 |
| FgenesH.VV78X094068.6_10 | COI1c | AT2G39940.1 | 2E-60 | COI1 | n.d. | n.d. | n.d. | coronatine insensitive 1 |
| Sim4.aln-TCVV002821 | COI1d | AT2G39940.1 | 5E-143 | COI1 | 13 | 12,2 | -1 | coronatine insensitive 1 |
| FgenesH.VV78X008535.11_1 | EDS1a | AT3G48090.1 | 2E-98 | EDS1 | 17 | 14,7 | -1 | enhanced disease susceptibility 1 |
| FgenesH.VV78X135063.5_1 | EDS1b | AT3G48090.1 | 3E-97 | EDS1 | 17 | 14,8 | 1 | enhanced disease susceptibility 1 |
| FgenesH.VV78X147478.6_1 | EIN2 | AT5G03280.1 | 0 | EIN2 | 8 | 43,7 | -1 | ethylene insensitive 2 |
| FgenesH.VV78X205831.4_5 | ETR1a | AT1G66340.1 | 0 | ETR1 | 7 | 15,4 | 1 | ethylene response 1 |
| FgenesH.VV78X132092.4_1 | ETR1b | AT1G66340.1 | 2E-141 | ETR1 | 5 | 18,5 | -1 | ethylene response 1 |
| FgenesH.VV78X207570.7_2 | ETR1c | AT1G66340.1 | 0 | ETR1 | 19 | 64,4 | 1 | ethylene response 1 |
| Sim4.aln-TCVV000467 | JAR1a | AT2G46370.1 | 0 | JAR1 | 15 | 39,8 | -1 | jasmonate resistant 1 |
| FgenesH.VV78X054233.12_1 | JAR1b | AT2G46370.1 | 1E-113 | JAR1 | 3 | 26,9 | -1 | jasmonate resistant 1 |
| FgenesH.VV78X004046.5_1 | JAR1c | AT2G46370.1 | 2E-111 | JAR1 | 7 | 55,1 | -1 | jasmonate resistant 1 |
| FgenesH.VV78X257495.4_1 | JAR1d | AT2G46370.1 | 5E-111 | JAR1 | 7 | 8,6 | -1 | jasmonate resistant 1 |
| Glimmer1.VV78X213600.7_4 | JAR1e | AT2G46370.1 | 4E-108 | JAR1 | n.d. | n.d. | n.d. | jasmonate resistant 1 |
| FgenesH.VV78X092673.12_2 | JAR1f | AT2G46370.1 | 3E-101 | JAR1 | 1 | 68,2 | -1 | jasmonate resistant 1 |
| Sim4.aln-TCVV022963 | JAR1g | AT2G46370.1 | 0 | JAR1 | n.d. | n.d. | n.d. | jasmonate resistant 1 |
| Sim4.aln-TCVV011761 | MLO1 | AT4G02600.1 | 2E-177 | MLO1 | 5 | 62,6 | -1 | barley mildew resistance locus homolog 1 |
| FgenesH.VV78X012793.13_1 | MLO2 | AT1G61560.1 | 3E-115 | MLO6 | 12 | 9,8 | 1 | barley mildew resistance locus homolog 6 |
| FgenesH.VV78X173560.18_1 | MLO3 | AT2G17480.1 | 3E-97 | MLO8 | 18 | 1,6 | 1 | barley mildew resistance locus homolog 8 |
| FgenesH.VV78X240627.13_1 | MLO4 | AT5G65970.1 | 7E-174 | MLO10 | 7 | 63,0 | 1 | barley mildew resistance locus homolog 10 |
| FgenesH.VV78X261497.8_1 | MLO5 | AT5G53760.1 | 2E-134 | MLO11 | 15 | 1,6 | -1 | barley mildew resistance locus homolog 11 |
| Twinscan1.VV78X230021.2_1 | MLO6 | AT2G39200.1 | 0 | MLO12 | n.d. | n.d. | n.d. | barley mildew resistance locus homolog 12 |
| FgenesH.VV78X208633.16_5 | MLO7 | AT4G24250.1 | 5E-88 | MLO13 | 5 | 62,6 | -1 | barley mildew resistance locus homolog 13 |
| FgenesH.VV79X002900.2_2 | MLO8 | AT2G44110.1 | 6E-137 | MLO15 | 7 | 41,6 | 1 | barley mildew resistance locus homolog 15 |
| Sim4.aln-TCVV003709 | MPK4a | AT4G01370.1 | 0 | MPK4 | 15 | 35,7 | -1 | MAP kinase 4 |
| FgenesH.VV79X002210.3_6 | MPK4b | AT4G01370.1 | 7E-170 | MPK4 | 6 | 11,2 | -1 | MAP kinase 4 |
| FgenesH.VV78X256675.17_2 | MPK4c | AT4G01370.1 | 3E-150 | MPK4 | n.d. | n.d. | n.d. | MAP kinase 4 |
| Sim4.aln-TCVV022715 | MPK4d | AT4G01370.1 | 1E-133 | MPK4 | 6 | 10,8 | -1 | MAP kinase 4 |
| Glimmer1.VV78X276383.3_2 | MPK4e | AT4G01370.1 | 8E-118 | MPK4 | 18 | 31,6 | -1 | MAP kinase 4 |
| FgenesH.VV78X150121.6_5 | MPK4f | AT4G01370.1 | 3E-113 | MPK4 | 4 | 51,5 | -1 | MAP kinase 4 |
| Sim4.aln-TCVV003025 | MPK4g | AT4G01370.1 | 0 | MPK4 | 2 | 0,6 | 1 | MAP kinase 4 |
| Sim4.aln-TCVV003860 | NDR1a | AT3G20600.1 | 6E-08 | NDR1 | n.d. | n.d. | n.d. | non race-specific disease resistance 1 |
| Sim4.aln-TCVV022133 | NDR1b | AT3G20600.1 | 1E-07 | NDR1 | 6 | 45,2 | 1 | non race-specific disease resistance 1 |
| Sim4.aln-TCVV001995 | NDR1c | AT3G20600.1 | 2E-05 | NDR1 | 8 | 52,8 | 1 | non race-specific disease resistance 1 |
| FgenesH.VV78X234451.2_5 | NDR1d | AT3G20600.1 | 2E-05 | NDR1 | 6 | 53,3 | 1 | non race-specific disease resistance 1 |
| FgenesH.VV78X170449.20_5 | NDR1e | AT3G20600.1 | 3E-13 | NDR1 | 10 | 22,2 | 1 | non race-specific disease resistance 1 |
| FgenesH.VV78X067529.9_1 | NPR1 | AT1G64280.1 | 2E-101 | NPR1 | 10 | 53,3 | 1 | nonexpresser of pr genes 1 |
| FgenesH.VV78X059103.7_2 | PAD4a | AT3G52430.1 | 2E-25 | PAD4 | 17 | 15,4 | 1 | phytoalexin-deficient 4 |
| FgenesH.VV78X029747.10_2 | PAD4b | AT3G52430.1 | 2E-106 | PAD4 | 7 | 63,5 | -1 | phytoalexin-deficient 4 |
| FgenesH.VV78X211230.5_4 | PDF1.2 | AT5G44420.1 | 3E-15 | PDF1.2 | 18 | 31,4 | 1 | plant defensin 1.2 |
| FgenesH.VV78X231296.5_1 | PDF2.1 | AT2G02120.1 | 3E-17 | PDF2.1 | 9 | 2,0 | -1 | plant defensin 2.1 |
| FgenesH.VV78X113969.14_2 | PEN1a | AT3G11820.1 | 7E-101 | PEN1 | 10 | 1,0 | -1 | penetration 1 |
| FgenesH.VV78X040508.9_6 | PEN1b | AT3G11820.1 | 4E-99 | PEN1 | 12 | 22,3 | 1 | penetration 1 |
| Sim4.aln-TCVV009391 | PEN1c | AT3G11820.1 | 1E-98 | PEN1 | 8 | 20,4 | 1 | penetration 1 |
| FgenesH.VV78X143603.3_1 | PEN1d | AT3G11820.1 | 1E-68 | PEN1 | 4 | 6,0 | -1 | penetration 1 |
| Glimmer1.VV78X017225.20_3 | PEN1e | AT3G11820.1 | 5E-108 | PEN1 | 8 | 20,4 | 1 | penetration 1 |
| Sim4.aln-TCVV003145 | PEN2a | AT2G44490.1 | 4E-110 | PEN2 | 17 | 2,6 | -1 | penetration 2 |
| FgenesH.VV78X094801.7_3 | PEN2b | AT2G44490.1 | 9E-108 | PEN2 | 7 | 43,2 | -1 | penetration 2 |
| FgenesH.VV78X236649.2_1 | PEN2c | AT2G44490.1 | 8E-107 | PEN2 | 6 | 3,9 | 1 | penetration 2 |
| Sim4.aln-TCVV012986 | PEN2d | AT2G44490.1 | 4E-106 | PEN2 | 19 | 49,0 | 1 | penetration 2 |
| FgenesH.VV78X156423.6_1 | PEN2e | AT2G44490.1 | 2E-146 | PEN2 | 19 | 37,2 | -1 | penetration 2 |
| FgenesH.VV78X189219.4_3 | PEN3a | AT1G59870.1 | 0 | PEN3 | 4 | 12,3 | -1 | penetration 3 |
| FgenesH.VV78X205215.10_1 | PEN3b | AT1G59870.1 | 0 | PEN3 | 6 | 47,7 | -1 | penetration 3 |
| FgenesH.VV78X075372.5_1 | PEN3c | AT1G59870.1 | 0 | PEN3 | 9 | 12,4 | 1 | penetration 3 |
| FgenesH.VV78X110417.4_1 | PEN3d | AT1G59870.1 | 0 | PEN3 | 8 | 56,1 | 1 | penetration 3 |
| FgenesH.VV78X103712.7_1 | PEN3e | AT1G59870.1 | 0 | PEN3 | 13 | 24,1 | -1 | penetration 3 |
| FgenesH.VV78X223301.15_1 | PEN3f | AT1G59870.1 | 0 | PEN3 | 13 | 24,1 | 1 | penetration 3 |
| FgenesH.VV78X127711.9_3 | PEN3g | AT1G59870.1 | 0 | PEN3 | n.d. | n.d. | n.d. | penetration 3 |
| FgenesH.VV78X117936.11_1 | PEN3h | AT1G59870.1 | 0 | PEN3 | 9 | 19,5 | -1 | penetration 3 |
| FgenesH.VV78X160145.5_1 | PEN3i | AT1G59870.1 | 0 | PEN3 | 9 | 12,0 | 1 | penetration 3 |
| FgenesH.VV78X137710.2_2 | PEN3j | AT1G59870.1 | 0 | PEN3 | 9 | 7,6 | 1 | penetration 3 |
| FgenesH.VV78X208183.15_6 | PR1a | AT2G14610.1 | 3E-57 | PR1 | 14 | 4,7 | 1 | pathogenesis-related protein 1 |
| Sim4.aln-A06012971 | PR1b | AT2G14610.1 | 1E-55 | PR1 | 14 | 4,7 | 1 | pathogenesis-related protein 1 |
| FgenesH.VV78X022284.12_1 | PR1c | AT2G14610.1 | 1E-51 | PR1 | 12 | 47,3 | 1 | pathogenesis-related protein 1 |
| FgenesH.VV78X022284.16_1 | PR1d | AT2G14610.1 | 8E-51 | PR1 | 12 | 47,3 | 1 | pathogenesis-related protein 1 |
| Sim4.aln-TCVV020057 | PR1e | AT2G14610.1 | 4E-50 | PR1 | 12 | 47,3 | 1 | pathogenesis-related protein 1 |
| Sim4.aln-A06016268 | PR1f | AT2G14610.1 | 1E-42 | PR1 | 3 | 24,1 | -1 | pathogenesis-related protein 1 |
| Sim4.aln-TCVV015070 | PR1g | AT2G14610.1 | 9E-36 | PR1 | 11 | 48,7 | -1 | pathogenesis-related protein 1 |
| FgenesH.VV78X065845.7_2 | PR1h | AT2G14610.1 | 5E-31 | PR1 | 12 | 47,3 | 1 | pathogenesis-related protein 1 |
| Sim4.aln-TCVV014844 | PR1i | AT2G14610.1 | 1E-57 | PR1 | 11 | 48,5 | 1 | pathogenesis-related protein 1 |
| FgenesH.VV78X104792.17_4 | PR2a | AT3G57260.1 | 5E-100 | PR2 | n.d. | n.d. | n.d. | pathogenesis-related protein 2 |
| Sim4.aln-TCVV014158 | PR2b | AT3G57260.1 | 2E-99 | PR2 | 8 | 62,1 | -1 | pathogenesis-related protein 2 |
| FgenesH.VV78X005385.7_1 | PR2c | AT3G57260.1 | 4E-97 | PR2 | n.d. | n.d. | n.d. | pathogenesis-related protein 2 |
| Sim4.aln-TCVV002522 | PR2d | AT3G57260.1 | 6E-97 | PR2 | 8 | 61,9 | 1 | pathogenesis-related protein 2 |
| Sim4.aln-TCVV013760 | PR2e | AT3G57260.1 | 3E-93 | PR2 | 6 | 42,6 | -1 | pathogenesis-related protein 2 |
| FgenesH.VV79X002804.6_3 | PR2f | AT3G57260.1 | 4E-91 | PR2 | 8 | 61,9 | 1 | pathogenesis-related protein 2 |
| FgenesH.VV78X156035.12_1 | PR2g | AT3G57260.1 | 4E-91 | PR2 | 8 | 61,9 | 1 | pathogenesis-related protein 2 |
| Sim4.aln-TCVV008882 | PR2h | AT3G57260.1 | 2E-105 | PR2 | 8 | 61,9 | 1 | pathogenesis-related protein 2 |
| FgenesH.VV78X092424.7_1 | PR3a | AT3G12500.1 | 4E-131 | PR3 | 3 | 5,7 | -1 | pathogenesis-related protein 3 |
| FgenesH.VV78X202842.8_4 | PR3b | AT3G12500.1 | 1E-126 | PR3 | 4 | 56,5 | 1 | pathogenesis-related protein 3 |
| FgenesH.VV78X202842.8_3 | PR3c | AT3G12500.1 | 6E-108 | PR3 | 4 | 56,5 | 1 | pathogenesis-related protein 3 |
| Sim4.aln-TCVV022799 | PR3d | AT3G12500.1 | 2E-90 | PR3 | 4 | 56,5 | 1 | pathogenesis-related protein 3 |
| FgenesH.VV78X148510.10_2 | PR3e | AT3G12500.1 | 7E-88 | PR3 | 7 | 11,4 | -1 | pathogenesis-related protein 3 |
| Sim4.aln-A06009875 | PR3f | AT3G12500.1 | 2E-131 | PR3 | 3 | 5,7 | -1 | pathogenesis-related protein 3 |
| FgenesH.VV78X140396.8_3 | PR5a | AT1G75040.1 | 2E-77 | PR5 | 13 | 69,3 | 1 | pathogenesis-related protein 5 |
| Sim4.aln-TCVV003768 | PR5b | AT1G75040.1 | 3E-74 | PR5 | 4 | 54,4 | -1 | pathogenesis-related protein 5 |
| Sim4.aln-TCVV023160 | PR5c | AT1G75040.1 | 1E-72 | PR5 | 17 | 3,4 | -1 | pathogenesis-related protein 5 |
| FgenesH.VV78X277947.6_1 | PR5d | AT1G75040.1 | 2E-71 | PR5 | 18 | 35,2 | 1 | pathogenesis-related protein 5 |
| Sim4.aln-TCVV001864 | PR5e | AT1G75040.1 | 2E-68 | PR5 | 18 | 35,2 | -1 | pathogenesis-related protein 5 |
| Sim4.aln-TCVV002553 | PR5f | AT1G75040.1 | 3E-68 | PR5 | 4 | 54,4 | -1 | pathogenesis-related protein 5 |
| Twinscan1.VV78X038915.3_2 | PR5g | AT1G75040.1 | 7E-64 | PR5 | n.d. | n.d. | n.d. | pathogenesis-related protein 5 |
| Sim4.aln-TCVV023777 | PR5h | AT1G75040.1 | 5E-58 | PR5 | n.d. | n.d. | n.d. | pathogenesis-related protein 5 |
| FgenesH.VV78X024334.9_6 | PR5i | AT1G75040.1 | 1E-57 | PR5 | 13 | 9,6 | -1 | pathogenesis-related protein 5 |
| Sim4.aln-TCVV000497 | PR5j | AT1G75040.1 | 2E-55 | PR5 | n.d. | n.d. | n.d. | pathogenesis-related protein 5 |
| Sim4.aln-TCVV022336 | PR5k | AT1G75040.1 | 8E-55 | PR5 | 8 | 49,1 | -1 | pathogenesis-related protein 5 |
| Sim4.aln-TCVV000722 | PR5l | AT1G75040.1 | 5E-52 | PR5 | 2 | 10,4 | -1 | pathogenesis-related protein 5 |
| Sim4.aln-TCVV014680 | PR5m | AT1G75040.1 | 6E-52 | PR5 | 14 | 29,0 | -1 | pathogenesis-related protein 5 |
| Sim4.aln-TCVV002226 | PR5n | AT1G75040.1 | 1E-51 | PR5 | n.d. | n.d. | n.d. | pathogenesis-related protein 5 |
| Sim4.aln-A06001521 | PR5o | AT1G75040.1 | 1E-50 | PR5 | n.d. | n.d. | n.d. | pathogenesis-related protein 5 |
| Sim4.aln-TCVV000596 | PR5p | AT1G75040.1 | 1E-47 | PR5 | 2 | 10,4 | 1 | pathogenesis-related protein 5 |
| FgenesH.VV78X075443.24_1 | PR5q | AT1G75040.1 | 8E-47 | PR5 | n.d. | n.d. | n.d. | pathogenesis-related protein 5 |
| Glimmer1.VV78X113271.4_2 | PR5r | AT1G75040.1 | 1E-46 | PR5 | n.d. | n.d. | n.d. | pathogenesis-related protein 5 |
| Sim4.aln-TCVV023629 | PR5s | AT1G75040.1 | 4E-46 | PR5 | 6 | 13,6 | -1 | pathogenesis-related protein 5 |
| FgenesH.VV78X002500.31_1 | PR5t | AT1G75040.1 | 7E-46 | PR5 | n.d. | n.d. | n.d. | pathogenesis-related protein 5 |
| FgenesH.VV78X244153.5_1 | PR5u | AT1G75040.1 | 5E-89 | PR5 | 18 | 29,3 | 1 | pathogenesis-related protein 5 |
| Sim4.aln-TCVV008048 | RAR1 | AT5G51700.1 | 3E-79 | RAR1 | 16 | 36,8 | -1 | required for Mla12 resistance 1 |
| Sim4.aln-TCVV015903 | SerINa | AT5G43570.1 | 9E-08 | SerPROT-IN | 5 | 18,1 | 1 | serine protease inhibitor |
| Sim4.aln-TCVV005767 | SerINb | AT5G43570.1 | 3E-07 | SerPROT-IN | 5 | 17,9 | -1 | serine protease inhibitor |
| Sim4.aln-TCVV004391 | SerINc | AT5G43570.1 | 3E-06 | SerPROT-IN | 13 | 3,0 | 1 | serine protease inhibitor |
| FgenesH.VV78X012064.11_5 | SerINd | AT5G43570.1 | 7E-10 | SerPROT-IN | n.d. | n.d. | n.d. | serine protease inhibitor |
| Sim4.aln-TCVV011259 | SerIN2a | AT1G47710.1 | 2E-128 | SerPROT-IN2 | 18 | 28,2 | 1 | serine protease inhibitor 2 |
| FgenesH.VV78X101338.11_1 | SerIN2b | AT1G47710.1 | 2E-142 | SerPROT-IN2 | n.d. | n.d. | n.d. | serine protease inhibitor 2 |
| Sim4.aln-TCVV017462 | TryINa | AT1G72290.1 | 2E-05 | TryPROT-IN | 12 | 20,3 | 1 | trypsin and protease inhibitor |
| FgenesH.VV78X069566.6_1 | TryINb | AT1G72290.1 | 4E-05 | TryPROT-IN | 17 | 31,9 | 1 | trypsin and protease inhibitor |
| Sim4.aln-TCVV005057 | TryINc | AT1G72290.1 | 5E-05 | TryPROT-IN | 17 | 31,9 | 1 | trypsin and protease inhibitor |
| FgenesH.VV78X144696.26_1 | TryINd | AT1G72290.1 | 6E-05 | TryPROT-IN | 17 | 31,7 | 1 | trypsin and protease inhibitor |
| FgenesH.VV78X268044.18_2 | TryINe | AT1G72290.1 | 2E-05 | TryPROT-IN | 17 | 31,9 | -1 | trypsin and protease inhibitor |

The NBS proteins were identified on the basis of the NB-ARC domain profile (IPR002182) and were classified according to the domain profiles of plant R proteins present in public domain libraries (Prints, HMMPIR Pfam, and SMART). Assignment to different classes was checked by comparison with Arabidopsis NBS proteins using the BLAST program. Phylogenetic analysis of NBS-LRR proteins was based on distance-matrix neighbour-joining analysis (Clustal X, bootstrap of 1000, [4]) carried out after alignment of sequences by TCoffee (version 5.05, [5]). Other groups of resistance-related proteins were identified by a similarity search with the BLAST program using as reference the sequences of Arabidopsis R proteins. Putative homologues and gene copy numbers were determined on the basis of a threshold of 60% similarity of aligned amino acids and manually checked.
