# Supplementary material for: A High Quality Draft Consensus Sequence of the Genome of a Heterozygous Grapevine Variety
Source: PLoS One. 2007 Dec 19;2(12):e1326. doi: 10.1371/journal.pone.0001326 (PMC2147077; doi:10.1371/journal.pone.0001326)
Supplement: Table S4. — Gene family members involved in the core phenylpropanoid pathway, flavonoid and stilbene branches in V. vinifera. (0.10 MB DOC) [file pone.0001326.s011.doc]

**Table S4.** Gene family members involved in the core phenylpropanoid pathway, flavonoid and stilbene branches in *V. vinifera*.

| Enzyme name | **Gene copy number** | | | **Sequence name** | Pathway | **Grape LGs** |
| --- | --- | --- | --- | --- | --- | --- |
|  | **Grape** | **Poplar** | **Arabidopsis** |  |  |  |
| PHENYLALANINE AMMONIA-LYASE | 13 | 5 | 4 | Vv_PAL a | Phenylpropanoid biosynthesis | 16 |
|  |  |  |  | Vv_PAL b | Phenylpropanoid biosynthesis | 16 |
|  |  |  |  | Vv_PAL c | Phenylpropanoid biosynthesis | 16 |
|  |  |  |  | Vv_PAL d | Phenylpropanoid biosynthesis | 16 |
|  |  |  |  | Vv_PAL e | Phenylpropanoid biosynthesis | 16 |
|  |  |  |  | Vv_PAL f | Phenylpropanoid biosynthesis | 8 |
|  |  |  |  | Vv_PAL g | Phenylpropanoid biosynthesis | 16 |
|  |  |  |  | Vv_PAL h | Phenylpropanoid biosynthesis | 16 |
|  |  |  |  | Vv_PAL i | Phenylpropanoid biosynthesis | 16 |
|  |  |  |  | Vv_PAL j | Phenylpropanoid biosynthesis | 13 |
|  |  |  |  | Vv_PAL k | Phenylpropanoid biosynthesis | 16 |
|  |  |  |  | Vv_PAL l | Phenylpropanoid biosynthesis | 6 |
|  |  |  |  | Vv_PAL m | Phenylpropanoid biosynthesis | 16 |
| CINNAMATE 4-HYDROXYLASE | 1 (2) | 2 (1) | 1 | Vv_C4H a | Phenylpropanoid biosynthesis | 6 |
|  |  |  |  | Vv_C4H b | Phenylpropanoid biosynthesis | 4 |
|  |  |  |  | Vv_C4H c | Phenylpropanoid biosynthesis | 11 |
| COUMARATE-4-CoA LIGASE | 1 | 5 | 4 | Vv_4CL | Phenylpropanoid biosynthesis | 11 |
| CHALCONE SYNTHASE | 4 | 6 (7) | 1 (3) | Vv_CHS a | Flavonoid biosynthesis | 14 |
|  |  |  |  | Vv_CHS b | Flavonoid biosynthesis | 14 |
|  |  |  |  | Vv_CHS c | Flavonoid biosynthesis | N/D |
|  |  |  |  | Vv_CHS d | Flavonoid biosynthesis | N/D |
| STILBENE SYNTHASE | 21 | 0 | 0 | Vv_STSY a | Stilbene biosynthesis | 16 |
|  |  |  |  | Vv_STSY b | Stilbene biosynthesis | 16 |
|  |  |  |  | Vv_STSY c | Stilbene biosynthesis | 16 |
|  |  |  |  | Vv_STSY d | Stilbene biosynthesis | 16 |
|  |  |  |  | Vv_STSY e | Stilbene biosynthesis | 16 |
|  |  |  |  | Vv_STSY f | Stilbene biosynthesis | 16 |
|  |  |  |  | Vv_STSY g | Stilbene biosynthesis | 16 |
|  |  |  |  | Vv_STSY h | Stilbene biosynthesis | 16 |
|  |  |  |  | Vv_STSY i | Stilbene biosynthesis | 16 |
|  |  |  |  | Vv_STSY j | Stilbene biosynthesis | 16 |
|  |  |  |  | Vv_STSY k | Stilbene biosynthesis | 16 |
|  |  |  |  | Vv_STSY l | Stilbene biosynthesis | 16 |
|  |  |  |  | Vv_STSY m | Stilbene biosynthesis | 10 |
|  |  |  |  | Vv_STSY n | Stilbene biosynthesis | 10 |
|  |  |  |  | Vv_STSY o | Stilbene biosynthesis | 16 |
|  |  |  |  | Vv_STSY p | Stilbene biosynthesis | 10 |
|  |  |  |  | Vv_STSY q | Stilbene biosynthesis | 10 |
|  |  |  |  | Vv_STSY r | Stilbene biosynthesis | 16 |
|  |  |  |  | Vv_STSY s | Stilbene biosynthesis | 10 |
|  |  |  |  | Vv_STSY t | Stilbene biosynthesis | 16 |
|  |  |  |  | Vv_STSY u | Stilbene biosynthesis | N/D |
| CHALCONE ISOMERASE | 1 | 1 (2) | 1 (2) | Vv_CHI | Flavonoid biosynthesis | 13 |
| FLAVANONE 3-HYDROXYLASE | 3 | 1 | 1 | Vv_F3H | Flavonoid biosynthesis | 4 |
|  |  |  |  | Vv_F3H | Flavonoid biosynthesis | 18 |
|  |  |  |  | Vv_F3H | Flavonoid biosynthesis | 4 |
| FLAVONOID 3'-HYDROXYLASE | 1 | 1 | 1 | Vv_F3'H | Flavonoid biosynthesis | 17 |
| FLAVONOID 3',5'-HYDROXYLASE | 10 | 2 | 0 | Vv_F3’5’H a | Flavonoid biosynthesis | 6 |
|  |  |  |  | Vv_F3’5’H b | Flavonoid biosynthesis | N/D |
|  |  |  |  | Vv_F3’5’H c | Flavonoid biosynthesis | 6 |
|  |  |  |  | Vv_F3’5’H d | Flavonoid biosynthesis | 6 |
|  |  |  |  | Vv_F3’5’H e | Flavonoid biosynthesis | 6 |
|  |  |  |  | Vv_F3’5’H f | Flavonoid biosynthesis | 6 |
|  |  |  |  | Vv_F3’5’H g | Flavonoid biosynthesis | N/D |
|  |  |  |  | Vv_F3’5’H h | Flavonoid biosynthesis | 8 |
|  |  |  |  | Vv_F3’5’H i | Flavonoid biosynthesis | N/D |
|  |  |  |  | Vv_F3’5’H j | Flavonoid biosynthesis | 6 |
| FLAVONOL SYNTHASE | 4 | 4 | 6 | Vv_FLS a | Flavonoid biosynthesis | 18 |
|  |  |  |  | Vv_FLS b | Flavonoid biosynthesis | 18 |
|  |  |  |  | Vv_FLS c | Flavonoid biosynthesis | 18 |
|  |  |  |  | Vv_FLS d | Flavonoid biosynthesis | 18 |
| Dihydroflavonol-4-reductase | 1 | 2 | 1 | Vv_DFR | Flavonoid biosynthesis | 18 |
| leucoanthocyanidin dioxygenase | 1 | 2 | 1 | Vv_LDOX | Flavonoid biosynthesis | 2 |
| leucoanthocyanidin reductase | 2 | 3 | 0 | Vv_LAR a | Flavonoid biosynthesis | 1 |
|  |  |  |  | Vv_LAR a | Flavonoid biosynthesis | 3 |
| anthocyanidin reductase | 1 | 2 | 1 | Vv_DFR | Flavonoid biosynthesis | 10 |
| UDP-glucose: flavanoid 3-O-glucosyltransferase | 1 | 1 | 1 | Vv_UFGT | Flavonoid biosynthesis | 16 |

Biochemical pathways and gene copy numbers in grape, poplar and Arabidopsis are reported for each enzyme. For each gene member, the sequence name and specific LG location are given. Poplar and Arabidopsis gene copy numbers were retrieved from the *Populus* genome portal (v1.1) (http://genome.jgi-psf.org/Poptr1_1) and the TAIR database (www.arabidopsis.org) respectively. Numbers in parenthesis indicate ´–like´ genes. N/D refers to sequences not anchored to LGs.
