# Supplementary material for: A High Quality Draft Consensus Sequence of the Genome of a Heterozygous Grapevine Variety
Source: PLoS One. 2007 Dec 19;2(12):e1326. doi: 10.1371/journal.pone.0001326 (PMC2147077; doi:10.1371/journal.pone.0001326)
Supplement: Table S5. — Putative genes encoding enzymes participating in the terpenoid pathway of V. vinifera. (0.15 MB DOC) [file pone.0001326.s012.doc]

**Table S5.** Putative genes encoding enzymes participating in the terpenoid pathway of *V. vinifera*.

| **Enzyme name** | **Sequence name** | **Putative process** | **Grape LGs** |
| --- | --- | --- | --- |
| NCED2 (NINE-CIS-EPOXYCAROTENOID DIOXYGENASE 2) | Vv_NCED2 a | abscisic acid biosynthetic process | 10 |
|  | Vv_NCED2 b | abscisic acid biosynthetic process | 19 |
| ABA3/ATABA3/LOS5/SIR3 (ABA DEFICIENT 3) (Mo-molybdopterin cofactor sulfurase/ selenocysteine lyase) | Vv_ABA3 | abscisic acid biosynthetic process | 19 |
| ABA2 (ABA DEFICIENT 2); oxidoreductase | Vv_ABA2 a | abscisic acid biosynthetic process | 6 |
|  | Vv_ABA2 b | abscisic acid catabolic process | 3 |
| UDP-GLYCOSYLTRANSFERASE/ABSCISIC ACID GLUCOSYLTRANSFERASE/ TRANSFERASE, TRANSFERRING GLYCOSYL GROUPS | Vv_ABAGT a | abscisic acid catabolic process | 12 |
|  | Vv_ABAGT B | abscisic acid metabolic process | 2 |
| CYP707A1 (cytochrome P450, family 707, subfamily A, polypeptide 1); oxygen binding | Vv_CYPT707A1 a | abscisic acid metabolic process | 3 |
|  | Vv_CYPT707A1 b | abscisic acid metabolic process | 6 |
|  | Vv_CYPT707A1 c | abscisic acid metabolic process | 7 |
|  | Vv_CYPT707A1 d | abscisic acid metabolic process | 18 |
|  | Vv_CYPT707A1 e | abscisic acid metabolic process | 5 |
| PHYB (PHYTOCHROME B); G-protein coupled photoreceptor/ signal transducer | Vv_PHYB a | abscisic acid metabolic process | 12 |
|  | Vv_PHYB b | abscisic acid metabolic process | 6 |
| AAO2 (ALDEHYDE OXIDASE 2) | Vv_AAO2 a | abscisic acid metabolic process | 18 |
|  | Vv_AAO2 B | abscisic acid metabolic process | 13 |
| CESA8 (CELLULASE SYNTHASE 8); cellulose synthase/ transferase, transferring glycosyl groups | Vv_CESA8 a | positive regulation of abscisic acid biosynthetic process | 10 |
|  | Vv_CESA8 b | positive regulation of abscisic acid biosynthetic process | 7 |
|  | Vv_CESA8 c | positive regulation of abscisic acid biosynthetic process | 1 |
|  | Vv_CESA8 d | positive regulation of abscisic acid biosynthetic process | 7 |
|  | Vv_CESA8 e | positive regulation of abscisic acid biosynthetic process | 4 |
|  | Vv_CESA8 f | positive regulation of abscisic acid biosynthetic process | 7 |
|  | Vv_CESA8 g | positive regulation of abscisic acid biosynthetic process | 16 |
| FUS3 (FUSCA 3); DNA binding / transcription factor | Vv_FUS3 | positive regulation of abscisic acid biosynthetic process | 10 |
| BETA-OHASE 2 (BETA-CAROTENE HYDROXYLASE 2); beta-carotene hydroxylase | Vv_BETA-OHASE2 | carotene catabolic process | 2 |
| ACLA-1 (ATP-citrate lyase A-1) | Vv_ACLA-1 | carotenoids metabolic process | 1 |
| CRTISO (carotenoid isomerase); carotenoid isomerase | Vv_CRTISO | carotenoids metabolic process | 8 |
| CYP97A3/LUT5 (CYTOCHROME P450-TYPE MONOOXYGENASE 97A3); carotene beta-ring hydroxylase/ oxygen binding | Vv_CYP97A3 a | carotenoids metabolic process | 4 |
|  | Vv_CYP97A3 b | carotenoids metabolic process | 8 |
| IM (IMMUTANS) Alternative oxidase 4, chloroplast precursor | VV_IM | carotenoids metabolic process | 10 |
| LUT2 (LUTEIN DEFICIENT 2); lycopene epsilon cyclase | VV_LUT2 | carotenoids metabolic process | 11 |
| CYP711A1 (MORE AXILLARY BRANCHES 1); oxygen binding / cytochrome P450 | Vv_CYP771A1 | carotenoids metabolic process | 4 |
| PAC (PALE CRESS) / similar to moesin actin-binding domain homologue | Vv_PAC | carotenoids metabolic process | 7 |
| PDS1 (PHYTOENE DESATURASE 1) | Vv_PDS1 | carotenoids metabolic process | 12 |
| PDS3 (PHYTOENE DESATURASE 3) | Vv_PDS3 | carotenoids metabolic process | 14 |
| PSY (PHYTOENE SYNTHASE); geranylgeranyl-diphosphate geranylgeranyltransferase | Vv_PSY | carotenoids metabolic process | 12 |
| ZDS (ZETA-CAROTENE DESATURASE); carotene 7,8-desaturase | Vv_ZDS | caroteooids metabolic process | 14 |
| LYC (LYCOPENE CYCLASE) | Vv_LYC a | carotenoids metabolic process | 8 |
|  | Vv_LYC b | carotenoids metabolic process | 6 |
| NPQ1 (NON-PHOTOCHEMICAL QUENCHING 1) / Violaxanthin de-epoxidase (VDE). | Vv_VDE | carotenoids metabolic process | 9 |
| UNKNOWN PROTEIN / similar to Os02g0651300 | Vv_UNKNOWN a | carotenoids metabolic process | 5 |
|  | Vv_UNKNOWN b | carotenoids metabolic process | 8 |
| RIF10 (RESISTANT TO INHIBITION WITH FSM 10); 3'-5'-exoribonuclease/ RNA binding / nucleic acid binding | Vv_RIF10 | carotenoids metabolic process | 8 |
| ABA1 (ABA DEFICIENT 1); zeaxanthin epoxidase | Vv_ABA1 a | xanthophyll biosynthetic process | 13 |
|  | Vv_ABA1 b | xanthophyll biosynthetic process | 7 |
| CCD1 (CAROTENOID CLEAVAGE DIOXYGENASE 1) | Vv_CCD1 | xanthophyll catabolic process | 1 |
| CCD8 (CAROTENOID CLEAVAGE DIOXYGENASE 8) | Vv_CCD8 | xanthophyll catabolic process | 4 |
| VTE1 (VITAMIN E DEFICIENT 1) / Tocopherol cyclase | Vv_VTE1 | xanthophyll metabolic process | 4 |
| PRENYLTRANSFERASE | Vv_PT a | general | 4 |
|  | Vv_PT b | general | 5 |
|  | Vv_PT c | general | 19 |
|  | Vv_PT d | general | 18 |
| GGPS (GERANYLGERANYL PIROPHOSPSHATE SYNTHASE) | Vv_GGPS | general | 6 |
| IDI (ISOPENTENYL DIPHOSPHATE δ-ISOMERASE) | Vv_IDI | general | 4 |
| GA4; GIBBERELLIN 3-BETA-DIOXYGENASE | Vv_GA4 | gibberellic acid biosynthetic process | 9 |
| GA2; ENT-KAURENE SYNTHASE | Vv_GA2 | gibberellic acid biosynthetic process | 19 |
| GA1; ENT-COPALYL DIPHOSPHATE SYNTHASE | Vv_GA1 | gibberellic acid biosynthetic process | 7 |
| GA3; OXYGEN BINDING | Vv_GA3 | gibberellic acid biosynthetic process | 18 |
| GA20; GIBBERELLIN 20-OXIDASE-RELATED | Vv_GA20OX a | gibberellic acid biosynthetic process | 15 |
|  | Vv_GA20OX b | gibberellic acid biosynthetic process | 2 |
| GA2OX7 (GIBBERELLIN 2-OXIDASE 7 | Vv_GA2OX7 | gibberellic acid biosynthetic process | 16 |
| GA2OX1 (GIBBERELLIN 2-OXIDASE 1) | Vv_GA2OX1 | gibberellic acid biosynthetic process | 9 |
| CTR1 (CONSTITUTIVE TRIPLE RESPONSE 1); kinase/ protein threonine/tyrosine kinase | Vv_CTR1 a | gibberellic acid biosynthetic process | 8 |
|  | Vv_CTR1 b | gibberellic acid biosynthetic process | 4 |
| GA5; GIBBERELLIN 20-OXIDASE/ GIBBERELLIN 3-BETA-DIOXYGENASE | Vv_GA5 | gibberellic acid biosynthetic process | 15 |
| GA2OX4 (GIBBERELLIN 2-OXIDASE 4); gibberellin 2-beta-dioxygenase | Vv_GA2OX4 | gibberellic acid biosynthetic process | 5 |
| TES (α-TERPINEOL SYNTHASE) | Vv_TES a | monoterpenoid biosynthesis pathway | 13 |
|  | Vv_TES b | monoterpenoid biosynthesis pathway | 16 |
|  | Vv_TES c | monoterpenoid biosynthesis pathway | 12 |
| LIS1 (LINALOOL SYNTHASE 1) | Vv_LIS1 a | monoterpenoid biosynthesis pathway | 10 |
| LIS2 (LINALOOL SYNTHASE 2) | Vv_LIS2 | monoterpenoid biosynthesis pathway | 10 |
| LIS1 (LINALOOL SYNTHASE 1) | Vv_LIS1 b | monoterpenoid biosynthesis pathway | 9 |
| LIMS ((R)-LIMONENE SYNTHASE 1); (+)-limonene synthase 1 | Vv_LIMS | monoterpenoid biosynthesis pathway | 19 |
| TPS-CIN (TERPENE SYNTHASE-LIKE SEQUENCE-1,8-CINEOLE); myrcene/(E)-beta-ocimene synthase | Vv_TPS-CIN a | monoterpenoid biosynthesis pathway | 1 |
|  | Vv_TPS-CIN b | monoterpenoid biosynthesis pathway | 17 |
|  | Vv_TPS-CIN c | monoterpenoid biosynthesis pathway | 14 |
|  | Vv_TPS-CIN d | monoterpenoid biosynthesis pathway | 1 |
| LUP2 (LUPEOL SYNTHASE 2) | Vv_LUP2 a | triterpenoids | 11 |
|  | Vv_LUP2 b | triterpenoids | 14 |
|  | Vv_LUP2 c | triterpenoids | 10 |
|  | Vv_LUP2 d | triterpenoids | 4 |
|  | Vv_LUP2 e | triterpenoids | 10 |
| CAS1 (CYCLOARTENOL SYNTHASE 1) | Vv_CAS1 | Sterols | 9 |
| PENTACYCLIC TRITERPENE SYNTHASE | Vv_PPS a | triterpenoids | 8 |
|  | Vv_PPS b | triterpenoids | 6 |
|  | Vv_PPS c | triterpenoids | 13 |
| TERPENE SYNTHASE/ CYCLASE family protein | Vv_TPS a | sesquiterpenoid process | 7 |
|  | Vv_TPS b | sesquiterpenoid process | 16 |
| TPS13/TPS13 (TERPENOID SYNTHASE13); cyclase | Vv_TPS c | sesquiterpenoid process | 18 |
| TPS (TERPENE SYNTHASE-like) | Vv_TPS d | terpene synthase | 19 |
|  | Vv_TPS e | terpene synthase | 16 |
|  | Vv_TPS f | terpene synthase | 19 |
| TERPENE CYCLASE/MUTASE-RELATED /CINNAMYL-ALCOHOL DEHYDROGENASE FAMILY | Vv_TPS g | terpenoid metabolic process | 18 |
| AACT (ACETOACETYL-CoA THIOLASE) | Vv_AACT a | mevalonate pathway | 12 |
|  | Vv_AACT b | mevalonate pathway | 10 |
| HMGS (HMG-CoA SYNTHASE) (3-hydroxy-3-methylglutaryl coenzyme A synthase) | Vv_HMGS | mevalonate pathway | 2 |
| HMGR1 (3-HYDROXY-3-METHYLGLUTARYL COA REDUCTASE) | Vv_HMGR1 | mevalonate pathway | 18 |
| HMGR2 (3-HYDROXY-3-METHYLGLUTARYL COA REDUCTASE 2) | Vv_HMGR2 a | mevalonate pathway | 3 |
|  | Vv_HMGR2 b | mevalonate pathway | 18 |
| MK/MVK (MEVALONATE KINASE / PHOSPHOMEVALONATE KINASE) | Vv_MVK | mevalonate pathway | 14 |
| MVD1 (MEVALONATE DIPHOSPHATE DECARBOXYLASE 1) | Vv_MVD1 | mevalonate pathway | 13 |
| DXPS1 (DXP SYNTHASE 1); 1-deoxy-D-xylulose-5-phosphate synthase 1 | Vv_DXS1 | mevalonate-independent pathway | 5 |
| DXPS2 (DXP SYNTHASE 2); 1-deoxy-D-xylulose-5-phosphate synthase 2 | Vv_DXS2 a | mevalonate-independent pathway | 7 |
|  | Vv_DXS2 b | mevalonate-independent pathway | 15 |
| DXPS3 (DXP SYNTHASE 3); 1-deoxy-D-xylulose-5-phosphate synthase 3 | Vv_DXS3 | mevalonate-independent pathway | 4 |
| DXR (1-deoxyxylulose-5-phosphate reductoisomerase) | Vv_DXR | mevalonate-independent pathway | 17 |
| ISPD (CDP-ME SYNTHASE) (4-Diphosphocytidyl-2C-methyl-D-erythritol synthase) | Vv_ISPD | mevalonate-independent pathway | 12 |
| ISPE (CDPMEK) (4-diphosphocytidyl-2C-methyl-D-erythritol kinase) | Vv_ISPE | mevalonate-independent pathway | 6 |
| ISPF (MECDP-synthase); 2-C-methyl-D-erythritol 2,4-cyclodiphosphate synthase | Vv_ISPF | mevalonate-independent pathway | 2 |
| ISPH (HMBPP REDUCTASE) (1-hydroxy-2-methyl-butenyl 4-diphosphate reductase) | Vv_ISPH | mevalonate-independent pathway | 3 |

Sequence name, putative pathway and location on the grape LGs are reported. The number of genes anchored per family was obtained by performing either a BLAST search of known sequences belonging to terpenoid pathways against gene prediction, or by retrieving gene predictions based on their putative function assigned by the KEGG and InterPro.
