# Supplementary material for: A High Quality Draft Consensus Sequence of the Genome of a Heterozygous Grapevine Variety
Source: PLoS One. 2007 Dec 19;2(12):e1326. doi: 10.1371/journal.pone.0001326 (PMC2147077; doi:10.1371/journal.pone.0001326)
Supplement: Table S6. — Transcription factors of V. vinifera. (2.20 MB DOC) [file pone.0001326.s013.doc]

**Table S6.** Transcription factors of *V. vinifera*.

| Gene_ID | **IPR (InterPro N.)** | **Transcription Factor family** | **Annotation (Pfam database)** |
| --- | --- | --- | --- |
| FgenesH.VV78X011820.9_2 | IPR003340 | ABI3VP1 | Transcriptional factor B3 |
| FgenesH.VV78X016243.17_2 | IPR003340 | ABI3VP1 | Transcriptional factor B3 |
| FgenesH.VV78X035471.3_5 | IPR003340 | ABI3VP1 | Transcriptional factor B3 |
| FgenesH.VV78X061885.11_1 | IPR003340 | ABI3VP1 | Transcriptional factor B3 |
| FgenesH.VV78X081207.5_1 | IPR003340 | ABI3VP1 | Transcriptional factor B3 |
| FgenesH.VV78X113969.14_1 | IPR003340 | ABI3VP1 | Transcriptional factor B3 |
| FgenesH.VV78X128587.3_5 | IPR003340 | ABI3VP1 | Transcriptional factor B3 |
| FgenesH.VV78X150189.9_1 | IPR003340 | ABI3VP1 | Transcriptional factor B3 |
| FgenesH.VV78X179024.3_7 | IPR003340 | ABI3VP1 | Transcriptional factor B3 |
| FgenesH.VV78X204419.5_4 | IPR003340 | ABI3VP1 | Transcriptional factor B3 |
| FgenesH.VV78X221870.3_4 | IPR003340 | ABI3VP1 | Transcriptional factor B3 |
| FgenesH.VV78X234759.34_5 | IPR003340 | ABI3VP1 | Transcriptional factor B3 |
| FgenesH.VV78X234759.34_6 | IPR003340 | ABI3VP1 | Transcriptional factor B3 |
| FgenesH.VV78X234759.34_9 | IPR003340 | ABI3VP1 | Transcriptional factor B3 |
| Sim4.aln-TCVV002072 | IPR003340 | ABI3VP1 | Transcriptional factor B3 |
| Sim4.aln-TCVV006121 | IPR003340 | ABI3VP1 | Transcriptional factor B3 |
| Sim4.aln-TCVV010739 | IPR003340 | ABI3VP1 | Transcriptional factor B3 |
| Sim4.aln-TCVV011315 | IPR003340 | ABI3VP1 | Transcriptional factor B3 |
| Sim4.aln-TCVV012691 | IPR003340 | ABI3VP1 | Transcriptional factor B3 |
| FgenesH.VV78X002223.5_1 | IPR001471 | AP2-EREBP | Pathogenesis-related transcriptional factor and ERF |
| FgenesH.VV78X006745.10_1 | IPR001471 | AP2-EREBP | Pathogenesis-related transcriptional factor and ERF |
| FgenesH.VV78X008158.8_1 | IPR001471 | AP2-EREBP | Pathogenesis-related transcriptional factor and ERF |
| FgenesH.VV78X014636.4_1 | IPR001471 | AP2-EREBP | Pathogenesis-related transcriptional factor and ERF |
| FgenesH.VV78X014656.31_1 | IPR001471 | AP2-EREBP | Pathogenesis-related transcriptional factor and ERF |
| FgenesH.VV78X015432.3_1 | IPR001471 | AP2-EREBP | Pathogenesis-related transcriptional factor and ERF |
| FgenesH.VV78X027619.6_3 | IPR001471 | AP2-EREBP | Pathogenesis-related transcriptional factor and ERF |
| FgenesH.VV78X029871.26_2 | IPR001471 | AP2-EREBP | Pathogenesis-related transcriptional factor and ERF |
| FgenesH.VV78X034923.8_1 | IPR001471 | AP2-EREBP | Pathogenesis-related transcriptional factor and ERF |
| FgenesH.VV78X036856.13_1 | IPR001471 | AP2-EREBP | Pathogenesis-related transcriptional factor and ERF |
| FgenesH.VV78X051556.7_8 | IPR001471 | AP2-EREBP | Pathogenesis-related transcriptional factor and ERF |
| FgenesH.VV78X051777.28_1 | IPR001471 | AP2-EREBP | Pathogenesis-related transcriptional factor and ERF |
| FgenesH.VV78X063704.11_1 | IPR001471 | AP2-EREBP | Pathogenesis-related transcriptional factor and ERF |
| FgenesH.VV78X065241.7_1 | IPR001471 | AP2-EREBP | Pathogenesis-related transcriptional factor and ERF |
| FgenesH.VV78X076062.5_1 | IPR001471 | AP2-EREBP | Pathogenesis-related transcriptional factor and ERF |
| FgenesH.VV78X077500.11_1 | IPR001471 | AP2-EREBP | Pathogenesis-related transcriptional factor and ERF |
| FgenesH.VV78X086884.5_2 | IPR001471 | AP2-EREBP | Pathogenesis-related transcriptional factor and ERF |
| FgenesH.VV78X087658.7_4 | IPR001471 | AP2-EREBP | Pathogenesis-related transcriptional factor and ERF |
| FgenesH.VV78X087755.4_1 | IPR001471 | AP2-EREBP | Pathogenesis-related transcriptional factor and ERF |
| FgenesH.VV78X088191.10_4 | IPR001471 | AP2-EREBP | Pathogenesis-related transcriptional factor and ERF |
| FgenesH.VV78X088704.6_1 | IPR001471 | AP2-EREBP | Pathogenesis-related transcriptional factor and ERF |
| FgenesH.VV78X091249.7_1 | IPR001471 | AP2-EREBP | Pathogenesis-related transcriptional factor and ERF |
| FgenesH.VV78X110092.5_2 | IPR001471 | AP2-EREBP | Pathogenesis-related transcriptional factor and ERF |
| FgenesH.VV78X112440.8_1 | IPR001471 | AP2-EREBP | Pathogenesis-related transcriptional factor and ERF |
| FgenesH.VV78X137856.13_2 | IPR001471 | AP2-EREBP | Pathogenesis-related transcriptional factor and ERF |
| FgenesH.VV78X155042.5_3 | IPR001471 | AP2-EREBP | Pathogenesis-related transcriptional factor and ERF |
| FgenesH.VV78X161484.7_2 | IPR001471 | AP2-EREBP | Pathogenesis-related transcriptional factor and ERF |
| FgenesH.VV78X178844.5_1 | IPR001471 | AP2-EREBP | Pathogenesis-related transcriptional factor and ERF |
| FgenesH.VV78X185891.8_3 | IPR001471 | AP2-EREBP | Pathogenesis-related transcriptional factor and ERF |
| FgenesH.VV78X197307.7_1 | IPR001471 | AP2-EREBP | Pathogenesis-related transcriptional factor and ERF |
| FgenesH.VV78X199584.24_2 | IPR001471 | AP2-EREBP | Pathogenesis-related transcriptional factor and ERF |
| FgenesH.VV78X209729.3_5 | IPR001471 | AP2-EREBP | Pathogenesis-related transcriptional factor and ERF |
| FgenesH.VV78X220757.27_1 | IPR001471 | AP2-EREBP | Pathogenesis-related transcriptional factor and ERF |
| FgenesH.VV78X228761.5_1 | IPR001471 | AP2-EREBP | Pathogenesis-related transcriptional factor and ERF |
| FgenesH.VV78X232607.28_1 | IPR001471 | AP2-EREBP | Pathogenesis-related transcriptional factor and ERF |
| FgenesH.VV78X232680.8_3 | IPR001471 | AP2-EREBP | Pathogenesis-related transcriptional factor and ERF |
| FgenesH.VV78X235548.5_1 | IPR001471 | AP2-EREBP | Pathogenesis-related transcriptional factor and ERF |
| FgenesH.VV78X236602.19_2 | IPR001471 | AP2-EREBP | Pathogenesis-related transcriptional factor and ERF |
| FgenesH.VV78X237477.4_1 | IPR001471 | AP2-EREBP | Pathogenesis-related transcriptional factor and ERF |
| FgenesH.VV78X245375.21_3 | IPR001471 | AP2-EREBP | Pathogenesis-related transcriptional factor and ERF |
| FgenesH.VV78X254758.5_2 | IPR001471 | AP2-EREBP | Pathogenesis-related transcriptional factor and ERF |
| FgenesH.VV78X258666.10_2 | IPR001471 | AP2-EREBP | Pathogenesis-related transcriptional factor and ERF |
| FgenesH.VV78X258697.4_1 | IPR001471 | AP2-EREBP | Pathogenesis-related transcriptional factor and ERF |
| FgenesH.VV78X271076.15_2 | IPR001471 | AP2-EREBP | Pathogenesis-related transcriptional factor and ERF |
| FgenesH.VV78X271076.15_6 | IPR001471 | AP2-EREBP | Pathogenesis-related transcriptional factor and ERF |
| FgenesH.VV78X277019.10_3 | IPR001471 | AP2-EREBP | Pathogenesis-related transcriptional factor and ERF |
| FgenesH.VV79X000700.5_6 | IPR001471 | AP2-EREBP | Pathogenesis-related transcriptional factor and ERF |
| FgenesH.VV79X002248.2_3 | IPR001471 | AP2-EREBP | Pathogenesis-related transcriptional factor and ERF |
| FgenesH.VV79X002296.12_1 | IPR001471 | AP2-EREBP | Pathogenesis-related transcriptional factor and ERF |
| FgenesH.VV79X002628.1_1 | IPR001471 | AP2-EREBP | Pathogenesis-related transcriptional factor and ERF |
| Glimmer1.VV78X029792.14_5 | IPR001471 | AP2-EREBP | Pathogenesis-related transcriptional factor and ERF |
| Glimmer1.VV78X039739.4_1 | IPR001471 | AP2-EREBP | Pathogenesis-related transcriptional factor and ERF |
| Glimmer1.VV78X041419.2_4 | IPR001471 | AP2-EREBP | Pathogenesis-related transcriptional factor and ERF |
| Glimmer1.VV78X064410.6_4 | IPR001471 | AP2-EREBP | Pathogenesis-related transcriptional factor and ERF |
| Glimmer1.VV78X081361.4_2 | IPR001471 | AP2-EREBP | Pathogenesis-related transcriptional factor and ERF |
| Glimmer1.VV78X085330.6_5 | IPR001471 | AP2-EREBP | Pathogenesis-related transcriptional factor and ERF |
| Glimmer1.VV78X105475.7_3 | IPR001471 | AP2-EREBP | Pathogenesis-related transcriptional factor and ERF |
| Glimmer1.VV78X135793.4_2 | IPR001471 | AP2-EREBP | Pathogenesis-related transcriptional factor and ERF |
| Glimmer1.VV78X141375.2_1 | IPR001471 | AP2-EREBP | Pathogenesis-related transcriptional factor and ERF |
| Glimmer1.VV78X194205.5_1 | IPR001471 | AP2-EREBP | Pathogenesis-related transcriptional factor and ERF |
| Glimmer1.VV78X237697.6_2 | IPR001471 | AP2-EREBP | Pathogenesis-related transcriptional factor and ERF |
| Glimmer1.VV78X252907.7_1 | IPR001471 | AP2-EREBP | Pathogenesis-related transcriptional factor and ERF |
| Glimmer1.VV78X268578.2_1 | IPR001471 | AP2-EREBP | Pathogenesis-related transcriptional factor and ERF |
| Sim4.aln-A06010527 | IPR001471 | AP2-EREBP | Pathogenesis-related transcriptional factor and ERF |
| Sim4.aln-A06011063 | IPR001471 | AP2-EREBP | Pathogenesis-related transcriptional factor and ERF |
| Sim4.aln-A06011836 | IPR001471 | AP2-EREBP | Pathogenesis-related transcriptional factor and ERF |
| Sim4.aln-A06015964 | IPR001471 | AP2-EREBP | Pathogenesis-related transcriptional factor and ERF |
| Sim4.aln-A06017254 | IPR001471 | AP2-EREBP | Pathogenesis-related transcriptional factor and ERF |
| Sim4.aln-A06017345 | IPR001471 | AP2-EREBP | Pathogenesis-related transcriptional factor and ERF |
| Sim4.aln-A06017475 | IPR001471 | AP2-EREBP | Pathogenesis-related transcriptional factor and ERF |
| Sim4.aln-A06017517 | IPR001471 | AP2-EREBP | Pathogenesis-related transcriptional factor and ERF |
| Sim4.aln-A06017627 | IPR001471 | AP2-EREBP | Pathogenesis-related transcriptional factor and ERF |
| Sim4.aln-A06018309 | IPR001471 | AP2-EREBP | Pathogenesis-related transcriptional factor and ERF |
| Sim4.aln-A06018312 | IPR001471 | AP2-EREBP | Pathogenesis-related transcriptional factor and ERF |
| Sim4.aln-TCVV000106 | IPR001471 | AP2-EREBP | Pathogenesis-related transcriptional factor and ERF |
| Sim4.aln-TCVV000115 | IPR001471 | AP2-EREBP | Pathogenesis-related transcriptional factor and ERF |
| Sim4.aln-TCVV000408 | IPR001471 | AP2-EREBP | Pathogenesis-related transcriptional factor and ERF |
| Sim4.aln-TCVV000614 | IPR001471 | AP2-EREBP | Pathogenesis-related transcriptional factor and ERF |
| Sim4.aln-TCVV000833 | IPR001471 | AP2-EREBP | Pathogenesis-related transcriptional factor and ERF |
| Sim4.aln-TCVV001329 | IPR001471 | AP2-EREBP | Pathogenesis-related transcriptional factor and ERF |
| Sim4.aln-TCVV001456 | IPR001471 | AP2-EREBP | Pathogenesis-related transcriptional factor and ERF |
| Sim4.aln-TCVV001676 | IPR001471 | AP2-EREBP | Pathogenesis-related transcriptional factor and ERF |
| Sim4.aln-TCVV001706 | IPR001471 | AP2-EREBP | Pathogenesis-related transcriptional factor and ERF |
| Sim4.aln-TCVV001718 | IPR001471 | AP2-EREBP | Pathogenesis-related transcriptional factor and ERF |
| Sim4.aln-TCVV001769 | IPR001471 | AP2-EREBP | Pathogenesis-related transcriptional factor and ERF |
| Sim4.aln-TCVV001815 | IPR001471 | AP2-EREBP | Pathogenesis-related transcriptional factor and ERF |
| Sim4.aln-TCVV001890 | IPR001471 | AP2-EREBP | Pathogenesis-related transcriptional factor and ERF |
| Sim4.aln-TCVV001923 | IPR001471 | AP2-EREBP | Pathogenesis-related transcriptional factor and ERF |
| Sim4.aln-TCVV001993 | IPR001471 | AP2-EREBP | Pathogenesis-related transcriptional factor and ERF |
| Sim4.aln-TCVV002148 | IPR001471 | AP2-EREBP | Pathogenesis-related transcriptional factor and ERF |
| Sim4.aln-TCVV003452 | IPR001471 | AP2-EREBP | Pathogenesis-related transcriptional factor and ERF |
| Sim4.aln-TCVV006566 | IPR001471 | AP2-EREBP | Pathogenesis-related transcriptional factor and ERF |
| Sim4.aln-TCVV006794 | IPR001471 | AP2-EREBP | Pathogenesis-related transcriptional factor and ERF |
| Sim4.aln-TCVV007744 | IPR001471 | AP2-EREBP | Pathogenesis-related transcriptional factor and ERF |
| Sim4.aln-TCVV008647 | IPR001471 | AP2-EREBP | Pathogenesis-related transcriptional factor and ERF |
| Sim4.aln-TCVV008800 | IPR001471 | AP2-EREBP | Pathogenesis-related transcriptional factor and ERF |
| Sim4.aln-TCVV008877 | IPR001471 | AP2-EREBP | Pathogenesis-related transcriptional factor and ERF |
| Sim4.aln-TCVV009829 | IPR001471 | AP2-EREBP | Pathogenesis-related transcriptional factor and ERF |
| Sim4.aln-TCVV010626 | IPR001471 | AP2-EREBP | Pathogenesis-related transcriptional factor and ERF |
| Sim4.aln-TCVV011753 | IPR001471 | AP2-EREBP | Pathogenesis-related transcriptional factor and ERF |
| Sim4.aln-TCVV012214 | IPR001471 | AP2-EREBP | Pathogenesis-related transcriptional factor and ERF |
| Sim4.aln-TCVV012903 | IPR001471 | AP2-EREBP | Pathogenesis-related transcriptional factor and ERF |
| Sim4.aln-TCVV013125 | IPR001471 | AP2-EREBP | Pathogenesis-related transcriptional factor and ERF |
| Sim4.aln-TCVV014793 | IPR001471 | AP2-EREBP | Pathogenesis-related transcriptional factor and ERF |
| Sim4.aln-TCVV015129 | IPR001471 | AP2-EREBP | Pathogenesis-related transcriptional factor and ERF |
| Sim4.aln-TCVV015549 | IPR001471 | AP2-EREBP | Pathogenesis-related transcriptional factor and ERF |
| Sim4.aln-TCVV016109 | IPR001471 | AP2-EREBP | Pathogenesis-related transcriptional factor and ERF |
| Sim4.aln-TCVV016414 | IPR001471 | AP2-EREBP | Pathogenesis-related transcriptional factor and ERF |
| Sim4.aln-TCVV017044 | IPR001471 | AP2-EREBP | Pathogenesis-related transcriptional factor and ERF |
| Sim4.aln-TCVV017601 | IPR001471 | AP2-EREBP | Pathogenesis-related transcriptional factor and ERF |
| Sim4.aln-TCVV019262 | IPR001471 | AP2-EREBP | Pathogenesis-related transcriptional factor and ERF |
| Sim4.aln-TCVV020098 | IPR001471 | AP2-EREBP | Pathogenesis-related transcriptional factor and ERF |
| Sim4.aln-TCVV022226 | IPR001471 | AP2-EREBP | Pathogenesis-related transcriptional factor and ERF |
| Sim4.aln-TCVV022805 | IPR001471 | AP2-EREBP | Pathogenesis-related transcriptional factor and ERF |
| Sim4.aln-TCVV023117 | IPR001471 | AP2-EREBP | Pathogenesis-related transcriptional factor and ERF |
| Sim4.aln-TCVV023283 | IPR001471 | AP2-EREBP | Pathogenesis-related transcriptional factor and ERF |
| Sim4.aln-TCVV023552 | IPR001471 | AP2-EREBP | Pathogenesis-related transcriptional factor and ERF |
| Twinscan1.VV78X016243.17_10 | IPR001471 | AP2-EREBP | Pathogenesis-related transcriptional factor and ERF |
| Twinscan1.VV78X016547.3_1 | IPR001471 | AP2-EREBP | Pathogenesis-related transcriptional factor and ERF |
| Twinscan1.VV78X230296.7_1 | IPR001471 | AP2-EREBP | Pathogenesis-related transcriptional factor and ERF |
| FgenesH.VV78X073333.6_2 | IPR010525 | ARF | Auxin response factor |
| FgenesH.VV78X090426.6_5 | IPR010525 | ARF | Auxin response factor |
| FgenesH.VV78X094999.4_6 | IPR010525 | ARF | Auxin response factor |
| FgenesH.VV78X139764.6_1 | IPR010525 | ARF | Auxin response factor |
| FgenesH.VV78X174339.13_1 | IPR010525 | ARF | Auxin response factor |
| FgenesH.VV78X180358.5_1 | IPR010525 | ARF | Auxin response factor |
| FgenesH.VV78X194429.9_2 | IPR010525 | ARF | Auxin response factor |
| FgenesH.VV78X218047.27_2 | IPR010525 | ARF | Auxin response factor |
| FgenesH.VV78X249697.12_3 | IPR010525 | ARF | Auxin response factor |
| Sim4.aln-A06001809 | IPR003311 | ARF | AUX/IAA protein |
| Sim4.aln-A06003291 | IPR003311 | ARF | AUX/IAA protein |
| Sim4.aln-A06003589 | IPR003311 | ARF | AUX/IAA protein |
| Sim4.aln-TCVV000210 | IPR003311 | ARF | AUX/IAA protein |
| Sim4.aln-TCVV000565 | IPR010525 | ARF | Auxin response factor |
| Sim4.aln-TCVV001557 | IPR003311 | ARF | AUX/IAA protein |
| Sim4.aln-TCVV003666 | IPR003311 | ARF | AUX/IAA protein |
| Sim4.aln-TCVV007142 | IPR003311 | ARF | AUX/IAA protein |
| Sim4.aln-TCVV007546 | IPR010525 | ARF | Auxin response factor |
| Sim4.aln-TCVV012938 | IPR003311 | ARF | AUX/IAA protein |
| Sim4.aln-TCVV016963 | IPR003311 | ARF | AUX/IAA protein |
| Sim4.aln-TCVV018098 | IPR003311 | ARF | AUX/IAA protein |
| Sim4.aln-TCVV018600 | IPR010525 | ARF | Auxin response factor |
| Sim4.aln-TCVV021348 | IPR003311 | ARF | AUX/IAA protein |
| Sim4.aln-TCVV021395 | IPR003311 | ARF | AUX/IAA protein |
| Sim4.aln-TCVV023050 | IPR003311 | ARF | AUX/IAA protein |
| Sim4.aln-TCVV023899 | IPR003311 | ARF | AUX/IAA protein |
| FgenesH.VV78X069434.3_2 | IPR000949 | ARID | ELM2 |
| Glimmer1.VV78X068549.3_1 | IPR001606 | ARID | AT-rich interaction region |
| Glimmer1.VV78X230125.6_2 | IPR001606 | ARID | AT-rich interaction region |
| Twinscan1.VV78X072657.15_1 | IPR001606 | ARID | AT-rich interaction region |
| FgenesH.VV78X001301.9_4 | IPR001789 | ARR-B | Response regulator receiver |
| FgenesH.VV78X027855.12_1 | IPR001789 | ARR-B | Response regulator receiver |
| FgenesH.VV78X039801.10_1 | IPR001789 | ARR-B | Response regulator receiver |
| FgenesH.VV78X044563.10_1 | IPR001789 | ARR-B | Response regulator receiver |
| FgenesH.VV78X044576.17_7 | IPR001789 | ARR-B | Response regulator receiver |
| FgenesH.VV78X045663.7_1 | IPR001789 | ARR-B | Response regulator receiver |
| FgenesH.VV78X059103.7_3 | IPR001789 | ARR-B | Response regulator receiver |
| FgenesH.VV78X069721.21_2 | IPR001789 | ARR-B | Response regulator receiver |
| FgenesH.VV78X099160.8_2 | IPR001789 | ARR-B | Response regulator receiver |
| FgenesH.VV78X128587.3_3 | IPR001789 | ARR-B | Response regulator receiver |
| FgenesH.VV78X139868.2_1 | IPR001789 | ARR-B | Response regulator receiver |
| FgenesH.VV78X169822.14_5 | IPR001789 | ARR-B | Response regulator receiver |
| FgenesH.VV78X179001.3_1 | IPR001789 | ARR-B | Response regulator receiver |
| FgenesH.VV78X211967.26_1 | IPR001789 | ARR-B | Response regulator receiver |
| FgenesH.VV78X230082.3_1 | IPR001789 | ARR-B | Response regulator receiver |
| FgenesH.VV78X246862.18_3 | IPR001789 | ARR-B | Response regulator receiver |
| FgenesH.VV78X265079.5_1 | IPR001789 | ARR-B | Response regulator receiver |
| FgenesH.VV79X002210.3_4 | IPR001789 | ARR-B | Response regulator receiver |
| Sim4.aln-A06017886 | IPR001789 | ARR-B | Response regulator receiver |
| Sim4.aln-TCVV000832 | IPR001789 | ARR-B | Response regulator receiver |
| Sim4.aln-TCVV003939 | IPR001789 | ARR-B | Response regulator receiver |
| Sim4.aln-TCVV008950 | IPR001789 | ARR-B | Response regulator receiver |
| Sim4.aln-TCVV010553 | IPR001789 | ARR-B | Response regulator receiver |
| Sim4.aln-TCVV012430 | IPR001789 | ARR-B | Response regulator receiver |
| Sim4.aln-TCVV014896 | IPR001789 | ARR-B | Response regulator receiver |
| Sim4.aln-TCVV016541 | IPR001789 | ARR-B | Response regulator receiver |
| Sim4.aln-TCVV019936 | IPR001789 | ARR-B | Response regulator receiver |
| Sim4.aln-TCVV022152 | IPR001789 | ARR-B | Response regulator receiver |
| Sim4.aln-TCVV022219 | IPR001789 | ARR-B | Response regulator receiver |
| FgenesH.VV78X014656.31_2 | IPR003311 | AUX/IAA | Aux/IAA protein |
| FgenesH.VV78X027241.4_5 | IPR003311 | AUX/IAA | Aux/IAA protein |
| FgenesH.VV78X074140.10_9 | IPR003311 | AUX/IAA | Aux/IAA protein |
| FgenesH.VV78X076870.9_3 | IPR003311 | AUX/IAA | Aux/IAA protein |
| FgenesH.VV78X116968.10_1 | IPR003311 | AUX/IAA | Aux/IAA protein |
| FgenesH.VV78X148510.10_1 | IPR003311 | AUX/IAA | Aux/IAA protein |
| FgenesH.VV78X166001.4_2 | IPR003311 | AUX/IAA | Aux/IAA protein |
| FgenesH.VV78X179742.7_1 | IPR003311 | AUX/IAA | Aux/IAA protein |
| FgenesH.VV78X193114.7_1 | IPR003311 | AUX/IAA | Aux/IAA protein |
| FgenesH.VV78X194399.8_1 | IPR003311 | AUX/IAA | Aux/IAA protein |
| FgenesH.VV78X221037.4_1 | IPR003311 | AUX/IAA | Aux/IAA protein |
| FgenesH.VV78X254686.13_1 | IPR003311 | AUX/IAA | Aux/IAA protein |
| Sim4.aln-A06000171 | IPR003311 | AUX/IAA | Aux/IAA protein |
| Sim4.aln-A06000258 | IPR003311 | AUX/IAA | Aux/IAA protein |
| Sim4.aln-A06000680 | IPR003311 | AUX/IAA | Aux/IAA protein |
| Sim4.aln-A06002835 | IPR003311 | AUX/IAA | Aux/IAA protein |
| Sim4.aln-A06005493 | IPR003311 | AUX/IAA | Aux/IAA protein |
| Sim4.aln-TCVV000055 | IPR003311 | AUX/IAA | Aux/IAA protein |
| Sim4.aln-TCVV000758 | IPR003311 | AUX/IAA | Aux/IAA protein |
| Sim4.aln-TCVV000976 | IPR003311 | AUX/IAA | Aux/IAA protein |
| Sim4.aln-TCVV007643 | IPR003311 | AUX/IAA | Aux/IAA protein |
| Sim4.aln-TCVV009063 | IPR003311 | AUX/IAA | Aux/IAA protein |
| Sim4.aln-TCVV011851 | IPR003311 | AUX/IAA | Aux/IAA protein |
| Sim4.aln-TCVV012356 | IPR003311 | AUX/IAA | Aux/IAA protein |
| FgenesH.VV78X156050.2_1 | IPR010409 | BBR/BPC | GAGA binding-like |
| Sim4.aln-TCVV001478 | IPR010409 | BBR/BPC | GAGA binding-like |
| Sim4.aln-TCVV002784 | IPR010409 | BBR/BPC | GAGA binding-like |
| Sim4.aln-TCVV004091 | IPR010409 | BBR/BPC | GAGA binding-like |
| FgenesH.VV78X003825.5_2 | IPR008540 | BES1 | BZR1, transcriptional repressor |
| FgenesH.VV78X136529.7_2 | IPR008540 | BES1 | BZR1, transcriptional repressor |
| Sim4.aln-A06014432 | IPR008540 | BES1 | BZR1, transcriptional repressor |
| Sim4.aln-TCVV001157 | IPR008540 | BES1 | BZR1, transcriptional repressor |
| Sim4.aln-TCVV016484 | IPR008540 | BES1 | BZR1, transcriptional repressor |
| FgenesH.VV78X146502.10_1 | IPR001279 | Trihelix | Beta-lactamase-like |
| FgenesH.VV78X200346.29_1 | IPR001279 | Trihelix | Beta-lactamase-like |
| FgenesH.VV78X276734.10_3 | IPR001279 | Trihelix | Beta-lactamase-like |
| FgenesH.VV79X004828.2_1 | IPR001279 | Trihelix | Beta-lactamase-like |
| Sim4.aln-A06016570 | IPR001279 | Trihelix | Beta-lactamase-like |
| Sim4.aln-TCVV000789 | IPR001279 | Trihelix | Beta-lactamase-like |
| Sim4.aln-TCVV011921 | IPR001279 | Trihelix | Beta-lactamase-like |
| Sim4.aln-TCVV014417 | IPR001279 | Trihelix | Beta-lactamase-like |
| Sim4.aln-TCVV015823 | IPR001279 | Trihelix | Beta-lactamase-like |
| FgenesH.VV78X064470.13_4 | IPR011108 | Trihelix | RNA-metabolising metallo-beta-lactamase |
| Sim4.aln-TCVV024200 | IPR011108 | Trihelix | RNA-metabolising metallo-beta-lactamase |
| FgenesH.VV78X014995.11_2 | IPR001092 | bHLH | Basic helix-loop-helix dimerization region bHLH |
| FgenesH.VV78X019759.4_1 | IPR001092 | bHLH | Basic helix-loop-helix dimerization region bHLH |
| FgenesH.VV78X021043.4_1 | IPR001092 | bHLH | Basic helix-loop-helix dimerization region bHLH |
| FgenesH.VV78X029428.17_2 | IPR001092 | bHLH | Basic helix-loop-helix dimerization region bHLH |
| FgenesH.VV78X029948.13_3 | IPR001092 | bHLH | Basic helix-loop-helix dimerization region bHLH |
| FgenesH.VV78X031409.4_4 | IPR001092 | bHLH | Basic helix-loop-helix dimerization region bHLH |
| FgenesH.VV78X037236.8_5 | IPR001092 | bHLH | Basic helix-loop-helix dimerization region bHLH |
| FgenesH.VV78X041585.12_1 | IPR001092 | bHLH | Basic helix-loop-helix dimerization region bHLH |
| FgenesH.VV78X047603.6_1 | IPR001092 | bHLH | Basic helix-loop-helix dimerization region bHLH |
| FgenesH.VV78X054068.9_3 | IPR001092 | bHLH | Basic helix-loop-helix dimerization region bHLH |
| FgenesH.VV78X054508.12_1 | IPR001092 | bHLH | Basic helix-loop-helix dimerization region bHLH |
| FgenesH.VV78X057541.5_4 | IPR001092 | bHLH | Basic helix-loop-helix dimerization region bHLH |
| FgenesH.VV78X058952.10_1 | IPR001092 | bHLH | Basic helix-loop-helix dimerization region bHLH |
| FgenesH.VV78X062106.13_1 | IPR001092 | bHLH | Basic helix-loop-helix dimerization region bHLH |
| FgenesH.VV78X062999.14_7 | IPR001092 | bHLH | Basic helix-loop-helix dimerization region bHLH |
| FgenesH.VV78X063486.6_4 | IPR001092 | bHLH | Basic helix-loop-helix dimerization region bHLH |
| FgenesH.VV78X067085.16_1 | IPR001092 | bHLH | Basic helix-loop-helix dimerization region bHLH |
| FgenesH.VV78X069473.9_5 | IPR001092 | bHLH | Basic helix-loop-helix dimerization region bHLH |
| FgenesH.VV78X071145.5_1 | IPR001092 | bHLH | Basic helix-loop-helix dimerization region bHLH |
| FgenesH.VV78X078136.20_1 | IPR001092 | bHLH | Basic helix-loop-helix dimerization region bHLH |
| FgenesH.VV78X091767.8_2 | IPR001092 | bHLH | Basic helix-loop-helix dimerization region bHLH |
| FgenesH.VV78X094999.4_7 | IPR001092 | bHLH | Basic helix-loop-helix dimerization region bHLH |
| FgenesH.VV78X106184.8_1 | IPR001092 | bHLH | Basic helix-loop-helix dimerization region bHLH |
| FgenesH.VV78X107022.2_1 | IPR001092 | bHLH | Basic helix-loop-helix dimerization region bHLH |
| FgenesH.VV78X107433.10_4 | IPR001092 | bHLH | Basic helix-loop-helix dimerization region bHLH |
| FgenesH.VV78X110477.3_1 | IPR001092 | bHLH | Basic helix-loop-helix dimerization region bHLH |
| FgenesH.VV78X113273.4_2 | IPR001092 | bHLH | Basic helix-loop-helix dimerization region bHLH |
| FgenesH.VV78X113383.10_3 | IPR001092 | bHLH | Basic helix-loop-helix dimerization region bHLH |
| FgenesH.VV78X114155.13_1 | IPR001092 | bHLH | Basic helix-loop-helix dimerization region bHLH |
| FgenesH.VV78X115094.10_1 | IPR001092 | bHLH | Basic helix-loop-helix dimerization region bHLH |
| FgenesH.VV78X116866.29_3 | IPR001092 | bHLH | Basic helix-loop-helix dimerization region bHLH |
| FgenesH.VV78X122741.11_1 | IPR001092 | bHLH | Basic helix-loop-helix dimerization region bHLH |
| FgenesH.VV78X123012.5_2 | IPR001092 | bHLH | Basic helix-loop-helix dimerization region bHLH |
| FgenesH.VV78X123948.6_5 | IPR001092 | bHLH | Basic helix-loop-helix dimerization region bHLH |
| FgenesH.VV78X124349.8_5 | IPR001092 | bHLH | Basic helix-loop-helix dimerization region bHLH |
| FgenesH.VV78X128740.28_1 | IPR001092 | bHLH | Basic helix-loop-helix dimerization region bHLH |
| FgenesH.VV78X132516.5_1 | IPR001092 | bHLH | Basic helix-loop-helix dimerization region bHLH |
| FgenesH.VV78X135624.56_1 | IPR001092 | bHLH | Basic helix-loop-helix dimerization region bHLH |
| FgenesH.VV78X139241.9_3 | IPR001092 | bHLH | Basic helix-loop-helix dimerization region bHLH |
| FgenesH.VV78X141153.17_2 | IPR001092 | bHLH | Basic helix-loop-helix dimerization region bHLH |
| FgenesH.VV78X147890.5_3 | IPR001092 | bHLH | Basic helix-loop-helix dimerization region bHLH |
| FgenesH.VV78X150121.6_6 | IPR001092 | bHLH | Basic helix-loop-helix dimerization region bHLH |
| FgenesH.VV78X150157.8_2 | IPR001092 | bHLH | Basic helix-loop-helix dimerization region bHLH |
| FgenesH.VV78X150703.6_1 | IPR001092 | bHLH | Basic helix-loop-helix dimerization region bHLH |
| FgenesH.VV78X150983.14_1 | IPR001092 | bHLH | Basic helix-loop-helix dimerization region bHLH |
| FgenesH.VV78X151465.9_1 | IPR001092 | bHLH | Basic helix-loop-helix dimerization region bHLH |
| FgenesH.VV78X151465.9_2 | IPR001092 | bHLH | Basic helix-loop-helix dimerization region bHLH |
| FgenesH.VV78X153936.6_1 | IPR001092 | bHLH | Basic helix-loop-helix dimerization region bHLH |
| FgenesH.VV78X155274.6_1 | IPR001092 | bHLH | Basic helix-loop-helix dimerization region bHLH |
| FgenesH.VV78X161040.13_3 | IPR001092 | bHLH | Basic helix-loop-helix dimerization region bHLH |
| FgenesH.VV78X175067.22_2 | IPR001092 | bHLH | Basic helix-loop-helix dimerization region bHLH |
| FgenesH.VV78X175900.11_1 | IPR001092 | bHLH | Basic helix-loop-helix dimerization region bHLH |
| FgenesH.VV78X186880.4_1 | IPR001092 | bHLH | Basic helix-loop-helix dimerization region bHLH |
| FgenesH.VV78X187599.15_2 | IPR001092 | bHLH | Basic helix-loop-helix dimerization region bHLH |
| FgenesH.VV78X201921.11_4 | IPR001092 | bHLH | Basic helix-loop-helix dimerization region bHLH |
| FgenesH.VV78X204536.4_5 | IPR001092 | bHLH | Basic helix-loop-helix dimerization region bHLH |
| FgenesH.VV78X207905.7_1 | IPR001092 | bHLH | Basic helix-loop-helix dimerization region bHLH |
| FgenesH.VV78X212429.8_1 | IPR001092 | bHLH | Basic helix-loop-helix dimerization region bHLH |
| FgenesH.VV78X228516.3_2 | IPR001092 | bHLH | Basic helix-loop-helix dimerization region bHLH |
| FgenesH.VV78X235304.6_1 | IPR001092 | bHLH | Basic helix-loop-helix dimerization region bHLH |
| FgenesH.VV78X250398.2_5 | IPR001092 | bHLH | Basic helix-loop-helix dimerization region bHLH |
| FgenesH.VV78X253703.2_2 | IPR001092 | bHLH | Basic helix-loop-helix dimerization region bHLH |
| FgenesH.VV78X253760.24_1 | IPR001092 | bHLH | Basic helix-loop-helix dimerization region bHLH |
| FgenesH.VV78X256440.3_6 | IPR001092 | bHLH | Basic helix-loop-helix dimerization region bHLH |
| FgenesH.VV78X259626.9_1 | IPR001092 | bHLH | Basic helix-loop-helix dimerization region bHLH |
| FgenesH.VV78X260730.14_3 | IPR001092 | bHLH | Basic helix-loop-helix dimerization region bHLH |
| FgenesH.VV78X262061.6_2 | IPR001092 | bHLH | Basic helix-loop-helix dimerization region bHLH |
| FgenesH.VV78X270712.13_1 | IPR001092 | bHLH | Basic helix-loop-helix dimerization region bHLH |
| FgenesH.VV78X277767.9_3 | IPR001092 | bHLH | Basic helix-loop-helix dimerization region bHLH |
| FgenesH.VV78X278653.3_1 | IPR001092 | bHLH | Basic helix-loop-helix dimerization region bHLH |
| FgenesH.VV79X001348.2_3 | IPR001092 | bHLH | Basic helix-loop-helix dimerization region bHLH |
| FgenesH.VV79X001897.2_1 | IPR001092 | bHLH | Basic helix-loop-helix dimerization region bHLH |
| FgenesH.VV79X003355.3_1 | IPR001092 | bHLH | Basic helix-loop-helix dimerization region bHLH |
| Glimmer1.VV78X064336.4_1 | IPR001092 | bHLH | Basic helix-loop-helix dimerization region bHLH |
| Glimmer1.VV78X159768.13_1 | IPR001092 | bHLH | Basic helix-loop-helix dimerization region bHLH |
| Glimmer1.VV78X177465.5_2 | IPR001092 | bHLH | Basic helix-loop-helix dimerization region bHLH |
| Glimmer1.VV78X200353.10_2 | IPR001092 | bHLH | Basic helix-loop-helix dimerization region bHLH |
| Sim4.aln-A06001488 | IPR001092 | bHLH | Basic helix-loop-helix dimerization region bHLH |
| Sim4.aln-A06002339 | IPR001092 | bHLH | Basic helix-loop-helix dimerization region bHLH |
| Sim4.aln-A06002868 | IPR001092 | bHLH | Basic helix-loop-helix dimerization region bHLH |
| Sim4.aln-A06005004 | IPR001092 | bHLH | Basic helix-loop-helix dimerization region bHLH |
| Sim4.aln-A06008509 | IPR001092 | bHLH | Basic helix-loop-helix dimerization region bHLH |
| Sim4.aln-A06009305 | IPR001092 | bHLH | Basic helix-loop-helix dimerization region bHLH |
| Sim4.aln-A06010289 | IPR001092 | bHLH | Basic helix-loop-helix dimerization region bHLH |
| Sim4.aln-A06010345 | IPR001092 | bHLH | Basic helix-loop-helix dimerization region bHLH |
| Sim4.aln-A06010407 | IPR001092 | bHLH | Basic helix-loop-helix dimerization region bHLH |
| Sim4.aln-A06012974 | IPR001092 | bHLH | Basic helix-loop-helix dimerization region bHLH |
| Sim4.aln-A06013287 | IPR001092 | bHLH | Basic helix-loop-helix dimerization region bHLH |
| Sim4.aln-A06017281 | IPR001092 | bHLH | Basic helix-loop-helix dimerization region bHLH |
| Sim4.aln-A06017369 | IPR001092 | bHLH | Basic helix-loop-helix dimerization region bHLH |
| Sim4.aln-A06018147 | IPR001092 | bHLH | Basic helix-loop-helix dimerization region bHLH |
| Sim4.aln-A06018180 | IPR001092 | bHLH | Basic helix-loop-helix dimerization region bHLH |
| Sim4.aln-A06019281 | IPR001092 | bHLH | Basic helix-loop-helix dimerization region bHLH |
| Sim4.aln-TCVV000457 | IPR001092 | bHLH | Basic helix-loop-helix dimerization region bHLH |
| Sim4.aln-TCVV000528 | IPR001092 | bHLH | Basic helix-loop-helix dimerization region bHLH |
| Sim4.aln-TCVV000610 | IPR001092 | bHLH | Basic helix-loop-helix dimerization region bHLH |
| Sim4.aln-TCVV000825 | IPR001092 | bHLH | Basic helix-loop-helix dimerization region bHLH |
| Sim4.aln-TCVV001463 | IPR001092 | bHLH | Basic helix-loop-helix dimerization region bHLH |
| Sim4.aln-TCVV001935 | IPR001092 | bHLH | Basic helix-loop-helix dimerization region bHLH |
| Sim4.aln-TCVV002202 | IPR001092 | bHLH | Basic helix-loop-helix dimerization region bHLH |
| Sim4.aln-TCVV002930 | IPR001092 | bHLH | Basic helix-loop-helix dimerization region bHLH |
| Sim4.aln-TCVV004332 | IPR001092 | bHLH | Basic helix-loop-helix dimerization region bHLH |
| Sim4.aln-TCVV004456 | IPR001092 | bHLH | Basic helix-loop-helix dimerization region bHLH |
| Sim4.aln-TCVV004944 | IPR001092 | bHLH | Basic helix-loop-helix dimerization region bHLH |
| Sim4.aln-TCVV005186 | IPR001092 | bHLH | Basic helix-loop-helix dimerization region bHLH |
| Sim4.aln-TCVV005574 | IPR001092 | bHLH | Basic helix-loop-helix dimerization region bHLH |
| Sim4.aln-TCVV007080 | IPR001092 | bHLH | Basic helix-loop-helix dimerization region bHLH |
| Sim4.aln-TCVV007418 | IPR001092 | bHLH | Basic helix-loop-helix dimerization region bHLH |
| Sim4.aln-TCVV007632 | IPR001092 | bHLH | Basic helix-loop-helix dimerization region bHLH |
| Sim4.aln-TCVV008191 | IPR001092 | bHLH | Basic helix-loop-helix dimerization region bHLH |
| Sim4.aln-TCVV008751 | IPR001092 | bHLH | Basic helix-loop-helix dimerization region bHLH |
| Sim4.aln-TCVV008775 | IPR001092 | bHLH | Basic helix-loop-helix dimerization region bHLH |
| Sim4.aln-TCVV008872 | IPR001092 | bHLH | Basic helix-loop-helix dimerization region bHLH |
| Sim4.aln-TCVV008932 | IPR001092 | bHLH | Basic helix-loop-helix dimerization region bHLH |
| Sim4.aln-TCVV009265 | IPR001092 | bHLH | Basic helix-loop-helix dimerization region bHLH |
| Sim4.aln-TCVV009284 | IPR001092 | bHLH | Basic helix-loop-helix dimerization region bHLH |
| Sim4.aln-TCVV009444 | IPR001092 | bHLH | Basic helix-loop-helix dimerization region bHLH |
| Sim4.aln-TCVV010252 | IPR001092 | bHLH | Basic helix-loop-helix dimerization region bHLH |
| Sim4.aln-TCVV010879 | IPR001092 | bHLH | Basic helix-loop-helix dimerization region bHLH |
| Sim4.aln-TCVV012015 | IPR001092 | bHLH | Basic helix-loop-helix dimerization region bHLH |
| Sim4.aln-TCVV012750 | IPR001092 | bHLH | Basic helix-loop-helix dimerization region bHLH |
| Sim4.aln-TCVV013587 | IPR001092 | bHLH | Basic helix-loop-helix dimerization region bHLH |
| Sim4.aln-TCVV014615 | IPR001092 | bHLH | Basic helix-loop-helix dimerization region bHLH |
| Sim4.aln-TCVV014804 | IPR001092 | bHLH | Basic helix-loop-helix dimerization region bHLH |
| Sim4.aln-TCVV015971 | IPR001092 | bHLH | Basic helix-loop-helix dimerization region bHLH |
| Sim4.aln-TCVV016068 | IPR001092 | bHLH | Basic helix-loop-helix dimerization region bHLH |
| Sim4.aln-TCVV016619 | IPR001092 | bHLH | Basic helix-loop-helix dimerization region bHLH |
| Sim4.aln-TCVV018198 | IPR001092 | bHLH | Basic helix-loop-helix dimerization region bHLH |
| Sim4.aln-TCVV021175 | IPR001092 | bHLH | Basic helix-loop-helix dimerization region bHLH |
| Sim4.aln-TCVV021246 | IPR001092 | bHLH | Basic helix-loop-helix dimerization region bHLH |
| Sim4.aln-TCVV021732 | IPR001092 | bHLH | Basic helix-loop-helix dimerization region bHLH |
| Sim4.aln-TCVV021991 | IPR001092 | bHLH | Basic helix-loop-helix dimerization region bHLH |
| Sim4.aln-TCVV022119 | IPR001092 | bHLH | Basic helix-loop-helix dimerization region bHLH |
| Sim4.aln-TCVV022164 | IPR001092 | bHLH | Basic helix-loop-helix dimerization region bHLH |
| Sim4.aln-TCVV022222 | IPR001092 | bHLH | Basic helix-loop-helix dimerization region bHLH |
| Sim4.aln-TCVV022861 | IPR001092 | bHLH | Basic helix-loop-helix dimerization region bHLH |
| Sim4.aln-TCVV024248 | IPR001092 | bHLH | Basic helix-loop-helix dimerization region bHLH |
| Twinscan1.VV78X043971.11_7 | IPR001092 | bHLH | Basic helix-loop-helix dimerization region bHLH |
| Twinscan1.VV78X081674.5_2 | IPR001092 | bHLH | Basic helix-loop-helix dimerization region bHLH |
| Twinscan1.VV78X136286.3_1 | IPR001092 | bHLH | Basic helix-loop-helix dimerization region bHLH |
| Twinscan1.VV78X194073.4_2 | IPR001092 | bHLH | Basic helix-loop-helix dimerization region bHLH |
| Twinscan1.VV78X233722.8_1 | IPR001092 | bHLH | Basic helix-loop-helix dimerization region bHLH |
| FgenesH.VV78X000641.11_1 | IPR011616 | bZIP | bZIP transcription factor, bZIP_1 |
| FgenesH.VV78X000824.7_1 | IPR011616 | bZIP | bZIP transcription factor, bZIP_1 |
| FgenesH.VV78X009646.11_1 | IPR011616 | bZIP | bZIP transcription factor, bZIP_1 |
| FgenesH.VV78X012470.5_2 | IPR011616 | bZIP | bZIP transcription factor, bZIP_1 |
| FgenesH.VV78X027343.7_4 | IPR011616 | bZIP | bZIP transcription factor, bZIP_1 |
| FgenesH.VV78X030262.6_1 | IPR004827 | bZIP | Basic-leucine zipper (bZIP) transcription factor |
| FgenesH.VV78X035471.3_2 | IPR011700 | bZIP | Basic leucine zipper |
| FgenesH.VV78X045078.13_2 | IPR011616 | bZIP | bZIP transcription factor, bZIP_1 |
| FgenesH.VV78X050398.8_1 | IPR011616 | bZIP | bZIP transcription factor, bZIP_1 |
| FgenesH.VV78X050398.8_2 | IPR011616 | bZIP | bZIP transcription factor, bZIP_1 |
| FgenesH.VV78X053765.4_1 | IPR004827 | bZIP | Basic-leucine zipper (bZIP) transcription factor |
| FgenesH.VV78X053933.4_2 | IPR011616 | bZIP | bZIP transcription factor, bZIP_1 |
| FgenesH.VV78X054945.11_2 | IPR011616 | bZIP | bZIP transcription factor, bZIP_1 |
| FgenesH.VV78X058501.7_1 | IPR011616 | bZIP | bZIP transcription factor, bZIP_1 |
| FgenesH.VV78X063138.9_3 | IPR011616 | bZIP | bZIP transcription factor, bZIP_1 |
| FgenesH.VV78X066969.8_2 | IPR011616 | bZIP | bZIP transcription factor, bZIP_1 |
| FgenesH.VV78X067114.4_2 | IPR011616 | bZIP | bZIP transcription factor, bZIP_1 |
| FgenesH.VV78X067972.26_3 | IPR004827 | bZIP | Basic-leucine zipper (bZIP) transcription factor |
| FgenesH.VV78X069977.11_2 | IPR011616 | bZIP | bZIP transcription factor, bZIP_1 |
| FgenesH.VV78X071755.11_1 | IPR011616 | bZIP | bZIP transcription factor, bZIP_1 |
| FgenesH.VV78X077429.7_1 | IPR011616 | bZIP | bZIP transcription factor, bZIP_1 |
| FgenesH.VV78X082964.7_1 | IPR004827 | bZIP | Basic-leucine zipper (bZIP) transcription factor |
| FgenesH.VV78X091232.6_3 | IPR011616 | bZIP | bZIP transcription factor, bZIP_1 |
| FgenesH.VV78X091414.6_3 | IPR004827 | bZIP | Basic-leucine zipper (bZIP) transcription factor |
| FgenesH.VV78X093827.5_1 | IPR011616 | bZIP | bZIP transcription factor, bZIP_1 |
| FgenesH.VV78X100436.13_1 | IPR011616 | bZIP | bZIP transcription factor, bZIP_1 |
| FgenesH.VV78X105368.14_10 | IPR011616 | bZIP | bZIP transcription factor, bZIP_1 |
| FgenesH.VV78X109895.7_4 | IPR011616 | bZIP | bZIP transcription factor, bZIP_1 |
| FgenesH.VV78X111726.11_6 | IPR011616 | bZIP | bZIP transcription factor, bZIP_1 |
| FgenesH.VV78X113416.19_1 | IPR011616 | bZIP | bZIP transcription factor, bZIP_1 |
| FgenesH.VV78X115359.4_1 | IPR011616 | bZIP | bZIP transcription factor, bZIP_1 |
| FgenesH.VV78X115539.4_1 | IPR011616 | bZIP | bZIP transcription factor, bZIP_1 |
| FgenesH.VV78X115722.8_4 | IPR004827 | bZIP | Basic-leucine zipper (bZIP) transcription factor |
| FgenesH.VV78X117635.8_1 | IPR011616 | bZIP | bZIP transcription factor, bZIP_1 |
| FgenesH.VV78X119118.16_2 | IPR011616 | bZIP | bZIP transcription factor, bZIP_1 |
| FgenesH.VV78X124966.6_2 | IPR011616 | bZIP | bZIP transcription factor, bZIP_1 |
| FgenesH.VV78X132344.3_1 | IPR011616 | bZIP | bZIP transcription factor, bZIP_1 |
| FgenesH.VV78X132404.9_5 | IPR011616 | bZIP | bZIP transcription factor, bZIP_1 |
| FgenesH.VV78X133702.19_3 | IPR011616 | bZIP | bZIP transcription factor, bZIP_1 |
| FgenesH.VV78X133795.13_7 | IPR011700 | bZIP | Basic leucine zipper |
| FgenesH.VV78X134315.9_6 | IPR011616 | bZIP | bZIP transcription factor, bZIP_1 |
| FgenesH.VV78X134383.5_1 | IPR004827 | bZIP | Basic-leucine zipper (bZIP) transcription factor |
| FgenesH.VV78X136296.10_2 | IPR011616 | bZIP | bZIP transcription factor, bZIP_1 |
| FgenesH.VV78X136581.9_2 | IPR004827 | bZIP | Basic-leucine zipper (bZIP) transcription factor |
| FgenesH.VV78X140622.6_1 | IPR011616 | bZIP | bZIP transcription factor, bZIP_1 |
| FgenesH.VV78X150121.6_4 | IPR011700 | bZIP | Basic leucine zipper |
| FgenesH.VV78X150580.50_2 | IPR011616 | bZIP | bZIP transcription factor, bZIP_1 |
| FgenesH.VV78X153336.4_2 | IPR011616 | bZIP | bZIP transcription factor, bZIP_1 |
| FgenesH.VV78X154954.16_4 | IPR011616 | bZIP | bZIP transcription factor, bZIP_1 |
| FgenesH.VV78X157626.10_1 | IPR011616 | bZIP | bZIP transcription factor, bZIP_1 |
| FgenesH.VV78X159123.8_2 | IPR011616 | bZIP | bZIP transcription factor, bZIP_1 |
| FgenesH.VV78X166060.9_1 | IPR011616 | bZIP | bZIP transcription factor, bZIP_1 |
| FgenesH.VV78X167880.8_2 | IPR011616 | bZIP | bZIP transcription factor, bZIP_1 |
| FgenesH.VV78X175150.2_4 | IPR011616 | bZIP | bZIP transcription factor, bZIP_1 |
| FgenesH.VV78X190904.10_2 | IPR011616 | bZIP | bZIP transcription factor, bZIP_1 |
| FgenesH.VV78X195339.37_1 | IPR011616 | bZIP | bZIP transcription factor, bZIP_1 |
| FgenesH.VV78X199623.7_2 | IPR011616 | bZIP | bZIP transcription factor, bZIP_1 |
| FgenesH.VV78X201757.19_1 | IPR011616 | bZIP | bZIP transcription factor, bZIP_1 |
| FgenesH.VV78X203041.4_3 | IPR011700 | bZIP | Basic leucine zipper |
| FgenesH.VV78X205240.10_3 | IPR011616 | bZIP | bZIP transcription factor, bZIP_1 |
| FgenesH.VV78X217549.24_2 | IPR011616 | bZIP | bZIP transcription factor, bZIP_1 |
| FgenesH.VV78X222065.21_5 | IPR011616 | bZIP | bZIP transcription factor, bZIP_1 |
| FgenesH.VV78X222494.4_1 | IPR004827 | bZIP | Basic-leucine zipper (bZIP) transcription factor |
| FgenesH.VV78X227435.3_1 | IPR011616 | bZIP | bZIP transcription factor, bZIP_1 |
| FgenesH.VV78X234661.5_2 | IPR011616 | bZIP | bZIP transcription factor, bZIP_1 |
| FgenesH.VV78X244647.5_1 | IPR011700 | bZIP | Basic leucine zipper |
| FgenesH.VV78X246316.7_1 | IPR011616 | bZIP | bZIP transcription factor, bZIP_1 |
| FgenesH.VV78X249627.4_1 | IPR011616 | bZIP | bZIP transcription factor, bZIP_1 |
| FgenesH.VV78X255602.8_6 | IPR004827 | bZIP | Basic-leucine zipper (bZIP) transcription factor |
| FgenesH.VV78X261958.8_4 | IPR011616 | bZIP | bZIP transcription factor, bZIP_1 |
| FgenesH.VV78X263815.34_4 | IPR004827 | bZIP | Basic-leucine zipper (bZIP) transcription factor |
| FgenesH.VV78X266596.7_3 | IPR011616 | bZIP | bZIP transcription factor, bZIP_1 |
| FgenesH.VV78X268410.8_2 | IPR004827 | bZIP | Basic-leucine zipper (bZIP) transcription factor |
| FgenesH.VV78X268761.4_2 | IPR011616 | bZIP | bZIP transcription factor, bZIP_1 |
| FgenesH.VV78X271304.8_1 | IPR011616 | bZIP | bZIP transcription factor, bZIP_1 |
| FgenesH.VV78X277202.14_4 | IPR011616 | bZIP | bZIP transcription factor, bZIP_1 |
| Glimmer1.VV78X022361.9_1 | IPR011616 | bZIP | bZIP transcription factor, bZIP_1 |
| Glimmer1.VV78X039579.5_1 | IPR004827 | bZIP | Basic-leucine zipper (bZIP) transcription factor |
| Glimmer1.VV78X074101.6_1 | IPR011616 | bZIP | bZIP transcription factor, bZIP_1 |
| Glimmer1.VV78X179937.10_4 | IPR011616 | bZIP | bZIP transcription factor, bZIP_1 |
| Glimmer1.VV78X202146.15_4 | IPR011616 | bZIP | bZIP transcription factor, bZIP_1 |
| Glimmer1.VV78X207570.7_1 | IPR011616 | bZIP | bZIP transcription factor, bZIP_1 |
| Glimmer1.VV78X255783.8_7 | IPR011616 | bZIP | bZIP transcription factor, bZIP_1 |
| Sim4.aln-A06012801 | IPR011616 | bZIP | bZIP transcription factor, bZIP_1 |
| Sim4.aln-A06013470 | IPR011616 | bZIP | bZIP transcription factor, bZIP_1 |
| Sim4.aln-A06016802 | IPR011616 | bZIP | bZIP transcription factor, bZIP_1 |
| Sim4.aln-A06017265 | IPR011700 | bZIP | Basic leucine zipper |
| Sim4.aln-A06017605 | IPR004827 | bZIP | Basic-leucine zipper (bZIP) transcription factor |
| Sim4.aln-A06018412 | IPR011616 | bZIP | bZIP transcription factor, bZIP_1 |
| Sim4.aln-A06019527 | IPR004827 | bZIP | Basic-leucine zipper (bZIP) transcription factor |
| Sim4.aln-TCVV000118 | IPR004827 | bZIP | Basic-leucine zipper (bZIP) transcription factor |
| Sim4.aln-TCVV000280 | IPR004827 | bZIP | Basic-leucine zipper (bZIP) transcription factor |
| Sim4.aln-TCVV000558 | IPR011700 | bZIP | Basic leucine zipper |
| Sim4.aln-TCVV000706 | IPR004827 | bZIP | Basic-leucine zipper (bZIP) transcription factor |
| Sim4.aln-TCVV000959 | IPR012900 | bZIP | G-box binding, MFMR |
| Sim4.aln-TCVV001573 | IPR004827 | bZIP | Basic-leucine zipper (bZIP) transcription factor |
| Sim4.aln-TCVV002675 | IPR004827 | bZIP | Basic-leucine zipper (bZIP) transcription factor |
| Sim4.aln-TCVV002842 | IPR011616 | bZIP | bZIP transcription factor, bZIP_1 |
| Sim4.aln-TCVV003792 | IPR011616 | bZIP | bZIP transcription factor, bZIP_1 |
| Sim4.aln-TCVV003825 | IPR004827 | bZIP | Basic-leucine zipper (bZIP) transcription factor |
| Sim4.aln-TCVV003978 | IPR011616 | bZIP | bZIP transcription factor, bZIP_1 |
| Sim4.aln-TCVV004155 | IPR011700 | bZIP | Basic leucine zipper |
| Sim4.aln-TCVV004570 | IPR004827 | bZIP | Basic-leucine zipper (bZIP) transcription factor |
| Sim4.aln-TCVV004623 | IPR004827 | bZIP | Basic-leucine zipper (bZIP) transcription factor |
| Sim4.aln-TCVV004796 | IPR011616 | bZIP | bZIP transcription factor, bZIP_1 |
| Sim4.aln-TCVV004855 | IPR011616 | bZIP | bZIP transcription factor, bZIP_1 |
| Sim4.aln-TCVV005161 | IPR011616 | bZIP | bZIP transcription factor, bZIP_1 |
| Sim4.aln-TCVV005494 | IPR011700 | bZIP | Basic leucine zipper |
| Sim4.aln-TCVV005896 | IPR011700 | bZIP | Basic leucine zipper |
| Sim4.aln-TCVV005900 | IPR011616 | bZIP | bZIP transcription factor, bZIP_1 |
| Sim4.aln-TCVV006385 | IPR011616 | bZIP | bZIP transcription factor, bZIP_1 |
| Sim4.aln-TCVV006451 | IPR011616 | bZIP | bZIP transcription factor, bZIP_1 |
| Sim4.aln-TCVV006547 | IPR011616 | bZIP | bZIP transcription factor, bZIP_1 |
| Sim4.aln-TCVV006608 | IPR011700 | bZIP | Basic leucine zipper |
| Sim4.aln-TCVV006629 | IPR011616 | bZIP | bZIP transcription factor, bZIP_1 |
| Sim4.aln-TCVV007599 | IPR004827 | bZIP | Basic-leucine zipper (bZIP) transcription factor |
| Sim4.aln-TCVV007738 | IPR011700 | bZIP | Basic leucine zipper |
| Sim4.aln-TCVV007790 | IPR004827 | bZIP | Basic-leucine zipper (bZIP) transcription factor |
| Sim4.aln-TCVV007809 | IPR004827 | bZIP | Basic-leucine zipper (bZIP) transcription factor |
| Sim4.aln-TCVV008902 | IPR011616 | bZIP | bZIP transcription factor, bZIP_1 |
| Sim4.aln-TCVV010119 | IPR011616 | bZIP | bZIP transcription factor, bZIP_1 |
| Sim4.aln-TCVV011800 | IPR011616 | bZIP | bZIP transcription factor, bZIP_1 |
| Sim4.aln-TCVV011888 | IPR011616 | bZIP | bZIP transcription factor, bZIP_1 |
| Sim4.aln-TCVV012729 | IPR004827 | bZIP | Basic-leucine zipper (bZIP) transcription factor |
| Sim4.aln-TCVV013852 | IPR011616 | bZIP | bZIP transcription factor, bZIP_1 |
| Sim4.aln-TCVV013896 | IPR011616 | bZIP | bZIP transcription factor, bZIP_1 |
| Sim4.aln-TCVV014608 | IPR011616 | bZIP | bZIP transcription factor, bZIP_1 |
| Sim4.aln-TCVV014687 | IPR011616 | bZIP | bZIP transcription factor, bZIP_1 |
| Sim4.aln-TCVV014920 | IPR004827 | bZIP | Basic-leucine zipper (bZIP) transcription factor |
| Sim4.aln-TCVV015253 | IPR011616 | bZIP | bZIP transcription factor, bZIP_1 |
| Sim4.aln-TCVV016857 | IPR011616 | bZIP | bZIP transcription factor, bZIP_1 |
| Sim4.aln-TCVV016942 | IPR011616 | bZIP | bZIP transcription factor, bZIP_1 |
| Sim4.aln-TCVV017069 | IPR011616 | bZIP | bZIP transcription factor, bZIP_1 |
| Sim4.aln-TCVV019314 | IPR012900 | bZIP | G-box binding, MFMR |
| Sim4.aln-TCVV019665 | IPR004827 | bZIP | Basic-leucine zipper (bZIP) transcription factor |
| Sim4.aln-TCVV023377 | IPR004827 | bZIP | Basic-leucine zipper (bZIP) transcription factor |
| Sim4.aln-TCVV023741 | IPR011616 | bZIP | bZIP transcription factor, bZIP_1 |
| Sim4.aln-TCVV023983 | IPR011616 | bZIP | bZIP transcription factor, bZIP_1 |
| Twinscan1.VV78X019152.8_10 | IPR011616 | bZIP | bZIP transcription factor, bZIP_1 |
| Twinscan1.VV78X026377.9_2 | IPR011616 | bZIP | bZIP transcription factor, bZIP_1 |
| Twinscan1.VV78X051051.14_8 | IPR004827 | bZIP | Basic-leucine zipper (bZIP) transcription factor |
| Twinscan1.VV78X195460.13_3 | IPR011616 | bZIP | bZIP transcription factor, bZIP_1 |
| Twinscan1.VV78X277820.7_1 | IPR004827 | bZIP | Basic-leucine zipper (bZIP) transcription factor |
| FgenesH.VV78X088054.4_2 | IPR010402 | C2C2-CO-like | CCT |
| FgenesH.VV78X093984.17_5 | IPR010402 | C2C2-CO-like | CCT |
| FgenesH.VV78X130502.3_3 | IPR010402 | C2C2-CO-like | CCT |
| FgenesH.VV78X194150.19_1 | IPR010402 | C2C2-CO-like | CCT |
| FgenesH.VV78X230082.3_2 | IPR010402 | C2C2-CO-like | CCT |
| FgenesH.VV78X255783.8_1 | IPR010402 | C2C2-CO-like | CCT |
| FgenesH.VV79X002210.3_3 | IPR010402 | C2C2-CO-like | CCT |
| Sim4.aln-A06009525 | IPR010402 | C2C2-CO-like | CCT |
| Sim4.aln-A06017850 | IPR010402 | C2C2-CO-like | CCT |
| Sim4.aln-TCVV000946 | IPR010402 | C2C2-CO-like | CCT |
| Sim4.aln-TCVV002070 | IPR010402 | C2C2-CO-like | CCT |
| Sim4.aln-TCVV002325 | IPR010402 | C2C2-CO-like | CCT |
| Sim4.aln-TCVV005481 | IPR010402 | C2C2-CO-like | CCT |
| Sim4.aln-TCVV007123 | IPR010402 | C2C2-CO-like | CCT |
| Sim4.aln-TCVV008188 | IPR010402 | C2C2-CO-like | CCT |
| Sim4.aln-TCVV008443 | IPR010402 | C2C2-CO-like | CCT |
| Sim4.aln-TCVV013106 | IPR010402 | C2C2-CO-like | CCT |
| Sim4.aln-TCVV017781 | IPR010402 | C2C2-CO-like | CCT |
| Sim4.aln-TCVV022392 | IPR010402 | C2C2-CO-like | CCT |
| Sim4.aln-TCVV023129 | IPR010402 | C2C2-CO-like | CCT |
| Sim4.aln-TCVV023198 | IPR010402 | C2C2-CO-like | CCT |
| FgenesH.VV78X015400.11_2 | IPR003851 | C2C2-Dof | Zinc finger, Dof-type |
| FgenesH.VV78X039063.5_3 | IPR003851 | C2C2-Dof | Zinc finger, Dof-type |
| FgenesH.VV78X076016.5_1 | IPR003851 | C2C2-Dof | Zinc finger, Dof-type |
| FgenesH.VV78X088874.5_1 | IPR003851 | C2C2-Dof | Zinc finger, Dof-type |
| FgenesH.VV78X089476.28_1 | IPR003851 | C2C2-Dof | Zinc finger, Dof-type |
| FgenesH.VV78X095025.7_2 | IPR003851 | C2C2-Dof | Zinc finger, Dof-type |
| FgenesH.VV78X135580.13_1 | IPR003851 | C2C2-Dof | Zinc finger, Dof-type |
| FgenesH.VV78X138031.14_2 | IPR003851 | C2C2-Dof | Zinc finger, Dof-type |
| FgenesH.VV78X166878.4_3 | IPR003851 | C2C2-Dof | Zinc finger, Dof-type |
| FgenesH.VV78X180438.8_1 | IPR003851 | C2C2-Dof | Zinc finger, Dof-type |
| FgenesH.VV78X263908.20_4 | IPR003851 | C2C2-Dof | Zinc finger, Dof-type |
| Glimmer1.VV78X017201.5_4 | IPR003851 | C2C2-Dof | Zinc finger, Dof-type |
| Glimmer1.VV78X037628.5_1 | IPR003851 | C2C2-Dof | Zinc finger, Dof-type |
| Glimmer1.VV78X179258.2_2 | IPR003851 | C2C2-Dof | Zinc finger, Dof-type |
| Glimmer1.VV78X236115.6_3 | IPR003851 | C2C2-Dof | Zinc finger, Dof-type |
| Glimmer1.VV78X261535.7_6 | IPR003851 | C2C2-Dof | Zinc finger, Dof-type |
| Sim4.aln-A06001976 | IPR003851 | C2C2-Dof | Zinc finger, Dof-type |
| Sim4.aln-A06009622 | IPR003851 | C2C2-Dof | Zinc finger, Dof-type |
| Sim4.aln-A06018049 | IPR003851 | C2C2-Dof | Zinc finger, Dof-type |
| Sim4.aln-TCVV003091 | IPR003851 | C2C2-Dof | Zinc finger, Dof-type |
| Sim4.aln-TCVV005657 | IPR003851 | C2C2-Dof | Zinc finger, Dof-type |
| Sim4.aln-TCVV007453 | IPR003851 | C2C2-Dof | Zinc finger, Dof-type |
| Sim4.aln-TCVV009332 | IPR003851 | C2C2-Dof | Zinc finger, Dof-type |
| Sim4.aln-TCVV010534 | IPR003851 | C2C2-Dof | Zinc finger, Dof-type |
| Sim4.aln-TCVV016563 | IPR003851 | C2C2-Dof | Zinc finger, Dof-type |
| Sim4.aln-TCVV020751 | IPR003851 | C2C2-Dof | Zinc finger, Dof-type |
| FgenesH.VV78X007757.13_4 | IPR000679 | C2C2-GATA | Zinc finger, GATA-type |
| FgenesH.VV78X053970.8_2 | IPR000679 | C2C2-GATA | Zinc finger, GATA-type |
| FgenesH.VV78X090426.6_4 | IPR000679 | C2C2-GATA | Zinc finger, GATA-type |
| FgenesH.VV78X168410.7_6 | IPR000679 | C2C2-GATA | Zinc finger, GATA-type |
| FgenesH.VV78X191569.25_2 | IPR000679 | C2C2-GATA | Zinc finger, GATA-type |
| FgenesH.VV78X216764.4_1 | IPR000679 | C2C2-GATA | Zinc finger, GATA-type |
| FgenesH.VV78X221945.9_1 | IPR000679 | C2C2-GATA | Zinc finger, GATA-type |
| FgenesH.VV78X242028.10_2 | IPR000679 | C2C2-GATA | Zinc finger, GATA-type |
| FgenesH.VV78X278779.4_1 | IPR000679 | C2C2-GATA | Zinc finger, GATA-type |
| Glimmer1.VV79X001623.2_2 | IPR000679 | C2C2-GATA | Zinc finger, GATA-type |
| Sim4.aln-A06016958 | IPR000679 | C2C2-GATA | Zinc finger, GATA-type |
| Sim4.aln-TCVV009200 | IPR000679 | C2C2-GATA | Zinc finger, GATA-type |
| Sim4.aln-TCVV012249 | IPR000679 | C2C2-GATA | Zinc finger, GATA-type |
| Sim4.aln-TCVV014872 | IPR000679 | C2C2-GATA | Zinc finger, GATA-type |
| Sim4.aln-TCVV015409 | IPR000679 | C2C2-GATA | Zinc finger, GATA-type |
| Sim4.aln-TCVV023040 | IPR000679 | C2C2-GATA | Zinc finger, GATA-type |
| Sim4.aln-TCVV023480 | IPR000679 | C2C2-GATA | Zinc finger, GATA-type |
| Sim4.aln-TCVV023936 | IPR000679 | C2C2-GATA | Zinc finger, GATA-type |
| FgenesH.VV78X023826.39_1 | IPR006780 | C2C2-YABBY | YABBY protein |
| Sim4.aln-TCVV000208 | IPR006780 | C2C2-YABBY | YABBY protein |
| Sim4.aln-TCVV009319 | IPR006780 | C2C2-YABBY | YABBY protein |
| Sim4.aln-TCVV022766 | IPR006780 | C2C2-YABBY | YABBY protein |
| Sim4.aln-TCVV022783 | IPR006780 | C2C2-YABBY | YABBY protein |
| FgenesH.VV78X019093.5_2 | IPR007087 | C2H2 | Zinc finger, C2H2-type |
| FgenesH.VV78X044576.17_1 | IPR007087 | C2H2 | Zinc finger, C2H2-type |
| FgenesH.VV78X044773.12_1 | IPR007087 | C2H2 | Zinc finger, C2H2-type |
| FgenesH.VV78X050744.10_2 | IPR007087 | C2H2 | Zinc finger, C2H2-type |
| FgenesH.VV78X054944.2_2 | IPR007087 | C2H2 | Zinc finger, C2H2-type |
| FgenesH.VV78X055432.12_2 | IPR007087 | C2H2 | Zinc finger, C2H2-type |
| FgenesH.VV78X061842.5_2 | IPR007087 | C2H2 | Zinc finger, C2H2-type |
| FgenesH.VV78X063132.15_3 | IPR007087 | C2H2 | Zinc finger, C2H2-type |
| FgenesH.VV78X063445.19_2 | IPR007087 | C2H2 | Zinc finger, C2H2-type |
| FgenesH.VV78X070138.9_1 | IPR007087 | C2H2 | Zinc finger, C2H2-type |
| FgenesH.VV78X071556.23_1 | IPR007087 | C2H2 | Zinc finger, C2H2-type |
| FgenesH.VV78X073310.28_3 | IPR007087 | C2H2 | Zinc finger, C2H2-type |
| FgenesH.VV78X077459.37_1 | IPR007087 | C2H2 | Zinc finger, C2H2-type |
| FgenesH.VV78X078964.16_1 | IPR007087 | C2H2 | Zinc finger, C2H2-type |
| FgenesH.VV78X083609.33_1 | IPR007087 | C2H2 | Zinc finger, C2H2-type |
| FgenesH.VV78X083645.12_4 | IPR007087 | C2H2 | Zinc finger, C2H2-type |
| FgenesH.VV78X087181.7_1 | IPR007087 | C2H2 | Zinc finger, C2H2-type |
| FgenesH.VV78X093155.30_11 | IPR007087 | C2H2 | Zinc finger, C2H2-type |
| FgenesH.VV78X099428.3_1 | IPR007087 | C2H2 | Zinc finger, C2H2-type |
| FgenesH.VV78X099670.6_3 | IPR007087 | C2H2 | Zinc finger, C2H2-type |
| FgenesH.VV78X116155.5_1 | IPR007087 | C2H2 | Zinc finger, C2H2-type |
| FgenesH.VV78X117801.19_12 | IPR007087 | C2H2 | Zinc finger, C2H2-type |
| FgenesH.VV78X129692.21_3 | IPR007087 | C2H2 | Zinc finger, C2H2-type |
| FgenesH.VV78X143442.4_4 | IPR007087 | C2H2 | Zinc finger, C2H2-type |
| FgenesH.VV78X162447.7_1 | IPR007087 | C2H2 | Zinc finger, C2H2-type |
| FgenesH.VV78X163745.21_2 | IPR007087 | C2H2 | Zinc finger, C2H2-type |
| FgenesH.VV78X174008.4_3 | IPR007087 | C2H2 | Zinc finger, C2H2-type |
| FgenesH.VV78X177522.3_1 | IPR007087 | C2H2 | Zinc finger, C2H2-type |
| FgenesH.VV78X179498.9_3 | IPR007087 | C2H2 | Zinc finger, C2H2-type |
| FgenesH.VV78X181906.5_3 | IPR007087 | C2H2 | Zinc finger, C2H2-type |
| FgenesH.VV78X190100.14_2 | IPR007087 | C2H2 | Zinc finger, C2H2-type |
| FgenesH.VV78X199310.5_5 | IPR007087 | C2H2 | Zinc finger, C2H2-type |
| FgenesH.VV78X204692.9_2 | IPR007087 | C2H2 | Zinc finger, C2H2-type |
| FgenesH.VV78X219674.11_3 | IPR007087 | C2H2 | Zinc finger, C2H2-type |
| FgenesH.VV78X227225.4_3 | IPR007087 | C2H2 | Zinc finger, C2H2-type |
| FgenesH.VV78X237207.11_2 | IPR007087 | C2H2 | Zinc finger, C2H2-type |
| FgenesH.VV78X242738.14_2 | IPR007087 | C2H2 | Zinc finger, C2H2-type |
| FgenesH.VV78X242878.11_4 | IPR007087 | C2H2 | Zinc finger, C2H2-type |
| FgenesH.VV78X254909.21_5 | IPR007087 | C2H2 | Zinc finger, C2H2-type |
| FgenesH.VV78X260584.5_1 | IPR007087 | C2H2 | Zinc finger, C2H2-type |
| Glimmer1.VV78X012233.18_2 | IPR007087 | C2H2 | Zinc finger, C2H2-type |
| Glimmer1.VV78X070138.9_3 | IPR007087 | C2H2 | Zinc finger, C2H2-type |
| Glimmer1.VV78X093155.30_14 | IPR007087 | C2H2 | Zinc finger, C2H2-type |
| Glimmer1.VV78X097754.15_6 | IPR007087 | C2H2 | Zinc finger, C2H2-type |
| Glimmer1.VV78X100517.5_3 | IPR007087 | C2H2 | Zinc finger, C2H2-type |
| Glimmer1.VV78X124416.7_4 | IPR007087 | C2H2 | Zinc finger, C2H2-type |
| Glimmer1.VV78X138791.8_2 | IPR007087 | C2H2 | Zinc finger, C2H2-type |
| Glimmer1.VV78X179149.4_2 | IPR007087 | C2H2 | Zinc finger, C2H2-type |
| Glimmer1.VV78X215512.2_3 | IPR007087 | C2H2 | Zinc finger, C2H2-type |
| Glimmer1.VV78X220600.5_2 | IPR007087 | C2H2 | Zinc finger, C2H2-type |
| Sim4.aln-A06001447 | IPR007087 | C2H2 | Zinc finger, C2H2-type |
| Sim4.aln-A06002278 | IPR007087 | C2H2 | Zinc finger, C2H2-type |
| Sim4.aln-A06004782 | IPR007087 | C2H2 | Zinc finger, C2H2-type |
| Sim4.aln-A06004833 | IPR007087 | C2H2 | Zinc finger, C2H2-type |
| Sim4.aln-A06011216 | IPR007087 | C2H2 | Zinc finger, C2H2-type |
| Sim4.aln-A06014895 | IPR007087 | C2H2 | Zinc finger, C2H2-type |
| Sim4.aln-A06015203 | IPR007087 | C2H2 | Zinc finger, C2H2-type |
| Sim4.aln-A06015826 | IPR007087 | C2H2 | Zinc finger, C2H2-type |
| Sim4.aln-TCVV001088 | IPR007087 | C2H2 | Zinc finger, C2H2-type |
| Sim4.aln-TCVV001818 | IPR007087 | C2H2 | Zinc finger, C2H2-type |
| Sim4.aln-TCVV001825 | IPR007087 | C2H2 | Zinc finger, C2H2-type |
| Sim4.aln-TCVV001876 | IPR007087 | C2H2 | Zinc finger, C2H2-type |
| Sim4.aln-TCVV001893 | IPR007087 | C2H2 | Zinc finger, C2H2-type |
| Sim4.aln-TCVV003694 | IPR007087 | C2H2 | Zinc finger, C2H2-type |
| Sim4.aln-TCVV005380 | IPR007087 | C2H2 | Zinc finger, C2H2-type |
| Sim4.aln-TCVV005565 | IPR007087 | C2H2 | Zinc finger, C2H2-type |
| Sim4.aln-TCVV006568 | IPR007087 | C2H2 | Zinc finger, C2H2-type |
| Sim4.aln-TCVV007755 | IPR007087 | C2H2 | Zinc finger, C2H2-type |
| Sim4.aln-TCVV008601 | IPR007087 | C2H2 | Zinc finger, C2H2-type |
| Sim4.aln-TCVV008947 | IPR007087 | C2H2 | Zinc finger, C2H2-type |
| Sim4.aln-TCVV010207 | IPR007087 | C2H2 | Zinc finger, C2H2-type |
| Sim4.aln-TCVV010263 | IPR007087 | C2H2 | Zinc finger, C2H2-type |
| Sim4.aln-TCVV013724 | IPR007087 | C2H2 | Zinc finger, C2H2-type |
| Sim4.aln-TCVV014780 | IPR007087 | C2H2 | Zinc finger, C2H2-type |
| Sim4.aln-TCVV015444 | IPR007087 | C2H2 | Zinc finger, C2H2-type |
| Sim4.aln-TCVV016120 | IPR007087 | C2H2 | Zinc finger, C2H2-type |
| Sim4.aln-TCVV020560 | IPR007087 | C2H2 | Zinc finger, C2H2-type |
| Sim4.aln-TCVV022280 | IPR007087 | C2H2 | Zinc finger, C2H2-type |
| Sim4.aln-TCVV022891 | IPR007087 | C2H2 | Zinc finger, C2H2-type |
| Sim4.aln-TCVV023061 | IPR007087 | C2H2 | Zinc finger, C2H2-type |
| Sim4.aln-TCVV023576 | IPR007087 | C2H2 | Zinc finger, C2H2-type |
| Twinscan1.VV78X015037.17_7 | IPR007087 | C2H2 | Zinc finger, C2H2-type |
| Twinscan1.VV78X035074.3_1 | IPR007087 | C2H2 | Zinc finger, C2H2-type |
| Twinscan1.VV78X102512.5_11 | IPR007087 | C2H2 | Zinc finger, C2H2-type |
| Twinscan1.VV78X144965.37_7 | IPR007087 | C2H2 | Zinc finger, C2H2-type |
| FgenesH.VV78X001301.9_2 | IPR000571 | C3H | Zinc finger, CCCH-type |
| FgenesH.VV78X004021.7_3 | IPR000571 | C3H | Zinc finger, CCCH-type |
| FgenesH.VV78X006605.6_1 | IPR000571 | C3H | Zinc finger, CCCH-type |
| FgenesH.VV78X010511.14_2 | IPR000571 | C3H | Zinc finger, CCCH-type |
| FgenesH.VV78X038396.7_1 | IPR000571 | C3H | Zinc finger, CCCH-type |
| FgenesH.VV78X057207.4_1 | IPR000571 | C3H | Zinc finger, CCCH-type |
| FgenesH.VV78X058952.10_4 | IPR000571 | C3H | Zinc finger, CCCH-type |
| FgenesH.VV78X059718.3_2 | IPR000571 | C3H | Zinc finger, CCCH-type |
| FgenesH.VV78X067066.11_3 | IPR000571 | C3H | Zinc finger, CCCH-type |
| FgenesH.VV78X075559.13_2 | IPR000571 | C3H | Zinc finger, CCCH-type |
| FgenesH.VV78X098029.4_1 | IPR000571 | C3H | Zinc finger, CCCH-type |
| FgenesH.VV78X120340.9_1 | IPR000571 | C3H | Zinc finger, CCCH-type |
| FgenesH.VV78X120395.27_10 | IPR000571 | C3H | Zinc finger, CCCH-type |
| FgenesH.VV78X147737.13_3 | IPR000571 | C3H | Zinc finger, CCCH-type |
| FgenesH.VV78X177830.4_1 | IPR000571 | C3H | Zinc finger, CCCH-type |
| FgenesH.VV78X179571.26_1 | IPR000571 | C3H | Zinc finger, CCCH-type |
| FgenesH.VV78X179691.5_1 | IPR000571 | C3H | Zinc finger, CCCH-type |
| FgenesH.VV78X209176.42_3 | IPR000571 | C3H | Zinc finger, CCCH-type |
| FgenesH.VV78X227619.10_1 | IPR000571 | C3H | Zinc finger, CCCH-type |
| FgenesH.VV78X228491.7_1 | IPR000571 | C3H | Zinc finger, CCCH-type |
| FgenesH.VV78X247281.71_4 | IPR000571 | C3H | Zinc finger, CCCH-type |
| Glimmer1.VV78X002638.10_2 | IPR000571 | C3H | Zinc finger, CCCH-type |
| Glimmer1.VV78X252469.11_3 | IPR000571 | C3H | Zinc finger, CCCH-type |
| Glimmer1.VV78X257465.39_4 | IPR000571 | C3H | Zinc finger, CCCH-type |
| Sim4.aln-A06012939 | IPR000571 | C3H | Zinc finger, CCCH-type |
| Sim4.aln-TCVV000233 | IPR000467 | C3H | D111/G-patch |
| Sim4.aln-TCVV001733 | IPR000571 | C3H | Zinc finger, CCCH-type |
| Sim4.aln-TCVV001967 | IPR000571 | C3H | Zinc finger, CCCH-type |
| Sim4.aln-TCVV002505 | IPR000571 | C3H | Zinc finger, CCCH-type |
| Sim4.aln-TCVV003215 | IPR000571 | C3H | Zinc finger, CCCH-type |
| Sim4.aln-TCVV005927 | IPR000571 | C3H | Zinc finger, CCCH-type |
| Sim4.aln-TCVV005938 | IPR000571 | C3H | Zinc finger, CCCH-type |
| Sim4.aln-TCVV006739 | IPR000571 | C3H | Zinc finger, CCCH-type |
| Sim4.aln-TCVV009243 | IPR000571 | C3H | Zinc finger, CCCH-type |
| Sim4.aln-TCVV010685 | IPR000571 | C3H | Zinc finger, CCCH-type |
| Sim4.aln-TCVV011670 | IPR000571 | C3H | Zinc finger, CCCH-type |
| Sim4.aln-TCVV012239 | IPR000571 | C3H | Zinc finger, CCCH-type |
| Sim4.aln-TCVV012589 | IPR000571 | C3H | Zinc finger, CCCH-type |
| Sim4.aln-TCVV015022 | IPR000571 | C3H | Zinc finger, CCCH-type |
| Sim4.aln-TCVV016551 | IPR000571 | C3H | Zinc finger, CCCH-type |
| Sim4.aln-TCVV021287 | IPR000571 | C3H | Zinc finger, CCCH-type |
| Sim4.aln-TCVV022888 | IPR000571 | C3H | Zinc finger, CCCH-type |
| FgenesH.VV78X069996.10_2 | IPR002867 | C6H5 | Zinc finger, C6HC-type |
| FgenesH.VV78X067790.25_1 | IPR000048 | CAMTA | IQ calmodulin-binding region |
| FgenesH.VV78X210461.8_2 | IPR005559 | CAMTA | CG-1 |
| FgenesH.VV78X271574.10_2 | IPR005559 | CAMTA | CG-1 |
| Sim4.aln-TCVV000498 | IPR000048 | CAMTA | IQ calmodulin-binding region |
| Sim4.aln-TCVV001626 | IPR000048 | CAMTA | IQ calmodulin-binding region |
| Sim4.aln-TCVV001900 | IPR000048 | CAMTA | IQ calmodulin-binding region |
| Sim4.aln-TCVV006588 | IPR000048 | CAMTA | IQ calmodulin-binding region |
| Sim4.aln-TCVV013244 | IPR005559 | CAMTA | CG-1 |
| Sim4.aln-TCVV016515 | IPR000048 | CAMTA | IQ calmodulin-binding region |
| Sim4.aln-TCVV019019 | IPR000048 | CAMTA | IQ calmodulin-binding region |
| Sim4.aln-TCVV023929 | IPR000048 | CAMTA | IQ calmodulin-binding region |
| Twinscan1.VV78X241396.15_1 | IPR000048 | CAMTA | IQ calmodulin-binding region |
| FgenesH.VV78X036116.11_3 | IPR003958 | CCAAT-Dr1 | Transcription factor CBF/NF-Y/archaeal histone |
| FgenesH.VV78X134315.9_5 | IPR003958 | CCAAT-Dr1 | Transcription factor CBF/NF-Y/archaeal histone |
| FgenesH.VV78X240579.7_1 | IPR003958 | CCAAT-Dr1 | Transcription factor CBF/NF-Y/archaeal histone |
| FgenesH.VV78X251901.13_1 | IPR003958 | CCAAT-Dr1 | Transcription factor CBF/NF-Y/archaeal histone |
| Sim4.aln-TCVV001409 | IPR003958 | CCAAT-Dr1 | Transcription factor CBF/NF-Y/archaeal histone |
| Sim4.aln-TCVV002897 | IPR003958 | CCAAT-Dr1 | Transcription factor CBF/NF-Y/archaeal histone |
| Sim4.aln-TCVV004403 | IPR003958 | CCAAT-Dr1 | Transcription factor CBF/NF-Y/archaeal histone |
| Sim4.aln-TCVV006446 | IPR003958 | CCAAT-Dr1 | Transcription factor CBF/NF-Y/archaeal histone |
| Sim4.aln-TCVV006973 | IPR003958 | CCAAT-Dr1 | Transcription factor CBF/NF-Y/archaeal histone |
| Sim4.aln-TCVV014039 | IPR003958 | CCAAT-Dr1 | Transcription factor CBF/NF-Y/archaeal histone |
| Sim4.aln-TCVV017560 | IPR003958 | CCAAT-Dr1 | Transcription factor CBF/NF-Y/archaeal histone |
| FgenesH.VV78X208578.6_1 | IPR001289 | CCAAT-HAP2 | CCAAT-binding transcription factor, subunit B |
| FgenesH.VV78X214798.14_1 | IPR001289 | CCAAT-HAP2 | CCAAT-binding transcription factor, subunit B |
| FgenesH.VV78X243821.10_1 | IPR001289 | CCAAT-HAP2 | CCAAT-binding transcription factor, subunit B |
| Sim4.aln-TCVV002740 | IPR001289 | CCAAT-HAP2 | CCAAT-binding transcription factor, subunit B |
| Sim4.aln-TCVV006932 | IPR001289 | CCAAT-HAP2 | CCAAT-binding transcription factor, subunit B |
| Sim4.aln-TCVV009073 | IPR001289 | CCAAT-HAP2 | CCAAT-binding transcription factor, subunit B |
| Sim4.aln-TCVV012181 | IPR001289 | CCAAT-HAP2 | CCAAT-binding transcription factor, subunit B |
| FgenesH.VV78X035009.6_1 | IPR003957 | CCAAT-HAP3 | Histone-like transcription factor/archaeal histone/DNA topoisomerase |
| FgenesH.VV78X047419.4_1 | IPR003957 | CCAAT-HAP3 | Histone-like transcription factor/archaeal histone/DNA topoisomerase |
| FgenesH.VV78X051309.14_7 | IPR003957 | CCAAT-HAP3 | Histone-like transcription factor/archaeal histone/DNA topoisomerase |
| FgenesH.VV78X064733.7_5 | IPR003957 | CCAAT-HAP3 | Histone-like transcription factor/archaeal histone/DNA topoisomerase |
| FgenesH.VV78X144965.37_2 | IPR003957 | CCAAT-HAP3 | Histone-like transcription factor/archaeal histone/DNA topoisomerase |
| FgenesH.VV78X188692.4_1 | IPR003957 | CCAAT-HAP3 | Histone-like transcription factor/archaeal histone/DNA topoisomerase |
| FgenesH.VV78X240850.2_4 | IPR003957 | CCAAT-HAP3 | Histone-like transcription factor/archaeal histone/DNA topoisomerase |
| FgenesH.VV78X276468.7_2 | IPR003957 | CCAAT-HAP3 | Histone-like transcription factor/archaeal histone/DNA topoisomerase |
| Sim4.aln-A06005776 | IPR003957 | CCAAT-HAP3 | Histone-like transcription factor/archaeal histone/DNA topoisomerase |
| Sim4.aln-A06011490 | IPR003957 | CCAAT-HAP3 | Histone-like transcription factor/archaeal histone/DNA topoisomerase |
| Sim4.aln-TCVV001780 | IPR003957 | CCAAT-HAP3 | Histone-like transcription factor/archaeal histone/DNA topoisomerase |
| Sim4.aln-TCVV007870 | IPR003957 | CCAAT-HAP3 | Histone-like transcription factor/archaeal histone/DNA topoisomerase |
| FgenesH.VV78X060572.10_4 | IPR007125 | CCAAT-HAP5 | Histone core |
| FgenesH.VV78X067245.5_1 | IPR007125 | CCAAT-HAP5 | Histone core |
| FgenesH.VV78X082395.11_1 | IPR007125 | CCAAT-HAP5 | Histone core |
| FgenesH.VV78X169932.4_1 | IPR007125 | CCAAT-HAP5 | Histone core |
| Glimmer1.VV78X095275.5_2 | IPR007125 | CCAAT-HAP5 | Histone core |
| Sim4.aln-A06001358 | IPR007125 | CCAAT-HAP5 | Histone core |
| Sim4.aln-A06001791 | IPR007125 | CCAAT-HAP5 | Histone core |
| Sim4.aln-A06002565 | IPR007125 | CCAAT-HAP5 | Histone core |
| Sim4.aln-A06002746 | IPR007125 | CCAAT-HAP5 | Histone core |
| Sim4.aln-A06003315 | IPR007125 | CCAAT-HAP5 | Histone core |
| Sim4.aln-A06004948 | IPR007125 | CCAAT-HAP5 | Histone core |
| Sim4.aln-A06006862 | IPR007125 | CCAAT-HAP5 | Histone core |
| Sim4.aln-A06007200 | IPR007125 | CCAAT-HAP5 | Histone core |
| Sim4.aln-A06007508 | IPR007125 | CCAAT-HAP5 | Histone core |
| Sim4.aln-A06014536 | IPR007125 | CCAAT-HAP5 | Histone core |
| Sim4.aln-A06014553 | IPR007125 | CCAAT-HAP5 | Histone core |
| Sim4.aln-A06014733 | IPR007125 | CCAAT-HAP5 | Histone core |
| Sim4.aln-A06016548 | IPR007125 | CCAAT-HAP5 | Histone core |
| Sim4.aln-TCVV000881 | IPR007125 | CCAAT-HAP5 | Histone core |
| Sim4.aln-TCVV001129 | IPR007125 | CCAAT-HAP5 | Histone core |
| Sim4.aln-TCVV003473 | IPR007125 | CCAAT-HAP5 | Histone core |
| Sim4.aln-TCVV003505 | IPR007125 | CCAAT-HAP5 | Histone core |
| Sim4.aln-TCVV003737 | IPR007125 | CCAAT-HAP5 | Histone core |
| Sim4.aln-TCVV003857 | IPR007125 | CCAAT-HAP5 | Histone core |
| Sim4.aln-TCVV006150 | IPR007125 | CCAAT-HAP5 | Histone core |
| Sim4.aln-TCVV007447 | IPR007125 | CCAAT-HAP5 | Histone core |
| Sim4.aln-TCVV009287 | IPR007125 | CCAAT-HAP5 | Histone core |
| Sim4.aln-TCVV009597 | IPR007125 | CCAAT-HAP5 | Histone core |
| Sim4.aln-TCVV009611 | IPR007125 | CCAAT-HAP5 | Histone core |
| Sim4.aln-TCVV009620 | IPR007125 | CCAAT-HAP5 | Histone core |
| Sim4.aln-TCVV013044 | IPR007125 | CCAAT-HAP5 | Histone core |
| Sim4.aln-TCVV022470 | IPR007125 | CCAAT-HAP5 | Histone core |
| Sim4.aln-TCVV022639 | IPR007125 | CCAAT-HAP5 | Histone core |
| Sim4.aln-TCVV022657 | IPR007125 | CCAAT-HAP5 | Histone core |
| Sim4.aln-TCVV023409 | IPR007125 | CCAAT-HAP5 | Histone core |
| Sim4.aln-TCVV023447 | IPR007125 | CCAAT-HAP5 | Histone core |
| Sim4.aln-TCVV023661 | IPR007125 | CCAAT-HAP5 | Histone core |
| Sim4.aln-TCVV023677 | IPR007125 | CCAAT-HAP5 | Histone core |
| Sim4.aln-TCVV023693 | IPR007125 | CCAAT-HAP5 | Histone core |
| Sim4.aln-TCVV023709 | IPR007125 | CCAAT-HAP5 | Histone core |
| Sim4.aln-TCVV023794 | IPR007125 | CCAAT-HAP5 | Histone core |
| Sim4.aln-TCVV023898 | IPR007125 | CCAAT-HAP5 | Histone core |
| Sim4.aln-TCVV023955 | IPR007125 | CCAAT-HAP5 | Histone core |
| Sim4.aln-TCVV024026 | IPR007125 | CCAAT-HAP5 | Histone core |
| Sim4.aln-TCVV024149 | IPR007125 | CCAAT-HAP5 | Histone core |
| Twinscan1.VV78X176945.13_2 | IPR007125 | CCAAT-HAP5 | Histone core |
| FgenesH.VV78X015144.6_2 | IPR005172 | CPP | Tesmin/TSO1-like, CXC |
| FgenesH.VV78X101832.7_1 | IPR005172 | CPP | Tesmin/TSO1-like, CXC |
| Sim4.aln-TCVV012503 | IPR005172 | CPP | Tesmin/TSO1-like, CXC |
| FgenesH.VV78X155059.4_5 | IPR002059 | CSD | Cold-shock protein, DNA-binding |
| FgenesH.VV78X159970.6_1 | IPR001932 | DBP | Protein phosphatase 2C-related |
| FgenesH.VV78X193609.6_1 | IPR001932 | DBP | Protein phosphatase 2C-related |
| Sim4.aln-A06003879 | IPR001932 | DBP | Protein phosphatase 2C-related |
| Sim4.aln-A06019640 | IPR001932 | DBP | Protein phosphatase 2C-related |
| Sim4.aln-TCVV007018 | IPR001932 | DBP | Protein phosphatase 2C-related |
| Sim4.aln-TCVV009522 | IPR001932 | DBP | Protein phosphatase 2C-related |
| Sim4.aln-TCVV015591 | IPR001932 | DBP | Protein phosphatase 2C-related |
| FgenesH.VV78X100755.7_1 | IPR004022 | DDT | DDT |
| FgenesH.VV78X157592.9_2 | IPR004022 | DDT | DDT |
| Glimmer1.VV78X011092.4_2 | IPR004022 | DDT | DDT |
| FgenesH.VV78X113719.6_2 | IPR003034 | SAP | DNA-binding SAP |
| FgenesH.VV78X151523.15_1 | IPR003034 | SAP | DNA-binding SAP |
| FgenesH.VV78X193630.6_1 | IPR003034 | SAP | DNA-binding SAP |
| Sim4.aln-TCVV011525 | IPR003034 | SAP | DNA-binding SAP |
| Sim4.aln-TCVV014974 | IPR003034 | SAP | DNA-binding SAP |
| FgenesH.VV78X137860.11_3 | IPR003316 | E2F-DP | Transcription factor E2F/dimerisation partner (TDP) |
| FgenesH.VV78X138031.14_3 | IPR003316 | E2F-DP | Transcription factor E2F/dimerisation partner (TDP) |
| FgenesH.VV78X233573.5_1 | IPR003316 | E2F-DP | Transcription factor E2F/dimerisation partner (TDP) |
| FgenesH.VV78X257382.43_3 | IPR003316 | E2F-DP | Transcription factor E2F/dimerisation partner (TDP) |
| Sim4.aln-TCVV004958 | IPR003316 | E2F-DP | Transcription factor E2F/dimerisation partner (TDP) |
| FgenesH.VV78X037171.18_2 | IPR006957 | EIL | Ethylene insensitive 3 |
| FgenesH.VV78X051556.7_3 | IPR006957 | EIL | Ethylene insensitive 3 |
| Sim4.aln-TCVV000476 | IPR006957 | EIL | Ethylene insensitive 3 |
| Sim4.aln-TCVV016219 | IPR006957 | EIL | Ethylene insensitive 3 |
| Twinscan1.VV78X201131.6_1 | IPR006957 | EIL | Ethylene insensitive 3 |
| FgenesH.VV78X005019.10_1 | IPR005202 | GRAS | GRAS transcription factor |
| FgenesH.VV78X036116.11_2 | IPR005202 | GRAS | GRAS transcription factor |
| FgenesH.VV78X041360.8_2 | IPR005202 | GRAS | GRAS transcription factor |
| FgenesH.VV78X102540.11_2 | IPR005202 | GRAS | GRAS transcription factor |
| FgenesH.VV78X134043.50_1 | IPR005202 | GRAS | GRAS transcription factor |
| FgenesH.VV78X169822.14_3 | IPR005202 | GRAS | GRAS transcription factor |
| FgenesH.VV78X187525.5_1 | IPR005202 | GRAS | GRAS transcription factor |
| FgenesH.VV78X190796.4_4 | IPR005202 | GRAS | GRAS transcription factor |
| FgenesH.VV78X211539.4_5 | IPR005202 | GRAS | GRAS transcription factor |
| FgenesH.VV78X219796.5_2 | IPR005202 | GRAS | GRAS transcription factor |
| FgenesH.VV78X235829.7_1 | IPR005202 | GRAS | GRAS transcription factor |
| FgenesH.VV78X247367.48_2 | IPR005202 | GRAS | GRAS transcription factor |
| FgenesH.VV78X271656.5_3 | IPR005202 | GRAS | GRAS transcription factor |
| FgenesH.VV78X272540.9_5 | IPR005202 | GRAS | GRAS transcription factor |
| Glimmer1.VV78X087117.8_1 | IPR005202 | GRAS | GRAS transcription factor |
| Glimmer1.VV78X112647.12_3 | IPR005202 | GRAS | GRAS transcription factor |
| Glimmer1.VV78X168671.5_2 | IPR005202 | GRAS | GRAS transcription factor |
| Glimmer1.VV78X204484.5_1 | IPR005202 | GRAS | GRAS transcription factor |
| Sim4.aln-A06001897 | IPR005202 | GRAS | GRAS transcription factor |
| Sim4.aln-A06014749 | IPR005202 | GRAS | GRAS transcription factor |
| Sim4.aln-TCVV000513 | IPR005202 | GRAS | GRAS transcription factor |
| Sim4.aln-TCVV002014 | IPR005202 | GRAS | GRAS transcription factor |
| Sim4.aln-TCVV002311 | IPR005202 | GRAS | GRAS transcription factor |
| Sim4.aln-TCVV004524 | IPR005202 | GRAS | GRAS transcription factor |
| Sim4.aln-TCVV004678 | IPR005202 | GRAS | GRAS transcription factor |
| Sim4.aln-TCVV005316 | IPR005202 | GRAS | GRAS transcription factor |
| Sim4.aln-TCVV008099 | IPR005202 | GRAS | GRAS transcription factor |
| Sim4.aln-TCVV009582 | IPR005202 | GRAS | GRAS transcription factor |
| Sim4.aln-TCVV012539 | IPR005202 | GRAS | GRAS transcription factor |
| Sim4.aln-TCVV013550 | IPR005202 | GRAS | GRAS transcription factor |
| Sim4.aln-TCVV015562 | IPR005202 | GRAS | GRAS transcription factor |
| Sim4.aln-TCVV017080 | IPR005202 | GRAS | GRAS transcription factor |
| Sim4.aln-TCVV017727 | IPR005202 | GRAS | GRAS transcription factor |
| Sim4.aln-TCVV023782 | IPR005202 | GRAS | GRAS transcription factor |
| Twinscan1.VV78X038863.21_5 | IPR005202 | GRAS | GRAS transcription factor |
| Twinscan1.VV78X246431.3_2 | IPR005202 | GRAS | GRAS transcription factor |
| FgenesH.VV78X012396.11_1 | IPR001356 | HB | Homeobox |
| FgenesH.VV78X020791.9_3 | IPR002913 | HB | Lipid-binding START |
| FgenesH.VV78X032979.11_2 | IPR001356 | HB | Homeobox |
| FgenesH.VV78X052697.14_2 | IPR001356 | HB | Homeobox |
| FgenesH.VV78X054788.8_1 | IPR001356 | HB | Homeobox |
| FgenesH.VV78X057071.10_1 | IPR005539 | HB | ELK |
| FgenesH.VV78X059323.8_1 | IPR001356 | HB | Homeobox |
| FgenesH.VV78X059360.5_1 | IPR001356 | HB | Homeobox |
| FgenesH.VV78X068576.4_4 | IPR001356 | HB | Homeobox |
| FgenesH.VV78X076481.6_2 | IPR001356 | HB | Homeobox |
| FgenesH.VV78X078650.7_8 | IPR001356 | HB | Homeobox |
| FgenesH.VV78X079656.5_2 | IPR002913 | HB | Lipid-binding START |
| FgenesH.VV78X083454.9_4 | IPR001356 | HB | Homeobox |
| FgenesH.VV78X087681.17_1 | IPR001356 | HB | Homeobox |
| FgenesH.VV78X110597.11_2 | IPR001356 | HB | Homeobox |
| FgenesH.VV78X118216.6_5 | IPR001356 | HB | Homeobox |
| FgenesH.VV78X162089.20_1 | IPR001356 | HB | Homeobox |
| FgenesH.VV78X165152.5_3 | IPR001356 | HB | Homeobox |
| FgenesH.VV78X179473.2_1 | IPR001356 | HB | Homeobox |
| FgenesH.VV78X207550.11_1 | IPR005540 | HB | KNOX1 |
| FgenesH.VV78X214500.13_5 | IPR001356 | HB | Homeobox |
| FgenesH.VV78X225787.14_3 | IPR001356 | HB | Homeobox |
| FgenesH.VV78X238230.4_1 | IPR001356 | HB | Homeobox |
| FgenesH.VV78X241236.14_12 | IPR001356 | HB | Homeobox |
| FgenesH.VV78X244179.3_2 | IPR001356 | HB | Homeobox |
| FgenesH.VV78X252893.7_1 | IPR001356 | HB | Homeobox |
| FgenesH.VV78X255096.3_3 | IPR001356 | HB | Homeobox |
| FgenesH.VV78X256089.20_2 | IPR001356 | HB | Homeobox |
| FgenesH.VV78X256089.20_3 | IPR002913 | HB | Lipid-binding START |
| FgenesH.VV78X263102.7_2 | IPR001356 | HB | Homeobox |
| FgenesH.VV78X264876.10_4 | IPR001356 | HB | Homeobox |
| FgenesH.VV78X266807.6_1 | IPR001356 | HB | Homeobox |
| FgenesH.VV78X267462.3_6 | IPR001356 | HB | Homeobox |
| FgenesH.VV79X008371.2_1 | IPR001356 | HB | Homeobox |
| Glimmer1.VV78X068252.9_2 | IPR001356 | HB | Homeobox |
| Glimmer1.VV78X074180.5_2 | IPR005539 | HB | ELK |
| Glimmer1.VV78X126512.6_2 | IPR001356 | HB | Homeobox |
| Glimmer1.VV78X150337.7_2 | IPR006563 | HB | POX |
| Sim4.aln-A06003362 | IPR005540 | HB | KNOX1 |
| Sim4.aln-A06004511 | IPR005540 | HB | KNOX1 |
| Sim4.aln-A06010450 | IPR001356 | HB | Homeobox |
| Sim4.aln-A06013254 | IPR005539 | HB | ELK |
| Sim4.aln-A06013767 | IPR001356 | HB | Homeobox |
| Sim4.aln-A06015547 | IPR013978 | HB | MEKHLA |
| Sim4.aln-A06016350 | IPR001356 | HB | Homeobox |
| Sim4.aln-A06019482 | IPR002913 | HB | Lipid-binding START |
| Sim4.aln-TCVV000646 | IPR001356 | HB | Homeobox |
| Sim4.aln-TCVV000864 | IPR001356 | HB | Homeobox |
| Sim4.aln-TCVV000997 | IPR001356 | HB | Homeobox |
| Sim4.aln-TCVV001386 | IPR001356 | HB | Homeobox |
| Sim4.aln-TCVV001716 | IPR002913 | HB | Lipid-binding START |
| Sim4.aln-TCVV001842 | IPR005539 | HB | ELK |
| Sim4.aln-TCVV002201 | IPR001356 | HB | Homeobox |
| Sim4.aln-TCVV002263 | IPR001356 | HB | Homeobox |
| Sim4.aln-TCVV002841 | IPR005539 | HB | ELK |
| Sim4.aln-TCVV004167 | IPR001356 | HB | Homeobox |
| Sim4.aln-TCVV006927 | IPR001356 | HB | Homeobox |
| Sim4.aln-TCVV007504 | IPR001356 | HB | Homeobox |
| Sim4.aln-TCVV007843 | IPR001356 | HB | Homeobox |
| Sim4.aln-TCVV008012 | IPR001356 | HB | Homeobox |
| Sim4.aln-TCVV008498 | IPR001356 | HB | Homeobox |
| Sim4.aln-TCVV008828 | IPR013978 | HB | MEKHLA |
| Sim4.aln-TCVV008846 | IPR005539 | HB | ELK |
| Sim4.aln-TCVV012193 | IPR005539 | HB | ELK |
| Sim4.aln-TCVV012768 | IPR005539 | HB | ELK |
| Sim4.aln-TCVV012875 | IPR001356 | HB | Homeobox |
| Sim4.aln-TCVV013230 | IPR001356 | HB | Homeobox |
| Sim4.aln-TCVV013386 | IPR013978 | HB | MEKHLA |
| Sim4.aln-TCVV014723 | IPR001356 | HB | Homeobox |
| Sim4.aln-TCVV015340 | IPR013978 | HB | MEKHLA |
| Sim4.aln-TCVV016079 | IPR005540 | HB | KNOX1 |
| Sim4.aln-TCVV016268 | IPR001356 | HB | Homeobox |
| Sim4.aln-TCVV016873 | IPR001356 | HB | Homeobox |
| Sim4.aln-TCVV018267 | IPR001356 | HB | Homeobox |
| Sim4.aln-TCVV019731 | IPR006563 | HB | POX |
| Sim4.aln-TCVV022874 | IPR002913 | HB | Lipid-binding START |
| Sim4.aln-TCVV023034 | IPR001356 | HB | Homeobox |
| Sim4.aln-TCVV023951 | IPR001356 | HB | Homeobox |
| Twinscan1.VV78X001506.3_1 | IPR001356 | HB | Homeobox |
| Twinscan1.VV78X154850.11_6 | IPR001356 | HB | Homeobox |
| FgenesH.VV78X042663.3_2 | IPR000910 | HMG | HMG1/2 (high mobility group)box |
| FgenesH.VV78X050883.21_3 | IPR000910 | HMG | HMG1/2 (high mobility group)box |
| FgenesH.VV78X240744.7_1 | IPR000910 | HMG | HMG1/2 (high mobility group)box |
| FgenesH.VV78X246043.27_3 | IPR000910 | HMG | HMG1/2 (high mobility group)box |
| Sim4.aln-TCVV003499 | IPR000910 | HMG | HMG1/2 (high mobility group)box |
| Sim4.aln-TCVV007341 | IPR000910 | HMG | HMG1/2 (high mobility group)box |
| Sim4.aln-TCVV012707 | IPR000910 | HMG | HMG1/2 (high mobility group)box |
| Sim4.aln-TCVV012796 | IPR000910 | HMG | HMG1/2 (high mobility group)box |
| Sim4.aln-TCVV021489 | IPR000910 | HMG | HMG1/2 (high mobility group)box |
| Sim4.aln-TCVV023149 | IPR000910 | HMG | HMG1/2 (high mobility group)box |
| Sim4.aln-TCVV023281 | IPR000910 | HMG | HMG1/2 (high mobility group)box |
| Sim4.aln-TCVV023819 | IPR000910 | HMG | HMG1/2 (high mobility group)box |
| FgenesH.VV78X000064.7_1 | IPR000232 | HSF | Heat shock factor (HSF)-type, DNA-binding |
| FgenesH.VV78X030689.25_1 | IPR000232 | HSF | Heat shock factor (HSF)-type, DNA-binding |
| FgenesH.VV78X048623.11_4 | IPR000232 | HSF | Heat shock factor (HSF)-type, DNA-binding |
| FgenesH.VV78X068286.4_2 | IPR000232 | HSF | Heat shock factor (HSF)-type, DNA-binding |
| FgenesH.VV78X139241.9_6 | IPR000232 | HSF | Heat shock factor (HSF)-type, DNA-binding |
| FgenesH.VV78X202140.8_1 | IPR000232 | HSF | Heat shock factor (HSF)-type, DNA-binding |
| FgenesH.VV78X232848.3_2 | IPR000232 | HSF | Heat shock factor (HSF)-type, DNA-binding |
| FgenesH.VV78X258796.7_2 | IPR000232 | HSF | Heat shock factor (HSF)-type, DNA-binding |
| FgenesH.VV78X272044.7_6 | IPR000232 | HSF | Heat shock factor (HSF)-type, DNA-binding |
| Sim4.aln-A06017765 | IPR000232 | HSF | Heat shock factor (HSF)-type, DNA-binding |
| Sim4.aln-TCVV001245 | IPR000232 | HSF | Heat shock factor (HSF)-type, DNA-binding |
| Sim4.aln-TCVV002011 | IPR000232 | HSF | Heat shock factor (HSF)-type, DNA-binding |
| Sim4.aln-TCVV004363 | IPR000232 | HSF | Heat shock factor (HSF)-type, DNA-binding |
| Sim4.aln-TCVV008725 | IPR000232 | HSF | Heat shock factor (HSF)-type, DNA-binding |
| Sim4.aln-TCVV019150 | IPR000232 | HSF | Heat shock factor (HSF)-type, DNA-binding |
| Sim4.aln-TCVV019286 | IPR000232 | HSF | Heat shock factor (HSF)-type, DNA-binding |
| Sim4.aln-TCVV019706 | IPR000232 | HSF | Heat shock factor (HSF)-type, DNA-binding |
| Sim4.aln-TCVV002737 | IPR003594 | HTPase | ATP-binding region, ATPase-like |
| FgenesH.VV78X011128.17_4 | IPR013129 | Jumonjii | Transcription factor jumonji |
| FgenesH.VV78X035889.3_2 | IPR013129 | Jumonjii | Transcription factor jumonji |
| FgenesH.VV78X039912.12_4 | IPR013129 | Jumonjii | Transcription factor jumonji |
| FgenesH.VV78X089690.6_9 | IPR003889 | Jumonjii | FY-rich, C-terminal |
| FgenesH.VV78X108288.6_4 | IPR013129 | Jumonjii | Transcription factor jumonji |
| FgenesH.VV78X109047.7_1 | IPR013129 | Jumonjii | Transcription factor jumonji |
| FgenesH.VV78X137407.9_1 | IPR013129 | Jumonjii | Transcription factor jumonji |
| FgenesH.VV78X141263.15_1 | IPR013129 | Jumonjii | Transcription factor jumonji |
| FgenesH.VV78X199681.46_2 | IPR004198 | Jumonjii | Zinc finger, C5HC2-type |
| FgenesH.VV78X199681.46_3 | IPR013129 | Jumonjii | Transcription factor jumonji |
| FgenesH.VV78X233818.5_1 | IPR003888 | Jumonjii | FY-rich, N-terminal |
| Sim4.aln-TCVV021701 | IPR013129 | Jumonjii | Transcription factor jumonji |
| Glimmer1.VV78X130314.7_15 | IPR002910 | LFY | Floricaula/leafy protein |
| FgenesH.VV78X031549.6_5 | IPR001781 | LIM | LIM, zinc-binding |
| FgenesH.VV78X106678.4_1 | IPR001781 | LIM | LIM, zinc-binding |
| FgenesH.VV78X106678.4_2 | IPR001781 | LIM | LIM, zinc-binding |
| FgenesH.VV78X214253.5_1 | IPR001781 | LIM | LIM, zinc-binding |
| Sim4.aln-A06016399 | IPR001781 | LIM | LIM, zinc-binding |
| Sim4.aln-A06016715 | IPR001781 | LIM | LIM, zinc-binding |
| Sim4.aln-TCVV000562 | IPR001781 | LIM | LIM, zinc-binding |
| Sim4.aln-TCVV002061 | IPR001781 | LIM | LIM, zinc-binding |
| Sim4.aln-TCVV012695 | IPR001781 | LIM | LIM, zinc-binding |
| Sim4.aln-TCVV021567 | IPR001781 | LIM | LIM, zinc-binding |
| FgenesH.VV78X098497.33_4 | IPR013720 | LUG | LisH |
| FgenesH.VV78X171653.20_9 | IPR013720 | LUG | LisH |
| Sim4.aln-TCVV012753 | IPR013720 | LUG | LisH |
| FgenesH.VV78X000062.6_3 | IPR002487 | MADS-box | Transcription factor, K-box |
| FgenesH.VV78X000546.9_1 | IPR002487 | MADS-box | Transcription factor, K-box |
| FgenesH.VV78X002580.7_2 | IPR002487 | MADS-box | Transcription factor, K-box |
| FgenesH.VV78X004516.11_1 | IPR002487 | MADS-box | Transcription factor, K-box |
| FgenesH.VV78X004841.6_2 | IPR002487 | MADS-box | Transcription factor, K-box |
| FgenesH.VV78X012363.25_2 | IPR002487 | MADS-box | Transcription factor, K-box |
| FgenesH.VV78X014869.2_1 | IPR002487 | MADS-box | Transcription factor, K-box |
| FgenesH.VV78X016424.5_2 | IPR002487 | MADS-box | Transcription factor, K-box |
| FgenesH.VV78X019039.32_1 | IPR002487 | MADS-box | Transcription factor, K-box |
| FgenesH.VV78X019867.7_3 | IPR002487 | MADS-box | Transcription factor, K-box |
| FgenesH.VV78X021255.14_1 | IPR002487 | MADS-box | Transcription factor, K-box |
| FgenesH.VV78X023618.5_2 | IPR002487 | MADS-box | Transcription factor, K-box |
| FgenesH.VV78X025879.16_6 | IPR002487 | MADS-box | Transcription factor, K-box |
| FgenesH.VV78X026477.7_3 | IPR002487 | MADS-box | Transcription factor, K-box |
| FgenesH.VV78X027473.20_2 | IPR002487 | MADS-box | Transcription factor, K-box |
| FgenesH.VV78X027947.4_1 | IPR002487 | MADS-box | Transcription factor, K-box |
| FgenesH.VV78X028385.7_1 | IPR002487 | MADS-box | Transcription factor, K-box |
| FgenesH.VV78X030902.10_2 | IPR002487 | MADS-box | Transcription factor, K-box |
| FgenesH.VV78X033737.3_2 | IPR002487 | MADS-box | Transcription factor, K-box |
| FgenesH.VV78X044649.11_2 | IPR002487 | MADS-box | Transcription factor, K-box |
| FgenesH.VV78X049274.8_2 | IPR002487 | MADS-box | Transcription factor, K-box |
| FgenesH.VV78X049375.8_3 | IPR002100 | MADS-box | Transcription factor, MADS-box |
| FgenesH.VV78X050106.69_2 | IPR002487 | MADS-box | Transcription factor, K-box |
| FgenesH.VV78X051620.15_2 | IPR002487 | MADS-box | Transcription factor, K-box |
| FgenesH.VV78X052756.19_1 | IPR002487 | MADS-box | Transcription factor, K-box |
| FgenesH.VV78X056049.28_1 | IPR002487 | MADS-box | Transcription factor, K-box |
| FgenesH.VV78X056641.15_2 | IPR002487 | MADS-box | Transcription factor, K-box |
| FgenesH.VV78X059924.14_3 | IPR002487 | MADS-box | Transcription factor, K-box |
| FgenesH.VV78X061432.11_1 | IPR002487 | MADS-box | Transcription factor, K-box |
| FgenesH.VV78X062957.19_1 | IPR002487 | MADS-box | Transcription factor, K-box |
| FgenesH.VV78X063148.12_2 | IPR002487 | MADS-box | Transcription factor, K-box |
| FgenesH.VV78X063148.12_3 | IPR002487 | MADS-box | Transcription factor, K-box |
| FgenesH.VV78X064134.4_2 | IPR002487 | MADS-box | Transcription factor, K-box |
| FgenesH.VV78X066670.11_2 | IPR002487 | MADS-box | Transcription factor, K-box |
| FgenesH.VV78X069105.7_8 | IPR002487 | MADS-box | Transcription factor, K-box |
| FgenesH.VV78X070525.10_2 | IPR002487 | MADS-box | Transcription factor, K-box |
| FgenesH.VV78X072657.15_2 | IPR002487 | MADS-box | Transcription factor, K-box |
| FgenesH.VV78X074917.2_2 | IPR002487 | MADS-box | Transcription factor, K-box |
| FgenesH.VV78X076072.5_2 | IPR002487 | MADS-box | Transcription factor, K-box |
| FgenesH.VV78X082548.5_1 | IPR002487 | MADS-box | Transcription factor, K-box |
| FgenesH.VV78X084696.3_1 | IPR002487 | MADS-box | Transcription factor, K-box |
| FgenesH.VV78X084826.13_2 | IPR002487 | MADS-box | Transcription factor, K-box |
| FgenesH.VV78X086357.8_1 | IPR002487 | MADS-box | Transcription factor, K-box |
| FgenesH.VV78X086810.12_5 | IPR002100 | MADS-box | Transcription factor, MADS-box |
| FgenesH.VV78X088545.27_1 | IPR002487 | MADS-box | Transcription factor, K-box |
| FgenesH.VV78X089070.3_3 | IPR002487 | MADS-box | Transcription factor, K-box |
| FgenesH.VV78X089563.10_1 | IPR002487 | MADS-box | Transcription factor, K-box |
| FgenesH.VV78X091232.6_5 | IPR002100 | MADS-box | Transcription factor, MADS-box |
| FgenesH.VV78X092191.7_2 | IPR002487 | MADS-box | Transcription factor, K-box |
| FgenesH.VV78X092251.6_1 | IPR002487 | MADS-box | Transcription factor, K-box |
| FgenesH.VV78X095727.11_4 | IPR002487 | MADS-box | Transcription factor, K-box |
| FgenesH.VV78X096132.5_1 | IPR002487 | MADS-box | Transcription factor, K-box |
| FgenesH.VV78X096400.15_4 | IPR002487 | MADS-box | Transcription factor, K-box |
| FgenesH.VV78X098355.31_1 | IPR002487 | MADS-box | Transcription factor, K-box |
| FgenesH.VV78X100696.16_4 | IPR002487 | MADS-box | Transcription factor, K-box |
| FgenesH.VV78X101456.3_1 | IPR002487 | MADS-box | Transcription factor, K-box |
| FgenesH.VV78X102158.25_1 | IPR002487 | MADS-box | Transcription factor, K-box |
| FgenesH.VV78X102184.21_6 | IPR002487 | MADS-box | Transcription factor, K-box |
| FgenesH.VV78X105742.11_3 | IPR002487 | MADS-box | Transcription factor, K-box |
| FgenesH.VV78X111902.14_4 | IPR002100 | MADS-box | Transcription factor, MADS-box |
| FgenesH.VV78X113383.10_1 | IPR002487 | MADS-box | Transcription factor, K-box |
| FgenesH.VV78X115637.8_1 | IPR002487 | MADS-box | Transcription factor, K-box |
| FgenesH.VV78X118531.9_1 | IPR002487 | MADS-box | Transcription factor, K-box |
| FgenesH.VV78X123043.13_1 | IPR002487 | MADS-box | Transcription factor, K-box |
| FgenesH.VV78X125421.34_1 | IPR002487 | MADS-box | Transcription factor, K-box |
| FgenesH.VV78X127711.9_17 | IPR002100 | MADS-box | Transcription factor, MADS-box |
| FgenesH.VV78X132196.14_2 | IPR002487 | MADS-box | Transcription factor, K-box |
| FgenesH.VV78X135584.5_3 | IPR002487 | MADS-box | Transcription factor, K-box |
| FgenesH.VV78X135848.11_1 | IPR002487 | MADS-box | Transcription factor, K-box |
| FgenesH.VV78X139419.4_5 | IPR002487 | MADS-box | Transcription factor, K-box |
| FgenesH.VV78X139551.8_3 | IPR002487 | MADS-box | Transcription factor, K-box |
| FgenesH.VV78X141303.10_4 | IPR002487 | MADS-box | Transcription factor, K-box |
| FgenesH.VV78X144369.6_1 | IPR002487 | MADS-box | Transcription factor, K-box |
| FgenesH.VV78X146297.8_1 | IPR002100 | MADS-box | Transcription factor, MADS-box |
| FgenesH.VV78X147598.8_2 | IPR002487 | MADS-box | Transcription factor, K-box |
| FgenesH.VV78X149994.4_1 | IPR002487 | MADS-box | Transcription factor, K-box |
| FgenesH.VV78X151159.11_3 | IPR002487 | MADS-box | Transcription factor, K-box |
| FgenesH.VV78X155567.6_1 | IPR002487 | MADS-box | Transcription factor, K-box |
| FgenesH.VV78X158137.17_2 | IPR002100 | MADS-box | Transcription factor, MADS-box |
| FgenesH.VV78X158559.11_2 | IPR002487 | MADS-box | Transcription factor, K-box |
| FgenesH.VV78X161110.7_1 | IPR002487 | MADS-box | Transcription factor, K-box |
| FgenesH.VV78X161752.29_1 | IPR002487 | MADS-box | Transcription factor, K-box |
| FgenesH.VV78X164448.23_1 | IPR002487 | MADS-box | Transcription factor, K-box |
| FgenesH.VV78X168807.3_3 | IPR002487 | MADS-box | Transcription factor, K-box |
| FgenesH.VV78X173221.14_2 | IPR002487 | MADS-box | Transcription factor, K-box |
| FgenesH.VV78X173878.33_4 | IPR002487 | MADS-box | Transcription factor, K-box |
| FgenesH.VV78X174008.4_6 | IPR002487 | MADS-box | Transcription factor, K-box |
| FgenesH.VV78X181169.22_4 | IPR002100 | MADS-box | Transcription factor, MADS-box |
| FgenesH.VV78X182805.4_1 | IPR002487 | MADS-box | Transcription factor, K-box |
| FgenesH.VV78X183599.12_2 | IPR002487 | MADS-box | Transcription factor, K-box |
| FgenesH.VV78X183906.11_5 | IPR002487 | MADS-box | Transcription factor, K-box |
| FgenesH.VV78X184680.18_3 | IPR002487 | MADS-box | Transcription factor, K-box |
| FgenesH.VV78X187551.11_1 | IPR002487 | MADS-box | Transcription factor, K-box |
| FgenesH.VV78X189813.4_2 | IPR002487 | MADS-box | Transcription factor, K-box |
| FgenesH.VV78X196859.11_2 | IPR002487 | MADS-box | Transcription factor, K-box |
| FgenesH.VV78X197160.15_1 | IPR002487 | MADS-box | Transcription factor, K-box |
| FgenesH.VV78X197194.16_2 | IPR002487 | MADS-box | Transcription factor, K-box |
| FgenesH.VV78X197380.21_1 | IPR002487 | MADS-box | Transcription factor, K-box |
| FgenesH.VV78X200957.8_1 | IPR002487 | MADS-box | Transcription factor, K-box |
| FgenesH.VV78X201719.38_2 | IPR002487 | MADS-box | Transcription factor, K-box |
| FgenesH.VV78X205449.7_2 | IPR002487 | MADS-box | Transcription factor, K-box |
| FgenesH.VV78X206979.2_2 | IPR002487 | MADS-box | Transcription factor, K-box |
| FgenesH.VV78X207817.3_3 | IPR002487 | MADS-box | Transcription factor, K-box |
| FgenesH.VV78X207836.12_3 | IPR002487 | MADS-box | Transcription factor, K-box |
| FgenesH.VV78X209150.6_3 | IPR002487 | MADS-box | Transcription factor, K-box |
| FgenesH.VV78X216759.19_1 | IPR002487 | MADS-box | Transcription factor, K-box |
| FgenesH.VV78X221210.20_4 | IPR002487 | MADS-box | Transcription factor, K-box |
| FgenesH.VV78X223201.11_2 | IPR002487 | MADS-box | Transcription factor, K-box |
| FgenesH.VV78X226696.2_1 | IPR002487 | MADS-box | Transcription factor, K-box |
| FgenesH.VV78X227841.3_2 | IPR002487 | MADS-box | Transcription factor, K-box |
| FgenesH.VV78X231140.8_5 | IPR002487 | MADS-box | Transcription factor, K-box |
| FgenesH.VV78X232794.5_1 | IPR002487 | MADS-box | Transcription factor, K-box |
| FgenesH.VV78X236190.6_1 | IPR002487 | MADS-box | Transcription factor, K-box |
| FgenesH.VV78X237309.23_2 | IPR002487 | MADS-box | Transcription factor, K-box |
| FgenesH.VV78X247183.3_2 | IPR002487 | MADS-box | Transcription factor, K-box |
| FgenesH.VV78X247504.29_2 | IPR002487 | MADS-box | Transcription factor, K-box |
| FgenesH.VV78X249052.7_13 | IPR002487 | MADS-box | Transcription factor, K-box |
| FgenesH.VV78X252678.5_5 | IPR002100 | MADS-box | Transcription factor, MADS-box |
| FgenesH.VV78X253224.9_1 | IPR002487 | MADS-box | Transcription factor, K-box |
| FgenesH.VV78X253426.21_1 | IPR002487 | MADS-box | Transcription factor, K-box |
| FgenesH.VV78X254161.10_2 | IPR002487 | MADS-box | Transcription factor, K-box |
| FgenesH.VV78X254698.5_1 | IPR002487 | MADS-box | Transcription factor, K-box |
| FgenesH.VV78X255663.12_3 | IPR002487 | MADS-box | Transcription factor, K-box |
| FgenesH.VV78X257611.5_1 | IPR002487 | MADS-box | Transcription factor, K-box |
| FgenesH.VV78X257611.5_2 | IPR002100 | MADS-box | Transcription factor, MADS-box |
| FgenesH.VV78X257611.5_3 | IPR002100 | MADS-box | Transcription factor, MADS-box |
| FgenesH.VV78X264232.7_2 | IPR002487 | MADS-box | Transcription factor, K-box |
| FgenesH.VV78X266421.7_2 | IPR002487 | MADS-box | Transcription factor, K-box |
| FgenesH.VV78X267210.11_2 | IPR002487 | MADS-box | Transcription factor, K-box |
| FgenesH.VV78X268842.18_6 | IPR002487 | MADS-box | Transcription factor, K-box |
| FgenesH.VV78X269991.9_3 | IPR002487 | MADS-box | Transcription factor, K-box |
| FgenesH.VV78X275520.7_1 | IPR002487 | MADS-box | Transcription factor, K-box |
| FgenesH.VV78X276235.4_3 | IPR002487 | MADS-box | Transcription factor, K-box |
| FgenesH.VV79X000559.3_4 | IPR002487 | MADS-box | Transcription factor, K-box |
| FgenesH.VV79X001348.2_2 | IPR002487 | MADS-box | Transcription factor, K-box |
| FgenesH.VV79X002193.4_1 | IPR002487 | MADS-box | Transcription factor, K-box |
| FgenesH.VV79X003118.2_2 | IPR002100 | MADS-box | Transcription factor, MADS-box |
| FgenesH.VV79X003667.3_2 | IPR002487 | MADS-box | Transcription factor, K-box |
| FgenesH.VV79X006609.2_1 | IPR002100 | MADS-box | Transcription factor, MADS-box |
| Glimmer1.VV78X076297.14_2 | IPR002100 | MADS-box | Transcription factor, MADS-box |
| Glimmer1.VV78X180501.7_2 | IPR002487 | MADS-box | Transcription factor, K-box |
| Glimmer1.VV78X271525.3_2 | IPR002487 | MADS-box | Transcription factor, K-box |
| Sim4.aln-A06004308 | IPR002487 | MADS-box | Transcription factor, K-box |
| Sim4.aln-A06007119 | IPR002487 | MADS-box | Transcription factor, K-box |
| Sim4.aln-A06010180 | IPR002487 | MADS-box | Transcription factor, K-box |
| Sim4.aln-A06010662 | IPR002100 | MADS-box | Transcription factor, MADS-box |
| Sim4.aln-A06012809 | IPR002487 | MADS-box | Transcription factor, K-box |
| Sim4.aln-A06015842 | IPR002100 | MADS-box | Transcription factor, MADS-box |
| Sim4.aln-A06016402 | IPR002487 | MADS-box | Transcription factor, K-box |
| Sim4.aln-A06018467 | IPR002487 | MADS-box | Transcription factor, K-box |
| Sim4.aln-TCVV000149 | IPR002487 | MADS-box | Transcription factor, K-box |
| Sim4.aln-TCVV000238 | IPR002487 | MADS-box | Transcription factor, K-box |
| Sim4.aln-TCVV000379 | IPR002487 | MADS-box | Transcription factor, K-box |
| Sim4.aln-TCVV000392 | IPR002487 | MADS-box | Transcription factor, K-box |
| Sim4.aln-TCVV000587 | IPR002487 | MADS-box | Transcription factor, K-box |
| Sim4.aln-TCVV001283 | IPR002487 | MADS-box | Transcription factor, K-box |
| Sim4.aln-TCVV002216 | IPR002487 | MADS-box | Transcription factor, K-box |
| Sim4.aln-TCVV002732 | IPR002487 | MADS-box | Transcription factor, K-box |
| Sim4.aln-TCVV002737 | IPR002487 | MADS-box | Transcription factor, K-box |
| Sim4.aln-TCVV002886 | IPR002487 | MADS-box | Transcription factor, K-box |
| Sim4.aln-TCVV003044 | IPR002487 | MADS-box | Transcription factor, K-box |
| Sim4.aln-TCVV003389 | IPR002487 | MADS-box | Transcription factor, K-box |
| Sim4.aln-TCVV003413 | IPR002487 | MADS-box | Transcription factor, K-box |
| Sim4.aln-TCVV004103 | IPR002487 | MADS-box | Transcription factor, K-box |
| Sim4.aln-TCVV004432 | IPR002487 | MADS-box | Transcription factor, K-box |
| Sim4.aln-TCVV004709 | IPR002487 | MADS-box | Transcription factor, K-box |
| Sim4.aln-TCVV004900 | IPR002487 | MADS-box | Transcription factor, K-box |
| Sim4.aln-TCVV004967 | IPR002100 | MADS-box | Transcription factor, MADS-box |
| Sim4.aln-TCVV005204 | IPR002487 | MADS-box | Transcription factor, K-box |
| Sim4.aln-TCVV005477 | IPR002487 | MADS-box | Transcription factor, K-box |
| Sim4.aln-TCVV005968 | IPR002487 | MADS-box | Transcription factor, K-box |
| Sim4.aln-TCVV007083 | IPR002487 | MADS-box | Transcription factor, K-box |
| Sim4.aln-TCVV007089 | IPR002487 | MADS-box | Transcription factor, K-box |
| Sim4.aln-TCVV007339 | IPR002487 | MADS-box | Transcription factor, K-box |
| Sim4.aln-TCVV007720 | IPR002487 | MADS-box | Transcription factor, K-box |
| Sim4.aln-TCVV007837 | IPR002487 | MADS-box | Transcription factor, K-box |
| Sim4.aln-TCVV008124 | IPR002487 | MADS-box | Transcription factor, K-box |
| Sim4.aln-TCVV008379 | IPR002487 | MADS-box | Transcription factor, K-box |
| Sim4.aln-TCVV008721 | IPR002487 | MADS-box | Transcription factor, K-box |
| Sim4.aln-TCVV008875 | IPR002487 | MADS-box | Transcription factor, K-box |
| Sim4.aln-TCVV009164 | IPR002487 | MADS-box | Transcription factor, K-box |
| Sim4.aln-TCVV009767 | IPR002487 | MADS-box | Transcription factor, K-box |
| Sim4.aln-TCVV010395 | IPR002487 | MADS-box | Transcription factor, K-box |
| Sim4.aln-TCVV012190 | IPR002487 | MADS-box | Transcription factor, K-box |
| Sim4.aln-TCVV012585 | IPR002487 | MADS-box | Transcription factor, K-box |
| Sim4.aln-TCVV013087 | IPR002487 | MADS-box | Transcription factor, K-box |
| Sim4.aln-TCVV013240 | IPR002487 | MADS-box | Transcription factor, K-box |
| Sim4.aln-TCVV013972 | IPR002487 | MADS-box | Transcription factor, K-box |
| Sim4.aln-TCVV014067 | IPR002487 | MADS-box | Transcription factor, K-box |
| Sim4.aln-TCVV015011 | IPR002487 | MADS-box | Transcription factor, K-box |
| Sim4.aln-TCVV015050 | IPR002487 | MADS-box | Transcription factor, K-box |
| Sim4.aln-TCVV015853 | IPR002100 | MADS-box | Transcription factor, MADS-box |
| Sim4.aln-TCVV016269 | IPR002487 | MADS-box | Transcription factor, K-box |
| Sim4.aln-TCVV016471 | IPR002487 | MADS-box | Transcription factor, K-box |
| Sim4.aln-TCVV016771 | IPR002487 | MADS-box | Transcription factor, K-box |
| Sim4.aln-TCVV016893 | IPR002487 | MADS-box | Transcription factor, K-box |
| Sim4.aln-TCVV017291 | IPR002487 | MADS-box | Transcription factor, K-box |
| Sim4.aln-TCVV017318 | IPR002487 | MADS-box | Transcription factor, K-box |
| Sim4.aln-TCVV018040 | IPR002487 | MADS-box | Transcription factor, K-box |
| Sim4.aln-TCVV019373 | IPR002487 | MADS-box | Transcription factor, K-box |
| Sim4.aln-TCVV020583 | IPR002487 | MADS-box | Transcription factor, K-box |
| Sim4.aln-TCVV022149 | IPR002487 | MADS-box | Transcription factor, K-box |
| Sim4.aln-TCVV022785 | IPR002487 | MADS-box | Transcription factor, K-box |
| Sim4.aln-TCVV023290 | IPR002487 | MADS-box | Transcription factor, K-box |
| Sim4.aln-TCVV023838 | IPR002487 | MADS-box | Transcription factor, K-box |
| Twinscan1.VV78X041959.2_4 | IPR002100 | MADS-box | Transcription factor, MADS-box |
| Twinscan1.VV78X090723.10_2 | IPR002487 | MADS-box | Transcription factor, K-box |
| Twinscan1.VV78X180501.7_3 | IPR002487 | MADS-box | Transcription factor, K-box |
| Twinscan1.VV78X271525.3_3 | IPR002487 | MADS-box | Transcription factor, K-box |
| Sim4.aln-A06014412 | IPR001387 | MBF1 | Helix-turn-helix type 3 |
| Sim4.aln-TCVV004597 | IPR001387 | MBF1 | Helix-turn-helix type 3 |
| FgenesH.VV78X000436.15_3 | IPR014778 | Myb | Myb, DNA-binding |
| FgenesH.VV78X000502.3_4 | IPR014778 | Myb | Myb, DNA-binding |
| FgenesH.VV78X001301.9_8 | IPR001005 | Myb | Myb, DNA-binding |
| FgenesH.VV78X003670.12_4 | IPR014778 | Myb | Myb, DNA-binding |
| FgenesH.VV78X003790.9_1 | IPR001005 | Myb | Myb, DNA-binding |
| FgenesH.VV78X009364.25_3 | IPR001005 | Myb | Myb, DNA-binding |
| FgenesH.VV78X011584.12_2 | IPR001005 | Myb | Myb, DNA-binding |
| FgenesH.VV78X013095.7_4 | IPR014778 | Myb | Myb, DNA-binding |
| FgenesH.VV78X014489.6_2 | IPR001005 | Myb | Myb, DNA-binding |
| FgenesH.VV78X016316.7_3 | IPR014778 | Myb | Myb, DNA-binding |
| FgenesH.VV78X017300.10_4 | IPR007526 | Myb related | SWIRM |
| FgenesH.VV78X022861.5_1 | IPR014778 | Myb | Myb, DNA-binding |
| FgenesH.VV78X023223.6_1 | IPR001005 | Myb | Myb, DNA-binding |
| FgenesH.VV78X024328.12_4 | IPR001005 | Myb | Myb, DNA-binding |
| FgenesH.VV78X024662.19_2 | IPR001005 | Myb | Myb, DNA-binding |
| FgenesH.VV78X026725.11_1 | IPR002487 | Myb | Myb, DNA-binding |
| FgenesH.VV78X026982.42_1 | IPR014778 | Myb | Myb, DNA-binding |
| FgenesH.VV78X027582.5_4 | IPR001005 | Myb | Myb, DNA-binding |
| FgenesH.VV78X035406.3_3 | IPR001005 | Myb | Myb, DNA-binding |
| FgenesH.VV78X036071.17_2 | IPR001005 | Myb | Myb, DNA-binding |
| FgenesH.VV78X037068.18_3 | IPR014778 | Myb | Myb, DNA-binding |
| FgenesH.VV78X039467.9_2 | IPR001005 | Myb | Myb, DNA-binding |
| FgenesH.VV78X041439.7_1 | IPR014778 | Myb | Myb, DNA-binding |
| FgenesH.VV78X043049.23_4 | IPR001005 | Myb | Myb, DNA-binding |
| FgenesH.VV78X043971.11_2 | IPR014778 | Myb | Myb, DNA-binding |
| FgenesH.VV78X044419.13_1 | IPR001005 | Myb | Myb, DNA-binding |
| FgenesH.VV78X045205.18_4 | IPR001005 | Myb | Myb, DNA-binding |
| FgenesH.VV78X045400.10_1 | IPR014778 | Myb | Myb, DNA-binding |
| FgenesH.VV78X045699.17_3 | IPR000433 | Myb related | Zinc finger, ZZ-type |
| FgenesH.VV78X046090.12_1 | IPR014778 | Myb | Myb, DNA-binding |
| FgenesH.VV78X046097.11_3 | IPR014778 | Myb | Myb, DNA-binding |
| FgenesH.VV78X046554.2_2 | IPR001005 | Myb | Myb, DNA-binding |
| FgenesH.VV78X048728.3_1 | IPR001005 | Myb | Myb, DNA-binding |
| FgenesH.VV78X048992.14_1 | IPR001005 | Myb | Myb, DNA-binding |
| FgenesH.VV78X050513.3_2 | IPR001005 | Myb | Myb, DNA-binding |
| FgenesH.VV78X050589.7_1 | IPR014778 | Myb | Myb, DNA-binding |
| FgenesH.VV78X050611.9_2 | IPR001005 | Myb | Myb, DNA-binding |
| FgenesH.VV78X051134.5_1 | IPR014778 | Myb | Myb, DNA-binding |
| FgenesH.VV78X053495.4_1 | IPR014778 | Myb | Myb, DNA-binding |
| FgenesH.VV78X059648.3_1 | IPR014778 | Myb | Myb, DNA-binding |
| FgenesH.VV78X059861.10_1 | IPR014778 | Myb | Myb, DNA-binding |
| FgenesH.VV78X060620.5_1 | IPR001005 | Myb | Myb, DNA-binding |
| FgenesH.VV78X060807.3_1 | IPR001005 | Myb | Myb, DNA-binding |
| FgenesH.VV78X061938.9_2 | IPR001005 | Myb | Myb, DNA-binding |
| FgenesH.VV78X070068.18_2 | IPR014778 | Myb | Myb, DNA-binding |
| FgenesH.VV78X070152.6_2 | IPR014778 | Myb | Myb, DNA-binding |
| FgenesH.VV78X073501.7_1 | IPR014778 | Myb | Myb, DNA-binding |
| FgenesH.VV78X081348.5_1 | IPR001005 | Myb | Myb, DNA-binding |
| FgenesH.VV78X081495.24_4 | IPR001005 | Myb | Myb, DNA-binding |
| FgenesH.VV78X087069.17_5 | IPR014778 | Myb | Myb, DNA-binding |
| FgenesH.VV78X089529.29_4 | IPR007526 | Myb related | SWIRM |
| FgenesH.VV78X090374.7_1 | IPR014778 | Myb | Myb, DNA-binding |
| FgenesH.VV78X091652.3_1 | IPR001005 | Myb | Myb, DNA-binding |
| FgenesH.VV78X092018.9_3 | IPR001005 | Myb | Myb, DNA-binding |
| FgenesH.VV78X092169.11_4 | IPR014778 | Myb | Myb, DNA-binding |
| FgenesH.VV78X096618.37_6 | IPR014778 | Myb | Myb, DNA-binding |
| FgenesH.VV78X097283.10_1 | IPR014778 | Myb | Myb, DNA-binding |
| FgenesH.VV78X097971.15_2 | IPR014778 | Myb | Myb, DNA-binding |
| FgenesH.VV78X102680.7_3 | IPR001005 | Myb | Myb, DNA-binding |
| FgenesH.VV78X103470.3_1 | IPR014778 | Myb | Myb, DNA-binding |
| FgenesH.VV78X104670.13_1 | IPR014778 | Myb | Myb, DNA-binding |
| FgenesH.VV78X108547.35_2 | IPR014778 | Myb | Myb, DNA-binding |
| FgenesH.VV78X111537.8_2 | IPR014778 | Myb | Myb, DNA-binding |
| FgenesH.VV78X113340.17_5 | IPR014778 | Myb | Myb, DNA-binding |
| FgenesH.VV78X118710.15_1 | IPR014778 | Myb | Myb, DNA-binding |
| FgenesH.VV78X119953.5_1 | IPR014778 | Myb | Myb, DNA-binding |
| FgenesH.VV78X120914.8_1 | IPR014778 | Myb | Myb, DNA-binding |
| FgenesH.VV78X122096.15_3 | IPR001005 | Myb | Myb, DNA-binding |
| FgenesH.VV78X123743.4_1 | IPR014778 | Myb | Myb, DNA-binding |
| FgenesH.VV78X124245.8_1 | IPR014778 | Myb | Myb, DNA-binding |
| FgenesH.VV78X124406.5_2 | IPR014778 | Myb | Myb, DNA-binding |
| FgenesH.VV78X126725.5_3 | IPR001005 | Myb | Myb, DNA-binding |
| FgenesH.VV78X127344.6_1 | IPR001005 | Myb | Myb, DNA-binding |
| FgenesH.VV78X130291.4_2 | IPR014778 | Myb | Myb, DNA-binding |
| FgenesH.VV78X131360.7_4 | IPR001005 | Myb | Myb, DNA-binding |
| FgenesH.VV78X132913.5_2 | IPR014778 | Myb | Myb, DNA-binding |
| FgenesH.VV78X134816.19_2 | IPR014778 | Myb | Myb, DNA-binding |
| FgenesH.VV78X137684.6_2 | IPR014778 | Myb | Myb, DNA-binding |
| FgenesH.VV78X138380.7_1 | IPR001005 | Myb | Myb, DNA-binding |
| FgenesH.VV78X140551.2_2 | IPR001005 | Myb | Myb, DNA-binding |
| FgenesH.VV78X140903.16_14 | IPR001005 | Myb | Myb, DNA-binding |
| FgenesH.VV78X142082.8_2 | IPR001005 | Myb | Myb, DNA-binding |
| FgenesH.VV78X142875.16_1 | IPR014778 | Myb | Myb, DNA-binding |
| FgenesH.VV78X145138.21_2 | IPR014778 | Myb | Myb, DNA-binding |
| FgenesH.VV78X145228.3_2 | IPR001005 | Myb | Myb, DNA-binding |
| FgenesH.VV78X152149.3_2 | IPR001005 | Myb | Myb, DNA-binding |
| FgenesH.VV78X153774.6_3 | IPR001005 | Myb | Myb, DNA-binding |
| FgenesH.VV78X155042.5_4 | IPR014778 | Myb | Myb, DNA-binding |
| FgenesH.VV78X155772.10_3 | IPR014778 | Myb | Myb, DNA-binding |
| FgenesH.VV78X157080.2_6 | IPR014778 | Myb | Myb, DNA-binding |
| FgenesH.VV78X159476.15_4 | IPR001005 | Myb | Myb, DNA-binding |
| FgenesH.VV78X160007.12_2 | IPR001005 | Myb | Myb, DNA-binding |
| FgenesH.VV78X167165.2_1 | IPR001005 | Myb | Myb, DNA-binding |
| FgenesH.VV78X167727.5_2 | IPR001005 | Myb | Myb, DNA-binding |
| FgenesH.VV78X167758.6_3 | IPR014778 | Myb | Myb, DNA-binding |
| FgenesH.VV78X169288.10_3 | IPR001005 | Myb | Myb, DNA-binding |
| FgenesH.VV78X169943.11_3 | IPR001005 | Myb | Myb, DNA-binding |
| FgenesH.VV78X172325.7_2 | IPR014778 | Myb | Myb, DNA-binding |
| FgenesH.VV78X174581.13_1 | IPR001005 | Myb | Myb, DNA-binding |
| FgenesH.VV78X178746.5_2 | IPR014778 | Myb | Myb, DNA-binding |
| FgenesH.VV78X179323.3_1 | IPR014778 | Myb | Myb, DNA-binding |
| FgenesH.VV78X183017.13_2 | IPR001005 | Myb | Myb, DNA-binding |
| FgenesH.VV78X183580.6_2 | IPR014778 | Myb | Myb, DNA-binding |
| FgenesH.VV78X185458.11_2 | IPR005818 | Myb related | Histone H1/H5 |
| FgenesH.VV78X185711.26_4 | IPR014778 | Myb | Myb, DNA-binding |
| FgenesH.VV78X187119.4_1 | IPR014778 | Myb | Myb, DNA-binding |
| FgenesH.VV78X187962.10_3 | IPR001005 | Myb | Myb, DNA-binding |
| FgenesH.VV78X189718.9_3 | IPR014778 | Myb | Myb, DNA-binding |
| FgenesH.VV78X197053.5_3 | IPR005818 | Myb related | Histone H1/H5 |
| FgenesH.VV78X200476.6_1 | IPR001005 | Myb | Myb, DNA-binding |
| FgenesH.VV78X200574.4_5 | IPR001005 | Myb | Myb, DNA-binding |
| FgenesH.VV78X203929.11_5 | IPR014778 | Myb | Myb, DNA-binding |
| FgenesH.VV78X204561.26_3 | IPR014778 | Myb | Myb, DNA-binding |
| FgenesH.VV78X207065.35_1 | IPR014778 | Myb | Myb, DNA-binding |
| FgenesH.VV78X207734.6_1 | IPR014778 | Myb | Myb, DNA-binding |
| FgenesH.VV78X221882.10_5 | IPR014778 | Myb | Myb, DNA-binding |
| FgenesH.VV78X223624.13_1 | IPR010561 | Myb related | DIRP |
| FgenesH.VV78X225435.4_2 | IPR001005 | Myb | Myb, DNA-binding |
| FgenesH.VV78X228609.9_4 | IPR001005 | Myb | Myb, DNA-binding |
| FgenesH.VV78X229062.6_7 | IPR001005 | Myb | Myb, DNA-binding |
| FgenesH.VV78X231367.10_1 | IPR001005 | Myb | Myb, DNA-binding |
| FgenesH.VV78X232607.28_2 | IPR001005 | Myb | Myb, DNA-binding |
| FgenesH.VV78X235683.20_1 | IPR001005 | Myb | Myb, DNA-binding |
| FgenesH.VV78X238506.24_3 | IPR001005 | Myb | Myb, DNA-binding |
| FgenesH.VV78X241236.14_4 | IPR014778 | Myb | Myb, DNA-binding |
| FgenesH.VV78X241396.15_3 | IPR014778 | Myb | Myb, DNA-binding |
| FgenesH.VV78X242076.6_2 | IPR014778 | Myb | Myb, DNA-binding |
| FgenesH.VV78X242367.25_1 | IPR001005 | Myb | Myb, DNA-binding |
| FgenesH.VV78X249052.7_7 | IPR007526 | Myb related | SWIRM |
| FgenesH.VV78X250929.7_1 | IPR005818 | Myb related | Histone H1/H5 |
| FgenesH.VV78X253664.11_2 | IPR000433 | Myb related | Zinc finger, ZZ-type |
| FgenesH.VV78X254247.10_2 | IPR001005 | Myb | Myb, DNA-binding |
| FgenesH.VV78X256089.20_1 | IPR001005 | Myb | Myb, DNA-binding |
| FgenesH.VV78X259940.16_4 | IPR001005 | Myb | Myb, DNA-binding |
| FgenesH.VV78X263055.10_1 | IPR014778 | Myb | Myb, DNA-binding |
| FgenesH.VV78X266838.11_3 | IPR001005 | Myb | Myb, DNA-binding |
| FgenesH.VV78X269243.4_2 | IPR014778 | Myb | Myb, DNA-binding |
| FgenesH.VV78X269243.4_3 | IPR014778 | Myb | Myb, DNA-binding |
| FgenesH.VV78X269352.12_1 | IPR001005 | Myb | Myb, DNA-binding |
| FgenesH.VV78X272111.5_5 | IPR014778 | Myb | Myb, DNA-binding |
| FgenesH.VV78X276432.20_4 | IPR014778 | Myb | Myb, DNA-binding |
| FgenesH.VV79X000441.2_1 | IPR001005 | Myb | Myb, DNA-binding |
| FgenesH.VV79X001214.4_1 | IPR001005 | Myb | Myb, DNA-binding |
| FgenesH.VV79X001945.2_2 | IPR014778 | Myb | Myb, DNA-binding |
| FgenesH.VV79X002449.2_1 | IPR014778 | Myb | Myb, DNA-binding |
| FgenesH.VV79X003101.4_5 | IPR001005 | Myb | Myb, DNA-binding |
| FgenesH.VV79X004925.2_1 | IPR014778 | Myb | Myb, DNA-binding |
| FgenesH.VV79X006335.3_1 | IPR014778 | Myb | Myb, DNA-binding |
| Glimmer1.VV78X017201.5_2 | IPR014778 | Myb | Myb, DNA-binding |
| Glimmer1.VV78X099246.24_15 | IPR014778 | Myb | Myb, DNA-binding |
| Glimmer1.VV78X099344.10_2 | IPR014778 | Myb | Myb, DNA-binding |
| Glimmer1.VV78X138990.3_2 | IPR001005 | Myb | Myb, DNA-binding |
| Glimmer1.VV78X152339.5_2 | IPR014778 | Myb | Myb, DNA-binding |
| Glimmer1.VV78X168958.5_2 | IPR014778 | Myb | Myb, DNA-binding |
| Glimmer1.VV78X207150.8_2 | IPR001005 | Myb | Myb, DNA-binding |
| Glimmer1.VV78X228068.5_1 | IPR014778 | Myb | Myb, DNA-binding |
| Glimmer1.VV78X268871.2_2 | IPR001005 | Myb | Myb, DNA-binding |
| Sim4.aln-A06001279 | IPR014778 | Myb | Myb, DNA-binding |
| Sim4.aln-A06002268 | IPR014778 | Myb | Myb, DNA-binding |
| Sim4.aln-A06002833 | IPR001005 | Myb | Myb, DNA-binding |
| Sim4.aln-A06002996 | IPR014778 | Myb | Myb, DNA-binding |
| Sim4.aln-A06003778 | IPR005818 | Myb related | Histone H1/H5 |
| Sim4.aln-A06006354 | IPR014778 | Myb | Myb, DNA-binding |
| Sim4.aln-A06009879 | IPR014778 | Myb | Myb, DNA-binding |
| Sim4.aln-A06011092 | IPR001005 | Myb | Myb, DNA-binding |
| Sim4.aln-A06011147 | IPR014778 | Myb | Myb, DNA-binding |
| Sim4.aln-A06011590 | IPR014778 | Myb | Myb, DNA-binding |
| Sim4.aln-A06012868 | IPR007526 | Myb related | SWIRM |
| Sim4.aln-A06013133 | IPR014778 | Myb | Myb, DNA-binding |
| Sim4.aln-A06013379 | IPR001005 | Myb | Myb, DNA-binding |
| Sim4.aln-A06014431 | IPR014778 | Myb | Myb, DNA-binding |
| Sim4.aln-A06015138 | IPR014778 | Myb | Myb, DNA-binding |
| Sim4.aln-A06016659 | IPR001005 | Myb | Myb, DNA-binding |
| Sim4.aln-A06017199 | IPR001005 | Myb | Myb, DNA-binding |
| Sim4.aln-A06017504 | IPR014778 | Myb | Myb, DNA-binding |
| Sim4.aln-A06017664 | IPR014778 | Myb | Myb, DNA-binding |
| Sim4.aln-A06018041 | IPR014778 | Myb | Myb, DNA-binding |
| Sim4.aln-A06018051 | IPR014778 | Myb | Myb, DNA-binding |
| Sim4.aln-TCVV000028 | IPR014778 | Myb | Myb, DNA-binding |
| Sim4.aln-TCVV000077 | IPR014778 | Myb | Myb, DNA-binding |
| Sim4.aln-TCVV000256 | IPR005818 | Myb related | Histone H1/H5 |
| Sim4.aln-TCVV000327 | IPR001005 | Myb | Myb, DNA-binding |
| Sim4.aln-TCVV000386 | IPR014778 | Myb | Myb, DNA-binding |
| Sim4.aln-TCVV000560 | IPR014778 | Myb | Myb, DNA-binding |
| Sim4.aln-TCVV000980 | IPR014778 | Myb | Myb, DNA-binding |
| Sim4.aln-TCVV001187 | IPR014778 | Myb | Myb, DNA-binding |
| Sim4.aln-TCVV001194 | IPR014778 | Myb | Myb, DNA-binding |
| Sim4.aln-TCVV001625 | IPR001005 | Myb | Myb, DNA-binding |
| Sim4.aln-TCVV001713 | IPR014778 | Myb | Myb, DNA-binding |
| Sim4.aln-TCVV001863 | IPR014778 | Myb | Myb, DNA-binding |
| Sim4.aln-TCVV001937 | IPR001005 | Myb | Myb, DNA-binding |
| Sim4.aln-TCVV002144 | IPR014778 | Myb | Myb, DNA-binding |
| Sim4.aln-TCVV002396 | IPR000433 | Myb related | Zinc finger, ZZ-type |
| Sim4.aln-TCVV002435 | IPR014778 | Myb | Myb, DNA-binding |
| Sim4.aln-TCVV002506 | IPR014778 | Myb | Myb, DNA-binding |
| Sim4.aln-TCVV003023 | IPR014778 | Myb | Myb, DNA-binding |
| Sim4.aln-TCVV003182 | IPR001005 | Myb | Myb, DNA-binding |
| Sim4.aln-TCVV003450 | IPR001005 | Myb | Myb, DNA-binding |
| Sim4.aln-TCVV003689 | IPR014778 | Myb | Myb, DNA-binding |
| Sim4.aln-TCVV003770 | IPR001005 | Myb | Myb, DNA-binding |
| Sim4.aln-TCVV003828 | IPR014778 | Myb | Myb, DNA-binding |
| Sim4.aln-TCVV003917 | IPR001005 | Myb | Myb, DNA-binding |
| Sim4.aln-TCVV004083 | IPR014778 | Myb | Myb, DNA-binding |
| Sim4.aln-TCVV004250 | IPR014778 | Myb | Myb, DNA-binding |
| Sim4.aln-TCVV004583 | IPR005818 | Myb related | Histone H1/H5 |
| Sim4.aln-TCVV004652 | IPR014778 | Myb | Myb, DNA-binding |
| Sim4.aln-TCVV004662 | IPR001005 | Myb | Myb, DNA-binding |
| Sim4.aln-TCVV004713 | IPR014778 | Myb | Myb, DNA-binding |
| Sim4.aln-TCVV004922 | IPR014778 | Myb | Myb, DNA-binding |
| Sim4.aln-TCVV005024 | IPR007526 | Myb related | SWIRM |
| Sim4.aln-TCVV005250 | IPR014778 | Myb | Myb, DNA-binding |
| Sim4.aln-TCVV005418 | IPR001005 | Myb | Myb, DNA-binding |
| Sim4.aln-TCVV005443 | IPR014778 | Myb | Myb, DNA-binding |
| Sim4.aln-TCVV005807 | IPR014778 | Myb | Myb, DNA-binding |
| Sim4.aln-TCVV005846 | IPR001005 | Myb | Myb, DNA-binding |
| Sim4.aln-TCVV005890 | IPR014778 | Myb | Myb, DNA-binding |
| Sim4.aln-TCVV006298 | IPR014778 | Myb | Myb, DNA-binding |
| Sim4.aln-TCVV006470 | IPR001005 | Myb | Myb, DNA-binding |
| Sim4.aln-TCVV007003 | IPR001005 | myb | SANT, DNA-binding |
| Sim4.aln-TCVV007049 | IPR001005 | Myb | Myb, DNA-binding |
| Sim4.aln-TCVV007176 | IPR014778 | Myb | Myb, DNA-binding |
| Sim4.aln-TCVV007487 | IPR014778 | Myb | Myb, DNA-binding |
| Sim4.aln-TCVV007540 | IPR014778 | Myb | Myb, DNA-binding |
| Sim4.aln-TCVV007999 | IPR001005 | Myb | Myb, DNA-binding |
| Sim4.aln-TCVV008090 | IPR001005 | Myb | Myb, DNA-binding |
| Sim4.aln-TCVV008223 | IPR005818 | Myb related | Histone H1/H5 |
| Sim4.aln-TCVV008641 | IPR014778 | Myb | Myb, DNA-binding |
| Sim4.aln-TCVV009033 | IPR014778 | Myb | Myb, DNA-binding |
| Sim4.aln-TCVV009136 | IPR005818 | Myb related | Histone H1/H5 |
| Sim4.aln-TCVV009545 | IPR001005 | Myb | Myb, DNA-binding |
| Sim4.aln-TCVV009595 | IPR014778 | Myb | Myb, DNA-binding |
| Sim4.aln-TCVV010264 | IPR001005 | Myb | Myb, DNA-binding |
| Sim4.aln-TCVV010308 | IPR014778 | Myb | Myb, DNA-binding |
| Sim4.aln-TCVV011208 | IPR001005 | myb | SANT, DNA-binding |
| Sim4.aln-TCVV011793 | IPR001005 | Myb | Myb, DNA-binding |
| Sim4.aln-TCVV011844 | IPR014778 | Myb | Myb, DNA-binding |
| Sim4.aln-TCVV011936 | IPR014778 | Myb | Myb, DNA-binding |
| Sim4.aln-TCVV012254 | IPR014778 | Myb | Myb, DNA-binding |
| Sim4.aln-TCVV013088 | IPR001005 | Myb | Myb, DNA-binding |
| Sim4.aln-TCVV013162 | IPR014778 | Myb | Myb, DNA-binding |
| Sim4.aln-TCVV013353 | IPR014778 | Myb | Myb, DNA-binding |
| Sim4.aln-TCVV013496 | IPR001005 | Myb | Myb, DNA-binding |
| Sim4.aln-TCVV013541 | IPR014778 | Myb | Myb, DNA-binding |
| Sim4.aln-TCVV014165 | IPR014778 | Myb | Myb, DNA-binding |
| Sim4.aln-TCVV015245 | IPR001005 | Myb | Myb, DNA-binding |
| Sim4.aln-TCVV015399 | IPR014778 | Myb | Myb, DNA-binding |
| Sim4.aln-TCVV015472 | IPR001005 | Myb | Myb, DNA-binding |
| Sim4.aln-TCVV016113 | IPR014778 | Myb | Myb, DNA-binding |
| Sim4.aln-TCVV016115 | IPR005818 | Myb related | Histone H1/H5 |
| Sim4.aln-TCVV016554 | IPR014778 | Myb | Myb, DNA-binding |
| Sim4.aln-TCVV016614 | IPR001005 | Myb | Myb, DNA-binding |
| Sim4.aln-TCVV016727 | IPR014778 | Myb | Myb, DNA-binding |
| Sim4.aln-TCVV016812 | IPR014778 | Myb | Myb, DNA-binding |
| Sim4.aln-TCVV016900 | IPR001005 | Myb | Myb, DNA-binding |
| Sim4.aln-TCVV017022 | IPR001005 | Myb | Myb, DNA-binding |
| Sim4.aln-TCVV017548 | IPR014778 | Myb | Myb, DNA-binding |
| Sim4.aln-TCVV017640 | IPR014778 | Myb | Myb, DNA-binding |
| Sim4.aln-TCVV017658 | IPR014778 | Myb | Myb, DNA-binding |
| Sim4.aln-TCVV019032 | IPR014778 | Myb | Myb, DNA-binding |
| Sim4.aln-TCVV019738 | IPR001005 | Myb | Myb, DNA-binding |
| Sim4.aln-TCVV020254 | IPR014778 | Myb | Myb, DNA-binding |
| Sim4.aln-TCVV021117 | IPR001005 | Myb | Myb, DNA-binding |
| Sim4.aln-TCVV021673 | IPR007526 | Myb related | SWIRM |
| Sim4.aln-TCVV021712 | IPR001005 | Myb | Myb, DNA-binding |
| Sim4.aln-TCVV021995 | IPR001005 | Myb | Myb, DNA-binding |
| Sim4.aln-TCVV022030 | IPR014778 | Myb | Myb, DNA-binding |
| Sim4.aln-TCVV022094 | IPR001005 | Myb | Myb, DNA-binding |
| Sim4.aln-TCVV022166 | IPR001005 | Myb | Myb, DNA-binding |
| Sim4.aln-TCVV023288 | IPR005818 | Myb related | Histone H1/H5 |
| Sim4.aln-TCVV024139 | IPR001005 | Myb | Myb, DNA-binding |
| Sim4.aln-TCVV024146 | IPR014778 | Myb | Myb, DNA-binding |
| Twinscan1.VV78X006594.2_7 | IPR014778 | Myb | Myb, DNA-binding |
| Twinscan1.VV78X024027.20_1 | IPR014778 | Myb | Myb, DNA-binding |
| Twinscan1.VV78X044640.12_7 | IPR001005 | Myb | Myb, DNA-binding |
| Twinscan1.VV78X095327.14_3 | IPR014778 | Myb | Myb, DNA-binding |
| Twinscan1.VV78X239782.46_7 | IPR014778 | Myb | Myb, DNA-binding |
| Twinscan1.VV78X269058.24_1 | IPR001005 | Myb | Myb, DNA-binding |
| Twinscan1.VV78X272111.5_9 | IPR001005 | myb | SANT, DNA-binding |
| Twinscan1.VV79X002487.2_6 | IPR001005 | Myb | Myb, DNA-binding |
| FgenesH.VV78X021591.4_2 | IPR002715 | NAC | Nascent polypeptide-associated complex NAC |
| FgenesH.VV78X037850.7_4 | IPR003441 | NAC | No apical meristem (NAM) protein |
| FgenesH.VV78X039610.6_2 | IPR003441 | Nac | No apical meristem (NAM) protein |
| FgenesH.VV78X045661.6_2 | IPR003441 | Nac | No apical meristem (NAM) protein |
| FgenesH.VV78X050837.6_2 | IPR003441 | Nac | No apical meristem (NAM) protein |
| FgenesH.VV78X055866.6_4 | IPR003441 | Nac | No apical meristem (NAM) protein |
| FgenesH.VV78X060572.10_3 | IPR003441 | Nac | No apical meristem (NAM) protein |
| FgenesH.VV78X065901.3_1 | IPR003441 | Nac | No apical meristem (NAM) protein |
| FgenesH.VV78X080122.17_1 | IPR003441 | Nac | No apical meristem (NAM) protein |
| FgenesH.VV78X095272.6_1 | IPR003441 | Nac | No apical meristem (NAM) protein |
| FgenesH.VV78X129887.25_3 | IPR003441 | Nac | No apical meristem (NAM) protein |
| FgenesH.VV78X131882.29_4 | IPR003441 | Nac | No apical meristem (NAM) protein |
| FgenesH.VV78X133673.15_1 | IPR003441 | Nac | No apical meristem (NAM) protein |
| FgenesH.VV78X135584.5_2 | IPR003441 | Nac | No apical meristem (NAM) protein |
| FgenesH.VV78X135841.6_1 | IPR003441 | Nac | No apical meristem (NAM) protein |
| FgenesH.VV78X140618.6_4 | IPR003441 | NAC | No apical meristem (NAM) protein |
| FgenesH.VV78X145383.32_2 | IPR003441 | NAC | No apical meristem (NAM) protein |
| FgenesH.VV78X151885.15_3 | IPR003441 | Nac | No apical meristem (NAM) protein |
| FgenesH.VV78X155772.10_4 | IPR003441 | Nac | No apical meristem (NAM) protein |
| FgenesH.VV78X171234.10_8 | IPR003441 | Nac | No apical meristem (NAM) protein |
| FgenesH.VV78X177830.4_2 | IPR003441 | NAC | No apical meristem (NAM) protein |
| FgenesH.VV78X179772.7_1 | IPR003441 | Nac | No apical meristem (NAM) protein |
| FgenesH.VV78X182260.9_1 | IPR003441 | Nac | No apical meristem (NAM) protein |
| FgenesH.VV78X184666.11_1 | IPR003441 | Nac | No apical meristem (NAM) protein |
| FgenesH.VV78X198301.8_3 | IPR003441 | NAC | No apical meristem (NAM) protein |
| FgenesH.VV78X202842.8_1 | IPR003441 | Nac | No apical meristem (NAM) protein |
| FgenesH.VV78X210061.3_6 | IPR003441 | NAC | No apical meristem (NAM) protein |
| FgenesH.VV78X211388.5_4 | IPR003441 | Nac | No apical meristem (NAM) protein |
| FgenesH.VV78X215902.12_2 | IPR003441 | Nac | No apical meristem (NAM) protein |
| FgenesH.VV78X219870.4_1 | IPR003441 | Nac | No apical meristem (NAM) protein |
| FgenesH.VV78X222766.12_4 | IPR003441 | Nac | No apical meristem (NAM) protein |
| FgenesH.VV78X227225.4_2 | IPR003441 | Nac | No apical meristem (NAM) protein |
| FgenesH.VV78X239632.6_1 | IPR003441 | Nac | No apical meristem (NAM) protein |
| FgenesH.VV78X241223.4_4 | IPR003441 | Nac | No apical meristem (NAM) protein |
| FgenesH.VV78X252678.5_1 | IPR003441 | Nac | No apical meristem (NAM) protein |
| FgenesH.VV78X266640.9_2 | IPR003441 | NAC | No apical meristem (NAM) protein |
| FgenesH.VV78X271653.5_1 | IPR003441 | Nac | No apical meristem (NAM) protein |
| FgenesH.VV78X272315.3_6 | IPR003441 | Nac | No apical meristem (NAM) protein |
| FgenesH.VV78X277513.23_3 | IPR003441 | NAC | No apical meristem (NAM) protein |
| Glimmer1.VV78X109532.13_5 | IPR003441 | Nac | No apical meristem (NAM) protein |
| Glimmer1.VV78X194222.8_1 | IPR003441 | Nac | No apical meristem (NAM) protein |
| Sim4.aln-A06005450 | IPR003441 | Nac | No apical meristem (NAM) protein |
| Sim4.aln-A06008427 | IPR003441 | Nac | No apical meristem (NAM) protein |
| Sim4.aln-A06012169 | IPR003441 | Nac | No apical meristem (NAM) protein |
| Sim4.aln-A06014555 | IPR002715 | NAC | Nascent polypeptide-associated complex NAC |
| Sim4.aln-A06014642 | IPR003441 | Nac | No apical meristem (NAM) protein |
| Sim4.aln-TCVV000441 | IPR003441 | Nac | No apical meristem (NAM) protein |
| Sim4.aln-TCVV001081 | IPR003441 | Nac | No apical meristem (NAM) protein |
| Sim4.aln-TCVV001493 | IPR003441 | Nac | No apical meristem (NAM) protein |
| Sim4.aln-TCVV001568 | IPR002715 | NAC | Nascent polypeptide-associated complex NAC |
| Sim4.aln-TCVV001661 | IPR003441 | Nac | No apical meristem (NAM) protein |
| Sim4.aln-TCVV001810 | IPR003441 | Nac | No apical meristem (NAM) protein |
| Sim4.aln-TCVV001975 | IPR003441 | Nac | No apical meristem (NAM) protein |
| Sim4.aln-TCVV002001 | IPR003441 | Nac | No apical meristem (NAM) protein |
| Sim4.aln-TCVV002830 | IPR003441 | Nac | No apical meristem (NAM) protein |
| Sim4.aln-TCVV003243 | IPR003441 | Nac | No apical meristem (NAM) protein |
| Sim4.aln-TCVV004114 | IPR003441 | Nac | No apical meristem (NAM) protein |
| Sim4.aln-TCVV004255 | IPR003441 | Nac | No apical meristem (NAM) protein |
| Sim4.aln-TCVV006183 | IPR003441 | Nac | No apical meristem (NAM) protein |
| Sim4.aln-TCVV008321 | IPR003441 | Nac | No apical meristem (NAM) protein |
| Sim4.aln-TCVV008473 | IPR003441 | Nac | No apical meristem (NAM) protein |
| Sim4.aln-TCVV008571 | IPR003441 | Nac | No apical meristem (NAM) protein |
| Sim4.aln-TCVV008966 | IPR003441 | Nac | No apical meristem (NAM) protein |
| Sim4.aln-TCVV009887 | IPR003441 | Nac | No apical meristem (NAM) protein |
| Sim4.aln-TCVV013689 | IPR003441 | Nac | No apical meristem (NAM) protein |
| Sim4.aln-TCVV014076 | IPR003441 | Nac | No apical meristem (NAM) protein |
| Sim4.aln-TCVV014421 | IPR003441 | Nac | No apical meristem (NAM) protein |
| Sim4.aln-TCVV015692 | IPR003441 | Nac | No apical meristem (NAM) protein |
| Sim4.aln-TCVV016009 | IPR003441 | Nac | No apical meristem (NAM) protein |
| Sim4.aln-TCVV017060 | IPR003441 | Nac | No apical meristem (NAM) protein |
| Sim4.aln-TCVV019039 | IPR003441 | Nac | No apical meristem (NAM) protein |
| Sim4.aln-TCVV019885 | IPR003441 | Nac | No apical meristem (NAM) protein |
| Sim4.aln-TCVV020279 | IPR003441 | Nac | No apical meristem (NAM) protein |
| Sim4.aln-TCVV021307 | IPR003441 | Nac | No apical meristem (NAM) protein |
| Sim4.aln-TCVV021886 | IPR003441 | Nac | No apical meristem (NAM) protein |
| Sim4.aln-TCVV021932 | IPR003441 | Nac | No apical meristem (NAM) protein |
| Sim4.aln-TCVV023404 | IPR002715 | NAC | Nascent polypeptide-associated complex NAC |
| Twinscan1.VV78X143304.6_1 | IPR003441 | Nac | No apical meristem (NAM) protein |
| FgenesH.VV78X003104.21_1 | IPR004330 | Orphans | FAR1 |
| FgenesH.VV78X015097.7_8 | IPR004330 | Orphans | FAR1 |
| FgenesH.VV78X020408.3_1 | IPR007527 | Orphans | Zinc finger, SWIM-type |
| FgenesH.VV78X036265.13_1 | IPR007527 | Orphans | Zinc finger, SWIM-type |
| FgenesH.VV78X038238.26_1 | IPR007527 | Orphans | Zinc finger, SWIM-type |
| FgenesH.VV78X040981.18_3 | IPR004330 | Orphans | FAR1 |
| FgenesH.VV78X042330.4_1 | IPR007527 | Orphans | Zinc finger, SWIM-type |
| FgenesH.VV78X047108.3_2 | IPR007527 | Orphans | Zinc finger, SWIM-type |
| FgenesH.VV78X048672.5_3 | IPR007527 | Orphans | Zinc finger, SWIM-type |
| FgenesH.VV78X055488.2_1 | IPR004330 | Orphans | FAR1 |
| FgenesH.VV78X056649.9_3 | IPR007527 | Orphans | Zinc finger, SWIM-type |
| FgenesH.VV78X059830.22_2 | IPR007527 | Orphans | Zinc finger, SWIM-type |
| FgenesH.VV78X063226.7_2 | IPR007527 | Orphans | Zinc finger, SWIM-type |
| FgenesH.VV78X066716.7_2 | IPR007527 | Orphans | Zinc finger, SWIM-type |
| FgenesH.VV78X072916.12_1 | IPR006189 | Orphans | CHASE |
| FgenesH.VV78X074017.10_4 | IPR007527 | Orphans | Zinc finger, SWIM-type |
| FgenesH.VV78X077132.15_11 | IPR000315 | Orphans | Zinc finger, B-box |
| FgenesH.VV78X077825.24_6 | IPR004330 | Orphans | FAR1 |
| FgenesH.VV78X080408.8_1 | IPR004330 | Orphans | FAR1 |
| FgenesH.VV78X082839.15_4 | IPR004330 | Orphans | FAR1 |
| FgenesH.VV78X088384.4_3 | IPR004330 | Orphans | FAR1 |
| FgenesH.VV78X090328.10_1 | IPR007527 | Orphans | Zinc finger, SWIM-type |
| FgenesH.VV78X090921.11_2 | IPR004330 | Orphans | FAR1 |
| FgenesH.VV78X097305.13_2 | IPR006189 | Orphans | CHASE |
| FgenesH.VV78X101223.4_1 | IPR007527 | Orphans | Zinc finger, SWIM-type |
| FgenesH.VV78X102512.5_4 | IPR004330 | Orphans | FAR1 |
| FgenesH.VV78X103081.6_1 | IPR007527 | Orphans | Zinc finger, SWIM-type |
| FgenesH.VV78X119308.4_2 | IPR004330 | Orphans | FAR1 |
| FgenesH.VV78X125871.6_3 | IPR007527 | Orphans | Zinc finger, SWIM-type |
| FgenesH.VV78X126014.8_4 | IPR007527 | Orphans | Zinc finger, SWIM-type |
| FgenesH.VV78X139516.11_2 | IPR004330 | Orphans | FAR1 |
| FgenesH.VV78X144144.4_1 | IPR007527 | Orphans | Zinc finger, SWIM-type |
| FgenesH.VV78X155658.13_2 | IPR007527 | Orphans | Zinc finger, SWIM-type |
| FgenesH.VV78X156728.18_2 | IPR004330 | Orphans | FAR1 |
| FgenesH.VV78X166416.6_2 | IPR004330 | Orphans | FAR1 |
| FgenesH.VV78X171202.65_5 | IPR007627 | Orphans | RNA polymerase sigma-70 region 2 |
| FgenesH.VV78X172597.34_1 | IPR004330 | Orphans | FAR1 |
| FgenesH.VV78X181633.12_5 | IPR004330 | Orphans | FAR1 |
| FgenesH.VV78X183252.9_2 | IPR000315 | Orphans | Zinc finger, B-box |
| FgenesH.VV78X186549.15_3 | IPR004330 | Orphans | FAR1 |
| FgenesH.VV78X191552.5_3 | IPR007527 | Orphans | Zinc finger, SWIM-type |
| FgenesH.VV78X194403.7_3 | IPR007527 | Orphans | Zinc finger, SWIM-type |
| FgenesH.VV78X200499.4_2 | IPR007527 | Orphans | Zinc finger, SWIM-type |
| FgenesH.VV78X201494.5_3 | IPR004330 | Orphans | FAR1 |
| FgenesH.VV78X203385.15_2 | IPR000315 | Orphans | Zinc finger, B-box |
| FgenesH.VV78X204584.11_5 | IPR004330 | Orphans | FAR1 |
| FgenesH.VV78X207637.2_1 | IPR004330 | Orphans | FAR1 |
| FgenesH.VV78X207993.16_1 | IPR004330 | Orphans | FAR1 |
| FgenesH.VV78X211090.8_3 | IPR004330 | Orphans | FAR1 |
| FgenesH.VV78X213144.3_3 | IPR007527 | Orphans | Zinc finger, SWIM-type |
| FgenesH.VV78X213722.17_1 | IPR004330 | Orphans | FAR1 |
| FgenesH.VV78X220380.3_2 | IPR004330 | Orphans | FAR1 |
| FgenesH.VV78X220380.3_4 | IPR004330 | Orphans | FAR1 |
| FgenesH.VV78X228491.7_6 | IPR004330 | Orphans | FAR1 |
| FgenesH.VV78X237207.11_1 | IPR006189 | Orphans | CHASE |
| FgenesH.VV78X238445.2_1 | IPR007527 | Orphans | Zinc finger, SWIM-type |
| FgenesH.VV78X240744.7_2 | IPR004330 | Orphans | FAR1 |
| FgenesH.VV78X242738.14_4 | IPR004330 | Orphans | FAR1 |
| FgenesH.VV78X242953.8_3 | IPR004330 | Orphans | FAR1 |
| FgenesH.VV78X250837.10_5 | IPR000315 | Orphans | Zinc finger, B-box |
| FgenesH.VV78X254227.5_4 | IPR004330 | Orphans | FAR1 |
| FgenesH.VV78X259035.12_1 | IPR004330 | Orphans | FAR1 |
| FgenesH.VV78X263438.4_4 | IPR007527 | Orphans | Zinc finger, SWIM-type |
| FgenesH.VV78X271449.13_3 | IPR004330 | Orphans | FAR1 |
| FgenesH.VV79X002400.3_1 | IPR007527 | Orphans | Zinc finger, SWIM-type |
| Glimmer1.VV78X142737.6_3 | IPR004330 | Orphans | FAR1 |
| Glimmer1.VV78X199742.12_2 | IPR004330 | Orphans | FAR1 |
| Glimmer1.VV78X212855.3_3 | IPR004330 | Orphans | FAR1 |
| Sim4.aln-A06005230 | IPR000315 | Orphans | Zinc finger, B-box |
| Sim4.aln-A06013636 | IPR007527 | Orphans | Zinc finger, SWIM-type |
| Sim4.aln-TCVV002300 | IPR000315 | Orphans | Zinc finger, B-box |
| Sim4.aln-TCVV002748 | IPR000315 | Orphans | Zinc finger, B-box |
| Sim4.aln-TCVV005355 | IPR007630 | Orphans | RNA polymerase sigma-70 region 4 |
| Sim4.aln-TCVV006116 | IPR000315 | Orphans | Zinc finger, B-box |
| Sim4.aln-TCVV007079 | IPR007527 | Orphans | Zinc finger, SWIM-type |
| Sim4.aln-TCVV007653 | IPR000315 | Orphans | Zinc finger, B-box |
| Sim4.aln-TCVV008811 | IPR000315 | Orphans | Zinc finger, B-box |
| Sim4.aln-TCVV009289 | IPR000315 | Orphans | Zinc finger, B-box |
| Sim4.aln-TCVV009567 | IPR007527 | Orphans | Zinc finger, SWIM-type |
| Sim4.aln-TCVV010085 | IPR007527 | Orphans | Zinc finger, SWIM-type |
| Sim4.aln-TCVV010651 | IPR000315 | Orphans | Zinc finger, B-box |
| Sim4.aln-TCVV011789 | IPR007527 | Orphans | Zinc finger, SWIM-type |
| Sim4.aln-TCVV012855 | IPR004330 | Orphans | FAR1 |
| Sim4.aln-TCVV013836 | IPR004330 | Orphans | FAR1 |
| Sim4.aln-TCVV015563 | IPR004330 | Orphans | FAR1 |
| Sim4.aln-TCVV017810 | IPR007527 | Orphans | Zinc finger, SWIM-type |
| Sim4.aln-TCVV018687 | IPR007527 | Orphans | Zinc finger, SWIM-type |
| Sim4.aln-TCVV018834 | IPR007527 | Orphans | Zinc finger, SWIM-type |
| Sim4.aln-TCVV018972 | IPR007527 | Orphans | Zinc finger, SWIM-type |
| Sim4.aln-TCVV020065 | IPR004330 | Orphans | FAR1 |
| Sim4.aln-TCVV020703 | IPR003661 | Orphans | Histidine kinase A, N-terminal |
| Sim4.aln-TCVV022584 | IPR000315 | Orphans | Zinc finger, B-box |
| Twinscan1.VV78X030462.7_1 | IPR007527 | Orphans | Zinc finger, SWIM-type |
| Twinscan1.VV78X216372.6_1 | IPR004330 | Orphans | FAR1 |
| FgenesH.VV78X020750.14_1 | IPR001965 | PHD | Zinc finger, PHD-type |
| FgenesH.VV78X030754.11_5 | IPR001965 | PHD | Zinc finger, PHD-type |
| FgenesH.VV78X035910.5_4 | IPR001965 | PHD | Zinc finger, PHD-type |
| FgenesH.VV78X065230.9_1 | IPR001965 | PHD | Zinc finger, PHD-type |
| FgenesH.VV78X073587.19_3 | IPR001965 | PHD | Zinc finger, PHD-type |
| FgenesH.VV78X074657.36_2 | IPR001965 | PHD | Zinc finger, PHD-type |
| FgenesH.VV78X085782.4_1 | IPR001966 | PHD | Zinc finger, PHD-type |
| FgenesH.VV78X087567.5_2 | IPR000313 | PHD | PWWP |
| FgenesH.VV78X106398.8_3 | IPR001967 | PHD | Zinc finger, PHD-type |
| FgenesH.VV78X127056.15_1 | IPR001965 | PHD | Zinc finger, PHD-type |
| FgenesH.VV78X137449.9_3 | IPR001965 | PHD | Zinc finger, PHD-type |
| FgenesH.VV78X148800.19_3 | IPR000313 | PHD | PWWP |
| FgenesH.VV78X159684.7_7 | IPR001965 | PHD | Zinc finger, PHD-type |
| FgenesH.VV78X159684.7_9 | IPR001965 | PHD | Zinc finger, PHD-type |
| FgenesH.VV78X163861.25_1 | IPR001965 | PHD | Zinc finger, PHD-type |
| FgenesH.VV78X187787.36_5 | IPR001965 | PHD | Zinc finger, PHD-type |
| FgenesH.VV78X200864.8_1 | IPR001968 | PHD | Zinc finger, PHD-type |
| FgenesH.VV78X231012.5_1 | IPR001965 | PHD | Zinc finger, PHD-type |
| FgenesH.VV78X241539.6_1 | IPR001965 | PHD | Zinc finger, PHD-type |
| FgenesH.VV78X243065.8_1 | IPR001965 | PHD | Zinc finger, PHD-type |
| FgenesH.VV78X243223.16_2 | IPR001965 | PHD | Zinc finger, PHD-type |
| FgenesH.VV78X247045.9_1 | IPR000313 | PHD | PWWP |
| FgenesH.VV78X248726.3_1 | IPR001965 | PHD | Zinc finger, PHD-type |
| FgenesH.VV78X254218.24_2 | IPR001965 | PHD | Zinc finger, PHD-type |
| FgenesH.VV78X274068.10_1 | IPR001965 | PHD | Zinc finger, PHD-type |
| Glimmer1.VV78X001803.8_2 | IPR001965 | PHD | Zinc finger, PHD-type |
| Glimmer1.VV78X087658.7_6 | IPR001965 | PHD | Zinc finger, PHD-type |
| Glimmer1.VV78X166132.4_1 | IPR001965 | PHD | Zinc finger, PHD-type |
| Glimmer1.VV78X175151.4_3 | IPR001965 | PHD | Zinc finger, PHD-type |
| Glimmer1.VV78X203722.10_2 | IPR001965 | PHD | Zinc finger, PHD-type |
| Glimmer1.VV78X225787.14_3 | IPR001965 | PHD | Zinc finger, PHD-type |
| Glimmer1.VV78X250253.3_2 | IPR001969 | PHD | Zinc finger, PHD-type |
| Sim4.aln-A06005971 | IPR001970 | PHD | Zinc finger, PHD-type |
| Sim4.aln-A06010850 | IPR001965 | PHD | Zinc finger, PHD-type |
| Sim4.aln-A06011476 | IPR001971 | PHD | Zinc finger, PHD-type |
| Sim4.aln-A06013306 | IPR001965 | PHD | Zinc finger, PHD-type |
| Sim4.aln-A06016151 | IPR001972 | PHD | Zinc finger, PHD-type |
| Sim4.aln-TCVV000963 | IPR001973 | PHD | Zinc finger, PHD-type |
| Sim4.aln-TCVV001391 | IPR001974 | PHD | Zinc finger, PHD-type |
| Sim4.aln-TCVV001668 | IPR001965 | PHD | Zinc finger, PHD-type |
| Sim4.aln-TCVV003636 | IPR001975 | PHD | Zinc finger, PHD-type |
| Sim4.aln-TCVV004145 | IPR001965 | PHD | Zinc finger, PHD-type |
| Sim4.aln-TCVV004221 | IPR001976 | PHD | Zinc finger, PHD-type |
| Sim4.aln-TCVV005017 | IPR001977 | PHD | Zinc finger, PHD-type |
| Sim4.aln-TCVV007338 | IPR001978 | PHD | Zinc finger, PHD-type |
| Sim4.aln-TCVV009042 | IPR001979 | PHD | Zinc finger, PHD-type |
| Sim4.aln-TCVV009474 | IPR001980 | PHD | Zinc finger, PHD-type |
| Sim4.aln-TCVV012891 | IPR001965 | PHD | Zinc finger, PHD-type |
| Sim4.aln-TCVV013137 | IPR001981 | PHD | Zinc finger, PHD-type |
| Sim4.aln-TCVV013354 | IPR001982 | PHD | Zinc finger, PHD-type |
| Sim4.aln-TCVV013797 | IPR001965 | PHD | Zinc finger, PHD-type |
| Sim4.aln-TCVV014125 | IPR001983 | PHD | Zinc finger, PHD-type |
| Sim4.aln-TCVV019837 | IPR000313 | PHD | PWWP |
| Sim4.aln-TCVV020019 | IPR001965 | PHD | Zinc finger, PHD-type |
| Sim4.aln-TCVV021302 | IPR001965 | PHD | Zinc finger, PHD-type |
| Sim4.aln-TCVV022064 | IPR001984 | PHD | Zinc finger, PHD-type |
| Sim4.aln-TCVV022175 | IPR001965 | PHD | Zinc finger, PHD-type |
| Sim4.aln-TCVV022281 | IPR001965 | PHD | Zinc finger, PHD-type |
| Sim4.aln-TCVV023229 | IPR001965 | PHD | Zinc finger, PHD-type |
| Sim4.aln-TCVV024131 | IPR000313 | PHD | PWWP |
| Twinscan1.VV78X261753.13_5 | IPR000313 | PHD | PWWP |
| Sim4.aln-TCVV014028 | IPR013742 | PBF-2-like | Plant transcription factor |
| Sim4.aln-TCVV019573 | IPR013742 | PBF-2-like | Plant transcription factor |
| FgenesH.VV78X093905.16_2 | IPR002083 | POZ like | MATH |
| FgenesH.VV78X137299.7_1 | IPR002083 | POZ like | MATH |
| FgenesH.VV78X153799.9_1 | IPR002083 | POZ like | MATH |
| Sim4.aln-A06017129 | IPR002083 | POZ like | MATH |
| Sim4.aln-TCVV012688 | IPR002083 | POZ like | MATH |
| Twinscan1.VV78X221690.8_2 | IPR002083 | POZ like | MATH |
| Sim4.aln-TCVV004935 | IPR007592 | GeBP | Protein of unknown function DUF573 |
| Sim4.aln-TCVV008998 | IPR007592 | GeBP | Protein of unknown function DUF573 |
| Sim4.aln-TCVV014711 | IPR007592 | GeBP | Protein of unknown function DUF573 |
| FgenesH.VV78X156000.48_1 | IPR006734 | PLATZ | Protein of unknown function DUF597 |
| FgenesH.VV78X242953.8_7 | IPR006734 | PLATZ | Protein of unknown function DUF597 |
| Sim4.aln-A06013286 | IPR006734 | PLATZ | Protein of unknown function DUF597 |
| Sim4.aln-A06016667 | IPR006734 | PLATZ | Protein of unknown function DUF597 |
| Sim4.aln-A06018093 | IPR006734 | PLATZ | Protein of unknown function DUF597 |
| Sim4.aln-TCVV002158 | IPR006734 | PLATZ | Protein of unknown function DUF597 |
| Sim4.aln-TCVV016248 | IPR006734 | PLATZ | Protein of unknown function DUF597 |
| Sim4.aln-TCVV016773 | IPR006734 | PLATZ | Protein of unknown function DUF597 |
| FgenesH.VV78X024027.22_2 | IPR007818 | SRS | Protein of unknown function DUF702 |
| FgenesH.VV78X221597.12_8 | IPR007818 | SRS | Protein of unknown function DUF702 |
| Glimmer1.VV78X027578.8_7 | IPR007818 | SRS | Protein of unknown function DUF702 |
| Sim4.aln-A06014649 | IPR007818 | SRS | Protein of unknown function DUF702 |
| Sim4.aln-TCVV006345 | IPR007818 | SRS | Protein of unknown function DUF702 |
| Sim4.aln-TCVV007624 | IPR007818 | SRS | Protein of unknown function DUF702 |
| FgenesH.VV78X073587.19_3 | IPR011016 | RING | RINGv |
| FgenesH.VV78X085782.4_1 | IPR011016 | RING | RINGv |
| FgenesH.VV78X106398.8_3 | IPR011016 | RING | RINGv |
| FgenesH.VV78X200864.8_1 | IPR011016 | RING | RINGv |
| Glimmer1.VV78X250253.3_2 | IPR011016 | RING | RINGv |
| Sim4.aln-A06005971 | IPR011016 | RING | RINGv |
| Sim4.aln-A06011476 | IPR011016 | RING | RINGv |
| Sim4.aln-A06016151 | IPR011016 | RING | RINGv |
| Sim4.aln-TCVV000963 | IPR011016 | RING | RINGv |
| Sim4.aln-TCVV001391 | IPR011016 | RING | RINGv |
| Sim4.aln-TCVV003636 | IPR011016 | RING | RINGv |
| Sim4.aln-TCVV004221 | IPR011016 | RING | RINGv |
| Sim4.aln-TCVV005017 | IPR011016 | RING | RINGv |
| Sim4.aln-TCVV007338 | IPR011016 | RING | RINGv |
| Sim4.aln-TCVV009042 | IPR011016 | RING | RINGv |
| Sim4.aln-TCVV009474 | IPR011016 | RING | RINGv |
| Sim4.aln-TCVV013137 | IPR011016 | RING | RINGv |
| Sim4.aln-TCVV013354 | IPR011016 | RING | RINGv |
| Sim4.aln-TCVV014125 | IPR011016 | RING | RINGv |
| Sim4.aln-TCVV022064 | IPR011016 | RING | RINGv |
| FgenesH.VV78X076853.5_2 | IPR003035 | RWP-RK | Plant regulator RWP-RK |
| FgenesH.VV78X083354.6_1 | IPR003035 | RWP-RK | Plant regulator RWP-RK |
| FgenesH.VV78X134548.14_1 | IPR003035 | RWP-RK | Plant regulator RWP-RK |
| FgenesH.VV78X156061.11_2 | IPR003035 | RWP-RK | Plant regulator RWP-RK |
| FgenesH.VV78X156692.10_1 | IPR003035 | RWP-RK | Plant regulator RWP-RK |
| FgenesH.VV78X223647.11_2 | IPR003035 | RWP-RK | Plant regulator RWP-RK |
| FgenesH.VV78X233216.7_3 | IPR003035 | RWP-RK | Plant regulator RWP-RK |
| Glimmer1.VV78X020893.8_4 | IPR003035 | RWP-RK | Plant regulator RWP-RK |
| Glimmer1.VV78X196743.5_3 | IPR003035 | RWP-RK | Plant regulator RWP-RK |
| Sim4.aln-A06013452 | IPR003035 | RWP-RK | Plant regulator RWP-RK |
| Sim4.aln-TCVV004521 | IPR003035 | RWP-RK | Plant regulator RWP-RK |
| Sim4.aln-TCVV005607 | IPR003035 | RWP-RK | Plant regulator RWP-RK |
| Sim4.aln-TCVV016230 | IPR003035 | RWP-RK | Plant regulator RWP-RK |
| Sim4.aln-TCVV017721 | IPR003035 | RWP-RK | Plant regulator RWP-RK |
| FgenesH.VV78X058725.19_2 | IPR006779 | S1Fa-like | DNA binding protein S1FA |
| Sim4.aln-A06010732 | IPR006779 | S1Fa-like | DNA binding protein S1FA |
| Sim4.aln-TCVV023798 | IPR006779 | S1Fa-like | DNA binding protein S1FA |
| FgenesH.VV78X008546.2_4 | IPR004333 | SBP | SBP |
| FgenesH.VV78X025227.7_2 | IPR004333 | SBP | SBP |
| FgenesH.VV78X050214.9_3 | IPR004333 | SBP | SBP |
| FgenesH.VV78X074716.3_4 | IPR004333 | SBP | SBP |
| FgenesH.VV78X102566.12_1 | IPR004333 | SBP | SBP |
| FgenesH.VV78X243089.3_1 | IPR004333 | SBP | SBP |
| Glimmer1.VV78X009490.48_8 | IPR004333 | SBP | SBP |
| Sim4.aln-A06010800 | IPR004333 | SBP | SBP |
| Sim4.aln-A06016577 | IPR004333 | SBP | SBP |
| Sim4.aln-TCVV001464 | IPR004333 | SBP | SBP |
| Sim4.aln-TCVV009255 | IPR004333 | SBP | SBP |
| Sim4.aln-TCVV009586 | IPR004333 | SBP | SBP |
| Sim4.aln-TCVV015097 | IPR004333 | SBP | SBP |
| Sim4.aln-TCVV016962 | IPR004333 | SBP | SBP |
| Sim4.aln-TCVV023043 | IPR004333 | SBP | SBP |
| Twinscan1.VV78X004301.10_2 | IPR004333 | SBP | SBP |
| Twinscan1.VV78X078738.30_3 | IPR004333 | SBP | SBP |
| Twinscan1.VV78X102566.12_7 | IPR004333 | SBP | SBP |
| FgenesH.VV78X018049.10_1 | IPR001214 | SET | SET |
| FgenesH.VV78X023773.8_1 | IPR001214 | SET | SET |
| FgenesH.VV78X040611.4_3 | IPR001214 | SET | SET |
| FgenesH.VV78X042193.6_4 | IPR001214 | SET | SET |
| FgenesH.VV78X051051.14_5 | IPR001214 | SET | SET |
| FgenesH.VV78X093984.17_7 | IPR001214 | SET | SET |
| FgenesH.VV78X111514.7_5 | IPR001214 | SET | SET |
| FgenesH.VV78X115611.4_1 | IPR001214 | SET | SET |
| FgenesH.VV78X115863.6_2 | IPR001214 | SET | SET |
| FgenesH.VV78X117496.2_3 | IPR001214 | SET | SET |
| FgenesH.VV78X148268.7_2 | IPR001214 | SET | SET |
| FgenesH.VV78X150157.8_1 | IPR001214 | SET | SET |
| FgenesH.VV78X161420.17_5 | IPR001214 | SET | SET |
| FgenesH.VV78X192714.18_11 | IPR001214 | SET | SET |
| FgenesH.VV78X216374.4_1 | IPR001214 | SET | SET |
| FgenesH.VV78X225378.8_1 | IPR001214 | SET | SET |
| FgenesH.VV78X259973.14_1 | IPR001214 | SET | SET |
| FgenesH.VV78X262710.3_4 | IPR001214 | SET | SET |
| FgenesH.VV78X264291.8_2 | IPR001214 | SET | SET |
| FgenesH.VV78X269838.4_2 | IPR001214 | SET | SET |
| Glimmer1.VV78X143690.12_3 | IPR001214 | SET | SET |
| Glimmer1.VV78X213710.9_2 | IPR001214 | SET | SET |
| Glimmer1.VV78X250316.5_1 | IPR001214 | SET | SET |
| Sim4.aln-A06011263 | IPR001214 | SET | SET |
| Sim4.aln-A06012617 | IPR001214 | SET | SET |
| Sim4.aln-A06012674 | IPR001214 | SET | SET |
| Sim4.aln-A06013431 | IPR001214 | SET | SET |
| Sim4.aln-TCVV009908 | IPR001214 | SET | SET |
| Sim4.aln-TCVV016146 | IPR001214 | SET | SET |
| Sim4.aln-TCVV022823 | IPR001214 | SET | SET |
| FgenesH.VV78X135233.9_2 | IPR007624 | Sigma70-like | RNA polymerase sigma-70 region 3 |
| Glimmer1.VV78X004959.5_12 | IPR007624 | Sigma70-like | RNA polymerase sigma-70 region 3 |
| Sim4.aln-TCVV000736 | IPR007624 | Sigma70-like | RNA polymerase sigma-70 region 3 |
| Sim4.aln-TCVV008020 | IPR007624 | Sigma70-like | RNA polymerase sigma-70 region 3 |
| Sim4.aln-TCVV014739 | IPR007624 | Sigma70-like | RNA polymerase sigma-70 region 3 |
| FgenesH.VV78X016531.16_1 | IPR000330 | SNF2 | SNF2-related |
| FgenesH.VV78X033737.3_3 | IPR000330 | SNF2 | SNF2-related |
| FgenesH.VV78X085811.7_5 | IPR000330 | SNF2 | SNF2-related |
| FgenesH.VV78X090328.10_10 | IPR000330 | SNF2 | SNF2-related |
| FgenesH.VV78X093132.14_1 | IPR000330 | SNF2 | SNF2-related |
| FgenesH.VV78X099943.17_1 | IPR000330 | SNF2 | SNF2-related |
| FgenesH.VV78X104218.13_6 | IPR000330 | SNF2 | SNF2-related |
| FgenesH.VV78X106640.32_6 | IPR000330 | SNF2 | SNF2-related |
| FgenesH.VV78X125257.5_2 | IPR000330 | SNF2 | SNF2-related |
| FgenesH.VV78X148303.13_1 | IPR000330 | SNF2 | SNF2-related |
| FgenesH.VV78X150337.7_3 | IPR000330 | SNF2 | SNF2-related |
| FgenesH.VV78X151904.11_1 | IPR000330 | SNF2 | SNF2-related |
| FgenesH.VV78X159529.6_1 | IPR000330 | SNF2 | SNF2-related |
| FgenesH.VV78X170167.3_1 | IPR000330 | SNF2 | SNF2-related |
| FgenesH.VV78X197346.10_1 | IPR000330 | SNF2 | SNF2-related |
| FgenesH.VV78X201737.12_1 | IPR009462 | SNF2 | Protein of unknown function DUF1086 |
| FgenesH.VV78X201737.12_4 | IPR000330 | SNF2 | SNF2-related |
| FgenesH.VV78X207637.2_5 | IPR000330 | SNF2 | SNF2-related |
| FgenesH.VV78X218293.12_2 | IPR000330 | SNF2 | SNF2-related |
| FgenesH.VV78X221725.4_1 | IPR000330 | SNF2 | SNF2-related |
| FgenesH.VV78X223143.4_1 | IPR000330 | SNF2 | SNF2-related |
| FgenesH.VV78X233805.4_10 | IPR000330 | SNF2 | SNF2-related |
| FgenesH.VV78X233805.4_8 | IPR000330 | SNF2 | SNF2-related |
| FgenesH.VV78X239622.11_5 | IPR000330 | SNF2 | SNF2-related |
| FgenesH.VV78X276335.6_1 | IPR000330 | SNF2 | SNF2-related |
| Sim4.aln-TCVV013433 | IPR000330 | SNF2 | SNF2-related |
| Sim4.aln-TCVV015432 | IPR000330 | SNF2 | SNF2-related |
| FgenesH.VV78X014746.3_2 | IPR013069 | TAZ | BTB/POZ |
| FgenesH.VV78X014784.5_2 | IPR013069 | TAZ | BTB/POZ |
| FgenesH.VV78X020893.8_1 | IPR013069 | TAZ | BTB/POZ |
| FgenesH.VV78X046426.8_3 | IPR009255 | TAZ | Transcriptional coactivation |
| FgenesH.VV78X096741.3_2 | IPR009255 | TAZ | Transcriptional coactivation |
| FgenesH.VV78X161014.26_1 | IPR013069 | TAZ | BTB/POZ |
| FgenesH.VV78X193990.5_4 | IPR013069 | TAZ | BTB/POZ |
| Sim4.aln-TCVV004308 | IPR013069 | TAZ | BTB/POZ |
| Sim4.aln-TCVV016767 | IPR000197 | TAZ | Zinc finger, TAZ-type |
| FgenesH.VV78X010330.4_1 | IPR005333 | TCP | TCP transcription factor |
| FgenesH.VV78X105762.7_1 | IPR005333 | TCP | TCP transcription factor |
| FgenesH.VV78X120795.6_1 | IPR005333 | TCP | TCP transcription factor |
| Glimmer1.VV78X021096.6_2 | IPR005333 | TCP | TCP transcription factor |
| Glimmer1.VV78X123770.2_5 | IPR005333 | TCP | TCP transcription factor |
| Glimmer1.VV78X128180.7_6 | IPR005333 | TCP | TCP transcription factor |
| Sim4.aln-A06000762 | IPR005333 | TCP | TCP transcription factor |
| Sim4.aln-A06002588 | IPR005333 | TCP | TCP transcription factor |
| Sim4.aln-TCVV001698 | IPR005333 | TCP | TCP transcription factor |
| Sim4.aln-TCVV002611 | IPR005333 | TCP | TCP transcription factor |
| Sim4.aln-TCVV006285 | IPR005333 | TCP | TCP transcription factor |
| Sim4.aln-TCVV009520 | IPR005333 | TCP | TCP transcription factor |
| Sim4.aln-TCVV009617 | IPR005333 | TCP | TCP transcription factor |
| Sim4.aln-TCVV012374 | IPR005333 | TCP | TCP transcription factor |
| Twinscan1.VV78X023076.3_1 | IPR005333 | TCP | TCP transcription factor |
| Twinscan1.VV78X064736.11_2 | IPR005333 | TCP | TCP transcription factor |
| FgenesH.VV78X012132.9_1 | IPR001810 | TUB | Cyclin-like F-box |
| FgenesH.VV78X187893.3_2 | IPR001810 | TUB | Cyclin-like F-box |
| FgenesH.VV78X233655.5_3 | IPR001810 | TUB | Cyclin-like F-box |
| Sim4.aln-A06005531 | IPR001810 | TUB | Cyclin-like F-box |
| Sim4.aln-TCVV002233 | IPR001810 | TUB | Cyclin-like F-box |
| Sim4.aln-TCVV003630 | IPR001810 | TUB | Cyclin-like F-box |
| Sim4.aln-TCVV006171 | IPR001810 | TUB | Cyclin-like F-box |
| Sim4.aln-TCVV009639 | IPR001810 | TUB | Cyclin-like F-box |
| Sim4.aln-TCVV013430 | IPR001810 | TUB | Cyclin-like F-box |
| Sim4.aln-TCVV023322 | IPR001810 | TUB | Cyclin-like F-box |
| Twinscan1.VV78X072959.3_1 | IPR001810 | TUB | Cyclin-like F-box |
| FgenesH.VV78X017606.11_1 | IPR003657 | WRKY | DNA-binding WRKY |
| FgenesH.VV78X018528.29_1 | IPR003657 | WRKY | DNA-binding WRKY |
| FgenesH.VV78X018825.12_3 | IPR003657 | WRKY | DNA-binding WRKY |
| FgenesH.VV78X040591.14_3 | IPR003657 | WRKY | DNA-binding WRKY |
| FgenesH.VV78X047849.6_3 | IPR003657 | WRKY | DNA-binding WRKY |
| FgenesH.VV78X073431.5_1 | IPR003657 | WRKY | DNA-binding WRKY |
| FgenesH.VV78X074716.3_3 | IPR003657 | WRKY | DNA-binding WRKY |
| FgenesH.VV78X087117.8_3 | IPR003657 | WRKY | DNA-binding WRKY |
| FgenesH.VV78X089168.35_3 | IPR003657 | WRKY | DNA-binding WRKY |
| FgenesH.VV78X111726.11_4 | IPR003657 | WRKY | DNA-binding WRKY |
| FgenesH.VV78X132881.3_2 | IPR003657 | WRKY | DNA-binding WRKY |
| FgenesH.VV78X156061.11_3 | IPR003657 | WRKY | DNA-binding WRKY |
| FgenesH.VV78X157796.9_2 | IPR003657 | WRKY | DNA-binding WRKY |
| FgenesH.VV78X161195.8_1 | IPR003657 | WRKY | DNA-binding WRKY |
| FgenesH.VV78X168903.5_1 | IPR003657 | WRKY | DNA-binding WRKY |
| FgenesH.VV78X168903.5_2 | IPR003657 | WRKY | DNA-binding WRKY |
| FgenesH.VV78X178339.3_9 | IPR003657 | WRKY | DNA-binding WRKY |
| FgenesH.VV78X183642.29_8 | IPR003657 | WRKY | DNA-binding WRKY |
| FgenesH.VV78X211539.4_4 | IPR003657 | WRKY | DNA-binding WRKY |
| FgenesH.VV78X228516.3_1 | IPR003657 | WRKY | DNA-binding WRKY |
| FgenesH.VV78X255622.5_3 | IPR003657 | WRKY | DNA-binding WRKY |
| FgenesH.VV78X258458.2_1 | IPR003657 | WRKY | DNA-binding WRKY |
| FgenesH.VV78X278469.4_1 | IPR003657 | WRKY | DNA-binding WRKY |
| Glimmer1.VV78X017715.5_1 | IPR003657 | WRKY | DNA-binding WRKY |
| Glimmer1.VV78X030349.9_2 | IPR003657 | WRKY | DNA-binding WRKY |
| Glimmer1.VV78X038611.6_6 | IPR003657 | WRKY | DNA-binding WRKY |
| Glimmer1.VV78X121610.5_3 | IPR003657 | WRKY | DNA-binding WRKY |
| Glimmer1.VV78X178339.3_13 | IPR003657 | WRKY | DNA-binding WRKY |
| Glimmer1.VV78X187875.7_2 | IPR003657 | WRKY | DNA-binding WRKY |
| Sim4.aln-A06008098 | IPR003657 | WRKY | DNA-binding WRKY |
| Sim4.aln-A06010251 | IPR003657 | WRKY | DNA-binding WRKY |
| Sim4.aln-A06012623 | IPR003657 | WRKY | DNA-binding WRKY |
| Sim4.aln-A06015003 | IPR003657 | WRKY | DNA-binding WRKY |
| Sim4.aln-A06015110 | IPR003657 | WRKY | DNA-binding WRKY |
| Sim4.aln-TCVV001455 | IPR003657 | WRKY | DNA-binding WRKY |
| Sim4.aln-TCVV001772 | IPR003657 | WRKY | DNA-binding WRKY |
| Sim4.aln-TCVV001794 | IPR003657 | WRKY | DNA-binding WRKY |
| Sim4.aln-TCVV001834 | IPR003657 | WRKY | DNA-binding WRKY |
| Sim4.aln-TCVV002024 | IPR003657 | WRKY | DNA-binding WRKY |
| Sim4.aln-TCVV002191 | IPR003657 | WRKY | DNA-binding WRKY |
| Sim4.aln-TCVV003334 | IPR003657 | WRKY | DNA-binding WRKY |
| Sim4.aln-TCVV003609 | IPR003657 | WRKY | DNA-binding WRKY |
| Sim4.aln-TCVV004904 | IPR003657 | WRKY | DNA-binding WRKY |
| Sim4.aln-TCVV005025 | IPR003657 | WRKY | DNA-binding WRKY |
| Sim4.aln-TCVV005035 | IPR003657 | WRKY | DNA-binding WRKY |
| Sim4.aln-TCVV008936 | IPR003657 | WRKY | DNA-binding WRKY |
| Sim4.aln-TCVV009455 | IPR003657 | WRKY | DNA-binding WRKY |
| Sim4.aln-TCVV011877 | IPR003657 | WRKY | DNA-binding WRKY |
| Sim4.aln-TCVV013564 | IPR003657 | WRKY | DNA-binding WRKY |
| Sim4.aln-TCVV013690 | IPR003657 | WRKY | DNA-binding WRKY |
| Sim4.aln-TCVV015779 | IPR003657 | WRKY | DNA-binding WRKY |
| Sim4.aln-TCVV018623 | IPR003657 | WRKY | DNA-binding WRKY |
| Sim4.aln-TCVV018753 | IPR003657 | WRKY | DNA-binding WRKY |
| Sim4.aln-TCVV021342 | IPR003657 | WRKY | DNA-binding WRKY |
| Sim4.aln-TCVV022972 | IPR003657 | WRKY | DNA-binding WRKY |
| Twinscan1.VV78X041870.2_2 | IPR003657 | WRKY | DNA-binding WRKY |
| FgenesH.VV78X039809.13_2 | IPR006456 | zf-HD | ZF-HD homeobox protein Cys/His-rich dimerisation region |
| FgenesH.VV78X131483.10_2 | IPR006456 | zf-HD | ZF-HD homeobox protein Cys/His-rich dimerisation region |
| FgenesH.VV78X144196.4_1 | IPR006456 | zf-HD | ZF-HD homeobox protein Cys/His-rich dimerisation region |
| FgenesH.VV78X167962.2_9 | IPR006456 | zf-HD | ZF-HD homeobox protein Cys/His-rich dimerisation region |
| FgenesH.VV78X205347.6_2 | IPR006456 | zf-HD | ZF-HD homeobox protein Cys/His-rich dimerisation region |
| Sim4.aln-A06012367 | IPR006456 | zf-HD | ZF-HD homeobox protein Cys/His-rich dimerisation region |
| Sim4.aln-A06014720 | IPR006456 | zf-HD | ZF-HD homeobox protein Cys/His-rich dimerisation region |
| Sim4.aln-A06017620 | IPR006456 | zf-HD | ZF-HD homeobox protein Cys/His-rich dimerisation region |
| Sim4.aln-TCVV006475 | IPR006456 | zf-HD | ZF-HD homeobox protein Cys/His-rich dimerisation region |
| Sim4.aln-TCVV014799 | IPR006456 | zf-HD | ZF-HD homeobox protein Cys/His-rich dimerisation region |
| Sim4.aln-TCVV022401 | IPR006456 | zf-HD | ZF-HD homeobox protein Cys/His-rich dimerisation region |
| Twinscan1.VV78X096604.7_3 | IPR006456 | zf-HD | ZF-HD homeobox protein Cys/His-rich dimerisation region |
| FgenesH.VV78X163126.5_2 | IPR010399 | ZIM | ZIM |
| Sim4.aln-TCVV000834 | IPR010399 | ZIM | ZIM |
| Sim4.aln-TCVV001678 | IPR010399 | ZIM | ZIM |
| Sim4.aln-TCVV008929 | IPR010399 | ZIM | ZIM |
| Sim4.aln-TCVV009082 | IPR010399 | ZIM | ZIM |
| Sim4.aln-TCVV014448 | IPR010399 | ZIM | ZIM |
| Sim4.aln-TCVV016621 | IPR010399 | ZIM | ZIM |
| Sim4.aln-TCVV016637 | IPR010399 | ZIM | ZIM |
| Sim4.aln-TCVV016976 | IPR010399 | ZIM | ZIM |
| Sim4.aln-TCVV017215 | IPR010399 | ZIM | ZIM |
| Sim4.aln-TCVV024045 | IPR010399 | ZIM | ZIM |
| Twinscan1.VV78X140880.10_1 | IPR010399 | ZIM | ZIM |
| FgenesH.VV78X195981.11_3 | IPR010666 | GRF | Zinc finger, GRF-type |
| Sim4.aln-TCVV008118 | IPR010666 | GRF | Zinc finger, GRF-type |
| Sim4.aln-TCVV021431 | IPR010666 | GRF | Zinc finger, GRF-type |
| Sim4.aln-TCVV023554 | IPR010666 | GRF | Zinc finger, GRF-type |
| Twinscan1.VV78X270825.7_2 | IPR010666 | GRF | Zinc finger, GRF-type |
| FgenesH.VV78X275378.15_5 | IPR001965 | Alfin-like | Zinc finger, PHD-type |
| Sim4.aln-A06000698 | IPR001965 | Alfin-like | Zinc finger, PHD-type |
| Sim4.aln-A06011551 | IPR001965 | Alfin-like | Zinc finger, PHD-type |
| Sim4.aln-TCVV004661 | IPR001965 | Alfin-like | Zinc finger, PHD-type |

The list contains the 2004 transcription factor genes found in the genome sorted by family. Passport data are gene ID, IPR number (according to InterPro database, [6]) followed by their putative functional annotation.
