# Supplementary material for: A High Quality Draft Consensus Sequence of the Genome of a Heterozygous Grapevine Variety
Source: PLoS One. 2007 Dec 19;2(12):e1326. doi: 10.1371/journal.pone.0001326 (PMC2147077; doi:10.1371/journal.pone.0001326)
Supplement: Table S7. — Repetitive elements in the assembled V. vinifera genome. (0.04 MB DOC) [file pone.0001326.s014.doc]

**Table S7. Repetitive elements in the assembled *V. vinifera* genome.**

| **Repeat class** | | **Copies**  **(no.)** | **Total length (Mb)** | **Alternative**  **estimate (Mb)^2^** |
| --- | --- | --- | --- | --- |
| **with ORF** | **without ORF** |
| DNA/Mutator |  | 1,949 | 1.96 | 2.4 |
| DNA/hAT |  | 6,150 | 3.65 | 4.4 |
| DNA/hAT:Dart |  | 549 | 0.44 | 0.5 |
| DNA/CACTA |  | 914 | 1.10 | 1.3 |
| Retro/LTR_Copia |  | 16,649 | 24.07 | 29.2 |
| Retro/LTR_Gypsy/gypsy |  | 6,117 | 7.75 | 9.4 |
| Retro/LTR_Gypsy/athila |  | 43,345 | 62.94 | 76.3 |
| Retro/non-LTR_Karma |  | 2,745 | 3.18 | 3.9 |
| Unclassified | | 6,266 | 3.42 | 4.1 |
|  | Long Tandem | 5,799 | 3.49 | 4.3 |
|  | Short Tandem ^1^ | 88,909 | 2.15 | 2.7 |
| **Total** | | **179,392** | **114.14** | **138.5** |

Elements were identified by BLAST against the Uniprot database (Uniprot Consortium 2007), followed by an all-*vs*-all comparison by the BLAST program.

^1^ = detailed in Table S8.

^2^ = based on number and total length of reads matching the identified repeat sequences. (see Materials and Methods).
