# Supplementary material for: A High Quality Draft Consensus Sequence of the Genome of a Heterozygous Grapevine Variety
Source: PLoS One. 2007 Dec 19;2(12):e1326. doi: 10.1371/journal.pone.0001326 (PMC2147077; doi:10.1371/journal.pone.0001326)
Supplement: Table S8. — Microsatellites identified in the assembled V. vinifera genome. (0.03 MB DOC) [file pone.0001326.s015.doc]

**Table S8. Microsatellites identified in the assembled *V. vinifera* genome.**

| **Unit size ^1^** | **Minimum number**  **of repeat units** | **Copies**  **(no.)** | **Total length (bp)** |
| --- | --- | --- | --- |
| 1 (A or T) | 18 | 12,759 | 262,430 |
| 1 (G or C) | 11 | 2,297 | 31,990 |
| 2 | 7 | 31,463 | 845,311 |
| 3 | 6 | 13,851 | 360,978 |
| 4 | 5 | 6,081 | 140,142 |
| 5 | 4 | 4,253 | 99,229 |
| 6 | 3 | 12,071 | 253,762 |
| 7 | 3 | 4,902 | 120,071 |
| 8 | 3 | 1,232 | 33,997 |
| **Total** |  | **88,909** | **2,147,911** |

**^1^** For unit sizes greater than 1, all possible combinations of different nucleotides were considered.
