# Supplementary material for: A High Quality Draft Consensus Sequence of the Genome of a Heterozygous Grapevine Variety
Source: PLoS One. 2007 Dec 19;2(12):e1326. doi: 10.1371/journal.pone.0001326 (PMC2147077; doi:10.1371/journal.pone.0001326)
Supplement: Table S9. — Current state of IASMA database dedicated to V. vinifera mature miRNAs and miRNAs*, including the predicted fold-back structures of the pre-miRNAs. (0.09 MB DOC) [file pone.0001326.s016.doc]

**Table S9.** Current state of IASMA database dedicated to *V. vinifera* mature miRNAs and miRNAs*, including the predicted fold-back structures of the pre-miRNAs.

| **miRNA family** | **miRNA (5’-3’)** | **Conserved miRNA* (5’-3’)** |
| --- | --- | --- |
|  |  |  |
| miR156/157 | >Vv-miR156a  UGACAGAAGAGAGAGAGCAC  >Vv-miR156b  UGACAGAGGAGAGUGAGCAC  >Vv-miR156c  UGACAGAAGAGAGUGAGCAC  >Vv-miR156d  UGACAGAAGAGAGAGAGCAC  >Vv-miR156e  UGACAGAAGAGAGUGAGCAC  >Vv-miR156f  UGACAGAAGAGAGAGAGCAC  >Vv-miR157a  UUGACAGAAGAUAGAGAGCAC  >Vv-miR157b  UUGACAGGAGAUAGAGAGCA  >Vv-miR157c  UGACAGAAGAAUAGAGAGCAC  >Vv-miR157d  UGACAGAAGAGAGAGAGCAC  >Vv-miR157e  UUGACAGAAGAGAGAGAGCAC | >Vv-miR156a*  GCUCUCUAUCUUCUGUCAACA  >Vv-miR156b*  GCUCUCUAUCUUCUGUCAACA  >Vv-miR156c*  GUCCUCUCUCUUCUCCUGUCA  >Vv-miR157a*  UGCUCACCUCUCUUUCUGUCA  >Vv-miR157b*  UGCUCACCUCUCUUUCUGUCAG |
| miR159/319 | >Vv-miR159a  UUUGGUUUGAAGGGAGCUCU  >Vv-miR159b  UUUGGAUUGAAGGGAGCUCUA  >Vv-miR159c  UUUGGAUUGAAGGAAGCUCUA  >Vv-miR159d  UUGGAGUGAAGGGAGCUCUA  >Vv-miR159e  UUUGGUUUGAAGGGAGCUCU  >Vv-miR319a  UUGGACUGAAGGGAGCUCC  >Vv-miR319b  UUGGACUGAAGGGAGCUCCCU  >Vv-miR319c  UUGGACUGAAGGGAGCUCCC  >Vv-miR319d  UUGGACUGAAGGGAGCUCCCU  >Vv-miR319e  UUGGACUGAAGGGAGCUCCCU |  |
| miR160 | >Vv-miR160a  UGCCUGGCUCCCUGUAUGCCA  >Vv-miR160b  UGCCUGGCUCCCUGAAUGCCA  >Vv-miR160c  UGCCUGGCUCCCUGUAUGCCA  >Vv-miR160d  UGGCAUGCAGGGAGCCAGGCA  >Vv-miR160e  UGCCUGGCUCCCUGUAUGCCA  >Vv-miR160f  UGCCUGGCUCCCUGCAUGCC |  |
| miR162 | >Vv-miR162  UCGAUAAACCUCUGCAUCCAG |  |
| miR164 | >Vv-miR164a  UGGAGAAGCAGGGCACGUGCA  >Vv-miR164b  UGGAGAACCAGGGCACGUGC  >Vv-miR164c  UGGAGAAGCAGGGCACGUGCA  >Vv-miR164d  UGGAGAAGCAGGGCACGUGCA | >Vv-miR164a*  CACGUGCCCUGCUUCUCCAAC  >Vv-miR164b*  CAUGUGCCCCUCUUCCCCAUC  >Vv-miR164c*  CACGUGCUCCCCUUCUCCAAC |
| miR165/166 | >Vv-miR166a  UCGGACCAGGCUUCAUUCCCC  >Vv-miR166b  UCGGACCAGGCUUCAUUCCCC  >Vv-miR166c  UCGGACCAGGCUUCAUUCCCC  >Vv-miR166d  UCGGACCAGGCUUCAUUCCCC  >Vv-miR166e  UCGGACCAGGCUUCAUUCCCC  >Vv-miR166f  UCGGACCAGGCUUCAUUCCCC  >Vv-miR166g  UCGGACCAGGCUUCAUUCC  >Vv-miR166h  UCGGACCAGGCUUCAUUCC  >Vv-miR166i  UCGGACCAGGCUUCAUUCCCC |  |
| miR167 | >Vv-miR167a  UGAAGCUGCCAGCAUGAUCUA  >Vv-miR167b  UGAAGCUGCCAGCAUGAUCU  >Vv-miR167c  UGAAGCUGCCAGCAUGAUCU  >Vv-miR167d  UAGAUCAUGCUGGCAGCUUCA  >Vv-miR167e  UAGAUCAUGCUGGCAGCUUCA | >Vv-miR167a*  AGGUCAUGCCCUGACAGCCUCACU  >Vv-miR167b*  UGUCAAGUUUUGACAG  >Vv-miR167c*  UAGACCAGGCCGCCCGUUUCCC  >Vv-miR167d*  UAGAUCAUGUGGCAGUUUCACC |
| miR168 | >Vv-miR168  UCGCUUGGUGCAGGUCGGGAA | >Vv-miR168*  CCCGCCUUGCAUCAACUGAAU |
| miR169 | >Vv-miR169a  AGCCAAGGAUGACUUGCCG  >Vv-miR169b  CAGCCAAGGAUGACUUGCCG  >Vv-miR169c  CAGCCAAGGAUGACUUGCCGA  >Vv-miR169d  AGCCAAGGAUGACUUGCCG  >Vv-miR169e  CAGCCAAGGAUGACUUGCCG  >Vv-miR169f  AGCCAAGGAUGACUUGCCG  >Vv-miR169g  AGCCAAGGAUGACUUGCCG  >Vv-miR169h  CAGCCAAGGAUGACUUGCCG  >Vv-miR169i  AGCCAAGGAUGACUUGCCG  >Vv-miR169j  AGCCAAGGAUGACUUGCCG  >Vv-miR169k  CAGCCAAGGAUGACUUGCCG  >Vv-miR169l  AGCCAAGGAUGACUUGCCG  >Vv-miR169m  AGCCAAGGAUGACUUGCC  >Vv-miR169n  CAGCCAAGGAUGACUUGCCGA  >Vv-miR169o  AGCCAAGGAUGACUUGCC  >Vv-miR169p  AGCCAAGGAUGACUUGCC  >Vv-miR169q  CAGCCAAGGAUGACUUGCCG | >Vv-miR169a*  GCAAGUCAUCCUUGGCUCAAU  >Vv-miR169b*  GUAGACUGUCAUUCUACCAA  >Vv-miR169c*  GGCAAGUCAUCCUUGGCU  >Vv-miR169d*  GGCAAGUCAUCCUUGGCU  >Vv-miR169e*  GGCAGGUUGUCCUUGGCUAC  >Vv-miR169f*  GGCAAGUCAUCCUUGGCUGC  >Vv-miR169g*  GUAGACUGUCAUUCUACCA  >Vv-miR169h*  GUUUCUUUGACUAUGC  >Vv-miR169i*  GCAAGUCAUCCUUGGCU  >Vv-miR169j*  GCAAGUCAUCCUUGGCUC  >Vv-miR169k*  GGCAGUCAUUCCUUCG  >Vv-miR169l*  CAGUUUCUAUUGCAAUU  >Vv-miR169m*  GGCAGUCACCUUGGCUAAU  >Vv-miR169n*  GGCAGGUUGUCCUUGGCUAC |
| miR170/171 | >Vv-miR170a  UGAUUGAGCCGUGCCAAUAUC  >Vv-miR170b  UGAUUGAGCCGCGUCAAUAUC  >Vv-miR170c  UGAUUGAGCCGUGCCAAUAUC  >Vv-miR170d  UGAUUGAGCCGUGCCAAUAUC  >Vv-miR170e  UGAUUGAGCCGUGCCAAUAUC  >Vv-miR170f  UGAUUGAGCCGUGCCAAUAUC  >Vv-miR170g  UGAUUGAGCCGUGCCAAUAUC  >Vv-miR171a  UGAUUGAGCCGCGCCAAUAUC  >Vv-miR171b  UGAUUGAGCCGCGCCAAUAUC  >Vv-miR171c  UUGAGCCGCGCCAAUAUC  >Vv-miR171d  UGAUUGAGCCGCGCCAAUAUC  >Vv-miR171e  UGAUUGAGCCGCGCCAAUAUC  >Vv-miR171f  GAUUGAGCCGCGCCAAUAUC | >Vv-miR170a*  UUGAACCUCACCAACAUCGC  >Vv-miR170b*  UUGAACCGUAUCAAUAUCUCG  >Vv-miR170c*  UUGAGCCGCGCCAAUAUCAC |
| miR172 | >Vv-miR172a  GAAUCUUGAUGAUGCUGCA  >Vv-miR172b  AGAAUCUUGAUGAUGCUGCAU  >Vv-miR172c  GAAUCUUGAUGAUGCUGCAU  >Vv-miR172d  AGAAUCCUGAUGAUGCUGCA  >Vv-miR172e  AGAAUCUUGAUGAUGCUGCAU  >Vv-miR172f  AGAAUCCUGAUGAUGCUGCA  >Vv-miR172g  AGAAUCUUGAUGAUGCUGCAU  >Vv-miR172h  GAAUCUUGAUGAUGCUGCAU  >Vv-miR172i  GAAUCUUCAUGAUGCUGCAU | >Vv-miR172a*  AGAAUCUUGAUGAUGCUGCAU  >Vv-miR172b*  GAAUCUUGAUGAUGCUGCAG  >Vv-miR172c*  AGAAUCUUGAUGAUGCUGCAU  >Vv-miR172d*  AGAAUCUUGAUGAUGCUGCAU  >Vv-miR172e*  GGAAUCUUGAUGAUGCUGCAU |
| miR390 | >Vv-miR390a  AAGCUCAGGAGGGAUAGCGCC  >Vv-miR390b  AAGCUCAGGAGGGAUAGCGCC | >Vv-miR390a*  GCUAUCCCUCCUGAGCUUAA |
| miR393 | >Vv-miR393a  UCCAAAGGGAUCGCAUUGAUCC  >Vv-miR393b  UCCAAAGGGAUCGCAUUGAUCC | >Vv-miR393a*  AUCAUGCUAUCCCUUAGGA  >Vv-miR393b*  AAUGCGAUCCCUUUGGAU |
| miR394 | >Vv-mir394a  UUGGCAUUCUGUCAACCUCC  >Vv-mir394b  UUGGCAUUCUGUCCACCUCC  >Vv-mir394c  UUGGCAUUCUGUCCACCUCC  >Vv-mir394d  UUGGCAUUCUGUCCACCUCC  >Vv-mir394e  UUGGCAUUCUGUCAACCUCC | >Vv-mir394a*  AGGUGGGCAUACUGCCAA  >Vv-mir394b*  AGGCGGCCAGGAUGCCAA  >Vv-mir394c*  AGGUGGCCAGCAUGCCAAA |
| miR395 | >Vv-mir395a  UGAAGUGUUUGGGGGAACUC  >Vv-mir395b  CUGAAGUGUUUGGGGGAACUC  >Vv-mir395c  CUGAAGUGUUUGGGGGAACUC  >Vv-mir395d  CUGAAGUGUUUGGGGGAACUC  >Vv-mir395e  GAGUUCCCCCAAACACUUCAG  >Vv-mir395f  CUGAAGUGUUUGGGGGAACUC  >Vv-mir395g  CUGAAGUGUUUGGGGGAACUC  >Vv-mir395h  CUGAAGUGUUUGGGGGAACUC  >Vv-mir395i  CUGAAGUGUUUGGGGGAACUC  >Vv-mir395j  CUGAAGUGUUUGGGGGAACUC  >Vv-mir395k  CUGAAGUGUUUGGGGGAACUC  >Vv-mir395l  CUGAAGUGUUUGGGGGAACUC  >Vv-mir395m  CUGAAGUGUUUGGGGGAACUC  >Vv-mir395n  CUGAAGUGUUUGGGGGAACUC  >Vv-mir395o  CUGAAGUGUUUGGGGGAACUC  >Vv-mir395p  CUGAAGUGUUUGGGGGAACUC | >Vv-mir395a*  CUGAAGUGUUUGGGGGAACUC  >Vv-mir395b*  UGAAGUGUUUGGGGGAACUC |
| miR396 | >Vv-miR396a  UUCCACGGCUUUCUUGAACU  >Vv-miR396b  UUCCACAGCUUUCUUGAACU  >Vv-miR396c  UUCCACAUCUUUCUUGAACU  >Vv-miR396d  UUCCACAGCUUUCUUGAACUG  >Vv-miR396e  UUCCACAGCUUUCUUGAACU  >Vv-miR396f  CCACAGCUUUCUUGAACU  >Vv-miR396g  UUCCACAGCUUUCUUGAACUG | >Vv-miR396a*  UCAAGAAAGAUGUGGAAAA  >Vv-miR396b*  GUUCAAUAAAGCUGUGGGAA  >Vv-miR396c*  CUUCAGAAAGUUGUGGAACA  >Vv-miR396d*  GCUCAAGAAAGCUGUGGGA |
| miR397 | >Vv-miR397a  UCAUUGAGUGCAGCAUUGAU  >Vv-miR397b  UCAUUGAGUGCAGCGUUGAUG |  |
| miR398 | >Vv-miR398a  UGUGUUCUCAGGUCGCCCCU  >Vv-miR398b  UGUGUUCUCAGGUCACCCCUU  >Vv-miR398c  UGUGUUCUCAGGUCGCCCCU | >Vv-miR398a*  GUGUUCUCAGGUGCCACUCC |
| miR399 | >Vv-miR399d  UGCCAAAGGAGAUUUGCCC  >Vv-miR399e  UGCCAAAGGAGAUUUGCCC  >Vv-miR399f  UGCCAAAGGAGAUUUGCCC  >Vv-miR399g  UGCCAAAGGAGAUUUGCCC  >Vv-miR399h  GGGCAAAUCUCCUUUGGCA  >Vv-miR399i  UGCCAAAGGAGAUUUGCCC  >Vv-miR399j  UGCCAAAGGAGAUUUGCCC  >Vv-miR399k  UGCCAAAGGAGAAUUGCCCUG  >Vv-miR399l  UGCCAAAGGAGAAUUGCCCUG  >Vv-miR399m  UGCCAAAGGAGAUUUGCCC  >Vv-miR399n  UGCCAAAGGAGAGUUGCCCUG  >Vv-miR399o  GCCAAAGGAGAGUUGCCCUG  >Vv-miR399p  UGCCAAAGGAGAGUUGCCCUG | >Vv-miR399d*  UCUUUCUUGGCAGGCACU  >Vv-miR399e*  UUUCCACUAGAAGGAACU  >Vv-miR399f*  UCUCCUUUGGCAGUGAGCU  >Vv-miR399g*  CUCCAUUGGCAGUUGGCC  >Vv-miR399h*  UCUUUCUUGGCAGGCAGU  >Vv-miR399i*  GGGCCUCUUUCACUUGGUAGG |
| miR400 | >Vv-miR400  AUGAGGGUAUUAUAAGUCAC |  |
| miR403 | >Vv-miR403a  UUAGAUUCACGCACAAACUC  >Vv-miR403b  UUAGAUUCACGCACAAACUCG  >Vv-miR403c  UUAGAUUCACGCACAAACUCG  >Vv-miR403d  UUAGAUUCACGCACAAACUCG  >Vv-miR403e  UUAGAUUCACGCACAAACUC  >Vv-miR403f  UUAGAUUCACGCACAAACUC  >Vv-miR403g  UUAGAUUCACGCACAAACUC  >Vv-miR403h  UUAGAUUCACGCACAAACUCG  >Vv-miR403i  UUAGAGUCACGCACAAACUC  >Vv-miR403j  UUAGAUUCACGCACAAACUCG  >Vv-miR403k  UUAGAUUCACGCACAAACUC  >Vv-miR403l  UUAGAUUCACGCACAAACUCG | >Vv-miR403a*  UUUGUGCGUGAAUCUAA |
| miR408 | >Vv-miR408  AUGCACUGCCUCUUCCCUGGC |  |
| miR414 | >Vv-miR414a  UCAUCUUCAUCAUCAUCGUCA  >Vv-miR414b  UCAUCUUCAUCUUCAUCGUCA  >Vv-miR414c  UCAUCUUCAUCAUCAUUGUCA  >Vv-miR414d  UCAUCUUCAUCAUCACCGUC  >Vv-miR414e  UCAUCUUCAUCAUCAUCG  >Vv-miR414f  UCAUCUUCAUCUUCAUCGUC  >Vv-miR414g  UCAUCUUCAUCAUCGUCGUCA  >Vv-miR414h  UCAUCUUCAUCUUCAUCGUCA |  |
| miR773 | >Vv-miR773  UUUGCUACCAGCUUUUGUCUC |  |
| miR782 | >Vv-miR782  CAAACACCUUGAUGUUCUU |  |
| miR827 | >Vv-miR827  UUAGAUGAUCAUCAACAAAC |  |
| **G C A ---- U - A A U**  **5’ UUUCUU CUCAAAUGAGUAUU C AACAACA GC UGU GA GC AUGAU A**  **3’ GGGGAA GAGUUUACUCGUAG G UUGUUGU UG ACG CU UG UACUG C**  **G A - UAGG U U C A U**  pre-miR827 | | |
| miR828 | >Vv-miR828  UCUUGCUCAAAUGAGUAUUCCA |  |
| **- C GUUA UUUA**  **5’ UGU UUUGUUG UGGUCAUCUAGUCAUU GUCAUG \**  **3’ ACA AAACAAC ACUAGUAGAUUAGUAG CGGUAC A**  **C U GA-- UCGC**  pre-miR828 | | |
| miR846 | >Vv-miR846  UUGAAUUGAAGGCUUGAAUU |  |
| **UG U GAAA AU CU**  **5’ GUGUUAUGUGG AUAGUUCAA GUU UUGAUU GGCA \**  **3’ UAUAGUGUACU UAUUAAGUU CGG AGUUAA UCGU C**  **UA - A--- GU AU**  pre-miR846 | | |

Sequences of the mature miRNA and the corresponding miRNA* identified in *V.vinifera* genome found in our study. For the newly identified miR827, miR828, and miR846 a stem-loop structure supports the BLAST alignment. The fold-back structure was predicted using mfold software [7]. The mature miRNA is highlighted in red.
