# Supplementary material for: A High Quality Draft Consensus Sequence of the Genome of a Heterozygous Grapevine Variety
Source: PLoS One. 2007 Dec 19;2(12):e1326. doi: 10.1371/journal.pone.0001326 (PMC2147077; doi:10.1371/journal.pone.0001326)
Supplement: Table S10. — Number of identified tRNAs containing specified anticodons compared with the corresponding numbers of Arabidopsis. (0.07 MB DOC) [file pone.0001326.s017.doc]

**Table S10**. Number of identified tRNAs containing specified anticodons compared with the

corresponding numbers of Arabidopsis.

| **Isotype** | **Anticodon counts** | | | | | | | | **Total** | **Total for Arabidopsis** |
| --- | --- | --- | --- | --- | --- | --- | --- | --- | --- | --- |
| Ser | AGA  14 | GGA  3 | CGA  3 | TGA  16 | ACT | GCT  11 |  |  | 47 | 64 |
| Arg | ACG  11 | GCG | CCG  4 | TCG  5 |  |  | CCT  8 | TCT  9 | 37 | 36 |
| Leu | AAG  11 | GAG | CAG  6 | TAG  12 |  |  | CAA  18 | TAA  5 | 52 | 42 |
| Ala | AGC  14 | GGC | CGC  4 | TGC  41 |  |  |  |  | 59 | 33 |
| Gly | ACC | GCC  23 | CCC  6 | TCC  17 |  |  |  |  | 46 | 41 |
| Pro | AGG  13 | GGG | CGG  4 | TGG  22 |  |  |  |  | 39 | 66 |
| Thr | AGT  12 | GGT  4 | CGT  3 | TGT  12 |  |  |  |  | 31 | 24 |
| Val | AAC  17 | GAC  4 | CAC  11 | TAC  7 |  |  |  |  | 39 | 30 |
| Phe | AAA | GAA  24 |  |  |  |  |  |  | 21 | 16 |
| Asn | ATT  1 | GTT  24 |  |  |  |  |  |  | 25 | 16 |
| Lys |  |  | CTT  20 | TTT  12 |  |  |  |  | 32 | 31 |
| Asp | ATC  5 | GTC  25 |  |  |  |  |  |  | 30 | 26 |
| Glu |  |  | CTC  23 | TTC  15 |  |  |  |  | 38 | 25 |
| His | ATG  1 | GTG  16 |  |  |  |  |  |  | 17 | 10 |
| Gln |  |  | CTG  15 | TTG  8 |  |  |  |  | 23 | 17 |
| Ile | AAT  12 | GAT |  | TAT  6 |  |  |  |  | 18 | 24 |
| Met |  |  | CAT  34 |  |  |  |  |  | 34 | 24 |
| Tyr | ATA | GTA  19 |  |  |  |  |  |  | 19 | 76 |
| Cys | ACA | GCA  17 |  |  |  |  |  |  | 17 | 15 |
| Trp |  |  | CCA  13 |  |  |  |  |  | 13 | 14 |

All searches were carried out using the tRNAscan-SE program [8].
