# Supplementary material for: A High Quality Draft Consensus Sequence of the Genome of a Heterozygous Grapevine Variety
Source: PLoS One. 2007 Dec 19;2(12):e1326. doi: 10.1371/journal.pone.0001326 (PMC2147077; doi:10.1371/journal.pone.0001326)
Supplement: Table S11. — Number of genes identified in the V. vinifera genome for each of the nine families of snRNAs compared to Arabidopsis. (0.04 MB DOC) [file pone.0001326.s018.doc]

**Table S11**. Number of genes identified in the *V. vinifera* genome for each of the nine

families of snRNAs compared to Arabidopsis.

| **snRNA** | ***Vitis vinifera*** | ***Arabidopsis thaliana*** |
| --- | --- | --- |
| U1 | 14 | 14 |
| U2 | 26 + 2 fragments | 18 |
| U4 | 6 | 11 |
| U5 | 16 | 14 |
| U6 | 21 | 13 |
| U11 | - | 1 |
| U12 | 2 | 1 |
| U4 atac | 1 | 1 |
| U6 atac | 1 | 2 |
| **Total** | **89** | **75** |

The genes were identified by searching for sequences similar to the Arabidopsis snRNAs [9] using the Match program (Myriad Genetics, Salt Lake City, Utah) with manual revision.
