# Supplementary material for: A High Quality Draft Consensus Sequence of the Genome of a Heterozygous Grapevine Variety
Source: PLoS One. 2007 Dec 19;2(12):e1326. doi: 10.1371/journal.pone.0001326 (PMC2147077; doi:10.1371/journal.pone.0001326)
Supplement: Table S12. — Number of genes included in the different snoRNA families identified in the V. vinifera genome by searching against Arabidopsis snoRNAs [10]. (0.10 MB DOC) [file pone.0001326.s019.doc]

**Table S12.** Number of genes included in the different snoRNA families identified in the *V. vinifera* genome by searching against Arabidopsis snoRNAs [10].

| **snoRNA** | ***Vitis*** | **Arab.** | **snoRNA** | ***Vitis*** | **Arab.** | **snoRNA** | ***Vitis*** | **Arab.** |
| --- | --- | --- | --- | --- | --- | --- | --- | --- |
| snoR1 | 1 | 2 | snoR53 | 0 | 1 | snoR103 | 3 | 1 |
| snoR3 | 1 | 0 | snoR58 | 2 | 2 | snoR104 | 2 | 1 |
| snoR4 | 0 | 2 | snoR59 | 0 | 2 | snoR105 | 0 | 1 |
| snoR5 | 2 | 2 | snoR64 | 0 | 1 | snoR106 | 0 | 1 |
| snoR6 | 3 | 3 | snoR65 | 0 | 1 | snoR107 | 1 | 1 |
| snoR7 | 3 | 2 | snoR66 | 0 | 1 | snoR108 | 0 | 1 |
| snoR8 | 0 | 2 | snoR67 | 1 | 1 | snoR109 | 0 | 1 |
| snoR9 | 1 | 2 | snoR68 | 1 | 2 | snoR110 | 0 | 1 |
| snoR10 | 2 | 2 | snoR69 | 1 | 1 | snoR111 | 2 | 1 |
| snoR11 | 2 | 1 | snoR72 | 0^1^ | 5 | snoR112 | 1 | 1 |
| snoR12 | 2 | 3 | snoR73 | 0 | 1 | U3 | 5 | 0 |
| snoR13 | 1 | 2 | snoR74 | 14 | 2 | U14 | 10 | 4 |
| snoR14 | 2 | 2 | snoR75 | 0 | 1 | U15 | 3 | 3 |
| snoR15 | 1 | 1 | snoR76 | 0 | 1 | U16 | 2 | 1 |
| snoR16 | 2 | 2 | snoR77 | 0 | 4 | U18 | 1 | 2 |
| snoR17 | 1 | 1 | snoR78 | 1 | 2 | U19 | 0 | 1 |
| snoR18 | 2 | 2 | snoR79 | 0 | 1 | U24 | 0 | 2 |
| snoR19 | 1 | 2 | snoR80 | 1 | 1 | U27 | 4 | 1 |
| snoR20 | 1 | 2 | snoR81 | 0 | 1 | U29 | 1 | 1 |
| snoR21 | 0 | 2 | snoR82 | 1 | 1 | U30 | 0 | 1 |
| snoR22 | 1 | 4 | snoR83 | 1 | 1 | U31 | 2 | 2 |
| snoR23 | 1 | 3 | snoR84 | 0 | 1 | U33 | 2 | 2 |
| snoR24 | 4 | 4 | snoR85 | 0 | 1 | U34 | 1 | 3 |
| snoR25 | 3 | 1 | snoR86 | 1 | 1 | U35 | 1 | 1 |
| snoR26 | 2 | 1 | snoR87 | 0 | 1 | U36 | 2 | 4 |
| snoR27 | 2 | 1 | snoR88 | 1 | 2 | U37 | 2 | 1 |
| snoR28 | 2 | 5 | snoR89 | 0 | 1 | U38 | 2 | 3 |
| snoR29 | 4 | 2 | snoR90 | 11 | 1 | U43 | 1 | 2 |
| snoR30 | 3 | 1 | snoR91 | 0 | 1 | U49 | 2 | 3 |
| snoR31 | 2 | 1 | snoR92 | 1 | 1 | U51 | 3 | 2 |
| snoR32 | 4 | 2 | snoR93 | 1 | 1 | U53 | 0 | 1 |
| snoR33 | 1 | 1 | snoR94 | 0 | 1 | U54 | 2 | 1 |
| snoR34 | 0 | 1 | snoR95 | 1 | 1 | U55 | 1 | 1 |
| snoR35 | 0 | 1 | snoR96 | 0 | 1 | U56 | 2 | 1 |
| snoR36 | 1 | 1 | snoR97 | 0 | 1 | U61 | 2 | 1 |
| snoR37 | 1 | 3 | snoR98 | 0 | 1 | U65 | 2 | 3 |
| snoR38 | 1 | 2 | snoR99 | 0 | 1 | U79 | 2 | 1 |
| snoR39 | 0 | 2 | snoR100 | 0 | 1 | U80 | 1 | 2 |
| snoR41 | 1 | 1 | snoR101 | 0 | 1 |  |  |  |
| snoR44 | 0 | 3 | snoR102 | 0 | 1 | **Total** | **166** | **190** |

^1^ The sequences of snoR72 and snoR5 were almost identical and were assigned to the snoR5 family.
